# Possible future scenarios of the general health social security system in Colombia for the year 2033

**DOI:** 10.1186/s40309-022-00213-7

**Published:** 2023-02-10

**Authors:** Pedro Leon Cruz Aguilar, Javier Enrique Medina Vásquez

**Affiliations:** 1 Facultad de Ciencias Económicas y Empresariales, Universidad Santiago de Cali, Calle 5 No. 62-00 Cali, Valle del Cauca Colombia; 2 Facultad de Ciencias de la Administración, Universidad del Valle, Calle 4B # 36-00 Cali, Valle del Cauca Colombia

**Keywords:** Future scenarios, Health system, Primary health care and insurance, Intuitive logic, Stakeholder analysis

## Abstract

This article formulates possible future scenarios for the Colombian General Health and Social Security System (GHSSS) in 2033 when it reaches its 40th anniversary. The research draws on intuitive logic from the School of Human and Social Foresight (Masini, Penser le futur, 2000). The approach considers that in contexts of high instability and high conflict of interests, a combination of methods is required for the analysis of relevant transformations. It entails applying methods based on evidence, expertise, interaction, and creativity as discussed by Keenan and Popper (Foresight 10:16–36, 2008). Three scenarios are devised with the participation of real actors representing a plurality of visions from the public, private, academic, and social sectors. Finally, two future scenarios are featured, the one chosen by the experts before the triumph of the current government and the one that the Ministry of Health will present to the Colombian Congress for approval in 2023.

**Supplementary Information:**

The online version contains supplementary material available at 10.1186/s40309-022-00213-7.

## Introduction

In Colombia, health is a fundamental right. Act 100 of 1993 created the General Health and Social Security System (GHSSS from now on), which introduced a market of regulated competition, under the stewardship of the Ministry of the sector; its financing is accomplished through the Administrator of Resources of the General System of Social Security in Health (ADRES), which manages the resources to guarantee their adequate flow and the sustainability of the system. It is based on the capitation payment unit, which is the value that ADRES pays for each insured to the Benefit Plan Administration Entities (APB), in charge of the health insurance in two regimes: the contributory, for people with payment capacity, and the subsidized, for people without payment capacity. They, in turn, provide the insured with the health services contemplated in the basic health plan, which are contracted with Health Service Delivery Institutions (IPS), which are in charge of assisting users.

The GHSSS in Colombia is characterized by having 96% of the population insured and a high uncertainty manifested in the slow flow of resources, the difficult access of the insured to health services, the increasing demand for services due to chronic non-communicable diseases (NCDs) because of the aging of the population as well as the low interaction between the APB and the IPS and the users to carry out prevention and health promotion activities.

In the country, there is a lack of studies about the future of the GHSSS, where the voice of different stakeholders of the system is heard. In this context, scenarios facilitate the analysis of flexible, consistent, and shared future alternatives by the social actors, contributing to decision-making, and considering different perspectives and viewpoints. Despite the significant improvements of the GHSSS, this analysis is both relevant and necessary because there are crucial political and economic disagreements between the government and the opposition about: its quality, financing, stability, and the role to be played by the state and the private sector.

This paper aims to formulate possible future scenarios for the Colombian GHSSS to the year 2033, to increase the feasibility of public policies facilitated by participation and anticipatory governance [1]. Next, it presents a theoretical reference on the future scenarios, the methodology used, the system’s trends and drivers of change, and closes with the results, recommendations, and conclusions.

### Future scenarios

According to Martelli [2] and Van der Heijden, Bradfield, Wright, and Cairns [3], the scenario method emerged in the 1950s. It aims to analyze strategic options for a social system and could be defined in different ways as follows:

- Narrative descriptions of the future that focus attention on causal processes and decision points [4]. Its function is not to predict a single future but to create interpretations of different future alternatives. It is considered a tool to make better decisions in situations of rapid change and where multiple factors interact in a complex way, so as to lower the level of uncertainty and reduce the margin of error [5].

Cruz and Medina [6] reviewed 2603 abstracts of articles from indexed journals published between 2003 and 2013 and concluded that there is neither a universal pattern nor a unique methodology for scenario development. Different ways of doing scenarios coexist because they focus on different themes, such as the techno-economic, territorial, corporate, educational, or socio-cultural aspects, which involve different mixes of quantitative, qualitative, and semi-quantitative methods. Hence, in each case and context a specific theme, appropriate to the system under study, must be chosen and designed.

According to Amer, Daim, and Jetter [7], an appropriate way to understand the diversity of techniques is to recognize the three main methodological perspectives for the formulation of future scenarios: intuitive logic, probabilistic modified trends (PMT) methodology, and strategic foresight (*La Prospective*). The first two originated in the USA and England, while the third one was in France. See Table 1.

**Table 1:** Comparison of the principal scenario development techniques

Scenario characteristics	Intuitive logic methodology	***La prospective*** methodology	Probabilistic modified trends (PMT) methodology
Purpose	Multiple, from a one-time activity to make sense of situations and developing strategy, to an ongoing learning activity.	Usually, a one-time activity associated with developing more effective policy and strategic decisions.	A one-time activity to make extrapolative predictions and policy evaluations.
Scope	Can be either broad or narrow, ranging from global, regional, country, and industry to a specific issue.	Generally, a narrow scope but examines a broad range of factors within that scope.	Scope is narrowly focused on the probability and impact of specific events.
Methodology type	Process-oriented approach, essentially subjective and qualitative.	Outcome-oriented approach, which is directed, objective, quantitative, and analytical relying on complex computer-based analysis and modeling.	Outcome-oriented approach, very directed, objective, quantitative, and analytical using computer-based extrapolative simulation models.
Tools	Generic tools like brainstorming, STEEP analysis, and stakeholder analysis.	Proprietary and structural tools like Micmac, SMIC, and Mactor analysis, etc.	Proprietary tools like trends impact and cross-impact analysis, etc.
Developing scenarios set	Defining the scenario logic as organizing themes or principles.	Matrices of sets of possible assumptions based on the key variables for the future.	Monte Carlo simulations create an envelope of uncertainty around base forecasts.
Evaluation criteria	Coherence, comprehensiveness, internal consistency, and novelty, supported by rigorous structural analysis and logic.	Coherence, comprehensiveness, and internal consistency tested by rigorous analysis; plausible and verifiable in retrospect.	Plausible and verifiable in retrospect.

*Source*: ([7], p. 28)

In this text, intuitive logic is adopted as the guiding perspective. Historically, Mazziota [8] states that from this approach, scenarios constitute a method of inquiry or investigation of the most relevant possible futures, as opposed to forecasting methods that tend to focus mainly on the most probable future. The classical methodological scheme comes from the work of Ian Wilson at the Stanford Research Institute and the corporate practices of the Shell Company, led by Pierre Wack, since the 1960s. Later, the successors of this tradition were authors such as Peter Schwartz, Kees van der Heijden, and others. Likewise, the school of human and social perspective, developed by Eleonora Barbieri Masini, and the school of metafutures, created by Sohail Inayatullah, are based on intuitive logic. Currently, Ramírez and Wilkinson [9] continue to enrich this approach at Oxford University. Similarly, new possibilities have been generated by Derbyshire [10, 11].

According to Martelli [12], intuitive logic refers to the application of a rule of coherence, drawn from a careful analysis of the problem to be analyzed, likewise resorting to social science models that seek to explain the future reality. For Wilson [13, 14], it is a way of making sense, through the construction of mental models, to make a balance between the known or predetermined elements and the uncertainties or unknown elements of a problematic situation. This requires formal logic and disciplined imagination, combining the analysis of facts and data with perceptions, through a structured methodology that requires the direct participation of decision-makers, external experts, and stakeholders or those responsible for the strategic decision to be analyzed.

van der Heijden [15] argues that this process-oriented perspective suggests that reality is socially constructed, through the conversation of social actors. Therefore, in this text, scenarios are assumed as a tool to multiply our knowledge about the possible futures that await us, stimulating creativity and questioning the status quo, contributing to improving the decision-making process [16, 17]. They seek to build parallel stories that anticipate the possible ways in which the future could develop, devised by actors of the system under study, using a methodological process developed for this purpose [18].

Future scenarios became an appropriate methodology for building future alternatives in organizations with high levels of complexity and uncertainty, such as health systems [19, 20]. The World Health Organization recommended this in 1990 [21]. The dynamic situation of the sector has generated a growing demand for medical care and new technologies, forcing health services to find innovative ways to cope with the new needs presented to users [22–25].

## Methodology

Now then, according to Masini and Medina [26], the human and social point of view leads to focus on the visions, values, and capacities of the social actors. This approach is fundamental because it analyzes determining aspects of the country from the point of view of human development, transcending the traditional perspective focused on economic growth and the financial equilibrium of the GHSSS. In addition, this framework incorporates socio-cultural elements, better addressing the criticisms that point out the failures of care for people and the conflicts of perception between service users and service providers. The methodological process can be done through the following stages (Table 2):

**Table 2:** Methodological process for the formulation of future scenarios

Stage	Activity	Results	Methods
Building of a database	Identifying the achievements, capabilities, and values of the GHSSS	Description of Colombia’s GHSSS	Literature review. Population projections with system dynamics
Delimitation of the system and of its general context	Focusing on the main dimensions of analysis		
Description of the system and its components	Identification of drivers	Drivers’ analysis: Environmental Institutional Technological	Literature review: Semi-structured interviews to identify and classify the key factors affecting Colombia’s GHSSS
	Identification of stakeholders and strategic decisions	Level of importance of values Perception of the political power of stakeholders	Defining GHSSS stakeholders and the questionnaire for the in-depth interview with categories contributing to identify the changes and the possible future alternatives, lasting for one hour.
	Identification of key variables by a group of decision makers	Identification of the factors of breakdown and issues emerging	Twenty-two in-depth interviews were conducted with different stakeholders, transcribed, and coded using the Atlas.ti software according to the categories identified a priori
Formulation of possible future scenarios and the scenario to be achieved	Create sets of questions and hypotheses for each scenario	Scenario validation workshop and formulation of the scenario to be achieved	Reflection exercise and in-depth analysis of the responses already coded to capture the existing relationships, detecting emerging phenomena in the topic of study, and questioning the status quo of the ideas obtained in the literature review.
	Develop the scenarios around the key variables and actors		Identification of critical uncertainties for the development of future scenarios through the answers given in the interviews. Generation of the narrative for each of the future scenarios based on the selected axes and the interviewees’ answers.
Required actions for the implementation of the chosen scenario		Recommendations	Aims and strategies for achieving the chosen scenario

*Source*: Masini and Medina [26], O’Brien and Meadows [27], and Cruz and Medina [28]

**Fig. 1:**
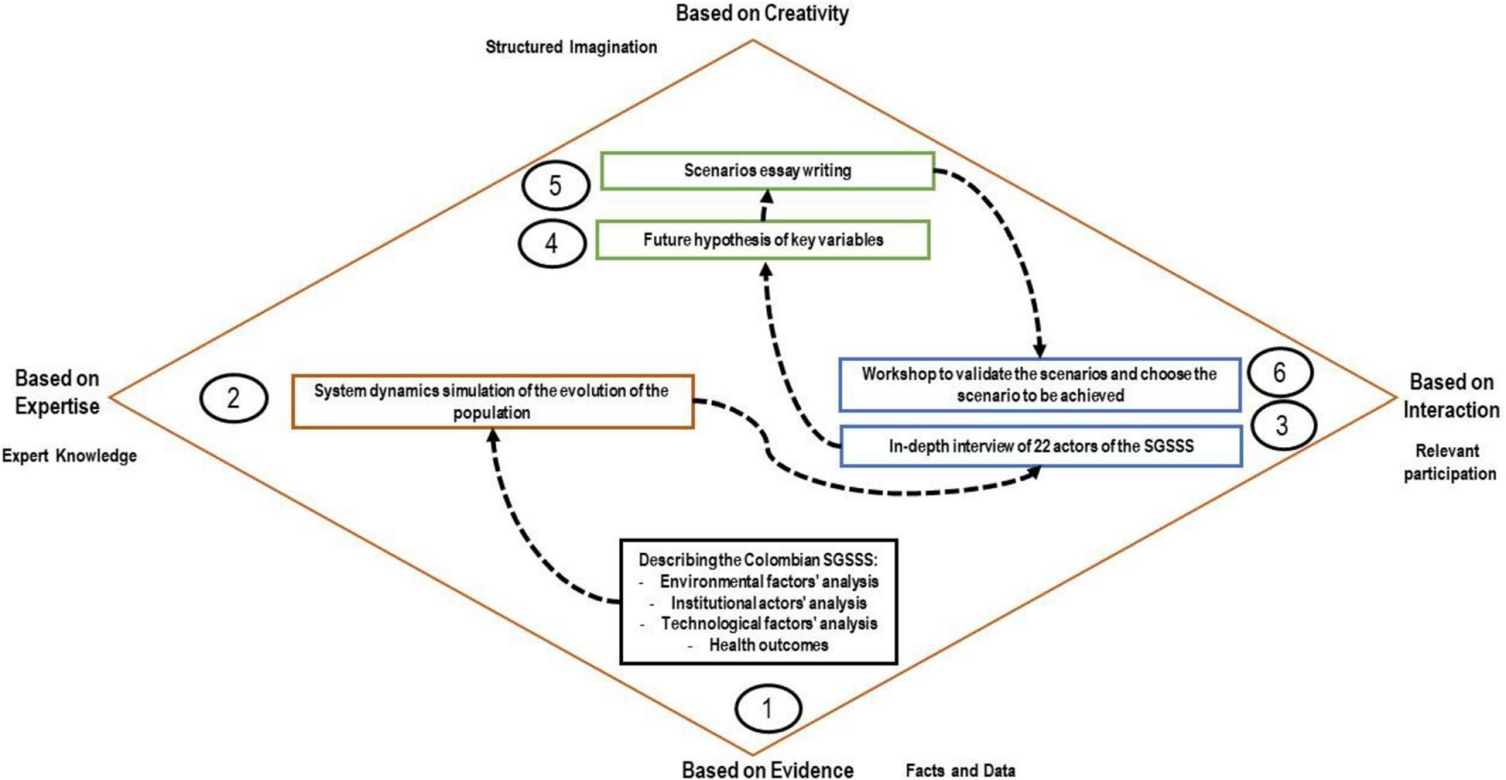
Logical diagram for the combination of methods. Source: Adapted from Cruz and Medina [28] and Popper [29]

This methodological framework can be visualized as shown in Fig. 1.

### Delimitation of the system and of its general context

OECD’s health at a glance edition served as a template for the description of the Colombian health system, adding the dimensions of demographics, health research and ICTs, coverage, access, and values, which are one feature of the Human and Social Perspective (Table 3).

**Table 3:** Dimensions of the Health Social Security System in Colombia

Factors	Dimension	Key variables
Environment	Demographics	Aging population
	Determinants of health	Population
		Access to drinking water
		Access to basic sanitation services
		Prevalence of overweight and obesity in adults
		Prevalence of alcohol consumption
		Prevalence of tobacco use
		Prevalence of psychoactive substance use
	Financial protection	Total health expenditure per capita
		Share of health care out-of-pocket spending
		Health expenditure as a percentage of GDP
Technology	Capacities of the GHSSS	Health insurance coverage
		Hospital beds per 1000 population
		Nurses per 1000 population
		Psychiatrists per 1000 population
		Pregnant women receiving prenatal care (%)
	Health research and ICTs	Level of health research
		Vaccination dependency
		Telemedicine consultations
Institutional	Health status	Life expectancy at birth
		Mortality rate in children under 1 year of age
		Mortality rate in children under 5 years of age
		Mortality rate due to NCDs
	Coverage and access	Health insurance coverage level
		Health care access
	Health inequality	Difference between central and peripheral regions for the following:
		Physicians per 1000 people
		Infant mortality rate
		Difference between the highest and lowest quintile:
		Under-five mortality rate (per 1000 people)
		COVID-19 mortality rate (per 10,000 people)
	Quality of care	Childhood immunization programs
		Cancer survival rate
	Values	Values that an activity must have to be called humane:
		Freedom
		Equality
		Solidarity
		Respect
		Dialogue

The interviewed GHSSS stakeholders represent the different sectors of society, namely the public and the private sectors, civil society, and academia. They were recruited to have a complete overview of the system (Table 4).

**Table 4:** List of interviewees

No.	Sector	Position
1	Public sector	Head of the National Health Institute
2		Senator
3		Senator
4		Health Secretary of the Municipality of Cali
5	Private sector	Head of the Imbanaco Medical Center
6		CEO of Emssanar EPS
7		CEO of Los Andes Clinic
8		Health Deputy Director of Comfandi
9		Marketing and Sales Manager at Versalles Clinic
10		Member of the Board of Directors of the Colombian Association of Hospitals and Clinics
11		Head of the Association of Pharmaceutical Laboratories, Research and Development
12	Civil Society	CEO of ECSIM Foundation
13		Project Manager at ECSIM Foundation
14		Head of the Colombian Association of Hepatic and Renal Patients
15		Coomeva-affiliated user
16		President of the union Sintrahospiclínicas at the HUV^a^
17	Academia	Head of the Doctorate degree in Public Health at Universidad Nacional de Colombia
18		Services Director at the Public Health Institute of the UPJ^b^
19		Head of the Public Health School, Universidad del Valle
20		Head of Internationalization of Universidad del Valle
21		Dean of the School of Health, Universidad Santiago de Cali
22		Head of the Pharmacology Research Group, Universidad del Valle

^a^ *HUV* Hospital Universitario del Valle. Valle’s University Hospital

^b^ *UPJ* Unidad Permanente de Justicia. Permanent Justice Unit

## Results

The results offer the description and the morphological analysis of the possible future scenarios for the next 10 years for the Colombian GHSSS.

### Description of the GHSSS and its components

Colombia has made progress in terms of health insurance coverage, covering 98.26% of the total population [30], where out-of-pocket spending represents 15.13% of the income. However, there are problems with access to services; one in four people who needed health services in 2019 could not access them [31], differences in both capacities and outcomes persist between the central and peripheral regions, and the main cause of mortality is the NCDs. The description of the system was based on the dimensions proposed in Table 3.

#### Demographics

Colombia had a population of 47,417,200 inhabitants in 2017, which increased steadily until reaching 51,609,474 in 2022, an upward trend expected to continue until 2064, when it will reach 63,197,004 inhabitants and will start a downward trend [32]. Projections using system dynamics indicate that, in the year 2033, the country will have 54,888,452 inhabitants, of which 12,755,270, equivalent to 23% of the population, will be over 60 years old, which will generate greater demand for medical services for NCDs, due to population aging.

#### Determinants of health

Access to drinking water and basic sanitation is paramount to improving health, contributing to the well-being of inhabitants, and keeping the population healthy. In Colombia, coverage of these services is increasing slowly. In 2000, 68% of the population enjoyed drinking water services while 71.7% had access to basic sanitation; in 2017, 73% while 89.63% enjoyed these services, respectively [33].

According to the 2019 national survey on the consumption of psychoactive substances, the lifetime prevalence of alcohol consumption in the population aged 12–65 years was 84%; tobacco, 33.3%; and psychoactive substances: marijuana, 8.3% and cocaine, 2.07% [34]. Unhealthy lifestyle habits contribute to increased disease burden.

Overweight and obesity have become a public health problem, as they are risk factors in multiple pathologies, such as hypertension, diabetes, high cholesterol, cardiovascular diseases, and in some types of cancer. From 2000 to 2017, the prevalence of overweight among adults increased from 46.7 to 58.6% and that of obesity from 14 to 22.1% [33].

#### Financial protection

Per capita health spending in Colombia has increased substantially. In current dollars, in 2000, it amounted to USD 132.88 and rose, in 2018, to USD 514.00. But it is still low compared to Latin American countries such as Uruguay (USD 1590) and Chile (USD 1456) [33]. In the same period, out-of-pocket health expenditure went from 13.68% as a percentage of current health expenditure to 15.13%, although it increased slightly, it is still lower than in Latin American countries such as Chile (33.24%), Argentina (27.73%), and Costa Rica (22.42%) [33].

Regarding current spending on health as a percentage of GDP, in Colombia, in 2018, it was 7.64%. In the same year, Latin American countries such as Argentina, Chile, and Uruguay invested 9.62%, 9.14%, and 9.20%, respectively, in health [33]. The largest proportion of this current expenditure on health is governmental (71.61%), as it is financed in most countries.

#### GHSSS capacities

With the pandemic, the number of beds in intensive care units doubled; before this event, there was a slight increase in the number of beds and low investment in public hospitals. In 2010, the country had 1.48 beds per 1000 inhabitants, and in 2017, it had 1.7. While in the same year (2017), countries such as Argentina and Cuba had 5 beds and Uruguay had 2.4 [33].

The number of physicians in the country has increased substantially from the period 1990 to 2017; it went from 1.02 physicians per 1000 people to 2.1, while countries such as Argentina and Chile, in 2017, had 4 and 4.88 physicians per 1000 people, respectively [35]. In the same period, nurses went from 0.42 per 1000 inhabitants to 1.27, a figure below that of countries such as Chile, which had 13.3; Cuba, with 7.7 and Costa Rica, with 3.18 [35].

Psychiatry specialists in 2017 amounted to 1003 professionals, equivalent to 0.021 per 1000 people [36]. In summary, there is an adequate number of general practitioners; however, most are concentrated in urban centers. The same is not true for nurses and specialists whose proportion is below WHO standards, and in specialties such as oncology and psychiatry, the situation is critical.

Care for pregnant women reduces the risk of infections during pregnancy and reflects the capabilities of a health system; during the period between 1990 and 2016, it increased from 82 to 97.2% [37], contributing to more babies being born healthy.

#### Research in health and information and communication technologies (ICT)

Health research lacks a shared future vision by the different actors of the system for the construction of knowledge. It is imperative to articulate researchers with companies in the health sector to work with research groups in the development of innovative proposals in applied research, promoting the creation of a structure to encourage the use of research results among the actors of the GHSSS [38].

Safe, effective, and appropriate medical technologies contribute to providing an adequate response to people who become ill. In Colombia, in 2019, there were per million inhabitants 1.2 CT scanners, 0.2 MRI units, 1.5 radiotherapy units, and 11.5 mammography units per million women between 50 and 69 years old, while the average in Latin America and the Caribbean was 8.3, 3.1, 1.4, and 110.4, respectively [39].

During the pandemic, the Ministry of Health issued Resolution 521 on March 28, 2020, which considers the provision of health services through telemedicine and support between health professionals digitally, which increased consultations by this means substantially and is expected to continue this upward trend.

#### Health status

Life expectancy in Colombia between 2000 and 2019 increased from 73.75 years to 79.31 years for both sexes: for men, from 69.71 years to 76.69 years, and for women, from 77.85 years to 81.87—an increase of 5.56 years, 6.98 years, and 4.02 years, respectively [33].

The opposite is true for the infant mortality rate. In 1960, for every 1000 live births, 94.27 died before reaching one year of age; in 2019, this figure decreased to 11.84 children, and the under-five mortality rate, in 1960, for every 1000 live births 135.2 died before reaching 5 years of age, and in 2019, this figure decreased to 13.8 [33]. While the mortality rate caused by NCDs is increasing, the mortality rate for ischemic diseases increased from 61.37 deaths per 100,000 inhabitants in 2005 to 79.32 in 2019; in the same period, deaths due to breast cancer increased from 8.53 per 100,000 inhabitants to 14.28.

#### Coverage and access

In Colombia in 2005, 78.49% of the total population was affiliated with an insurance company. In 2020, 98.26% of the population had health insurance coverage [30]. The problem lies in access to services; in 2019, barriers such as the timeliness of the appointment, the quality of the service, the geographical location of the care center, and the amount of paperwork prevented access to medical services for one in four people who needed them [31].

#### Health inequalities

Health inequalities are regional in nature between provinces located in the central zone versus those located in the peripheral zone, and socially between the lowest and highest strata. If the number of physicians per 1000 people is disaggregated by region, it is found that, in 2017, while in Bogotá, there were 4 physicians per 1000 inhabitants, in peripheral regions such as Putumayo, there were 0.96 physicians per 1000 people, in La Guajira, 0.92; in Chocó, 0.54; and in Vaupés, 0.37—that is less than one physician per 1000 inhabitants [36].

The same happens with the infant mortality rate; in this regard, we found that the infant mortality rate in 2019, in Bogotá, was 9.6 per 1000 live births; in peripheral regions such as Chocó, the infant mortality rate was 27.4 per 1000 live births; in La Guajira, 23.05; in Vaupés, 23; and in Vichada, 29.4 [40].

In the social sphere, the under-five mortality rate in 2016 presented a difference between the lowest and highest income quintile, of 20.3 deaths per 1000 live births. In 2015, in births attended by qualified health personnel, there was a difference between the lowest and highest income quintile, of 10.8%. In the mortality rate of COVID-19, accumulated between March 2020 and May 2021, a difference between socioeconomic strata is presented. In strata 1 to 3, the mortality rate was 18.4 deaths per 10,000 inhabitants and in strata 4 to 6, 14.7 [41].

#### Quality of care

The quality of care in a health system can be measured by aspects such as childhood vaccination. In 2019 in Colombia, the vaccination rate against diphtheria, tetanus toxoid, and pertussis (DTP3) in children around 1 year old reached 92%, and the coverage rate of the first dose of measles vaccine (MCV1) in 1-year-old children was 96% [33].

The effectiveness of a health system in providing care to cancer patients contributes to identifying and reporting avoidable inequalities, which can be assessed through the survival rates of patients diagnosed with each type of cancer. In adults aged 15–99 years, between 2010 and 2014, in Colombia, the 5-year net survival rate for breast cancer reached 72.1%, the 5-year net survival rate for colon cancer was 34.5%, the 5-year net survival rate for cervical cancer was 49.4%, and the 5-year net survival rate for rectal cancer was 32.7; while in Latin America, the averages were 78.4%, 51.7%, 59.5%, and 45.8%, respectively [39].

#### Values

One of the fundamental components of people’s well-being is health; thus, health systems must be humane and, as such, any activity to be called so must observe five values: freedom, equality, solidarity, respect, and dialog [42]. According to the stakeholders interviewed, these values should be practiced in the GHSSS in the following order of importance: equality first, followed by respect, solidarity, freedom, and dialog. Values should contribute to the humanization of the system.

### Future alternatives based on the morphological analysis

With the election of President Gustavo Petro in 2022, it becomes possible to migrate to a single, public, universal, and preventive health system (*Health for all*), proposed as an alternative to the *Integrated Territorial Action Model* advocated by previous governments. This means leaving three possible scenarios for the future of the GHSSS (Table 5).

**Table 5:** Morphological analysis of the GHSSS to 2033

Factors	Dimensions	If you can access, we will provide care for you	Integrated territorial action model	Health for all
Environment	Demographics	The trend is towards an aging population and a decrease in healthy years of life.	A marked aging trend prevails, the adoption of primary health care contributes to older adults enjoying more healthy years of life.	There is a marked aging trend; the adoption of a preventive health care model keeps sick older adults under control and detects ailments in the healthy ones at an early stage.
	Determinants of health	The Ministry of Health has a seat on the intersectoral commission on health determinants; however, it cannot integrate health actions with the activities carried out in the determinants.	The Ministry of Health has a seat on the intersectoral commission on health determinants. Because of the model’s territorial approach, in some regions, it has integrated the actions carried out in health with the activities carried out in the determinants.	The Ministry of Health coordinates the management of health determinants to articulate health actions with the activities carried out in the determinants.
	Financial protection	Out-of-pocket and per capita health spending lower than the average for Latin American countries. The sustainability of the system is threatened by the bankruptcy of the APB and the debt among actors.	Out-of-pocket and per capita spending on health is lower than the average for Latin American countries. The sustainability of the system is threatened by debt among stakeholders.	Out-of-pocket and per capita health expenditure better than the average of Latin American countries. Financed by taxes and contributions
Technology	GHSSS capabilities	Specialists, physicians, nurses, and the hospital infrastructure concentrate in urban centers, leaving peripheral regions without these services. The health staff is employed through intermediation.	The family health physician is the entry point to the system. The model’s territorial approach triggers a migration of health professionals from urban centers to some peripheral regions. The health staff is employed through intermediation.	The primary health teams based in the territory reach an equitable distribution of human talent and the system’s resources. All health workers are guaranteed decent, stable, and dignified work.
	Health research and ICTs	Low investment in health research and high dependence on vaccines and the number of telemedicine consultations is increasing.	Increased investment in science and technology to lower medicine costs and recover autonomy in vaccines. Increasing number of telemedicine consultations.	A knowledge-intensive system is promoted to access better treatments and devices. Autonomy in the production of vaccines and biologics is recovered. Health is in the cloud, with a single interoperable information system.
	Health status	Life expectancy close to the average of Latin American countries and NCDs are the leading cause of mortality.	Life expectancy better than the Latin American average, with a moderate increase in the NCD mortality rate.	Life expectancy close to the average of OECD countries, with a low increase in the NCD mortality rate.
	coverage and access	Insurance is universal, but access to health services has administrative, geographic, regulatory, and supply barriers.	Insurance is universal, but access to health services has administrative barriers to specialist appointments and procedures.	An integrated and comprehensive health network covers the population, with an extramural team based in the territory as an entry point to the system, guaranteeing the right to health regardless of the person’s payment capacity.
	Health inequities	Health inequalities between the center and the periphery of the country and the different social strata exhibit an upward trend.	The differential approach allows sectors in the periphery to benefit from better infrastructure and more health workers, thus reducing inequities among regions, although inequities among socioeconomic strata persist.	As extramural medical teams are installed in rural areas and in the most vulnerable sectors of the cities, the differences among regions and socioeconomic strata are reduced.
	Quality of care	Childhood vaccination rate close to the average for Latin American countries and survival rate at five years of age for cancers far below the average for other Latin American countries.	Childhood vaccination rate better than the average for Latin American countries, and survival rate at 5 years for cancers close to OECD countries	Childhood vaccination rate close to the average of OECD countries and survival rate to 5 years for cancers better than the average of Latin American countries.
	Values	The main value is equality, and the stakeholder with the greatest political power are the APBs.	The main value is equality, and the stakeholder with the greatest political power are the APBs.	The main value is dialog and the stakeholder with the greatest political power is the Ministry of Health.

Three possible scenarios can be developed from the futures hypotheses presented in Table 5.

### Scenario 1: If you can access, we will provide care for you

It is characterized by a multiple-insurance model with a curative care strategy, and its main objective is universal insurance with universal coverage. While life expectancy is increasing, healthy life years are decreasing due to NCDs, out-of-pocket spending is one of the lowest in Latin America, and the slow flow of resources has generated a billionaire debt from the insurers to the IPS, putting the sustainability of the system at risk. The care deficit is growing steadily due to the aging of the population, which demands more services. Health inequities between territories and socioeconomic strata are deepening. During the pandemic, telemedicine received a significant boost, and dependence on vaccine production became evident. The main value is equality.

### Scenario 2: Integrated territorial action model

It is characterized by having multiple insurance models with a PHC strategy, with a family health physician at the head of a multidisciplinary team as the point of contact with the user. Its fundamental purpose is to guarantee the right to health in the territories. The decrease in the prevalence of NCDs increases life expectancy and healthy life years, out-of-pocket expenses are among the lowest in Latin America, the number of PHCs has decreased to ten, the high indebtedness of these with the IPS persists, the deficit in specialized physician’s visit shows an upward trend, inequities in health between regions and social classes show a downward trend, investment in science and technology and consultations by telemedicine is increasing, and the country remains dependent on the production of vaccines. The main value is equality.

### Scenario 3: Health for all

It is a public, unique, universal health system with a preventive care model, whose purpose is to guarantee the right to health through the coordination of actions on the determinants of health and the activities of the system. Its entry point is the extramural teams that are part of an integrated and comprehensive health network that will provide timely and quality care to each of the inhabitants affiliated. The Ministry of Health will manage the health system under the leadership of the national health council, which will be in charge of the political coordination and governance of the system. The health secretariats and territorial health councils will guide the formation of integrated and comprehensive health services networks. The ADRES, as a single health fund, will collect taxes and health contributions, contracting, and paying health services to the IPS. Health will be in the cloud with a unique and interoperable information system. The central value is the dialog that will strengthen the trust between the actors to build the desired future where Colombians will have the health they wish to have.

### Role of the actors performing GHSSS functions

It is crucial to describe the role of these GHSSS actors because they are the ones who carry out the functions of direction and governance, financing, insurance, and service delivery to guarantee the right to health Table 6.

**Table 6:** Role of the main GHSSS stakeholders

Role	If you can access, we will provide care for you	Integrated territorial action model	Health for all
Ministry of Health	It is responsible for guiding the health system and for formulating, adopting, directing, coordinating, executing, and evaluating public health policy.		It is responsible for the direction, governance, and implementation of public policies issued by the National Health Council.
Single Health Fund	Administrator of the GHSSS resources: resource collection and recognition and payment of the UPC to insurance companies.		It runs the system through the collection of health taxes and contributions, contracting and paying for health services.
Insurance Companies	Benefit Plan Administrators: manage the health risk and hire the provision of basic plan services with the IPS.	Benefit Plan Administrators: manage the health risk and hire the provision of basic plan services with the IPS, in a designated territory.	Disappear
Territorial Health Secretariats	To progressively guarantee the population’s access to health services.		It coordinates the creation of comprehensive and integrated health networks and public health programs.
Service providers	Health service delivery institutions: they care for the sick.	Health service delivery institutions: serve users with an interdisciplinary team led by a physician specializing in family health. Through a PHC strategy.	Public and private service providers make up an integrated and comprehensive regional health network, with extra-mural teams for active search, early detection, and work with the community based on the territory, which is the entry point to the system.
Users	Health insured, affiliated to the different health insurance regimes.		They are part of a comprehensive and integrated health network, entering the system through the extra-mural teams.

### Actions required for the implementation of the chosen scenario

A group of experts worked on and validated the scenarios in a workshop. They considered the *Integrated Territorial Action Model* and the *Health for All* scenarios, at the end opting for the *Integrated Territorial Action Model* scenario, as it was deemed more plausible for implementation.

- To provide financial resources to implement the PHC strategy, which emphasizes health promotion (education) and disease prevention (early detection) to keep the population healthy. This implies the creation of interdisciplinary teams, led by a family health specialist.
- Establish the necessary incentives so that universities (in their undergraduate and specialization programs) offer programs that teach the PHC strategy so that students feel motivated to take them.
- Invest in public hospital infrastructure and the incorporation of ICTs into the provision of health services, starting with the single electronic medical record lodged in the cloud.
- Provide territorial health institutions with the capacity to lead the coordination of intersectoral activities that intervene in the social determinants of health.

With President Petro in office, the Ministry of Health will choose and implement the *Health for All* scenario in the country. To do so, it must:

- Submit a bill to the Congress (which will be done in February 2023) to approve the new health model.
- Prioritize the PHC in the budget and add to ADRES the functions of contracting and paying for health services.
- Set a single interoperable information system.
- Form integrated and comprehensive health networks in each region to which users will be affiliated, so as to guarantee people’s access to health services nearby their places of residence.
- Establish extra-mural active search teams, which are the entry point to the territorial preventive health program and who would progressively cover the whole territory, starting with the peripheral areas and the most vulnerable sectors.
- Guarantee the right to health (Act 1751/2015).
- Guarantee health workers dignified, safe, and decent jobs.
- Align preventive health program actions with health determinants activities.

## Conclusions

The methodology combined different methods, drawing from an approach based on intuitive logic. It facilitated the analysis of the interaction of the stakeholders and their future visions, being appropriate for characterizing the reasons for conflict and possible solutions. This human and social perspective is considered relevant for analyzing social systems such as the GHSSS, characterized by intense tension between ideologies and frames of reference. The scenario approach enabled the definition and characterization of the GHSSS, the analysis of different strategic options, and the proposition of plausible recommendations.

From the three possible future scenarios proposed, the experts chose the *Integrated Territorial Action Model*, as it had the highest plausibility level to date. However, the new progressive government promotes the *Health for All* scenario, which implements a public, single, preventive health system coordinating actions on social determinants with those carried out in the system. Affiliation replaces insurance with integrated and comprehensive health networks with extra-mural teams linked to the region that will progressively cover the entire territory. The function of contracting and paying for the services provided will be transferred to the single health fund. Access to services will be timely and of high quality regardless of the income level of the person requesting them. And there will be a single interoperable system in the cloud.

Changes in health invite transformative ways of thinking about the health system, breaking with the status quo of the prevailing structures. It gives the health authority back to the Ministry, granting it control over the contracting and provision of health services through the single health fund and to the territories over the establishment of integrated and comprehensive health networks (previously in the hands of the insurance companies).

The political tension between the scenario proposed by the experts and the scenario developed by the new office illustrates the conflict between a technical and a political vision that seeks to accelerate radical transformations in the GHSSS. The former focused on institutional stability, and the latter focused on the institutional response to people with less access, quality, and timeliness of care. The future development of the GHSSS relies on these different visions and values. Reality will prove whether the new office has the necessary capacities to change the system effectively.

## Supplementary Information

**Additional file 1.**

## Data Availability

The database searched are the following: Así Vamos en salud: https://www.asivamosensalud.org/indicadores/estado-de-salud Banco Mundial: https://datos.bancomundial.org/ DANE: https://www.dane.gov.co/index.php/estadisticas-por-tema Observatorio de Talento Humano en Salud: www.sispro.gov.co/observatorios/talento-humano/indicadores/Paginas/Indicadores.aspx SISPRO: https://www.sispro.gov.co/Pages/Home.aspx WHO Global Health Observatory: https://www.who.int/data/gho

## Abbreviations

ADRES: Administrator of Resources of the General System of Social Security in Health
DANE: Departamento Administrativo Nacional de Estadísticas (National Administrative Department of Statistics)
EPS: Empresas promotoras de salud (health promotion agencies)
GSSHS: General Health Social Security System
IPS: Instituciones prestadoras de servicios de salud (health service delivery institutions)
NCDs: Chronic non–communicable disease
OECD: Organization for Economic Co-operation and Development
PHC: Primary health care
PMT: Probabilistic modified trends
USD: United State dollar
WHO: World Health Organization
