# Supplementary material for: Possible future scenarios of the general health social security system in Colombia for the year 2033
Source: Eur J Futures Res. 2023 Feb 10;11(1):1. doi: 10.1186/s40309-022-00213-7 (PMC9911950; doi:10.1186/s40309-022-00213-7)
Supplement: Supplementary file 1 — Additional file 1. [file 40309_2022_213_MOESM1_ESM.rtf]

Lista códigos-citas
Código-filtro: Todos
______________________________________________________________________

UH:	Vision de futuro_2033_d
File:	 [C:\Users\pelec\OneDrive\Documentos\Trabajo de grado_docto...\Vision de futuro_2033_d.hpr7]
Edited by:	Super
Date/Time:	2022-11-11 08:20:12
______________________________________________________________________

Código: Calidad en la atención : sistema de Gestión de la Calidad {25-0}

P 9: 2. Transcripcion Alex Duran_ok.docx - 9:20 [Que mucho es carreta, que es u..]  (107:107)   (Super)
Códigos:	[Calidad en la atención : sistema de Gestión de la Calidad] 
No memos

Que mucho es carreta, que es un tema documental y que no ha creado todavía la forma en que la gente perciba que usted tiene un sistema de calidad en algún área, me explico, tú no puedes hablar de un sistema de calidad exitoso si tienes mortalidad materna, con una sola muerte quiere decir que tus sistema no es bueno, mientras que la gente haga cola tu sistema de calidad no es bueno, tú me puedes documentar de que atienden a la gente, pero lo que me esta documentando es lo mal que suceden las cosas, mientras existan las quejas por autorizaciones y todo eso, usted puede decir que tengo el sistema de calidad este u otro, pero mientras el ciudadano no perciba que usted obtuvo algo no se puede hablar de un sistema calidoso

P11: 4. Transcripcion Armando Gonzales_ok.docx - 11:20 [Y lo hicimos así porque nosotr..]  (109:109)   (Super)
Códigos:	[Calidad en la atención : sistema de Gestión de la Calidad] 
No memos

Y lo hicimos así porque nosotros pensábamos que una frase corta era más fácil alinear a toda la gente dentro de esa frase, que todo el mundo cada vez que esté realizando un acto médico esté pensando en la frase, más que la misión misma. Y eso lo adaptamos desde el año 2002, decidimos crear la parte de garantía y calidad en salud, que la arrancamos aquí a raíz del libro, "errar es humano" entonces empezamos a trabajar en la seguridad del paciente y en eso siempre ha sido un líder. 

P11: 4. Transcripcion Armando Gonzales_ok.docx - 11:21 [Pues yo tengo una percepción m..]  (113:113)   (Super)
Códigos:	[Calidad en la atención : sistema de Gestión de la Calidad] 
No memos

Pues yo tengo una percepción muy cualitativa sobre eso, porque diríamos que los dos sistemas finales están implementados de manera irregular, el componente de la auditoria médica es un componente que se ha tergiversado mucho, y se ha llevado más a la auditoria de cuenta, que a la auditoria del acto clínico. Entonces diríamos que las instituciones han ponderado y priorizado más la parte financiera, que el resultado clínico como tal. Y lo segundo es que el sistema de información no ha sido incluso bien desarrollado por el ministerio, apenas el año pasado salió una resolución acerca de eso y aun no se ha implementado muchísimo en el país, el cuarto componente que es el de sistema de información de la calidad, que al final lo que busca es que haya un ranking hospitalario, en Colombia por servicios y por hospitales, pero eso todavía está sin resolverse. 

P11: 4. Transcripcion Armando Gonzales_ok.docx - 11:22 [Es incompleto, y fuera de eso ..]  (117:117)   (Super)
Códigos:	[Calidad en la atención : sistema de Gestión de la Calidad] 
No memos

Es incompleto, y fuera de eso lo que esta completo no funciona bien, no funciona con el objetivo que tiene, un ejemplo la política de seguridad del paciente dice que alrededor del evento, del error que suceda en una clínica se reúnan médicos, profesionales, clínicas, aseguradores, pacientes  para que todos aprendamos, y resulta que eso no se puede implementar porque al hacer eso, el asegurador dice que al ser un evento adverso y como eso fue un error suyo, entonces yo no le voy a pagar, entonces eso desvirtúa totalmente el objetivo de la política

P11: 4. Transcripcion Armando Gonzales_ok.docx - 11:16 [DL: El sistema fue empezado en..]  (121:121)   (Super)
Códigos:	[Calidad en la atención : sistema de Gestión de la Calidad] 
No memos

DL: El sistema fue empezado en el 93, primero fue el decreto 2174 que fue en el 96, luego lo cambio por el 1011 que fue en el 2006, y llevamos todos estos años y uno ve que esto no avanza, porque la calidad y de hecho usted ve que el ministerio mira la calidad, y para el ministerio no tiene prioridad, usted nunca oye hablar al ministro de calidad usted lo oye siempre hablar es de plata y la parte financiera, cuando uno llama al ministerio y pregunta por el de calidad el de calidad ni siquiera pasa, de hecho a mí me invitaron a  presentar el modelo de Imbanaco que te conté, a Argentina y a Perú y allá va a estar Arias que es el de calidad del ministerio. Entonces uno no ve en el ministerio el espíritu porque en este país se hable de calidad eso no se nota, ellos están es empecinados en manejar la parte financiera no la parte de calidad, entonces yo decirte que en 20 años si se va a hablar de calidad en este país y va a ser prioritario para el ministerio pues yo no sé. Le voy a dar un ejemplo, para que usted lo mire, el reuso en este momento Colombia tiene un problema enorme al rededor del reuso de dispositivos que se usan varias veces en varios pacientes, el invima dice no reusen, pero los aseguradores no pagan, si yo uso este aparato como dice una sola vez no pagan lo que vale ese aparato solo una vez, ellos lo pagan al factor de usos, se reduce a tres o cuatro veces. Entones hay una presión del gobierno porque no haya reuso, pero no regulan el reuso, ni dicen a partir de ahora en adelante los dispositivos que sean de un solo uso, los aseguradores tienen que pagarlos como un solo uso, nadie asegura eso. Entonces hay un problema enorme alrededor del reuso en este momento en el país porque está pendiente de la plata y no de la calidad, no lo regulan porque hay muchos intereses económicos en el sector, para que cada vez la atención en salud cueste menos. 

P12: 5.Transcripcion Carlos Fajardo_ok.docx - 12:8 [el sistema de gestión de calid..]  (34:34)   (Super)
Códigos:	[Calidad en la atención : sistema de Gestión de la Calidad] 
No memos

el sistema de gestión de calidad, hay unas instituciones que se han demarcado y se han proyectado a darle cumplimiento a esos estándares, en Nariño tenemos unos ejemplos muy interesantes como son el hospital departamental, el hospital infantil, y esta el hospital de Ipiales, en el valle esta Imbanaco, la fundación valle del Lili, son instituciones que por sus sistemas de calidad garantizan y generan una confianza en la relación entre institución y la  EPS, pero igualmente le permite tener mucha confianza al usuario de ser atendido en esas instituciones, por eso muchas veces esto genera un sobrecosto natural, ya que quien no quiere que Ensanar tiene hoy la red con Imbanaco valle del Lili, los envié  a una institución pública que no está ni acreditada, y más bien viene la presión a querer ser atendidos en esas instituciones.

P12: 5.Transcripcion Carlos Fajardo_ok.docx - 12:9 [En relación de calidad es muy ..]  (38:38)   (Super)
Códigos:	[Calidad en la atención : sistema de Gestión de la Calidad] 
No memos

En relación de calidad es muy bueno y la presión de la gente para ser atendido en estos centros es alta , hay unas normas en la 1438 establecidas el 60% debe ser contratado con la red pública, en este caso con el HUV, este como tal no tiene un sistema de calidad en la prestación del servicio, hoy esta en la peor de sus crisis, y las instituciones de lo que usted me plantea del primer nivel que se evidencia mucho como fortalece en el ecuador, acá no existe, acá existen unas instituciones donde lo único que hacen es remitir al segundo y tercer nivel y hay unas complejidades que se puede decir que se vuelve monopolio, trasplantes HUV no hace entonces hay que recurrir a la fundación valle del Lili, o tratamiento con niños sobre todo de oncología y de canceres, los está trabajando Imbanaco. Entonces ha tocado buscar como una garantía para que las personas que padecen esas enfermedades sean tratadas directamente en instituciones con una calidad de mayor competencia. 


P12: 5.Transcripcion Carlos Fajardo_ok.docx - 12:21 [Yo creo que sigue en manos de ..]  (134:134)   (Super)
Códigos:	[Calidad en la atención : sistema de Gestión de la Calidad] 
No memos

Yo creo que sigue en manos de unos pocos, yo no lo veo el interés del gobierno porque no ha visto programas que realmente estén fortaleciendo la red pública, porque el privado si buscan mucho innovar y cumplir ciertos principios de calidad o ciertos parámetros de calidad que le permiten trascender y ser mejores. Pero en programas que sean institucionales, de buscar que estas instituciones públicas mejoren en procesos de calidad no le veo que haya claridad en programas verdaderamente por parte del gobierno acerca de sacarlo adelante en ese aspecto.

P13: 6 y 7.Transcripcion Diego Gomes y Gonzalo Gonzales_ok.docx - 13:30 [Yo creo que en eso hemos aumen..]  (167:167)   (Super)
Códigos:	[Calidad en la atención : sistema de Gestión de la Calidad] 
No memos

Yo creo que en eso hemos aumentado muchísimo y hay hospitales que son extraordinariamente buenos en eso, ahora viene lo mismos, los otros hospitales que no están aplicando y no lo han hecho, es un problema que no lo tengamos y es una falta de capacidad de la superintendencia de exigirlo y de la secretaria seccionomica de exigirlo, pero creo que en eso hemos avanzado muchísimo y tenemos hospitales magníficos, no solo en el aseguramiento de procesos, en la parte de seguridad del paciente se ha avanzado extraordinariamente.

P16: 10.Transcripcion Angela Tascon.docx - 16:11 [Me parece que es un derecho ba..]  (46:46)   (Super)
Códigos:	[Calidad en la atención : sistema de Gestión de la Calidad] [modelo de salud actual] 
No memos

Me parece que es un derecho bandera y que dijéramos todo el norte tiene que ser ese, el norte del sector tiene que ser ese porque realmente ahí está la igualdad ahí está la solidaridad, ahí está la oportunidad, a mí eso como teórico es excelente pero ahí hay un término muy importante que es la calidad y que de verdad dijéramos el gobierno exige bastante al respecto pero no está el medio para que se dé entonces por eso hay tantas cosas que es lo que uno ve en los noticieros, tantos eventos adversos tantas cosas porque no es prioritario y no se eso en que parte quepa pero por ejemplo la calidad que no debería de ser un costo, más bien un beneficio la calidad vale mucho porque el sector o el gobierno, no facilita entonces el hecho de que usted este certificado acreditado, el gobierno trata de sobresaltar eso, no tiene mucho respaldo porque no hay leyes que favorezcan eso que es lo que muchas veces se ha solicitado y pretendido que haya un tratamiento de tipo de impuestos, o de tipo de exportación de insumos o algo, para estas entidades que logran estos escalones de excelencia y de calidad. Entonces pues lastimosamente no es congruente la exigencia con las facilidades que se dan, pero para mí ese decreto transversal al secto

P16: 10.Transcripcion Angela Tascon.docx - 16:22 [Pues yo diría que la tendencia..]  (90:90)   (Super)
Códigos:	[Calidad en la atención : sistema de Gestión de la Calidad] 
No memos

Pues yo diría que la tendencia es buena, es como a mejorar los controles que se están haciendo a nivel de secretaria me parecen que son buenos, faltan pero yo creería que todas las entidades estamos con ese focos de tener un sistema de gestión de calidad no de papel si no operativo y bueno, y pues hemos crecido en eso, tenemos clínicas importantes a nivel internacional muy posicionadas muy respaldadas por la parte privada como es el caso de la fundación, sabemos muy bien que la fundación está muy respaldada en el sector privada y que cuando se quiere se puede. Pero también hay mucha permicidad y están las clínicas de garaje que ocasionan estos eventos adversos que se presentan, pero yo creería que la voluntad y la parte de calidad está muy interiorizada en todas las instituciones, es que definitivamente si los servicios no se prestan con calidad usted va perdiendo sus clientes, el sector yo diría que está caminando algunos más lentos que otros pero si se siente más.

P16: 10.Transcripcion Angela Tascon.docx - 16:23 [No, el gobierno no, ha ofrecid..]  (94:94)   (Super)
Códigos:	[Calidad en la atención : sistema de Gestión de la Calidad] 
No memos

No, el gobierno no, ha ofrecido desde el primer gobierno de Uribe ofrecieron estas famosas zonas francas, no se dieron, han ofrecido mecanismos de tipo impositivo de impuestos para las instituciones que tengan estas distinciones, que han escalado en calidad pero no hay estimulo del gobierno en eso, que digan las instituciones que están certificadas o que tengan algún premio de excelencia yo sé que están las grandes pero dos, tres clínicas muy posicionadas acá en la ciudad luchan impresionante con respecto a los recursos porque el gobierno no premia eso.

P16: 10.Transcripcion Angela Tascon.docx - 16:24 [Porque el privado tiene papá p..]  (98:98)   (Super)
Códigos:	[Calidad en la atención : sistema de Gestión de la Calidad] 
No memos

 Porque el privado tiene papá por decirlo de alguna manera tiene jefe, cuando las cosas tienen al jefe a alguien a quien le duela directamente pero el gobierno es muy etéreo, es un papá. El privado fuera de eso tiene más continuidad, usted ve un gerente cuantos años tiene el gerente allí, mientras tanto en el público hay muchos cambios, se paran muchos compromisos, por eso el privado tiene por lo menos el foco de dar una rentabilidad de sostenerse, al público no le importa. Entonces por eso el privado lleva la delantera en eso, pero los casos exitosos de los públicos son de verdad demostrables, el hospital de Pasto es una belleza porque lo cogieron unos líderes éticos, empoderados del servicio como al de entrega y ahí son los casos puntuales que uno dice si se puede. Habría que mirar a ver y copiar el gobierno, yo digo que hay ejemplos a seguir, el gobierno podría entrar a investigar a implementar y promocionar, pero uno ve que un hospital se acredita y quien se emociona, quien saca la cara, quien se enorgullece, el gobierno no, el sector privado es quien se muestra feliz acá en el valle por el posicionamiento del Valle del Lili, que distinción del gobierno, no he visto polo menos, no hay incentivo, no se involucra tampoco, son instituciones que el gobierno hiciera un alto y se resaltara esas cosas que la hicieron brillar para que se vuelvan públicas y sirvan de ejemplo, puede hasta llegar a uno a desmotivarlo.

P16: 10.Transcripcion Angela Tascon.docx - 16:25 [No yo creo que sigue aumentand..]  (102:102)   (Super)
Códigos:	[Calidad en la atención : sistema de Gestión de la Calidad] 
No memos

No yo creo que sigue aumentando, porque los ejemplos así sean muy puntuales, nos muestran a los que estamos acá en la barrera mirando que si se puede ser exitoso prestando servicios con calidad, entonces a mí me parece que es algo lento pero que si va en ascenso y que esta parte normativa en la parte de seguridad del paciente por decirlo así que es el eje transversal en este momento de la acreditación, si esa exigencia sigue así, la gente se mete en el cuento o desaparece. Entonces yo creo que a pesar de todos los negativos y a pesar de que no hay incentivo y demás tiende a ser una convicción. 

P18: 11. Transcripcion Fabian Mendez.docx - 18:24 [Digamos que creo que el sistem..]  (104:104)   (Super)
Códigos:	[Calidad en la atención : sistema de Gestión de la Calidad] 
No memos

Digamos que creo que el sistema funciona para lo que es de alto costo, por la forma de cómo está concebido el modelo hay unos indicadores que uno dice el sistema funciona, uno dice por experiencia propia o por otros que hay un interés porque desde las EPS los prestadores, de responder con calidad frente a estas enfermedades de alto costo porque son rentables y aquí yo digo que la cosa es relativamente buena, pero cuando usted se va a calidad del día a día, del paciente hipertenso crónico, diabético crónico, desafortunadamente los indicadores no son de producto si no indicadores de proceso, yo le doy esta población usted me tiene que responder por la población de ellas, sino yo le doy esta población y usted me tiene que al final responder con cuantas consultas dio cuantas vacunas aplico. Es una cosa de proceso pero finalmente no de resultados y allí es donde yo digo que necesitamos un cambio fundamental en la forma en que se evalúa la calidad, hacia resultados. Se ha tratado, se ha dicho que ese es el cambio que tiene que dar el sistema, pero yo veo que todavía la superintendencia tiene problemas muy grandes, la capacidad resolutiva de la superintendencia, las violaciones que hay, el cumplimiento de los requisitos mínimos de las IPS, solamente digamos cuando uno mira los exabruptos que pasa con el sistema es que se da cuenta que está mirando el pico del iceberg de un problema de calidad muy grande. Entonces yo creo que mientras dentro de este sistema sin cambia el modelo mientras tengamos una superintendencia que tiene que estar apagando incendios, hay una capacidad resolutiva  muy limitada donde hay además unos poderes económicos muy grandes, es muy difícil que la calidad realmente apunte a productos y está por eso muy dependiente de procesos. Pero no realmente si la salud de las personas mejora o no que ese es verdaderamente el verdadero producto, a mí no me interesa cuantas consultas le hace a un hipertenso a mí me interesa es que la presiona arterial está controlada, a mí no me debería interesar si estoy pensando en calidad, cuanta atenciones le hace a un diabético, lo que me interesa es que la hemoglobina glicolisada este bajo control. Lo que pasa es que un modelo medicalizado y sin visión integral va a ser muy difícil pensar en indicadores de calidad más acorde s a una visión de salud ligada a bienestar, ligada a ma allá de lo exclusivamente médico

P18: 11. Transcripcion Fabian Mendez.docx - 18:25 [Supuestamente lo que debería a..]  (108:108)   (Super)
Códigos:	[Calidad en la atención : sistema de Gestión de la Calidad] 
No memos

Supuestamente lo que debería apuntar es hacia allá, hacia una calidad basada en productos más que en procesos eso es lo que todos esperamos que pase, y no creo que evoluciones hacia allá. Es decir se necesitaría que realmente el derecho a la salud tal como está establecido en la ley estatutaria entraría a ser defendido y que prevaleciera, el asunto es que en todo este modelo prevalece es el interés económico sobre el derecho a la salud y eso hace que la calidad no sea precisamente que el modelo le interese perfeccionar mecanismos donde la calidad digamos con esa visión integral se alcance, entonces yo a futuro veo esos dos escenarios, el escenario de una defensa real de la salud que pudiera entonces amarrarse a proceso e gestión de calidad reales amarrados a productos, versus un escenario como el tendencial de ahora con el mismo mecanismo una superintendencia y un sistema de calidad allí que coloca herramientas y frente a los problemas pone remedios pero son paños de agua tibia de un asunto estructural muy grande. 

P19: 12. Transcripcion Fabio Osorio.docx - 19:16 [Yo no sé si lo podría comparar..]  (78:78)   (Super)
Códigos:	[Calidad en la atención : sistema de Gestión de la Calidad] 
No memos

Yo no sé si lo podría comparar con los entes de control de Colombia, todos los hospitales deben tener un sistema de calidad en atención pero acá lastimosamente en el hospital nos pasó que hubo un sistema de calidad, que empezó muy bien pero lastimosamente se burocrático y se llenó de gente, porque aprovecharon ese espacio pa meter gente y pagar favores políticos. El sistema de gestión de calidad como ley es excelente si se cumple no habría ningún problema pero sí creo que debe haber un seguimiento hasta desde el mismo portero que atiende a la gente con calidad, hasta la misma enfermera que lo chuza para sacarle una muestra de sangre, hasta el mismo doctor que decide operarlo, si este sistema de calidad está enfocado, ingresa el paciente hasta el mismo que le dice usted tiene salida estos son sus derechos y esta es la cuenta, si este sistema de gestión de calidad es transversal en todos los aspectos seria magnifico, que se invierta en ese sistema pero que no se burocratice y que se aplique como dice la norma, la norma es bonita pero se convierte en otra muerte cuando no se aplica.

P26: 16. Transcripcion Senador Ospina.docx - 26:15 [Yo creo que no, que son esfuer..]  (62:62)   (Super)
Códigos:	[Calidad en la atención : sistema de Gestión de la Calidad] 
No memos

Yo creo que no, que son esfuerzos muy puntuales en determinadas instituciones que se hacen como debe ser, creo que cuando muchas instituciones han dejado de pagar oportunamente a su trabajador o de adquirir insumos con oportunidad, tener obsolescencia tecnología, lejos estaremos hablando de un modelo responsable en calidad.


P29: 18. Transcripcion Carlos Hernandez.docx - 29:16 [CH: El sistema de calidad es f..]  (73:73)   (Super)
Códigos:	[Calidad en la atención : sistema de Gestión de la Calidad] 
No memos

CH: El sistema de calidad es fundamental lo que me incomoda de los sistemas de calidad como procesos para certificación,  y lo que debería hacer es un proceso de acreditación no de sacar egos, si no de acreditarse ante la ciudadanía, yo debería tener en Colombia un sistema de calidad y el reto es decir si yo pudiera contestar estas preguntas yo diría estoy montando un sistema de calidad, en que clínica en Colombia tiene la menor tasa de fallos en reemplazos articulares, cual es el porcentaje de éxito en cirugía de tumor cerebral cual es la tasa de complicaciones en pacientes de cuatrerismo cardiaco, cual es el mejor centro de mayor numero de infartos tasa de sobreviva a 10 años en infarto en Colombia. Porque lo que está en la mente de la gente es un nivel, está habilitada para prestar el servicio. Esta certificada, pero es eso lo que nosotros necesitamos, cuando usted se sube a un avión a usted le dicen tiene el 98% de que no se caiga, este tiene el 70% usted donde se sub, pero a la población en salud le están diciendo eso. Entonces cuando usted esta montado en un sistema de calidad está bien, yo creo que el tema de acreditación ese es un tema internacional está diciendo que allá le va a ir bien, porque tiene muy bien estandarizado los temas, pero la información a la gente no le llega en el sentido de si era cirugía plástica, estos son los 10 mejores centros, cuando uno va a un hotel sabe lo que está pagando, cuando va a uno d8% de que no se caiga, este tiene el 70% usted donde se sub, pero a la población en salud le están diciendo eso. Entonces cuando usted esta montado en un sistema de calidad está bien, yo creo que el tema de acreditación ese es un tema internacional está diciendo que allá le va a ir bien, porque tiene muy bien estandarizado los temas, pero la información a la gente no le llega en el sentido de si era cirugía plástica, estos son los 10 mejores centros, cuando uno va a un hotel sabe lo que está pagando, cuando va a uno de 4 hace un sacrificio, pero en Colombia la estrella es el paciente, tu asumes el riesgo, no sabes a donde estas entrando estas entrando a un sistema donde lo confortable es motivo de satisfacción, como me sucedía cuando trabajaba en el seguro social, ósea que cuando tienen un cáncer bien llevado no hubiera sido mejor que digan porque me detecto temprano el cáncer. No me voy del seguro porque allá le hacen la diálisis a mi papa, y eso es carísimo y la pregunta que yo me hago es esa diálisis pudo haber sido evitable, él pudo vivir 20 años con hipertensión sin necesidad de diálisis. Ese es el problema que tenemos que el sistema no le ha generado conciencia ciudadana, que a lo mejor no es la consulta especializada que lo mejor es que usted vaya a un post que le detectan tempranamente los riesgos y no es el médico especialista, no vamos a encontrar a un oftalmólogo haciendo examen ginecológico a una mujer, ningún ginecólogo le hace un examen de ojo al paciente. Entonces la caridad no es un tema de muros ni de confort o que el proceso esté bien descrito, para mí la calidad es un tema de resultados de salud y uno debería de tener la idea de entrar a una entidad donde el resultado va a ser bueno, eso no está en discusión en este momento, son sistemas que le ayudan a copiar información del que lo está haciendo bien pero aún le falta otro componente, quienes lo están haciendo mejor y los resultados de salud se ven reflejados en indicadores de salud de la población. Son clínicas muy buenas motivo de orgullo pero no te está diciendo que en la ciudad de Cali los pacientes de infarto eran evitables, si los pacientes con complicaciones eran evitables. Las clínicas son buenas pero yo quisiera un sistema de salud que digiera si usted entra a eta entidad le van a diagnosticar muy temprano el cáncer  y no va a tener que ir a tantas clínicas tan buenas que hay. Yo prefiero que exista una conciencia ciudadana. Que es lo que uno necesita en un sistema de calidad, no sea un tema de institución eso lo tenemos que hacer, pero un tema de conciencia ciudadana es porque yo me voy a hacer con esta aseguradora para tener un mejor resultado en salud, eso no está en la mente de la gente. Resultados en salud es yo soy diabético y no he tenido que ir a  la diálisis. 

P31: 20.Transcripcion Hellen Mendoza.docx - 31:9 [lo que quería agregarle del si..]  (27:27)   (Super)
Códigos:	[Calidad en la atención : sistema de Gestión de la Calidad] 
No memos

 lo que quería agregarle del sistema de gestión de la calidad, nosotros hemos hecho avances importantes en los sistemas de gestión de calidad y hoy tenemos habilitación, tenemos acreditación, tenemos muchas cosas que nos permiten disminuir el riesgo que teníamos en 20 o 30 años, si usted mira nosotros hemos tenido bloques de crecimiento la 1122 que salió en el 2011, fue una de las primeras cuando salió la ley fue la primera modificación de la ley 100 levanto la mano y dijo si es cierto que debemos tener atención en salud, tenemos que garantizar cobertura, pero la cobertura no se garantiza sola, se garantiza con unos índices de calidad que tienen que ser a su vez medio con un indicadores y con ciertas cosas que se fueron reglamentando mediante fueron saliendo otras leyes y otros decretos reglamentarios posteriores. Pero que está pasando, hay una desigualdad en ,la aplicación del sistema de gestión de calidad, hay unas clínicas a las que les aplican todos los métodos, habilitación acreditación, todas las normas, nos miden los espacios los arquitectos con metro, para garantizar que la pintura no sea toxica lo cual es muy bueno, pero hay clínicas que están en Cali y en otras regiones del país que no cumplen habilitación y todo el mundo lo sabe y sin embargo allá no llega la secretaria, y cuando la secretaria llega nunca pasa nada. 


P31: 20.Transcripcion Hellen Mendoza.docx - 31:11 [HM: pero hay otras cosas que v..]  (31:31)   (Super)
Códigos:	[Calidad en la atención : sistema de Gestión de la Calidad] 
No memos

HM: pero hay otras cosas que van más allá y si ellos quisieran podrían establecer otros mecanismos de presión, la habilitación va muy de la mano con infraestructura, cuando empezó la habilitación la primera vez que salió iba muy de la mano con infraestructura, hojas de vida, protocolos y recurso humano, poco a poco la norma fue evolucionando hoy tenemos indicadores de calidad y estamos obligados a presentar el PAME , cuando usted tiene los indicadores de calidad de una clínica y usted tiene los indicadores de una clínica B, sabiendo que en la clínica B hay reinfección, hay reingreso, hay infecciones, hay estancias hospitalarias prolongadas, usted está viendo que el indicador no corresponde con la realidad algo está pasando. Entonces por más que se preste en el equipo por más que legue el contratista y preste su equipo hay algo que usted no puede ocultar y no puede negar y es la realidad d unos indicadores de calidad.

P31: 20.Transcripcion Hellen Mendoza.docx - 31:12 [HM: Acreditarse cuesta pero cu..]  (43:43)   (Super)
Códigos:	[Calidad en la atención : sistema de Gestión de la Calidad] 
No memos

HM: Acreditarse cuesta pero cuando salió la acreditación si usted se va a la norma primaria de acreditación se vendió con el concepto de que las clínicas y los hospitales que tuvieran acreditados iban a tener un plus en facturación pero el gobierno no cumplió y entonces cuánto vale hacer acreditación, es muy costoso pero lo costoso ni siquiera es la implementación de la acreditación lo costoso es sostener la acreditación y garantizar a cada visita de acreditación usted va subiendo el estándar y cada vez que eleva el estándar es supremamente costo de garantizar y entonces si a usted le están pagando hoy a 6 meses a usted le toca financiar la plata, le toca conciliar sus cuentas para poder tener un poquito de flujo para poder garantizar el pago de proveedores y el pago de recurso humano y todo lo que usted necesita para funcionar, y adicional tiene que hacer un pago adicional para poder garantizar que está acreditado, donde queda la sostenibilidad financiera de las instituciones. Y si usted mira ya nos ha pasado, que paso con DIME, es muy difícil que salga de la situación en la que esta y están haciendo esfuerzos importantes y la gente que está allí es gente supremamente capacitada, el profesor Varela está al frente de DIME y está haciendo un gran esfuerzo, pero el problema es que de nada sirve el esfuerzo por un equipo cuando del otro lado el asegurador no le está reconociendo y no le esa pagando. Y si usted va y mira que paso con la clínica de san Fernando se acabó se la comió el sistema y muchas otras instituciones que se han venido quedando en el tiempo porque la sostenibilidad no existe, entonces todos existen todos deben estar pero si usted mira hoy, hay un pecado en cada uno, hay un pecado en cobertura en la unidad de pago por capitación, no es suficiente para pagar lo que se necesita, hay un pecado en la capacidad instalada porque hoy tenemos. 

P31: 20.Transcripcion Hellen Mendoza.docx - 31:14 [HM: Pero mire que en el exteri..]  (51:51)   (Super)
Códigos:	[Calidad en la atención : sistema de Gestión de la Calidad] 
No memos

HM: Pero mire que en el exterior no le interesa la acreditación colombiana, lo que le está pidiendo a usted es la acreditación del Joint Comisión, lo que pasa es que para poder llegar al Joint Comision ellos arrancan con la acreditación colombiana, pero usted la acreditación colombiana la pone en estados unidos y la pone en Europa y no es reconocida es un sistema de acreditación en Colombia para Colombia. Con que vende fundación, con la Joint comisión. 

P31: 20.Transcripcion Hellen Mendoza.docx - 31:15 [HM: Es que la Joint Comision e..]  (55:55)   (Super)
Códigos:	[Calidad en la atención : sistema de Gestión de la Calidad] 
No memos

HM: Es que la Joint Comision es más exigente que la acreditación colombiana entonces uno por donde arranca, arranca por los procesos de calidad arranca con habilitación que es teóricamente lo más básico de habilitación, me voy para la ISO como cuando usted quiere hacerlo por pasos, y de ISO ya tengo un pasito porque ya tengo todos los procesos estandarizados y acreditados por la ISO, entonces y atengo brinco para la acreditación, y de acreditación la misma fase para llegar a Joint Comision, y entrar a la Joint Comision de una es una cosa besti

P31: 20.Transcripcion Hellen Mendoza.docx - 31:16 [HM: Vale la pena que usted mir..]  (59:59)   (Super)
Códigos:	[Calidad en la atención : sistema de Gestión de la Calidad] 
No memos

HM: Vale la pena que usted mire unos casos, mire cuantas IPS estaban acreditadas en Colombia hace 10 años y cuantas IPS están acreditadas en Colombia hoy, y como se ha ido perdiendo la acreditación y como algunas IPS han tomado la decisión de dejar perder entre comillas la acreditación.

______________________________________________________________________

Código: Capacidades del sistema : accesso a la salud {31-0}

P 9: 2. Transcripcion Alex Duran_ok.docx - 9:17 [Pues uno muy fácil de responde..]  (87:87)   (Super)
Códigos:	[Capacidades del sistema : accesso a la salud] 
No memos

Pues uno muy fácil de responder es la geográfica, no en Cali pero si en Colombia y una de las razones que nosotros desarrollamos telemedicina cuando fui gerente, porque en Colombia salvo ciertos territorios tenemos privilegios de acceso geográficos, Cali es uno de ellos, pero es la excepción, le voy a poner el ejemplo del pacifico colombiano, usted solamente tiene dos formas de salir del pacifico colombiano por carretera en dos puntos, no tiene más, usted o sale por buenaventura o sale por Tumaco y en una población tan extensa que va desde el golfo de panamá, hasta el ecuador en un país como Colombia si usted solo puede salir por tierra en dos puntos, por buenaventura si no lo coge un derrumbe o dos si se somete a la precariedad de la autopista Tumaco - Pasto. Y dígame si usted es un paciente y se enferma en ese territorio como sale entonces fiamos que el acceso es una realidad de apuño en este país, pero el otro que repercute mucho en salud es el tema yo le digo socio medioambiental, para ponerle un ejemplo Miranda Cauca, está a solo 40 minutos de Cali, en la parte plana, ni siquiera está en la loma ni nada, pero usted nunca a encontrar un especialista ahí, ni en Corinto ni en Pradera, de pronto en Florida, porque uno antes por el conflicto armado, dos porque el especialista no se te va a vivir  Miranda, porque Miranda no le ofrece nada a la familia del especialista, si usted quiere ir a verse una película o a un centro comercial necesita algo y no lo encuentra, entonces digamos que el acceso en Colombia esta mediado por esos temas, y por eso todo el mundo vive en Cali, y en Cali encuentra especialista de lo que usted quiera, pero a solo 30 minutos o 40 no tienes nada. 

P 9: 2. Transcripcion Alex Duran_ok.docx - 9:18 [yo sueño con la primera IPS vi..]  (91:91)   (Super)
Códigos:	[Capacidades del sistema : accesso a la salud] 
No memos

 yo sueño con la primera IPS virtual en Colombia, ósea que nosotros tengamos una IPS que pueda cubrir toda Colombia, ese es un sueño que tengo allá e incluso como iniciativa propia que espero algún día empezar a desarrollar que es crear una IPS que pueda prestarle a usted un servicio en Leticia, en la guajira en Tumaco, eso se puede hacer, bajo la forma tradicional no, porque hacer infraestructuras es complejo, pero si se puede

P 9: 2. Transcripcion Alex Duran_ok.docx - 9:19 [Y por eso insisto tanto en el ..]  (95:95)   (Super)
Códigos:	[Capacidades del sistema : accesso a la salud] 
No memos

Y por eso insisto tanto en el tema, para no ir muy lejos Buenaventura, que no es tan dramático como en Timbiqui que allá tenemos telemedicina, Buenaventura, en la medida que los territorios entiendan que el tema de salud no se va a solucionar desde el tema de prestación de servicios de salud, las condiciones de vida de la gente van a cambiar, para eso la Organización Mundial de la Salud, ha implementado el tema de salud en todas las políticas, STP, y es como cuando se diseña el plan de desarrollo y se tiene en cuenta estos componentes hace que el territorio cambie, me explico, si tu quieres cambiar las condiciones en salud en Buenaventura por ejemplo, hoy en día el principal componente al cual debes invertirle en salud es al de educación y cuando me refiero eduación no me refiero solamente a acceder a primaria o bachillerato, si no a lo que se le enseña a los niños en estos colegios, la autodeterminación, el autocuidado, el día que en Buenaventura hagan eso, los indicadores de salud van a variar, antes no, porque puedes tener la mega clínica puesta en medicina pero si no le enseñas a la gente que el estado no todo te lo da, ni todo te lo resuelve, cosas como la violencia, el embarazo en adolescentes, proyecto de vida y todos eso, eso no va a cambiar, pero a la respuesta concreta que si existe herramientas si, si no que desde la planificación se debe pensar en la salud no como un resultado.

P 9: 2. Transcripcion Alex Duran_ok.docx - 9:21 [Y no soy versado en el tema, s..]  (115:115)   (Super)
Códigos:	[Capacidades del sistema : accesso a la salud] 
No memos

Y no soy versado en el tema, si algo le tengo como prevención son a las nuevas aplicaciones, pero mire no hay nada más innovador que la necesidad, de verdad cuando uno está apretado es que uno piensa como resolver los problemas y yo pienso que una de mis fortalezas es no perder la sensibilidad por la gente, entonces cuando la gente se manifiesta en uno y centra las esperanzas en uno, la gente está esperando que uno medianamente resuelva, y lo que hago es como revisar que en el mundo, ha dado resultados y lo apropio, eso técnicamente seria innovación pero en el dicho popular seria buscándole la comba al palo, y yo pienso que siempre hay que tratar de tener actitud propositiva, porque si bien lo de telemedicina esta normado, por el país, en Cali funciona por fuera del sistema, nosotros hoy no tenemos telemedicina en el régimen contributivo, así no tengamos contrato con eso, toca funcionar por fuera del sistema, si usted me dice cuanto le cobramos a usted por una consulta de telemedicina, nosotros nos la inventamos, le vamos a cobrar 70 mil pesos, eso no está en el sistema entonces digamos que no es el chicle por la tecnología, es mas también como el chicle por querer resolver las cosas, y le doy una perla más ahora hemos descubierto que por ejemplo uno resuelve por telemedicina el tema de consulta, pero no resolvemos por ejemplo el tema de que tómese este laboratorio y cual le toca bajar a Cali, como resolver eso por ejemplo, entonces por un mes vamos a dar a funcionar, que un dron recoja la muestra se la toman allá, el dron la recoge la procesamos y por internet le entregamos el resultado. Donde se hace eso en el mundo, pues no se hace en ninguna parte del mundo, pero volvemos al tema que es lo que nos motiva, no es la tecnología si no que siempre cuando uno está pensando como resuelvo el tema, pero si el tema es tecnológico, pues lo resolvemos, pero no siempre, por ejemplo en salud del campo ahora que lo vamos a hacer el relanzamiento, cómo resolvemos el tema de acceso en ciertos territorios, con un caballo eso es más prehistórico, no tiene nada de tecnológico  es mas en aras de resolver, en Pance como sacamos un paciente por la carreta, necesitamos una cuatrimoto que pueda cargar, entonces no es tecnológico, es más un chicle por querer resolver las cosas

P10: 3. Transcripcion-Alexandra_Matallana_ok.docx - 10:20 [Porque como está escrito en la..]  (186:186)   (Super)
Códigos:	[Capacidades del sistema : accesso a la salud] 
No memos

Porque como está escrito en la ley digamos las capacidades para prevenirlas, se delegó y no digamos toda la parte de salud pública toda la parte de promoción y prevención es una parte que se dejó a un lado y se dedicó a la curación a los terceros y cuartos niveles pero la  resolutividad en lo primero niveles, yo creo que ahí esta el quick de la cosa y creo que nuevamente si hacemos unas rutas de atención entrando por la baja resolutividad y allí se resuelven los problemas seguramente ese 53% que hay de muertes evitables seguramente podremos evitar algunas de ese 53%, muchas yo creería. Fortaleciendo mucho la capacidad del recurso humano del primer nivel para que resuelvan y no para que remitan,  porque en este momento lo que hace la baja complejidad es un sistema de remisión, a ti no te solucionan absolutamente nada en la baja complejidad no te lo solucionan, porque eso también es algo para el sistema de formación del recurso humano en salud en Colombia, que tenemos que formar personal de salud que pueda resolver en los niveles bajos antes de que los pacientes se nos compliquen y se nos mueran. Entonces hay que hacerlos y las Universidades y los currículos y los pensum deben dirigirse hacia allá, ósea tenemos que formar recurso humano capacitado para que pueda funcionar el MIAS.

P10: 3. Transcripcion-Alexandra_Matallana_ok.docx - 10:21 [Es claro si tú no tienes un ta..]  (190:190)   (Super)
Códigos:	[Capacidades del sistema : accesso a la salud] 
No memos

Es claro si tú no tienes un talento humano formado para algo pues no le puedes pedir que lo haga, porque no tiene la capacidad la masa crítica para hacerlo, si tu solamente formas médicos que reciben al paciente no lo miran a los ojos no pregunta que tiene y solamente da órdenes para exámenes y órdenes para especialistas, ¿que estas resolviendo allí? Nada.

P10: 3. Transcripcion-Alexandra_Matallana_ok.docx - 10:22 [Pero nuevamente escrito pero, ..]  (197:197)   (Super)
Códigos:	[Capacidades del sistema : accesso a la salud] 
No memos

Pero nuevamente escrito pero, bajas tu pero allá no se está haciendo nada, te digo acabo de venir de la Guajira… nada, está en el papel.


P10: 3. Transcripcion-Alexandra_Matallana_ok.docx - 10:25 [Yo pienso que las barreras adm..]  (228:228)   (Super)
Códigos:	[Capacidades del sistema : accesso a la salud] 
No memos

Yo pienso que las barreras administrativas, que no permiten  que las personas ingresen al sistema de salud, yo pienso que esas barreras administrativas… pues que no se si serán intencionales o no intencionales para que la gente no llegue al servicio pero pues lo más importante son las barreras administrativas, porque allí es donde las personas no pueden entrar al sistema. Y la barrera del desconocimiento por parte de la población de cómo es la ruta,  como es que funciona, porque es que llega a la ventanilla a pedir la cita pero entonces tocaba traer otro papel y cuando va a traer el otro papel pero entonces tiene que también traer la remisión de yo no sé qué y vuelve, esas barreras administrativas también son unas barreras de información de la gente, ósea las EPS deberían también tener informada a su gente y decir mire si usted quiere entrar a esto,  tiene que entrar así, pero la gente no sabe y solo cuando tiene una enfermedad digamos o algo crónico o algo fuerte es que se da realmente cuenta  cómo funciona el sistema.

P11: 4. Transcripcion Armando Gonzales_ok.docx - 11:5 [Excelente en la teoría, eso es..]  (17:17)   (Super)
Códigos:	[Capacidades del sistema : accesso a la salud] 
No memos

 Excelente en la teoría, eso es lo que debería hacer siempre. Si uno tiene un paciente que tiene diabetes, pero lo importante es que se atienda todas las dificultades del manejo del diabético en una sola institución y no es conveniente que hayan centros que tienen servicios limitados y que el resto de servicios tiene que ser enviado a otras partes, lo ideal sería que usted llegara a cualquier institución con la patología o enfermedad que llegue, y allí recibiera todos los servicios de manera integrada hasta la radioterapia cuando se requiere para recibir todo su tratamiento y no por fracciones, eso no es lo ideal. Colocar en Colombia un modelo de gestión integral es una cosa complicada porque ¿qué se hace con toda esa cantidad de IPS que no ofrecen si no servicios limitados?, que se va a hacer con ellas, tendría que hacer una reforma muy radical para decir que solamente la atención medica se puede prestar si se garantiza la integridad de la atención, en un país como este y en cualquier otro. 

P11: 4. Transcripcion Armando Gonzales_ok.docx - 11:12 [Yo pienso que sí, lo que pasa ..]  (77:77)   (Super)
Códigos:	[Capacidades del sistema : accesso a la salud] 
No memos

Yo pienso que sí, lo que pasa es que los recursos no son suficientes pero se lo están garantizando, entonces se atiende a quien tiene las patologías más graves; porque por fuerza hay que atenderlos pero hay patologías que vos llegas a pedir una consulta y te la dan para cuatro meses ahí no te están garantizando nada, si tú te sientes enfermo y te dan una cita a cuatro meses, te sientes mal, entonces que hace la gente, cuando se siente enfermo o acude a la tutela o va a urgencias; entonces el 80% de las personas que van a urgencias no debería ser atendidas en urgencias, podrían ser atendidos por consulta. Y ustedes ya lo comprobaron y es un dato que se ha manejado siempre, entonces la gente que llega a urgencias no requieren la atención de urgencias pero la gente no tiene otra alternativa, porque si te sientes enfermo y te van a atender en 3 meses pues tu buscas otra alternativa.

P12: 5.Transcripcion Carlos Fajardo_ok.docx - 12:4 [La capacidad actual del sistem..]  (14:14)   (Super)
Códigos:	[Capacidades del sistema : accesso a la salud] [Desigualdades en salud] 
No memos

La capacidad actual del sistema que está instalada sigue siendo muy dispar tenemos un gran problema en el departamento del valle donde hacemos presencia, con un hospital universitario donde sobre todo lo que es correspondiente a la baja complejidad, no hay instituciones que hoy resuelvan toda esa problemática de salud. Lo mismo pasa en Nariño, los niveles de alta complejidad los hospitales municipales no existen y eso hace que toda esa carga de enfermedad de la baja complejidad este yendo a los grandes hospitales, entonces se congestionan los hospitales universitarios, se congestionan el hospital San Pedro, se congestiona el Hospital Universitario del Valle, se congestiona fundación Valle del Lili, porque los usuarios pretenden siempre resolver a través de las urgencia,  resolver problemas que debieron haberse resuelto en el primer nivel, que es donde realmente no hay resolutividad en esa parte. 

P12: 5.Transcripcion Carlos Fajardo_ok.docx - 12:6 [Exactamente, a pesar de que ha..]  (22:22)   (Super)
Códigos:	[Capacidades del sistema : accesso a la salud] 
No memos

Exactamente, a pesar de que hay la resolución la 1479 donde dice que los departamentos deben hacerse responsables del no POS, pero eso no existe, hasta ahora ha habida algunas intentos de controlar, sobre todo si no hay una concertación en lo que es la rama judicial con el sistema del gobierno que regula o reglamenta la parte de salud, va  a ser complicado porque hoy Colombia tiene un post infinito, ya que a través de la tutela se accede a cualquier tipo de tratamiento o a cualquier tipo de medicamento o a cualquier tipo de intervención. 

P12: 5.Transcripcion Carlos Fajardo_ok.docx - 12:25 [Creo que ahí hay una concepció..]  (94:94)   (Super)
Códigos:	[Capacidades del sistema : accesso a la salud] 
No memos

Creo que ahí hay una concepción muy amplia de lo que es el derecho a la salud, para mí eso es lo que afecta hoy al sistema como tal, porque se plantea que el derecho a la salud es todo lo que se quiera, no hay unos limitantes y como es un derecho fundamental entonces si la persona está en postrada y no tiene para pañales, hay que darle para pañales, si el niño no tiene una buena alimentación y necesita multivitamínicos, entonces hay que darle ensure. Entonces el derecho a la salud debe tener como unos limitantes, si una persona se hizo una cirugía plástica, tuvo para hacerse colocar senos, para hacerse colocar cola, y cuando se presentan las complicaciones entonces las EPS son las que tienen que responder por las complicaciones, o cuando las personas buscan a través de estos procedimientos en el exterior, cuando en el país son experimentales. Entonces el derecho a la salud creo que lo hemos ampliado tanto que hoy todo es permitido.

P12: 5.Transcripcion Carlos Fajardo_ok.docx - 12:26 [i no hay un marco de referenci..]  (102:102)   (Super)
Códigos:	[Capacidades del sistema : accesso a la salud] 
No memos

i no hay un marco de referencia, un marco hasta donde va ese derecho no, si va como hoy esta que es libre todo, es muy difícil que el gobierno pueda garantizar ese derecho. Porque es amplio y si no me cumplen entonces están violando mi derecho a la salud. 

P12: 5.Transcripcion Carlos Fajardo_ok.docx - 12:24 [Yo creo que eso sigue igual, h..]  (122:122)   (Super)
Códigos:	[Capacidades del sistema : accesso a la salud] 
No memos

Yo creo que eso sigue igual, hoy el país se está centralizando a hacer vías en los centros de mayor acopio, en los centros donde hay importaciones, uno no ve que haya una vía en una veredita de Tumaco, o de buenaventura, están haciendo la doble calzada para buenaventura para que haya el transporte de carga, hay poblaciones que están a 14 horas y no se ve un mejoramiento de la vía, entonces el acceso como tal no creo que vaya a mejorar, puede ser que si entra un concepto que se viene hoy promulgando mucho hoy que es la tele medicina y a través de los centro especializados eso permite mejorar un poco el acceso mas no que haya un cambio sustancial en eso. 

P13: 6 y 7.Transcripcion Diego Gomes y Gonzalo Gonzales_ok.docx - 13:19 [Nosotros no tenemos unas barre..]  (117:117)   (Super)
Códigos:	[Capacidades del sistema : accesso a la salud] 
No memos

Nosotros no tenemos unas barreras consistentes de salud realmente, ese es un mito colombiano y un mito profundamente injusto con el sistema, que barreras de salud vamos a tener cuando tenemos menos colas de atención para cirugías para citas con especialistas, para citas con subespecialistas, cuando no tenemos copagos significativos, cuando no tenemos cuotas moderadoras significativas, cuando cualquier persona accede a tratamientos de 200 y 300 millones de pesos que fácilmente multiplican por diez sus ingresos anuales, cuales son las barreras reales de salud nuestra. Esos son de los peores mitos que tenemos, nosotros en Colombia somos de los países con menos barreras de salud en el mundo.

P15: 9. Transcripcion Julian Duran_ok.docx - 15:20 [Pues a ciencia cierta no sabrí..]  (114:114)   (Super)
Códigos:	[Capacidades del sistema : accesso a la salud] 
No memos

Pues a ciencia cierta no sabría decirse cuál es la razón pero de sentido común uno diría que esas son enfermedades que están asociadas a la vida sedentaria de las personas y que son enfermedades que tienden a presentarse con la edad entonces lo que puede reflejar eso es el cambio en la pirámide poblacional, donde las personas mayores tienden a aumentar el porcentaje, entonces al aumentar estas personas tienden a aumentar las enfermedades que les dan a ellos a ese segmento de la población, como son las cardiovasculares o las del corazón las relacionadas con las arritmias del corazón


P15: 9. Transcripcion Julian Duran_ok.docx - 15:23 [Las primera tiene que ver con ..]  (126:126)   (Super)
Códigos:	[Capacidades del sistema : accesso a la salud] 
No memos

Las primera tiene que ver con el régimen subsidiado, es un régimen al que no todo el mundo accede si no que tiene que cumplir ciertas condiciones económicas para poder acceder a los beneficios que dan este régimen y quedar en un nivel de sisben que si le permita aprovechar estos beneficios. En el régimen contributivo tiene que ver con la informalidad que debería aumentarse el empleo formal, el empleo que paga prestaciones sociales y que paga en el régimen contributivo, entonces si la economía formal no aumenta más que la población entonces va a haber una brecha que se va a generar y es que el régimen contributivo no va a tener entonces como seguir creciendo, como absorber nuevos usuarios. 

P16: 10.Transcripcion Angela Tascon.docx - 16:13 [Porque la prevención no es un ..]  (54:54)   (Super)
Códigos:	[Capacidades del sistema : accesso a la salud] 
No memos

Porque la prevención no es un estandarte por más en lo que he leído, porque nosotros no manejamos aquí programa de prevención y redención pero en lo que uno lee de los informes hay un dinero importante para promoción y prevención, pero ese dinero es uno de los que más se embolata, y eso hace que precisamente la gente cuando se descubre el cáncer o la enfermedad que se descubra ya se convierte en algo de alto costo, entonces a mí me parece que el gobierno le falta mucho mas dijéramos analizas respecto sobre esa promoción y prevención van a  rebajar los cosos y va a mejorar la eficiencia del dinero del sector, entonces la promoción y la prevención yo diría en estos momentos es fundamental ahí, porque es mucha consecuencia de estas enfermedades que están liderando los primeros puestos en muerte, es la pate alimenticia, acá es arroz, papas y ACPM. Comemos muy mal entonces a la gente hay que educarla, a la gente las verduras no le gusta, apenas estamos entrando a esta era verde, vea europea con la dieta mediterránea, entonces ha sido mucho la educación y en la pirámide poblacional el mayor porcentaje son los estratos más bajos, y tienen menos acceso a la educación, acceso a las salud es digamos universal, pero que me gano yo con que pueda y tenga la facilidad de un régimen subsidiado donde me dan una cita médica si no puedo conseguir los remedios si como mal si no pongo en práctica porque no tengo la educación. La gente tiene muchos fetiches y creencias culturales antiguas que precisamente no favorecen a la salud, entonces acá comemos muy mal y siempre oímos que lo que comemos de jóvenes es lo que se vive de viejo y ahí es donde dijéramos como macro por eso le hablo de la promoción y prevención y en eso hay un campo muy importante para esta parte nutricional. Que genera un infarto, la mala alimentación eso está muy entrelazado ahí esta parte. 


P16: 10.Transcripcion Angela Tascon.docx - 16:17 [Si no hay derecho que con un a..]  (70:70)   (Super)
Códigos:	[Capacidades del sistema : accesso a la salud] 
No memos

Si no hay derecho que con un aseguramiento de un 98% el 25% no llegue y ahí se funden varias cosas, una importante es la cultura la gente trata como tiene tan fácil acceso a comprarse el aspirina o el antibiótico allí, esto le demanda tiempo porque cuando ya van usted lo dijo muy claro la urgencias están colapsadas, pero porque no hay promoción ni prevención, otra parte cultural que tiene que ver mucho con los valores, el colombiano es el campeón para ir al médico a que le den dos días de incapacidad por un guayabo, si le pasa a uno acá cerca esa idiosincrasia es complicada porque como tenemos fácil acceso a cualquier medicamento, que eso es otra parte grave, eso hace que la gente los primeros cuidados se los de folclóricamente por tradición, y ya cuando esta grave va a colapsar a urgencias, entonces eso hace que el sistema no pueda porque el sistema permite que la gente haga todos estos goles, pero la cultura la educación es definitivas y le impacta mucho al sector, gente que no se hace los tratamientos porque no hay promoción porque no hay esa organización, a nivel de promoción y prevención para que la gente tenga conciencia de que debe hacerse el tratamiento completo. Mire por lo menos yo me quedo aterrada con las noticias de la guajira, que venimos hace rato con este tema de los niños desnutridos que se mueren, a muchos les ha llegado la ayuda adecuada y científica y no la han aceptado, tenemos casos de papas que sacan al niño del hospital y se lo llevan porque la cultura no los ha permeado todavía y siguen con su parte cultural.

P18: 11. Transcripcion Fabian Mendez.docx - 18:7 [no creo que el modelo aguante ..]  (11:11)   (Super)
Códigos:	[Capacidades del sistema : accesso a la salud] 
No memos

 no creo que el modelo aguante más los asuntos de corrupción que tenemos, la iliquidez del sistema lo que estamos viendo cada vez más, los problemas de falta de acceso real porque lo que tenemos es una población cubierta entre comillas pero sin real acceso

P18: 11. Transcripcion Fabian Mendez.docx - 18:8 [Como ya lo han dicho algunos e..]  (15:15)   (Super)
Códigos:	[Capacidades del sistema : accesso a la salud] 
No memos

Como ya lo han dicho algunos estudios de proyección demográfica en el país, estamos en un proceso de transición avanzado con la diferencia de que en otros países este proceso tomo mucho más tiempo que el que nosotros estamos teniendo que afrontar, lo que a Francia le tomo 50 años nosotros lo hemos tenido que vivir en 20, y eso implica retos muy grandes para el sistema con una población cada vez más envejecida pero que tiene debido a la precarización de las capacidades laborales no tienen seguridad social, o es digamos esta situación precarias donde no tienen tampoco acceso a pensión y se va sumando toda una carga para un sistema donde las enfermedades crónicas van a ser cada vez más atendidas, y lo que encontramos es que consistente con otros estudios que tenemos una población que depende de sus familiares directa o indirectamente para poder acceder al sistema de salud que la capacidad que tiene para cubrir los gastos de bolsillo en salud es reducida, entonces esta es una carga que cada vez va a ser más y más grande, y va a la postre con lo que uno puede imaginarse en un término de mediano plazo, en términos de enfermedad renal crónica, hipertensión y diabetes, seguro van a poner aún más en situación crítica en términos financieros a un sistema como el actual. Entonces necesitamos reformular hacer una visión más en promoción a la salud porque lo que se nos viene hacia adelante es más grave un modelo totalmente curativo.

P19: 12. Transcripcion Fabio Osorio.docx - 19:12 [Es cuando un piensa que es un ..]  (62:62)   (Super)
Códigos:	[Capacidades del sistema : accesso a la salud] 
No memos

Es cuando un piensa que es un negocio y como que maquillan algo y prefieren que la gente se enferme a que prevengan la enfermedad, yo siempre lo he dicho acá en Colombia el sistema privilegia la atención del enfermo y no la atención de la persona que se puede enfermar, acá prefieren meterle mas plata a un insumo o medicamento que se pueda vender y se saque la comisión a un médico en casa desde el médico familiar, dese la misma educación donde se prevenga donde se promocione la prevenciones salud para que la gente no se enferme, si acá en Colombia se sigue privilegiando la atención mas no la prevención no pasa nada, la prevención no les deja plata para robarse porque esa es de educación de hábitos de salud, de estilos de vida saludable, de servicios públicos, de servicios de atención psicosocial acá en Colombia donde se deje de promocionar tanto los alimentos que hacen daño porque las enfermedades cardiovasculares no aparecen de un día para otro ellas aparecen es por el mal manejo de hábitos de vida saludable en los seres humanos en Colombia, acá se privilegia por eso lo de las bebidas azucaradas, la diabetes aparece porque el colombiano prefiere almorzar desayunar y comer con una gaseosa a con un jugo natural, a con una línea de educación básica de dieta y nutrición a los colombianos, para quitarles el negocio pero ellos saben que el negocio de la azúcar más que todo en el valle con tanto ingenio azucarero, es más rentable que la gente tome y consuma gaseosa a que la gente de verdad tenga un estilo de vida saludable y tome otra bebida que no le haga tanto daño como la azucarada, acá la gente prefiere comer llenarse de grasa, todo eso es una cadena, que va llevando a que el paciente sufra un infarto porque se le tapono una arteria. Acá preferimos que nos invada la comida en grasa y no la saludable como debería de ser. 

P19: 12. Transcripcion Fabio Osorio.docx - 19:13 [Lo que yo conozco es uno la gr..]  (66:66)   (Super)
Códigos:	[Capacidades del sistema : accesso a la salud] 
No memos

Lo que yo conozco es uno la gran tramitomania que hay que hacer, al paciente le mandan miles de órdenes para que tenga acceso directo al centro de salud si una persona no está al día en su cuota de la EPS simplemente le dicen no está afiliado y fuera del sistema, una barrera para que el derecho a la salud sea fundamental es que hay mucha demanda y poca oferta, el hospital le llegan 30 pacientes pero solo hay un médico y la gente quiere atención rápida, pero él no se puede volver la máquina de escribir y que el Windows le arroje lo que tiene, entonces yo creo que es la poca oportunidad de médicos para atención de gran cantidad de pacientes. Colombia no está sacando especialistas un médico general no te puede atender un diabetes si no es un médico internista, no hay gran cantidad de cirujanos no hay gran cantidad de anestesiólogos, entonces uno piensa es que quieren cerrar el negocio para que no haya tanta oferta porque la demanda sigue creciendo, entones es increíble que cada día solamente haya un médico especialista, en la ciudad hay un solo hematólogo pediatra y la cantidad de niños con cáncer es impresionante, porque no se profesionalizan más en especialidades para que cuando haya esa gran cantidad de pacientes haya esa oportunidad entonces yo creo que una de las barreras que hay es que no se forman profesionales que no son de un médico general que solo lo detecta y remite al especialista pro cuando remite le dan la cita para dentro de 6 meses, porque como es uno solo tiene que atender a gran cantidad de pacientes. 

P22: 13. Transcripcion Maria Ines Pantoja.docx - 22:20 [Mire la primera barrera es las..]  (103:103)   (Super)
Códigos:	[Capacidades del sistema : accesso a la salud] [cobertura] 
No memos

Mire la primera barrera es las citas médicas, si a usted no le abren esas puerta  de citas médicas, no tiene nada, porque no tiene exámenes ni tratamiento. De la principal barrera es el acceso, nosotros tenemos una encuesta que hicimos con el ministerio sobre barreras y esa encuesta empieza por el acceso, entonces las cita médicas, los exámenes, luego la remiso al especialista, las citas con especialista, los exámenes, el tratamiento. Entonces si a mí no me abren esta puerta para ingresar al sistema es muy difícil porque después no entro, y después aquí otra hay otra puerta que es entrar donde el especialista, y esto se demora de 6 a 8 meses y después donde el especialista la barrera para los exámenes, los que están en el POS y los que no están en el POS. Luego la barrera para procedimientos porque como no hay red los procedimientos los tienen que pagar por evento , y eso sale costosísimo, porque ya las IPS no quieren prestar servicios si no son pagos por adelantado, entonces ya son unas barreras que el paciente tiene, eso es toda una cadena y luego ya en la continuidad del tratamiento también hay barreras de acceso, porque aquí el médico me ordeno el no POS por 6 meses, para probar el no POS la ley dice que son 2 días hábiles, pero las EPS abusivamente son 10 o 15 días para entregarle al paciente la orden del medicamento. Entonces a mí el no POS me lo aprobaron por 6 meses, entonces en este momento las EPS que están haciendo, volviéndome a que yo tengo que pedir otra autorización para yo poderlos reclamar, ósea cada vez tengo que averiguarlo, eso es ilegal eso no aparece en ninguna norma, entonces mientras me dan la autorización yo puedo perder hasta 15 días de tratamiento, hemos hecho el ejercicio de perdida de tratamiento y los tratamientos van de dos meses a 6 meses, ósea que las EPS se están ahorrando una UPC de 6 meses, o de dos meses dependiendo. Ósea que la barrera es una cadena que tiene la persona.


P22: 13. Transcripcion Maria Ines Pantoja.docx - 22:21 [Lo que yo veo es que esto cada..]  (107:107)   (Super)
Códigos:	[Capacidades del sistema : accesso a la salud] [cobertura] 
No memos

Lo que yo veo es que esto cada día se está aumentando más, han estado peor las barreras de acceso al sistema, hemos hablado del secretario de salud, de Capitalsalud, cuatro meses sin atender a los pacientes, nos prometió que la otra semana iba a empezar. Es un desgaste yendo a las EPS con la Supersalud, y como que nadie ha encontrado la solución, yo veo que esto esta cada día empeorándose, es una bomba de tiempo que hay muy grande

P22: 13. Transcripcion Maria Ines Pantoja.docx - 22:22 [Yo pienso que por el maltrato ..]  (115:115)   (Super)
Códigos:	[Capacidades del sistema : accesso a la salud] [cobertura] 
No memos

 Yo pienso que por el maltrato que se le da a la gente, prefieren quedarse en su casa tomando agüita de hiervas que ir al médico,  por el nivel cultural también las personas del régimen subsidiado son personas que no tienen una cultura y son personas muy vulnerables entonces yo pienso que prefieren quedarse en su casa enfermos, porque es insólito que el régimen subsidiado sean menos, porque es gente que tiene menos condiciones saludables, en el régimen contributivo la gente vive un poco mejor, tienen un poquito más de calidad de vida, dependiendo del nivel en que este, influye el maltrato que se le da a la gente, también el nivel de cultura, y esa gente a veces más bien acude a yerbateros a otra case de atención pero no acuden a los médicos. Por ejemplo entre los indígenas, ellos prefieren acudir a sus sabios a sus chamanes, que a los médicos porque en el chamán encuentran uno igual y al ser igual uno confía más en la persona que es igual a uno, que en el que esta allá con prepotencia, se siente menos confianza. Yo estuve hace poquito en un resguardo indígena en Tibu, precisamente recogiendo esto de las barreras, esa pobre gente vive en una mísera cafesalud tiene allá atiende a casi la mitad de la tribu, y no tiene oficina allá en Tibu, y los indígenas tienen que venir a doce horas a Tibu y ahí no encuentran de cafesalud una oficina que los atiendan para darle la cita, y tienen que ir hasta Cúcuta. Entonces como se va a ir esa persona con esa pobreza que tienen, él va donde el chamán y le da cualquier cosa, yo digo que esa es una de las causas. Hay una tribu que tiene mucha tuberculosis y otra cirrosis de las dos comunidades de los indígenas motilones Bari, hay una comunidad que tiene prevalencia de hepatitis de cirrosis, y otro que tiene de tuberculosis, entones ellos me decían a mi como me voy a ir hasta Cúcuta a pedir citas entonces la enfermedad esta avanzando entre ellos. Cuando está muy grave lo llevan al hospital y si no encuentran a alguien que los ayude a entrar los dejan 3 o 4 días en urgencias, eso le tienen ellos pavor, llegan y los mandan a un sitio donde ellos tienen para atenderlo que eso si tienen, y duran allí meses sin poder llegar a sus familias porque no le dan la cita con el especialista, entonces la gente dice a qué voy. Voy a gastar u mes que se me daña los cultivos, entonces prefiero quedarme acá y no buscar atención. Y los indígenas son del subsidiado que poco consultan este servicio


P22: 13. Transcripcion Maria Ines Pantoja.docx - 22:23 [Ojala tuvieran red, pero no ha..]  (119:119)   (Super)
Códigos:	[Capacidades del sistema : accesso a la salud] [cobertura] 
No memos

Ojala tuvieran red, pero no hay red es que, que red van a tener si no tienen por lo menos una oficina en Tibu para atender a los indígenas, hay para el contributivo mas no para el subsidiado. 

P24: 15. Transcripcion Martha Balbuena.docx - 24:9 [Muchas veces nosotros pensamos..]  (70:70)   (Super)
Códigos:	[Capacidades del sistema : accesso a la salud] 
No memos

Muchas veces nosotros pensamos que todo lo de salud lo tiene que resolver salud y resulta que acá entra algo muy importante que es la intersectorialidad entonces si la parte de la violencia tiene mucho que ver con la parte social, si la gente no tiene trabajo o vivienda, todo lo que son condiciones de bienestar, a veces me pregunto y el gobierno dice que hay vivienda, pero esa vivienda cumple con lo mínimo… son bonita pero creo que una vivienda es pequeña las que da el gobierno, y resulta que allí entran 6 personas a vivir, la gente están dentro de un condominio pero realmente si el gobierno hubiera pensado que en vez de hacer eso tan pequeñito hubiera hecho algo más grande, pero ahí yo digo esta la parte social de salud, de recreación si empezáramos a mejorar las condiciones que la persona debe tener que es lo mínimo podría hacer algo, el gobierno dice vivienda pero donde está el trabajo para poder la gente llevar algo, donde está la recreación, pero eso no es. 

P24: 15. Transcripcion Martha Balbuena.docx - 24:10 [Si y que no podemos trabajar c..]  (74:74)   (Super)
Códigos:	[Capacidades del sistema : accesso a la salud] 
No memos

Si y que no podemos trabajar cada uno por su lado, y se tiene que dar mucho la parte de intersectorialidad y le voy a contar algo, yo digo llega la factura, yo he dicho bueno el gobierno o que no puede dar trabajo y que nos llegara la vigilancia allí mismo, cada barrio tiene que buscar la forma alarmas, vigilancia por cuadra, pero también en muchos barrios y yo lo viví en esa comuna cuando están los muchachos fumando y no haciendo nada, porque no tienen recreación, ese grupo social para disminuir esos problemas y ellos mismos son las que hacen la violencia ósea sin trabajo sin nada, tendríamos que mirar la parte de determinantes social

P31: 20.Transcripcion Hellen Mendoza.docx - 31:8 [Porque uno mira las edades de ..]  (27:27)   (Super)
Códigos:	[Capacidades del sistema : accesso a la salud] 
No memos

 Porque uno mira las edades de la gente, la población colombiana donde está, el Pareto de la población colombiana esta, nosotros tenemos un peso importante entre los 45 y 49 años, tenemos una población envejecida pero ha ido creciendo pero también nos están creciendo los niños, adonde vamos nosotros a impactar con el MIAS, a la población más joven en los niños y no los adolescentes, el viejito que tiene la hipertensión o diabetes el adulto que tiene el cáncer, ahí no podíamos impactar con el MIAS como si podremos impactar en un niño que está en crecimiento y desarrollo, y el que esta arrancando hoy, en 20 años va a ser un adulto joven sin hipertensión, sin diabetes, sin enfermedades asociadas a riesgo cardiovascular, porque desde pequeñito los hemos cogido y les hemos enseñándolos hábitos de prevención. Nosotros hoy no estamos acostumbrados a cuidarnos, tomamos trago, fumamos, trasnochamos, comida chatarra, porque asumimos que tenemos el derecho a enfermarnos y la clínica tiene la obligación de atendernos porque la EPS tener que pagar, nosotros hoy no estamos diciendo que si nosotros garantizamos nuestro autocuidado, no va haber rentabilidad financiera, pero va a haber rentabilidad social

______________________________________________________________________

Código: cobertura {20-0}

P15: 9. Transcripcion Julian Duran_ok.docx - 15:5 [Yo pensaría que es difícil est..]  (23:23)   (Super)
Códigos:	[cobertura] 
No memos

 Yo pensaría que es difícil establecer eso, porque como muestran las cifras,  la población esta aumentando y la cobertura no está aumentando al mismo ritmo, entonces se demoraría muchos años en alcanzar la cobertura universal.

P15: 9. Transcripcion Julian Duran_ok.docx - 15:6 [Pues lo más posible es que no ..]  (27:27)   (Super)
Códigos:	[cobertura] 
No memos

Pues lo más posible es que no se alcance


P15: 9. Transcripcion Julian Duran_ok.docx - 15:7 [Pues el principal problema es ..]  (31:31)   (Super)
Códigos:	[cobertura] 
No memos

Pues el principal problema es que no se logra consolidar un sistema de salud que sea eficiente y sostenible, entonces el sistema actual no logra dar la cobertura y no se ven que se estén implementando las estrategias o las políticas para lograrlo. 

P15: 9. Transcripcion Julian Duran_ok.docx - 15:8 [Una dinámica como la informali..]  (35:35)   (Super)
Códigos:	[cobertura] 
No memos

 Una dinámica como la informalidad por ejemplo hace que la gente tienda más hacia el régimen subsidiado y en general pues las personas están viendo que en el régimen subsidiado tienen ciertos beneficios que no logran con el contributivo y es muy posible que la gente se siga desviando hacia el régimen subsidiado. 

P22: 13. Transcripcion Maria Ines Pantoja.docx - 22:4 [Hay una cuestión que a mí siem..]  (7:7)   (Super)
Códigos:	[cobertura] 
No memos

Hay una cuestión que a mí siempre me preocupa y es el régimen subsidiado, me preocupa porque en el futuro vamos a tener personas muy enfermas, ancianas y sin seguridad social, porque esas personas no van a tener una pensión y no van a tener absolutamente nada. En el proyecto que tenía el ministerio del nuevo sistema de afiliación, había una parte donde las personas se podían afiliar con menos del mínimo, a mí me parecía muy bien, pero esa parte no la dejaron pasar porque esa parte le tocaba era al ministerio de hacienda, pero si me preocupa mucho el problema del régimen subsidiado. El problema de desempleo también afecta a la salud porque son personas que sin empleo afectan porque no aportan al sistema, entonces al no afectar están afectado la parte económica del sistema de salud, el no tener trabajo también yo creo que enferma a la gente, por ejemplo Bogotá ha sido una ciudad donde han inventado el Bogotá sin hambre, muchas cosas para darle de comer a la gente, pero la gente no necesita solo comida, la gente necesita otras cosas especialmente educación, necesita fuentes de trabajo, porque la comida se acaba, viene un gobierno diferente se les acaba eso y entonces tenemos gente que ya no tiene ni para comer y eso generan un problema social bastante grave

P22: 13. Transcripcion Maria Ines Pantoja.docx - 22:6 [Yo creo que la afiliación pued..]  (15:15)   (Super)
Códigos:	[cobertura] 
No memos

Yo creo que la afiliación puede ser del 95% al 100%, pero ya que eso sea efectivo en una prestación de servicio es otra cosa, eso es como tan insólito ver que las personas tenga que hacer cola a las 3 de la mañana, y que muchísimas de esas personas no alcanzan a coger ni siquiera una ficha para que le presten el servicio, tiene que ir al otro día a hacer lo mismo. Yo creo que eso es totalmente aberrante porque a las EPS le están pagando adelantado la UPC, se las dan adelantadas y porque no ofrecer los servicios somos deberían de prestarlo. Ahí está violando todos los derechos y el respeto a la persona humana, martirizar a una persona para que todos los días vaya a pedir una cita, yo no creo que uno alentado haga esa cola tan impresionante entonces es un sistema que no hay quien lo controle se parece que se salió de las manos de todo el mundo, los medios han mostrado las colas lo saben el ministro,  el presidente lo saben las  EPS, la corte ha llamado a las EPS al orden pero no hay quien las controle.

P22: 13. Transcripcion Maria Ines Pantoja.docx - 22:7 [yo creo que como estamos va a ..]  (19:19)   (Super)
Códigos:	[cobertura] 
No memos

 yo creo que como estamos va a aumentar, porque el país no ha pensado en lo más importante es dar fuentes de empleo, porque la gente pueda contribuir al sistema de salud y también puedan tener un mejor bienestar. Yo creo que esto va a seguir subiendo, también hay mucha gente que le falta conciencia que tiene con qué pagar y no pagan y el estado tiene que ponerse en esa tarea de que los que puedan pagar lo haga, porque hay mucha gente el régimen subsidiado que no debería estar en el régimen subsidiado, deberían estar en el contributivo porque tienen capacidad de pago. Y las personas que no tiene capacidad de pago, si vemos son mayor en este momento y yo creo que esto si no cambian esta situación esto va a seguir elevándose el número de personas con régimen subsidiado, y menos con régimen contributivo, yo creo que eso está en lógica con el desempleo, si hay desempleo la gente no podrá aportar al régimen contributivo, entones yo digo que no podemos separar lo de la afiliación con el trabajo porque si yo tengo trabajo tengo que contribuir o la empresa contribuye, pero si no tengo trabajo o un empleo fijo, los vendedores de la calle, los mismos taxista que muchas veces no tienen el régimen contributivo, y devengando un sueldo deberían contribuir. Han hecho muchas cosas y el ministerio ha querido como crear unas formas de afiliación pero no sé, por eso yo le decía se ha fallado porque las bases no son sólidas, tenemos unas bases del sistema de salud muy quebradizas, sin consistencia. 

P22: 13. Transcripcion Maria Ines Pantoja.docx - 22:14 [Pues mire a nosotros con la le..]  (76:76)   (Super)
Códigos:	[cobertura] 
No memos

Pues mire a nosotros con la ley estatutaria nos alegramos mucho, habían muchas cosas con la cuales no estábamos de acuerdo y estuvimos precisamente el 20 de julio del 2013 pasamos el día en el congreso de la republica porque ese día se aprobaba la ley estatuaria, salió con algunas deficiencias como el no apoyo a los servicios concomitentes, incondicionamiento en la participación ciudadana, entre otras cosas. Cuando la corte abrió toda la parte que iba a ser la audiencia pública estuvimos en la corte hablando de como ciertos artículos de la ley estatutaria nos quitaban derechos ya adquiridos, y eso no se puede un derecho adquirido antes hay que progresar en el derecho, y nosotros trabajamos mucho la ley estatutaria con la sentencia de la corte, hicimos un paralelo y lo que concluimos era que estaba violando unos derechos de las personas y eso se le expuso a la corte, por ejemplo la participación ciudadana está en la ley completamente clara, y la participación la condicionaban con las decisiones ya adoptadas, entonces nosotros hicimos un trabajo de 3 días, se mandó ese documento a la corte y después se sostuvo en la plenaria que hizo la corte para esto, afortunadamente para la corte tuvo en cuenta esto porque esto era de la constitución, y logramos de que la ley quedara más ajustada a lo que nosotros queríamos, de ahí para acá lo fundamental fue que se declaró la ley a la salud como un derecho fundamental, dice que no puede renunciar ni uno mismo no colectivamente a ese derecho porque es un derecho fundamental. Pero del escrito a los hechos es totalmente diferente parece que esto fue retroceso y la ley estatutaria no se está cumpliendo, porque si es un derecho fundamental se tendría que respetar, pero no lo respetan porque tienen una posición dominante la EPS y contra ellos nada, ni la corte ni nada. Si yo no le hago caso a nadie, y los entes de control son muy débiles, no tenemos entes capaces de regular y organizar eso. En este momento en la superintendencia de salud esta de delegado del derecho a los pacientes el doctor Varela que estuvo en la superintendencia financiera y resulta que él quiere poner el modelo a salud, y nosotros le decimos es un derecho totalmente diferente porque, porque a mí me están cobrando más por este servicio, a mí eso no me afecta en mi integridad personal, pero a la salud si me afecta. Con unos espacios muy largos de solución y los problemas de salud toca inmediato porque por eso estamos como estamos, a la gente no se le atiende a tiempo no se tiene promoción y prevención, y entonces cuando llegamos llegamos crónic

P22: 13. Transcripcion Maria Ines Pantoja.docx - 22:20 [Mire la primera barrera es las..]  (103:103)   (Super)
Códigos:	[Capacidades del sistema : accesso a la salud] [cobertura] 
No memos

Mire la primera barrera es las citas médicas, si a usted no le abren esas puerta  de citas médicas, no tiene nada, porque no tiene exámenes ni tratamiento. De la principal barrera es el acceso, nosotros tenemos una encuesta que hicimos con el ministerio sobre barreras y esa encuesta empieza por el acceso, entonces las cita médicas, los exámenes, luego la remiso al especialista, las citas con especialista, los exámenes, el tratamiento. Entonces si a mí no me abren esta puerta para ingresar al sistema es muy difícil porque después no entro, y después aquí otra hay otra puerta que es entrar donde el especialista, y esto se demora de 6 a 8 meses y después donde el especialista la barrera para los exámenes, los que están en el POS y los que no están en el POS. Luego la barrera para procedimientos porque como no hay red los procedimientos los tienen que pagar por evento , y eso sale costosísimo, porque ya las IPS no quieren prestar servicios si no son pagos por adelantado, entonces ya son unas barreras que el paciente tiene, eso es toda una cadena y luego ya en la continuidad del tratamiento también hay barreras de acceso, porque aquí el médico me ordeno el no POS por 6 meses, para probar el no POS la ley dice que son 2 días hábiles, pero las EPS abusivamente son 10 o 15 días para entregarle al paciente la orden del medicamento. Entonces a mí el no POS me lo aprobaron por 6 meses, entonces en este momento las EPS que están haciendo, volviéndome a que yo tengo que pedir otra autorización para yo poderlos reclamar, ósea cada vez tengo que averiguarlo, eso es ilegal eso no aparece en ninguna norma, entonces mientras me dan la autorización yo puedo perder hasta 15 días de tratamiento, hemos hecho el ejercicio de perdida de tratamiento y los tratamientos van de dos meses a 6 meses, ósea que las EPS se están ahorrando una UPC de 6 meses, o de dos meses dependiendo. Ósea que la barrera es una cadena que tiene la persona.


P22: 13. Transcripcion Maria Ines Pantoja.docx - 22:21 [Lo que yo veo es que esto cada..]  (107:107)   (Super)
Códigos:	[Capacidades del sistema : accesso a la salud] [cobertura] 
No memos

Lo que yo veo es que esto cada día se está aumentando más, han estado peor las barreras de acceso al sistema, hemos hablado del secretario de salud, de Capitalsalud, cuatro meses sin atender a los pacientes, nos prometió que la otra semana iba a empezar. Es un desgaste yendo a las EPS con la Supersalud, y como que nadie ha encontrado la solución, yo veo que esto esta cada día empeorándose, es una bomba de tiempo que hay muy grande

P22: 13. Transcripcion Maria Ines Pantoja.docx - 22:22 [Yo pienso que por el maltrato ..]  (115:115)   (Super)
Códigos:	[Capacidades del sistema : accesso a la salud] [cobertura] 
No memos

 Yo pienso que por el maltrato que se le da a la gente, prefieren quedarse en su casa tomando agüita de hiervas que ir al médico,  por el nivel cultural también las personas del régimen subsidiado son personas que no tienen una cultura y son personas muy vulnerables entonces yo pienso que prefieren quedarse en su casa enfermos, porque es insólito que el régimen subsidiado sean menos, porque es gente que tiene menos condiciones saludables, en el régimen contributivo la gente vive un poco mejor, tienen un poquito más de calidad de vida, dependiendo del nivel en que este, influye el maltrato que se le da a la gente, también el nivel de cultura, y esa gente a veces más bien acude a yerbateros a otra case de atención pero no acuden a los médicos. Por ejemplo entre los indígenas, ellos prefieren acudir a sus sabios a sus chamanes, que a los médicos porque en el chamán encuentran uno igual y al ser igual uno confía más en la persona que es igual a uno, que en el que esta allá con prepotencia, se siente menos confianza. Yo estuve hace poquito en un resguardo indígena en Tibu, precisamente recogiendo esto de las barreras, esa pobre gente vive en una mísera cafesalud tiene allá atiende a casi la mitad de la tribu, y no tiene oficina allá en Tibu, y los indígenas tienen que venir a doce horas a Tibu y ahí no encuentran de cafesalud una oficina que los atiendan para darle la cita, y tienen que ir hasta Cúcuta. Entonces como se va a ir esa persona con esa pobreza que tienen, él va donde el chamán y le da cualquier cosa, yo digo que esa es una de las causas. Hay una tribu que tiene mucha tuberculosis y otra cirrosis de las dos comunidades de los indígenas motilones Bari, hay una comunidad que tiene prevalencia de hepatitis de cirrosis, y otro que tiene de tuberculosis, entones ellos me decían a mi como me voy a ir hasta Cúcuta a pedir citas entonces la enfermedad esta avanzando entre ellos. Cuando está muy grave lo llevan al hospital y si no encuentran a alguien que los ayude a entrar los dejan 3 o 4 días en urgencias, eso le tienen ellos pavor, llegan y los mandan a un sitio donde ellos tienen para atenderlo que eso si tienen, y duran allí meses sin poder llegar a sus familias porque no le dan la cita con el especialista, entonces la gente dice a qué voy. Voy a gastar u mes que se me daña los cultivos, entonces prefiero quedarme acá y no buscar atención. Y los indígenas son del subsidiado que poco consultan este servicio


P22: 13. Transcripcion Maria Ines Pantoja.docx - 22:23 [Ojala tuvieran red, pero no ha..]  (119:119)   (Super)
Códigos:	[Capacidades del sistema : accesso a la salud] [cobertura] 
No memos

Ojala tuvieran red, pero no hay red es que, que red van a tener si no tienen por lo menos una oficina en Tibu para atender a los indígenas, hay para el contributivo mas no para el subsidiado. 

P23: 14. Transcripcion Mario Hernandez.docx - 23:4 [Pues es decir este tipo de cos..]  (7:7)   (Super)
Códigos:	[cobertura] 
No memos

Pues es decir este tipo de cosas no se dan de manera natural, no es que haya una tendencia obvia y natural que siempre se va dando por inercia en inflación, tasa de desempleo e informalidad, no yo creo que acá hay una articulación entre cosas planeadas y cosas no planeadas primera cosa, y segundo hay un gran impacto de una orientación política que va impactando este tipo de variables. A mi manera de ver la forma como se ha venido trabajando en la política pública desde la década de los 90, es que se quiere hacer un control de la inflación a toda cosa a través de un mecanismo monetarista que en principio define una lógica de masa monetaria vs tasas de interés, y las tasas de interés y también la tasa de cambio influye mucho, va definiendo supuestamente una serie de estímulos para fortalecer el sector financiero, que es el motor de las económicas actualmente en el régimen de financiación, y que implica entonces pues simplemente buscar que en esas tasas de interés se conserve el capital y eso mantenga un cierto control de la tasa monetaria disponible, ya no es el banco de la republica el que emite y sostiene y dice yo quiero llegar hasta allá, si no esta lógica de un mercado financiero que define las cosas, es una política monetarista que viene impulsándose desde hace mucho tiempo, por eso yo creo que al contrario tendera a disminuir la inflación a toda costa si sigue esa política monetarista. El desempleo no depende tanto de ese elemento si no que depende de otro asunto que es como se genera puestos de trabajo con cierta estabilidad con cierta posibilidad de generar riqueza en el sentido de un valor real en la economía y eso con la tendencia que hemos venido teniendo de reprimarizacion de la economía, y de una financiamiento de la relación comercial transnacional, pues Colombia se está convirtiendo cada vez más en un gran san Andresito, donde no se necesita unos empleos estables, al contrario es más bien una gran informalidad del empleo que seguramente seguirá aumentando y por tanto desempleo e informalidad seguirán teniendo la tendencia que hemos visto hasta ahora, si continua insisto ese modelo de desarrollo que han venido implantando. Esa es mi manera de ver el problema.


P23: 14. Transcripcion Mario Hernandez.docx - 23:6 [Pues mire el modelo como esta ..]  (15:15)   (Super)
Códigos:	[cobertura] 
No memos

Pues mire el modelo como esta puede perfectamente seguir diciendo que tiene una cobertura casi total, porque es una cobertura de aseguramiento que no implica necesariamente acceso real y efectivo, oportuno y de calidad resolutivo y todas esas cosas que uno le pone al acceso de los bienes y servicios en salud, pues la cobertura ya es prácticamente universal y eso se va a seguir manteniendo porque, el sistema todo esta estructurado así, un seguro para pobres un seguro para medios, que son los no pobres todo aquel que te reciba más de un salario mínimo ya es no pobre y una cosa complementaria que de todos modos si deja una diferencia para quienes puedan pagar medicina prepagada, entonces con ese modelo todos tienen un seguro, serán muy pocas personas los indigentes que no están en régimen subsidiado o los que están en un tránsito por ahí, pero por lo general aumenta la flexibilidad laboral, ya no se necesita tampoco que haya un empleador, se obliga al trabajador independiente que tenga cualquier tipo de ingreso, ya se puede detectar muy fácilmente con los sistemas de información entonces queda obligado todo el mundo a aportarle algo al sistema y quedar en el régimen contributivo o demostrar su grado de pobreza, nivel 1 y 2 para quedar en el régimen subsidiado que cada vez serán menos, los del régimen subsidiado, porque simplemente se aumenta o más bien va exigiendo cada vez un umbral más bajo como un puntaje más bajo de sisben de manera que son menos población que tienen menos derecho a exigir el régimen subsidiado, eso ya ha pasado tres veces, vamos en la tercera encuesta de sisben, y cada vez sacan más gente de pobre, eso es una forma de disminuir la demanda al régimen subsidiado. Y por otra parte a todo el que tenga cualquier ingreso se va a tratar de monetarizar las cosas para tener información de cualquier vendedor de dulces cuanto se gana, y se le entre una lógica que dice si usted pone platica, 20.000 pesos mensuales el estado le da 20.000 pesos para que tenga su bono pensional, esa es una forma de registrar a todo individuo que tenga más de 20.000 pesos diarios o bueno 20.000 pesos mensuales o como sea, ahí se va bancarizando se va identificando quien está pagando cosas y a ese se le va a decir usted debería pagar entonces su salud, ahora cualquier contrato de prestación de servicios ponga su porcentaje de ARL así no sepa que es un riesgo laboral así nunca conozca que está en la tabla ARL, no sabe cómo exigirle derechos allá pero aporta, y en salud obligado totalmente y en pensiones obligado totalment

P23: 14. Transcripcion Mario Hernandez.docx - 23:7 [O incluso puede que se manteng..]  (19:19)   (Super)
Códigos:	[cobertura] 
No memos

O incluso puede que se mantenga pero tiende a disminuir la del subsidiado y a aumentar la del contributivo en todas partes y con eso se mantendrá una cobertura universal, eso no tiene ningún problema porque ese es el aseguramiento de cualquier manera obligatorio lo que está funcionando sin importar si usted pueda acceder a estos, se supone que el asegurador tiene que garantizar su acceso y para eso se le van a alinear los incentivos dándole una parte de la UPC por resultados que ese es el otro instrumento supuestamente para que las EPS finalmente haga lo que tiene que hacer que es gestión del riesgo en salud. 

P23: 14. Transcripcion Mario Hernandez.docx - 23:8 [Pues digamos comparativamente ..]  (22:22)   (Super)
Códigos:	[cobertura] [Protección financiera : financiación del SGSSS] 
No memos

Pues digamos comparativamente es bajo con respecto a otros países entonces ha generado otra forma de control del gasto que afecta obviamente la oportunidad la calidad la pertinencia, todo y pues claro uno tiende a pensar entonces aumentemos la UPC para que aumente otra cosa, pero si la lógica de la regulación del mercado de aseguramiento en Colombia consiste en mantener un mismo valor por persona a cambio de un paquete de servicios pues el asegurador trata todo el tiempo de no gastarse ese valor, es decir hace una cantidad de mecanismos para recibir la UPC pero no gastársela en la prestación directa de los servicios, eso es lo que explica realmente todas las barreras de acceso que hay, toda la falta de oportunidad que hay, toda la falta de calidad, porque ahí se concentra su esfuerzo de rentabilidad, mientras eso no cambie auméntele la UPC todo lo que quiera y eso es como echar cada vez más en un saco roto, unas venas abiertas ahí tras hundir a la gente. Entonces sinceramente esa lógica es la que debemos discutir a fondo porque a mi juicio eso siempre mantiene una perspectiva de control del gasto, que se distancia fuertemente de las necesidades de la población, claro no puede controlar el gasto pero en función de necesidades reales epidemiológicas demográficas etc., pero no en función de necesidades de mercado y de rentabilidad de los otros sectores que se lucran con la atención en salud. 


P29: 18. Transcripcion Carlos Hernandez.docx - 29:2 [La cobertura está bien pero no..]  (15:15)   (Super)
Códigos:	[cobertura] 
No memos

 La cobertura está bien pero no es una cobertura real es una cobertura numérica, cuando uno va a decir cobertura real efectiva de servicios va a encontrar que las poblaciones no hay inversión en los municipios y veredas siguen siendo hospitales públicos de toda la vida, la gente recibe según lo que oferte entonces el traslado de esos subsidios en oferta y demanda no es cierto porque cuando van a encontrar encuentran el mismo hospital público donde los aseguradores no hicieron desarrollo porque no hay interés y no genera lucro, entonces de que sirve cobertura si la que es efectivo no se da. Entonces no hay una garantías explicita que los derechos y servicios que la gente tiene va acompañado con el estímulo para que la red lo haga y porque la red no lo hace, porque la red pública digamos el pequeño municipio no tiene las escalas para hacerlo y la red privada si no existe un volumen y una tarifa diferencial que genere renta no lo hace, Colombia ha aumentado muy bien la red de servicios en las grandes capitales no en los municipios pequeños. 

P31: 20.Transcripcion Hellen Mendoza.docx - 31:4 [cobertura el concepto de cober..]  (19:19)   (Super)
Códigos:	[cobertura] 
No memos

cobertura el concepto de cobertura debe cambiar, nosotros los colombianos decimos y nos llenamos la boca diciendo que somos s uno de los pocos países del mundo con una cobertura casi del 100%, pero cuando nosotros miramos la cobertura y miramos a que tienen derecho nuestro pacientes vemos que una cosa es el contributivo y otra cosa es el subsidiado y a su vez una cosa es el subsidiado en las cabeceras municipales y otra cosa es el subsidiado en la red dispersa en los municipios y las veredas. Un paciente con un carnet de subsidiado puede tener acceso a una consulta de especialista y la pueden dar en 6 meses, entonces usted tiene una cobertura supuesta porque tiene un derecho a un servicio pero que no se está prestando efectivamente, entonces yo pienso que nosotros debemos definir lo que para nosotros es cobertura y que no sea una cobertura de tener el derecho si no que ese derecho de vedad sea efectivo.

P31: 20.Transcripcion Hellen Mendoza.docx - 31:25 [HM: Bueno, que barreras hay to..]  (130:130)   (Super)
Códigos:	[cobertura] 
No memos

HM: Bueno, que barreras hay todas esas barreras las hay en barreras de oferta, disponibilidad de recursos médicos y distancia al centro como le dije cuando arrancamos esta conversación, la cobertura y la capacidad instalada en este país no está distribuida de manera adecuada, recursos mecidos hoy hay una población importante, hablemos de Cali hay una población de mujeres embarazadas y de niños que no tienen acceso a un recurso medico porque cada vez hay menos pediatras y menos ginecolopstetras en las IPS, para atender la baja complejidad que es lo que no da plata. Entones el acceso no lo hay, ahora estamos en poico epidemiológico en enfermedades respiratorias del niño esta es una pasadita por club Noel, yo hice una pasadita por urgencia en Versalles y es una cosa enloquecedora, no hay donde atender a los pacientes, institucionales por tramites de atención también, nosotros tenemos una realidad y es que hay unas EPS que han derivado y han hecho integraciones verticales totales con IPS tipo clínica nuestra, o lo que antes era la corporación Comfenalco unilibre, entones esos 100% de esos pacientes se atienden allá, si usted está afiliado a una EPS de esas y llega a una clínica cualquiera a usted no lo van a atender allí, entonces la IPS que lo recibió a usted tiene que hacer unos trámites y toda unos movimientos de referencia para mandarlo a la clínica que lo van a atender eso que significa que la IPS pierde recursos propios que nadie le va a reconocer y el paciente puede estar perdiendo un tiempo valioso para recuperación e incluso para su vida . la calidad del servicio esto es teoría agente principal, cuando a usted le dan una plata por atender un paciente usted puede dilatar los procedimientos quirúrgicos, puede utilizar medicamentos con menos calidad, usted puede hacer muchas cosa dentro de la atención del servicio que al final van a redundar de pronto no inmediatamente pero en un tiempo posterior en la calidad de ese paciente, usted puede estar trabajando un medicamento para la predion o usar un genérico de buena calidad o un genérico gatofolio, eso que significa que si usted le está dando un antihipertensivo de baja calidad ese paciente se le va a infartar en unos años, lo que pasa es que hoy como esta trabajado con un modelo de cápita o un modelo donde la transferencia de riesgo lo está usted orientando a tratar de mejorar su rentabilidad financiera, usted está buscando mecanismos para mejorar su flujo de caja, usted le está dando al paciente el medicamento se lo da cada 30 días, cual es la calidad de la molécula el principio activo que al final es lo que determina cual va a ser la enfermedad de un futuro,, hoy nosotros no estamos evaluando la evitabilidad, por eso le digo el MIAS así lo arranquemos hoy antes de 20 años no vamos a tener nada diferente. En barreras de demanda falta de dinero si y no, porque hoy los colombianos estamos acostumbrados a que todo se hace por tutela entonces hoy que la falta de dinero no es una excusa, es más si usted va hoy y dice que no tiene para pagar el copago. 

P31: 20.Transcripcion Hellen Mendoza.docx - 31:26 [HM: Por supuesto porque el gas..]  (133:133)   (Super)
Códigos:	[cobertura] 
No memos

HM: Por supuesto porque el gasto de bolsillo aumenta a lo que hay que apostarle es que en la medida que se cambie el modelo y en la medida que el post se disminuya ya se convierte, estas son las inclusiones porque las exclusiones ya no existen, en medida que el MIAS se vaya implementando baja la carga de enfermedad al bajar la carga de enfermedad, el gasto de bolsillo se disminuye por lógica ya no tengo que comprar cosas por fuera porque ya estoy sano, aprendí a comer bien, tuve un modelo que desde la infancia me levanto para., estos 20 años de transición van a ser los más duros porque el colombiano va a tener que pagar hoy cosas que nunca han pagado. Vamos a tener los dos modelos, no podemos irnos para el MIAS olvidando el modelo curativo porque hay gente que necesita el modelo curativo y las EPS necesitan estabilizarse financieramente y hoy en lo que yo veo no creo que el sistema el gobierno acabe las EPS, acabar las EPS para devolvernos la modelo que tuvimos antes del 93 sería un retroceso demasiado grande, no creo que lo hagamos

______________________________________________________________________

Código: Desigualdades en salud {10-0}

P11: 4. Transcripcion Armando Gonzales_ok.docx - 11:17 [Deben cambiar porque lo que te..]  (97:97)   (Super)
Códigos:	[Desigualdades en salud] 
No memos

 Deben cambiar porque lo que te digo uno como que no ve que el desarrollo del país avance, lo ve en unas áreas, pero no lo ve en áreas apartadas y las personas se van a seguir embarazando y van a tener muy poca atención en ese embarazo y eso las puede llevar a riesgos muy grandes, uno no lo visualiza. Lo de la mortalidad infantil es igualitico, la misma cosa si no hay obstetras, tampoco van a haber pediatras ni el mismo médico general y a mí me ha tocado ver en veredas de acá del valle que es un departamento "desarrollado" atendiendo a los pacientes por un auxiliar de enfermería, y lo hacen muy bien yo hice una visita a cualquiera de estas áreas y me quede aterrado de lo que esa señora, porque una persona a nivel de auxiliar sea capaz de hacer un diagnóstico de todas las personas, llevarlas por diagnósticos para ver que tiene dos epilépticos y esos dos los llevaba a san isidro y hacerle una descripción total de las condiciones de salud de esa es mi paciente, estoy hablando de bastantes años cuando yo hacía salud pública, y dije que es esto, el personal auxiliar de enfermería que podía hacer un diagnóstico perfecto, el pozo séptico mire que lo tengo separado aquí del afluente de agua pero está bien separado para que no contamine, una cosa que esa clase de salud pública que en mi vida la escuche de una auxiliar nunca se me va a olvidar. Entonces yo dije la potencialidad tan tremenda que tiene la gente y la potencialidad que tienen de sobrevivir en condiciones difíciles. Tú me dices yo nací en una aldea en una población pequeña, no se allá que persona las atendía pero mira que bastante bien, entonces hay otra cosa que no se puede decir en la grabación, que son cosas que son diferentes a lo clásico que estamos mencionando.

P12: 5.Transcripcion Carlos Fajardo_ok.docx - 12:4 [La capacidad actual del sistem..]  (14:14)   (Super)
Códigos:	[Capacidades del sistema : accesso a la salud] [Desigualdades en salud] 
No memos

La capacidad actual del sistema que está instalada sigue siendo muy dispar tenemos un gran problema en el departamento del valle donde hacemos presencia, con un hospital universitario donde sobre todo lo que es correspondiente a la baja complejidad, no hay instituciones que hoy resuelvan toda esa problemática de salud. Lo mismo pasa en Nariño, los niveles de alta complejidad los hospitales municipales no existen y eso hace que toda esa carga de enfermedad de la baja complejidad este yendo a los grandes hospitales, entonces se congestionan los hospitales universitarios, se congestionan el hospital San Pedro, se congestiona el Hospital Universitario del Valle, se congestiona fundación Valle del Lili, porque los usuarios pretenden siempre resolver a través de las urgencia,  resolver problemas que debieron haberse resuelto en el primer nivel, que es donde realmente no hay resolutividad en esa parte. 

P12: 5.Transcripcion Carlos Fajardo_ok.docx - 12:23 [Claro que sí, aunque quiero ac..]  (126:126)   (Super)
Códigos:	[Desigualdades en salud] 
No memos

Claro que sí, aunque quiero aclarar que los indicadores de mortalidad materna en Colombia bajaron, cuando entra la ley 100  bajaron. Hoy tienen unos indicadores y hay unas poblaciones muy altas, pero hay poblaciones que han bajado totalmente porque la atención del parto, la atención materna, se volvió institucional, pongo el ejemplo de la comunidad Awa en Ricaurte, hay muchas personas de esa población que están siendo atendidas a través del parto institucional, en la misma costa hay mucha población que tiene sus consultas preventivas. Pero que se llegue al 100% y en los 20 años no creo, porque hay un problema de accesibilidad, porque todavía no hay los accesos que faciliten esto, exceptuando que el gobierno vaya a generar unos programas especiales para darle una solución, que no lo veo todavía muy cercano que el gobierno vaya a destinar recursos. Ahí tenemos el problema que sacan por todos los medios de la guajira, como los niños de una población indígena se mueren a cada rato por desnutrirse, o lo que pasa en el choco

P16: 10.Transcripcion Angela Tascon.docx - 16:15 [No pues, eso va con una inters..]  (62:62)   (Super)
Códigos:	[Desigualdades en salud] 
No memos

No pues, eso va con una intersección como en una red con todo, entonces como llego yo a los sitios más alejados con MIAS, es decir acá no hay prevención para nada sabemos lo del cambio climático y llegan los aguaceros y barren con todo. Es como que las debilidades son por varios ángulos que apuntan a un bienestar general, entonces como llego a las regiones apartadas si los muchachitos se demoran 3 o 4 horas en llegar a la escuela. Si el promotor no llega, si no hay agua, entonces ese es un tema que se me paso, el sistema de los servicios públicos, la verdad no me acuerdo pero el porcentaje poblacional que no le llega agua potable es muy alto, entonces allí vienen  muchas enfermedades de tipo digestivo

P16: 10.Transcripcion Angela Tascon.docx - 16:16 [Ni buenaventura, que es el pri..]  (66:66)   (Super)
Códigos:	[Desigualdades en salud] 
No memos

Ni buenaventura, que es el principal puerto del pacifico colombiano y la gente uno de sus mayores problemas es de agua, entonces yo digo que el proyecto como tal es muy bueno pero debe ir pegado de las otras cosas, porque que me gano yo con decirle coma bien y lávese las manos, pero no hay. 

P18: 11. Transcripcion Fabian Mendez.docx - 18:22 [Afortunadamente lo que uno ve ..]  (95:95)   (Super)
Códigos:	[Desigualdades en salud] 
No memos

Afortunadamente lo que uno ve y lo que encuentra es también diferencias marcadísimas entre lo urbano y lo rural, y entre las etnias usted es negro o aborigen le va peor en muchos de los indicadores, yo creo que a futuro va a depender mucho de lo que pase en la política agraria del país yo cada vez más soy un convencido de que la industria es de las peores cosas que le han pasado al país, y en el valle del cauca la industria es súper negativa pensando en la sostenibilidad alimentaria, este valle del cuica no va a ser sostenible mientras tengamos una industria de la caña, el 80% de los alimentos que nosotros consumimos son producidos por el 20% de las tierras que manejan los pequeños agricultores. Esta promesa que hizo la agroindustria y esa falacia que nos han vendido que solo la agroindustria permite el desarrollo de suficiente alimentación para la población es falso, lo que la agro industria ha hecho es producir alimentos para las vacas y ahora cada vez más biocombustibles, en ese sentido lo rural pasa por una verdadera defensa de los pequeños agricultores, pasa por la defensa de los recursos naturales y digamos esos habitantes con un país cada vez más urbanizado deberían ser protegidos al 100%, eso es la base que hoy podemos tener para tener agua, para tener alimentos. Entonces si este país no hace un camino importante ahora que yo creo que la firma de la paz abre una posibilidad, pueden ser muy buenos pero también pueden ser muy malas, y es que sin el freno de las FARC muchos recursos naturales van a estar para este espíritu depredador que tiene el hombre y lo que estamos viendo en el pacifico, la pelea que tenemos con el glifosato, es cierto e imagínese un ministro de salud defendiendo que no se utilice el glifosato para la eliminación de los cultivos de coca, pero tenemos acá mismo el problema del glifosato en el norte del cauca y en el sur del valle, entonces estamos desarrollando un proyecto con comunidades afro e indígenas, comunidades que la avioneta pasa 3 veces a  la semana rosea glifosato y comunidades digamos que se han visto cada vez más atrapadas y están en este proceso de estar en el despojo de muchos años, décadas y ahora como me decía una negra de acá del tiple, yo antes salía al patio y encontraba plátano yuca, y ahora tengo que ir a la tienda y me vale 1000 pesos un plátano, los cultivos se acabaron. Entonces si no tenemos una política clara de defensa de estos pequeños agricultores que son los que repito producen la base de la seguridad alimentaria de las ciudades estamos mal. Estas diferencias seguirán. 

P19: 12. Transcripcion Fabio Osorio.docx - 19:15 [Es triste que la gente de los ..]  (74:74)   (Super)
Códigos:	[Desigualdades en salud] 
No memos

Es triste que la gente de los corregimientos tengan que padecer, acá llega gente que sale en lancha monta en caballo, y dura casi 6 horas para llegar a un hospital y que le digan que no hay cita porque no hay médico. Si desde ahora no se toma medidas oportunas para que eso cambie seguramente las personas de los pueblos seguirán padeciendo, hay que mandar al pueblo al vigía de la salud, al médico familiar, el gobierno tiene la capacidad ahora que dice que no hay conflicto y no hay plata para la guerra, esa plata inviértanla en esas personas que visitaban las veredas y detectaba al paciente con tiempo para que con tiempo llegar a la ciudad a hacerse el tratamiento, pero acá se privilegia viviendas, ya no se tiene el discurso de la guerra, que también son válidas, pero puede tener educación seguridad, pero si no tiene salud nada de eso va a servir, la salud no lo es todo pero sin ella yo estoy seguro que no somos nada y si el gobierno dese ahora no empieza a trabajar de esas partes aisladas desde la prevención hasta ese médico que iba a la vereda podríamos mejorar de resto todo va a seguir igual en los 20 años.

P22: 13. Transcripcion Maria Ines Pantoja.docx - 22:9 [Mira nosotros hicimos la pregu..]  (27:27)   (Super)
Códigos:	[Desigualdades en salud] 
No memos

Mira nosotros hicimos la pregunta cuando estuvimos en toda la nivelación de planes de beneficio, estuvimos en el lanzamiento, y todo eso que hicieron y nosotros estuvimos hablando lo de la UPC  que había un desequilibrio entre la UPC del contributivo y del subsidiado y la respuesta que nos dieron era que el régimen subsidiado no paga incapacidades, que ahí se basaba en la diferencia, aunque la UPC lo que se quieres es que se iguale, entonces porque no pagaban incapacidades, eso es verdad el contributivo tiene que pagar incapacidades en cambio el subsidiado no, que esa era la diferencia.

P26: 16. Transcripcion Senador Ospina.docx - 26:14 [Si se detiene en términos de d..]  (58:58)   (Super)
Códigos:	[Desigualdades en salud] 
No memos

Si  se detiene en términos de determinantes sociales que genera una mayor demanda de bienes y servicios en salud, en las zonas periféricas del país, todo el mundo lo sabe que si no existe agua pues vamos a tener diarrea si no existe calidad de alimentación vamos a tener desnutrición, si maneja el tema de la violencia vamos a tener trauma, si no se maneja el tema de los vectores vamos a tener malaria chicunguña, paludismo, dengue. Uno pensaría que hacia esas zonas periféricas la única manera de hacer sostenible el sistema es en la medida que se intervenga las determinantes de salud que las condiciona, talvez el momento que vivimos de postconflicto y acuerdo posibilitaría la intervención de esas determinantes.

P30: 19. Transcripcion Felipe Garcia.docx - 30:22 [FG: El problema viene de que e..]  (109:109)   (Super)
Códigos:	[Desigualdades en salud] 
No memos

FG: El problema viene de que el hecho de que nuestra estructura es centralista, mire yo no voy a decir que estados unidos es un paraíso pero la estructura federativa es mucho más equitativa en términos de modelo de salud que un modelo centralista, México también y está más repartido tiene lógicamente estados pobres y ricos pero tiene como una cierta estratificación adaptativa, pero Colombia como es un estado tan central y ese es el principal problema, salga de las principales ciudades Cali Bogotá, Medellín, barranquilla, Bucaramanga, algo de santa marta, de ahí para allá esta todo en la periferia la periferia tiene inclusive hundido el estado es que una delas causas de porque Colombia ha tenido esa guerrilla tan históricamente una de las causas es el olvido del estado en esas zonas y eso es innegable, entonces usted ha tocado algo que es importante y es que el modelo esta segmentado según la medicina occidental, factual, causa efecto, directa que es la medicina occidental es el gran problema de la medicina actual. Me duele la cabeza démosle un fármaco para que evite el dolor de cabeza pero el problema no está en la cabeza, ese aspecto las otras medicinas que llaman alternativas juegan un rol más abierto, complementario a lo que llaman la medicina alopática y complementarias porque inclusive, uno dice bueno la medicina china si no funcionara no habría chinos, todos estarían muertos pero hay mil y pico de millones de chinos que se tratan con medicina de china y es la demostración de que esa medicina es eficaz también. Nos enseñaron a la medicina occidental de efecto causa, completamente una causa la produce un efecto, entonces si le duele la cabeza el problema está en la cabeza pero la medicina china tiene otro universo y otra manera de ver esa disfunción en la cabeza y el problema puede estar en que uno de esos flujos de energía, de esos meridianos, no es que sea agujita acá y acá y funciona, yo he sido paciente y funciona me quita el dolor. Entonces el problema en Colombia es que se centró en una medicina completamente alopática, factual esa medicina que te cobra por la causa y el efecto, y todo el sistema administrativo toda la tecnología está en eso. En México la están introduciendo la medicina no tradicionales como parte del sistema de salud de ellos, acá se ha intentado pero ha habido mucha resistencia, entonces cuando usted nació en Tumaco había una tradición de prácticas médicas, las parteras la medicina del chaman o de lo que sea, que son conocimientos milenarios y ancestrales, yo siempre he dicho si la medicina antes de la occidental no existieran no habrían humanos, nos hubiéramos muerto. Lo que sí ha hecho la medicina occidental es ir a un diagnóstico más enfocado menos empírico sobre la mase más fisiológica, no voy a negar la fisiología una enfermedad se debe a que una función fisiológica está fallando, una falla fisiológica es que un x sistema no funciona bien por ciertas cosas. Las medicinas chinas lo ve de otra forma, en ultimas llega a lo mismo pero esta es más integral y esta es más causa efecto ese es el problema. Entonces un sistema que se centra sobre eso es un sistema que va a fallar tarde o temprano para mí, porque se centra en un sistema comercial de proveedores y a los antojos de las farmacéuticas. Mire yo tengo muchos amigos, llega el visitador médico doctor este es el nuevo remedio para la diabetes, y si usted lo receta le damos unos bonitos y si tiene que irse a una reunión a México nosotros le ayudamos, entonces se vuelve como un incentivo no digo que sea antiético, para que esa persona escriba de ese remedio y entonces toda las famosas internacionales farmacéuticas están punzando el sistema, mire cuando llegó el zika compraron no sé qué tanto millones de dosis de un fármaco que combatía el zika, que había detrás de es

______________________________________________________________________

Código: modelo de salud actual {91-0}

P 8: 1. Transcripcion Martha Lucia  ospina.docx - 8:1 [del sistema de salud, en su co..]  (7:7)   (Super)
Códigos:	[modelo de salud actual] 
No memos

del sistema de salud, en su contexto

P 8: 1. Transcripcion Martha Lucia  ospina.docx - 8:2 [Y hay un común denominador y e..]  (7:7)   (Super)
Códigos:	[modelo de salud actual] 
No memos

 Y hay un común denominador y es que la gente en general no tiene claro que es un sistema de salud, y hacemos un revuelto de los roles que tiene el sistema y por eso somos por un lado tan injusto con el sistema y por otro lado tan permisivos con el sistema en otras cosas. Entonces los sistemas de salud e cualquier país del mundo tienen 3 grandes objetivos y el de Colombia también,

P 8: 1. Transcripcion Martha Lucia  ospina.docx - 8:3 [uno de los objetivos es evita ..]  (7:7)   (Super)
Códigos:	[modelo de salud actual] 
No memos

uno de los objetivos es evita la quiebra financiera de la gente, por eso el sistema de salud es aquel que me permite yo no tener que vender la casita para pagar una enfermedad, ejemplo un cáncer, no vender la vaca que es mi sostén no quedar en la calle, ese respaldo financiero que lamentablemente es además ignorado y casi que es un tema

P 8: 1. Transcripcion Martha Lucia  ospina.docx - 8:4 [una de las grandes funciones y..]  (7:7)   (Super)
Códigos:	[modelo de salud actual] 
No memos

una de las grandes funciones y por eso hay entidades detrás del aseguramiento que en nuestro caso son las EPS, pero así desaparecieran siempre habrá un alguien que hace las veces de aseguramiento, eso es totalmente necesario en cualquier modelo de aseguramiento y eso se sabe, nosotros siempre tendremos un asegurador y tendremos el sistema de financiamiento y el sistema de cuentas del estado y el ministerio en su rol de coordinador del aseguramiento

P 8: 1. Transcripcion Martha Lucia  ospina.docx - 8:5 [La segunda función que tiene q..]  (7:7)   (Super)
Códigos:	[modelo de salud actual] 
No memos

La segunda función que tiene que ver con la calidad de vida y el sistema de seguridad en salud apoya a la seguridad y lo hace mediante la seguridad sanitaria, entonces tener esas garantías para la vida que tenemos, esas garantías que se traducen en lo cotidiano y se nos olvida, nosotros no hervimos la leche hace cuanto años, no hervimos el agua, se nos olvida que eso es un avance de la seguridad sanitaria del país, y ese es el sistema que esta actuando y no abrimos la almohada para ver que tiene por dentro de relleno, damos por hecho que tiene una cosa segura, y a un niño no dudamos que el pañal le va a hacer daño, ni nunca pensamos que el medicamento que nos vayamos a tomar no sea el medicamento, porque hay alguien detrás

P 8: 1. Transcripcion Martha Lucia  ospina.docx - 8:6 [la tercera línea es sin duda e..]  (7:7)   (Super)
Códigos:	[modelo de salud actual] 
No memos

la tercera línea es sin duda el tema de atención, pero que se atiende, también se nos olvida el daño en salud, uno atiende es el daño la consecuencia, y el daño en salud no se produce en su mayoría en el sector salud, el sector salud es un sifón que recibe el daño proveniente, un país que tiene la tasa de actividad física de la región solo el 26% de los niños en edad escolar tienen asegurada la atención física, porque no está asegurada por el ministerio de educación que la educación física sea algo obligatorio en los países, porque en Colombia se confunde la actividad física con el deporte, el niño que no es talentoso es rechazado y la clase de educación física no existe, y se sustituyó por deporte y es la clase a sacrificar, entonces eso nos está labrando un camino hacia la enfermedad cardiovascular. Aunque tenemos logros como la disminución de tabaquismo, tenemos poblaciones como los adolescentes con algo consumo de tabaquismo. Tenemos una obesidad creciente, y una obesidad que es de pobres y mujeres entonces es un país que no está trabajando por mitigar el daño en salud, y el programa deportivo es a la vuelta de la esquina, coge el riesgo cuando ya está ahí, pero el estilo de vida las condiciones que determinan la calidad de la vida y en la salud que tendremos, no está en el sector salud y los otros sectores, no lo han entendido y no lo ven asociados, por ejemplo los polideportivos del barrio no pueden ser utilizados porque lo atracan, porque lo que se necesidad es un tema de control policial y de aseo para que el polideportivo pueda ser usado. Entonces ese tipo de cosas no estamos garantizando esas condiciones que lleven a una buena cantidad en salud

P 8: 1. Transcripcion Martha Lucia  ospina.docx - 8:7 [al tercer punto, atención al d..]  (7:7)   (Super)
Códigos:	[modelo de salud actual] 
No memos

 al tercer punto, atención al daño en salud y lo vemos todos los días es que el servicio de salud no me atiende, que no hay citas, y entonces se traslada a servicio de salud una cantidad de culpas que no tiene por ejemplo, con los especialistas es que no hay suficientes, y no los hay porque ellos mismos se autorregulan los cupos son limitados, y así el estado haya metido la mano para que hayan más cupos por los motivos que sean ellos se autorregulan ósea que los cupos son limitados y no hay incentivos y cohesiones para que ellos se ubiquen en zonas rurales, entonces se concentra el 98 de ellos en la zona andina y claramente esas regiones nunca le darán especialistas.

P 8: 1. Transcripcion Martha Lucia  ospina.docx - 8:8 [La presión tecnológica, una pr..]  (7:7)   (Super)
Códigos:	[modelo de salud actual] 
No memos

La presión tecnológica, una presión de la industria de una manera descomunal e invisible, que hace que se demande lo último en tecnología para todos y ya, sin que necesariamente sea costo benéfico, entonces una presión en la zona financiera terrible matamos a un mosquito con un cañón, habiendo tecnologías de tan bajo costo y unas muy buenas, un ejemplo de un hipertensivo muy sencillo, los inhibidores de la eca, son hipertensivos que son muy buenos, pero al otro día que perdieron la patente al otro día no servían para nada porque perdieron la patente de la casa original que les comercializaba, y la misma visita médica s encargo de desprestigiarnos y decir que ahora todos los hipertensos deben tratarse con ARA 2 que vale 100 veces más cada pastilla que los inhibidores de la eca. Porque un país como nosotros tiene que montar a todo el mundo en un ARA 2 cuando el inhibidor de la eca es buenísimo, vale 30 pesos una pastilla. Y así tenemos innumerables ejemplos la presión tecnológica es brutal y eso va minando los recursos del sistema,

P 8: 1. Transcripcion Martha Lucia  ospina.docx - 8:9 [en atención en salud tenemos 1..]  (7:7)   (Super)
Códigos:	[modelo de salud actual] 
No memos

 en atención en salud tenemos 1, lo que te digo la expectativa desmesurada de la población por falta de información y manipulación. 2, la presión tecnológica, 3 sobre diagnóstico de sobre uso, los médicos algunos de ellos aceptados por la propia industria tienen un sobre uso, y unas cuotas de formulación y de solicitud de cosas lamentablemente, otros no son así, pero tienen una medicina defensiva porque con la judalizacion, ahora con la penalización del servicio médico sentencias muy graves de la corte  contra médicos que han actuado de la mejor manera que creían en ese momento hace que actúen en una medicina preventiva y que se aseguren y pidan muchas cosas que no se requerirían. 4 Cosa, la mala calidad de la educación medica, que hace que se haya desmejorado bastante y hace que haya poca clínica, mucho ejercicio médico y mucho doctor house, mucha solicitud de exámenes y ayuda y cantidad de cosas, que aumenta la carga del sistema, entonces todo eso hace que la tercera línea que es la atención se vea sobre cargada por un sobre uso y un sobrecosto que obviamente le quita oportunidad, a los que realmente lo necesitan. Entonces ese es el sistema y estamos en esas tres líneas, y yo que conservaría, todo el tema de seguridad sanitaria que el país ha desarrollado que es muy importante todo el tema que tiene que ver con lo que hace el invima y nosotros mismos, le metería plata a eso, creo que vale la pena. Yo conservaría del tema de aseguramiento, el modelo de tener una unidad de pago por capitación que sea representativa pero, la modificaría porque es poco predictiva la unidad de pago por capitación que tenemos y me matan cuando diga eso, tiene un R2  de 3%, una predictibilidad muy baja, porque tiene variables de predicción, ya hoy en día con toda la información que tenemos claramente ya debería estar acogiendo otro tipo de variables o de diagnóstico principal consumo de medicamentos, porque la a haría más predictiva pero el concepto en sí mismo de tener un valor de representación lo conservaría, es importantísimo por afiliado y conservaría las EPS pero las reduciría en número claramente, tendría que ser una sola no porque sería muy peligroso, pero debería ser unas 10, que tuvieran instalaciones más grandes con una unidad de pago mucho más predictiva y una evaluación basada en desempeño.

P 9: 2. Transcripcion Alex Duran_ok.docx - 9:6 [Dos una cosa que le hace mucho..]  (19:19)   (Super)
Códigos:	[modelo de salud actual] 
No memos

Dos una cosa que le hace mucho daño al sistema, es pensar que el sistema todo lo cubre, el pensar que tenemos un sistema de salud que cubre todo, es una amenaza en el corto y en el largo plazo. Entonces tú ves jueces legislando sobre el ideal, en Colombia vivimos pensando que vivimos en suiza, en noruega, entonces legislamos sobre el ideal, por ejemplo que la señora no tiene quien la cuide, entonces el estado tiene que entra a suplir que usted no pueda suplir su familiar, y eso hace mucho daño porque nosotros, hay un concepto básico en salud y es el tema del autocuidado, si bien usted tiene un derecho como persona a la salud, también tiene unas obligaciones, por ejemplo usted está obligado al autocuidado, no tiene que venirle alguien a decirle venga mínimo camine 30 minutos, eso es un tema más suyo. Eso nos hace daño, el pensar que el sistema todo lo resuelve y que estamos en un sistema ideal para poner 

P 9: 2. Transcripcion Alex Duran_ok.docx - 9:8 [este es un tema pensado, cuand..]  (27:27)   (Super)
Códigos:	[modelo de salud actual] 
No memos

 este es un tema pensado, cuando digo que el modelo de aseguramiento o de un administrador en el sistema esta, es porque soy consciente que en el sistema esta  sometido a unas imposiciones a nivel internacional y que en los próximos 20 años, Colombia no va a poder salir de ahí, porque se lo digo porque la OCDE hace como tres meses dijo mire evaluamos el sistema de salud colombiano y queremos decir que es uno de los mejores del mundo, entonces cuando uno dice, si la OCDE lo está diciendo eso, está ambientando el tema de sigan operando así, y es están avalando que las cosas funcionen así y el gobierno colombiano no va a hacer mayor tema contra es

P 9: 2. Transcripcion Alex Duran_ok.docx - 9:9 [Emssanar es una empresa solida..]  (31:31)   (Super)
Códigos:	[modelo de salud actual] 
No memos

Emssanar es una empresa solidaria, coosalud es una cooperativa, tiene modelos financieros incorporados acá, lo que pasa es que no tienen el letrero de banco pero funcionan tal cual. Por eso yo pienso que ahí hay que hacerle un ajuste al tema.

P 9: 2. Transcripcion Alex Duran_ok.docx - 9:12 [En lo público, una cosa que ha..]  (48:48)   (Super)
Códigos:	[modelo de salud actual] 
No memos

En lo público, una cosa que hace mucho daño que ahora siento que cada vez es menos es que el político tradicional veía el tema de salud como un fortín, en lo  burocrático y en lo financiero, para sus campañas y su crecimiento y sus cosas; en el valle lo dejo a su buen entender pero se han construido emporios desde lo Público desde el sector salud, porque el sector salud aguanta la crítica, y entonces prima lo político y lo burocrático y lo financiero

P 9: 2. Transcripcion Alex Duran_ok.docx - 9:13 [uno ve como por ejemplo alguie..]  (48:48)   (Super)
Códigos:	[modelo de salud actual] 
No memos

uno ve como por ejemplo alguien que no tenía precisamente lo que usted decía ahora, cero experiencia dirigiendo hospitales y entonces usted lo pone de un momento a otro dirigir un hospital complejo, se va a reventar porque este sistema ni yo que llevo 26 años en él, porque yo nací en el sistema, a veces no entiendo cosas, toca preguntar porque no entiendo tal cosa, entones dígame usted que este en un consultorio y yo lo saco a dirigir un hospital con una dinámica compleja, y de aquí a que usted aprenda como funciona el sistema se revienta y al político lo que le interesa es que esa persona sea de su resorte para poderla colocar ahí, entonces donde usted ve personas exitosas en el país, en donde ve una curva de aprendizaje y una curva de cúmulos de experiencias exitosas, entonces muy bien lo decía usted ahora, lo de Pasto no paso porque los últimos 2, 3 años eso es una suma de muchos años de trabajo y de proceso maduro que se ha respetado. Lo que pasa en Medellín lo mismo, lo que pasa con las empresas sociales en Cali,

P 9: 2. Transcripcion Alex Duran_ok.docx - 9:14 [Hay que hacer una claridad en ..]  (67:67)   (Super)
Códigos:	[modelo de salud actual] 
No memos

Hay que hacer una claridad en Cali, en Colombia es cierto eso, en Cali no, en Cali la primera causa de morir es la violencia, y volvemos al tema de autocuidado y como sociedad nos apersonemos de los temas y es que no existe ninguna tecnología, salvo nosotros como sociedad resolviendo un tema de salud publica que es la violencia. Hoy en día eso no lo resolvemos con ningún aparato, si no como sociedad, debemos ponernos de acuerdo y decir cómo vamos a enfrentar ese tema, la segunda causa de muerte en Cali es el tema de la hipertensión arterial, y al igual que el cáncer y eso también ya está demostrado que el 42% de lo que le pasa a uno en salud, tiene que ver con uno mismo tiene la explicación del tipo de sociedad que hemos construido, me explico hoy somos una ciudad americana, que no existe diferencia entre una ciudad americana y nosotros somos igual de sedentarios, somos consumidores de la misma chatarra que ellos consumen y de los mismos hábitos, es mas a veces más en algunos temas que acá, yo creo que en Colombia consumismos más Coca Cola que en Estados Unidos, nuestros niños ya son obesos, y eso que significa, de que entre mayor obesidad mayor riesgo de cáncer por ejemplo, cáncer de colon ,de seno en la mujeres, en la medida de que nos comportamos como los americanos de que nada nos importa, de que yo  y yo, el sexo no es seguro, cáncer de cérvix, el fumar eso lo aprendimos de las películas. Entonces para la respuesta concreta el perfil epidemiológico nuestro es producto de la sociedad que nosotros construimos, entonces hemos construido una sociedad americanizada, industrializada, todos queremos tener carro, todos queremos andar en moto mínimo y eso tiene sus implicacione

P 9: 2. Transcripcion Alex Duran_ok.docx - 9:15 [i lo considero por una razón s..]  (75:75)   (Super)
Códigos:	[modelo de salud actual] 
No memos

i lo considero por una razón sencilla, y es que digamos el MIAS no va a ser un modelo único para Colombia, precisamente el MIAS respeta digamos la particularidad de los territorios y es cada territorio el que tiene que construir su modelo y ahí un acierto del ministro en eso, y es que el MIAS de Antioquia y Valle va a ser muy diferente al de Bogotá, hoy como funcionamos, funcionamos desde Bogotá y todos los gerentes de Colombia terminan haciendo lo que Bogota dice, muy pocos se atreven a romper como bueno diciendo si hay unos derroteros, pero es que acá como le resolvemos a la gente de las zonas rurales, el modelo de aseguramiento en las zonas rurales en Cali no sirve. Y en el modelo de aseguramiento tú tienes población dispersa y entonces uno propone pero poca gente hace eso, en eso tienes razón, entonces digamos que el MIAS es una invitación para que el territorio construya su modelo de atención, aquí hemos empezado a botar corriente sobre el particular de lo que debe ser el modelo porque uno de los errores que no hemos comentado del sistema es que es un sistema que se basa en el hacer y hacer, a ti te pagan por actividad, por cuantos pacientes ves, pero eso está bien para un sistema de producción, pero esto no es una industria, esto es un sistema y el tema del servicio tiene que ser por resultados.

P10: 3. Transcripcion-Alexandra_Matallana_ok.docx - 10:4 [no, en este momento no.]  (46:46)   (Super)
Códigos:	[modelo de salud actual] 
No memos

 no, en este momento no.

P10: 3. Transcripcion-Alexandra_Matallana_ok.docx - 10:5 [bueno digamos que estamos en a..]  (50:50)   (Super)
Códigos:	[modelo de salud actual] 
No memos

bueno digamos que estamos en algunos cambios estructurales del sistema, precisamente estamos trabajando en el Modelo Integral de Atención de Salud- MIAS, y ese modelo integral MIAS está en pleno diseño y desarrollo y le apuesta a unas políticas de diferenciación de enfoque diferencial por regiones, incluso por departamentos  y que dentro del mismo departamento reconoce que hay diversidades y entonces lo que se está tratando es de construir el  MIAS desde la región, si eso llega a un feliz término y las rutas de atención, ósea si esto plantea hacer más rutas de atención por patologías cierto, entonces si esas rutas de atención las podemos construir por patologías así el perfil epidemiológico cambie , la cargue de enfermedad de Colombia cambie, si tenemos las rutas de atención y si funcionan las rutas de atención, eso sería algo importante, eso sería algo que garantiza que este 21% de esta población mayor a los 60 años puede ser atendida, si esto funciona, pero estamos hablando también de algo a futuro porque lo estamos, está recién salido entonces pues yo hablo como del sistema de salud ideal y poniendo mucho optimismo frente al MIAS, que realmente se construya desde la región, que realmente se construya sobre la carga de enfermedad de cada una de las regiones con enfoque diferencial de etnico, con enfoque diferencial cultural, si se hace muy juicioso nos va a permitir hacer un monitoreo de la población regional, tenemos estos niños pero tenemos estas personas mayores de 60 y que vamos a hacer con ellas, pero en estos momentos como estamos si nos quedaríamos como estamos,  no. Estoy hablando de que si ponemos en funcionamiento el MIAS, y lo ponemos a funcionar bien dependería mucho de eso. 

P10: 3. Transcripcion-Alexandra_Matallana_ok.docx - 10:6 [pues pienso que si ha sido un ..]  (62:62)   (Super)
Códigos:	[modelo de salud actual] 
No memos

 pues pienso que si ha sido un modelo de aseguramiento que ha sido exitoso, más exitoso en el aseguramiento, en las coberturas de aseguramiento, porque ya a pasar del aseguramiento al acceso de los servicios de salud hay una brecha muy grande,  que tenemos que cerrar con algo,  digamos que la expectativa es cerrarlos con las MIAS

P10: 3. Transcripcion-Alexandra_Matallana_ok.docx - 10:7 [No en ningún momento, no porqu..]  (66:66)   (Super)
Códigos:	[modelo de salud actual] 
No memos

No en ningún momento, no porque tenemos población indígena, digamos esa brecha cultural yo creo que, no creo que tengamos el 100% de la cobertura de la población colombiana asegurada, no creería, yo creería que podamos llegar al 98 al 99.

P10: 3. Transcripcion-Alexandra_Matallana_ok.docx - 10:16 [ósea lo que te dije, como qued..]  (170:170)   (Super)
Códigos:	[modelo de salud actual] 
No memos

ósea lo que te dije, como quedan escritas las cosas pues… pero a mí lo que me cuestiona es que la brecha que existe entre lo que se escribe y lo que realmente pasa abajo eso es lo que debemos mirar, no tanto lo que se escribe porque lo que se escribe no se está cumpliendo. Entonces lo preocupante es esa brecha que hay entre lo que se escribe y lo que realmente sucede cuando tú vas, y no tiene que salir lejos, aquí en Bogotá, pero cuando vas a los territorios ahí sí que los ves, la brecha que hay entre lo escrito y lo que realmente el colombiano tiene de salud, y el acceso que tiene a la salud, el acceso es una cosa inhumana, totalmente inhumana. Entonces pueden escribir todo lo que usted quiera, pero si realmente esa brecha nunca se cierra… yo creo eso yo creo que si se puede escribir mucho pero si no hacemos algo para que eso que está escrito se cumpla, difícil.

P10: 3. Transcripcion-Alexandra_Matallana_ok.docx - 10:17 [Bueno digamos que el flujo fin..]  (173:173)   (Super)
Códigos:	[modelo de salud actual] 
No memos

Bueno digamos que el flujo financiero del sistema, es que hay mucha plata que dentro de ese flujo que se escapa y no queda en salud. Y realmente que con este cambio que estamos trabajando que se dé con el MIAS con las rutas de atención. Si  estas rutas de atención puedan bajar al ciudadano del común y estos se apropian de ellas, podrán transitar de una manera fluida por el sistema; porque lo que pasa es que en este momento no les permite transitar, lo que hace el sistema es que la persona va a acceder al sistema y lo primero que hace es que encuentra todas las barreras para que no acceda, el tema es el no acceso, parece ser, como si esta personas que trabajan con el dinero de la salud de Colombia trabajaran por el no acceso, es el no acceso a la salud. Tengo una persona muy cercana a mí que es diabético que tiene enfermedad crónica, parece que tiene falla renal, necesitaba cita con el urólogo, el de urgencias le dijo vaya a una cita prioritaria y fue hace 15 días y la cita prioritaria con el urólogo es el 23 de junio. ¿Tú crees que un paciente sistémicamente comprometido se va a aguantar hasta el 23 de junio? Claro le toco pagar particular y le pusieron una sonda y ahora esta con una sonda. Pero el sistema no le dio respuesta por su enfermedad, lo único que hicieron fue ponerle una barrera. Entonces es duro.


P10: 3. Transcripcion-Alexandra_Matallana_ok.docx - 10:19 [Hum, pues digamos que lo que y..]  (181:181)   (Super)
Códigos:	[modelo de salud actual] 
No memos

Hum, pues digamos que lo que yo te digo a mi me cuestiona mucho entre lo que está escrito y lo que realmente sucede … porque es que si tu lees la ley tú dices huy el estado Colombiano defiende el derecho a la salud, pero baja a la realidad al mundo real al día a día y la ley lo dice lo promulga, pero no es así,  entonces lo que te digo nuevamente,  lo reitero, el reto está en poder cerrar esa brecha entre lo que está escrito, porque lo importante no es hacer normas de leyes porque eso si somos expertos en hacer decretos, leyes, léete tú los últimos 2 años, has un mapa normativo del sistema, es una cosa loca y entonces ya tu habla de una resolución y te hablan de una que ya derogo la anterior de la anterior, si yo creo que somos un país lleno de normas y la mitad no las cumplimos.


P10: 3. Transcripcion-Alexandra_Matallana_ok.docx - 10:23 [y al final todos los colombian..]  (209:209)   (Super)
Códigos:	[modelo de salud actual] 
No memos

 y al final todos los colombianos terminamos pagando todos los estudios que se hacen, entonces de pronto si sería algo importante tenemos el perfil epidemiológico de ahora y si podríamos hacer una prospectiva, una visión del futuro de donde queremos llegar pero realmente lo más importantes es bajar todas esas tasas que tenemos. 

P11: 4. Transcripcion Armando Gonzales_ok.docx - 11:13 [Yo creo que eso va muy asociad..]  (85:85)   (Super)
Códigos:	[modelo de salud actual] 
No memos

Yo creo que eso va muy asociado al desarrollo mismo del país, pero mira que esas causas de mortalidad son las frecuentes en un país desarrollado, o se mueren en el primer año y después de ahí disminuyen dramáticamente, o se mueren después de los 40, y a que se debe a muchísimos factores, el principal factor es el estrés, que es lo que más enferma. Los 5 factores de riesgo más importante son primero el estrés, segundo el cigarrillo, tercero diabetes, cuarto es hiperlipidemias, y quinto es obesidad. Entonces esos son los cinco factores más importantes de riesgo y el primero que se llama estrés, el principal factor de riesgo en salud y el estrés contribuye que las otras se vuelvan mucho más graves porque el estrés daña la salud mental y eso enferma a las personas son teorías que están influyendo muchísimo, entonces en un país como el nuestro donde hay tanta inequidad tanta desigualdad, el estrés en un país como el nuestro es fuerte. 

P11: 4. Transcripcion Armando Gonzales_ok.docx - 11:14 [A la desigualdad a la falta de..]  (89:89)   (Super)
Códigos:	[modelo de salud actual] 
No memos

A la desigualdad a la falta de recursos, a la falta de acceso a los servicios, no tenemos agua potable en un porcentaje de la población. Entonces todos esos factores, hacen que todo se vuelva una cadena y donde uno puede vislumbrar las soluciones, no las ve como fáciles entonces está condenado a convivir con esta situación que tenemos. 

P12: 5.Transcripcion Carlos Fajardo_ok.docx - 12:11 [Se va quedando porque no tiene..]  (46:46)   (Super)
Códigos:	[modelo de salud actual] 
No memos

Se va quedando porque no tiene el afán de actualizarse,  de innovarse si no que se ha ido quedando desactualizado tecnológicamente. Hoy quienes tienen los tratamientos de punta son las IPS privadas, la privada es quien hace inversiones que son realmente importantes para este tipo de tratamientos. Aunque hay excepciones como el caso de  Nariño, donde el Hospital departamental, trajo un equipo de última tecnología que le permite tener un mayor acceso a muchos de esos pacientes que se enviaban antes a Imbanaco, valle del Lili, están siendo ya tratados porque el hospital opto por esto, el de Medellín también.


P13: 6 y 7.Transcripcion Diego Gomes y Gonzalo Gonzales_ok.docx - 13:5 [Yo creo que hay un tema centra..]  (13:13)   (Super)
Códigos:	[modelo de salud actual] 
No memos

Yo creo que hay un tema central en todo el sistema y es que el sistema tiene agentes, que se están relacionando para prestar el servicio a una persona que lo necesita, y los agentes entre si no tienen confianza, la persona que busca servicios piensa que el prestador está tratando de evadir el servicio porque no tiene capacidad, el prestador piensa que el paciente está utilizando mal el servicio y que la EPS en un momento dado le está diciendo cosas que no es en costos, en fin. Y en el fondo hay un problema de confianza me parece que lo primero que hay que hacer es estructurar un servicio con una buena información que sea transparente para todos los agentes y que haya una relación de confianza entre ellos. Yo diría otra cosa, es indudable que el sistema de salud se volvió un botín político y por ahí se han sacado muchos fondos y se han elegido muchos políticos

P13: 6 y 7.Transcripcion Diego Gomes y Gonzalo Gonzales_ok.docx - 13:6 [El modelo del ordenamiento ent..]  (15:15)   (Super)
Códigos:	[modelo de salud actual] 
No memos

El modelo del ordenamiento entre todos los agentes, se debe pasar a un modelo de racionamiento donde se construyan contratos de beneficios común y en beneficio de la salud, y en beneficio del paciente, actualmente el modelo de relacionamiento es un modelo que gana o pierde en el cual el primer afectado es el paciente, el segundo es la viabilidad general del sistema, el tercer gran perdedor es la EPS  y el cuarto gran perdedor son las IPS. Ese modelo de relacionamiento se tiene que cambiar radicalmente. 

P13: 6 y 7.Transcripcion Diego Gomes y Gonzalo Gonzales_ok.docx - 13:28 [Pero todo eso hace que haya un..]  (155:155)   (Super)
Códigos:	[modelo de salud actual] 
No memos

Pero todo eso hace que haya un diagnostico erróneo y simplista,  y es decir que la salud tuvo un problema fundamental que es la corrupción, no, el problema fundamental de la salud ha sido el esquema de relacionamiento las EPS con las IPS.

P13: 6 y 7.Transcripcion Diego Gomes y Gonzalo Gonzales_ok.docx - 13:29 [Y usted sabe que el 60 el 70% ..]  (163:163)   (Super)
Códigos:	[modelo de salud actual] 
No memos

Y usted sabe que el 60 el 70% del costo de la salud son honorarios, y usted sabe que los profesionales de la salud son el grupo de mayor ingreso en Colombia y todavía dicen que no es suficiente

P14: 8. Transcripcion Jorge E. Robledo_ok.docx - 14:5 [Ellos en cobertura formal podr..]  (15:15)   (Super)
Códigos:	[modelo de salud actual] [modelo de salud futuro] 
No memos

Ellos en cobertura formal podrán mantener el indicador muy alto porque además esto es un régimen en el que estamos hoy montados sobre la publicidad y sobre el engaño. Los carnet son baratos de entregar, el problema es que todos sabemos las barreras de acceso, entonces usted tiene carnet y él lo protege de todo pero inclusive ya empieza a haber problemas de acceso en las prepagadas, esto se está degenerando rápido más todavía, entonces lo que va a pasar más adelante, el punto es que las barreras de acceso van a hacer cada vez mayores, porque pues no puede ser de otra manera porque las medidas que deberían tomarse, como por ejemplo acabar con la intermediación de las EPS, esa esta claro que no la toman bajo ninguna consideración, eso lo tiene claro el Banco Mundial, toda la banda neoliberal de la cual hace parte Alejandro Gaviria (Ministro de Salud)  entonces no hay otra manera de equilibrar un sistema desequilibrado que  es reduciendo los servicios a la gente. Lo ha dicho Gaviria en cierto sentido en todos los tonos él dice que el POS es demasiado costoso, todos los que son los derechos superan la capacidad financiera del sistema, es su gran teoría lo que nunca explica es que si eso es así, porque se les permite las ganancias a las EPS y porque se le permiten todos los fraudes a las EPS, porque si falta plata lo menos seria pedirle la coherencia de reducir los costos y unos de los costos principales de este asunto son las ganancias, que son distinta a los costos de administración 

P14: 8. Transcripcion Jorge E. Robledo_ok.docx - 14:7 [Cuando yo hice el primer debat..]  (23:23)   (Super)
Códigos:	[modelo de salud actual] 
No memos

 Cuando yo hice el primer debate grande de salud, que fue contra saludcoop por fraudes y por cosas, en esos días había una ola muy grande contra las EPS, yo dije una frase que estaba resultando cierta, yo me temo que la política oficial se va a convertir en sacar a los vivos poderosos para reemplazarlos por otros más vivos y más poderosos, y eso es lo que se está cumpliendo, sacaron a Palacino y van a entregarle eso a quien sabe que magnate de verdad, seguramente extranjero porque es evidente que la orientación no solo en salud, sino en todo es que todo el aparato productivo fundamental del País quede en manos de las transnacionales. 

P14: 8. Transcripcion Jorge E. Robledo_ok.docx - 14:8 [yo siempre he dicho que yo me ..]  (25:25)   (Super)
Códigos:	[modelo de salud actual] 
No memos

yo siempre he dicho que yo me imaginaba que el gran poder económico cuando la crisis de saludcoop, hablaban entre ellos y decían "oye y ese Palacino a qué hora se nos tomó esta vaina"

P14: 8. Transcripcion Jorge E. Robledo_ok.docx - 14:9 [Yo creo que esta evidentemente..]  (27:27)   (Super)
Códigos:	[modelo de salud actual] 
No memos

Yo creo que esta evidentemente amenazada, primero porque el sistema se modelo con unos supuestos que nunca se cumplieron, acuérdese que los supuestos con los que se diseñó la ley 100 era que hasta tal fecha iban a haber unos niveles de pleno empleo y la informalidad casi no iba a existir, ellos montaron una fantasía en los números para decir que ese sistema había funcionado, en su propia lógica de aseguramiento no se cumplieron los supuestos fundamentales que siempre son al final en este tipo de sistema, supuestos de empleo y de salarios son determinantes que además generan el problema del subsidiado, dependiendo de eso así de grande es el subsidiado que se come una gran parte de los recursos públicos, eso fallo desde el primer día. Pero además de eso todo lo demás que sabemos, hace que haya, un déficit grave, muy signado por la corrupción, acá hay un problema de corrupción de proporciones mayúsculas que ha invadido todo el sistema y de derroches y las transnacionales de los medicamentos, esto es como una gavilla contra ese pobre sistema y nadie defiende el sistema, que es una de las cosas graves de haber convertido al cartel de las EPS haberlos convertidos en defecto como en el ministerio de salud, es que aquí nadie defiende el sistema. Este es un sistema que no tiene auditoria publica, es un sistema donde la UPC se la inventan estos tipos como se les da la gana, en el primer debate grande mío pudimos demostrar como saludcoop inflaba la UPC, usted sabe lo que significa en plata inflar una UPC eso con el paso de los años lo que significa en robos al sistema, ahora eso está como una coladera porque uno tampoco puede dejarse llevar de la impresión, ello tienen mil mecanismos para ordeñarlo y que las utilidades se vayan a otro sitios no es que no estén, están ocultas de mil maneras contables, entonces la situación es gravísima y yo creo que esto va a terminar colapsando, yo creo que va a llegar un momento en que literalmente el sistema se va a desfondar, el problema de que no atiendan se va a volver tan masivo que esto va a ser una especie de conmoción. Pero tengo entendido que en cierto sentido quieren dejar que las cosas vayan hasta el colapso total para en medio de ese caos, imponer cualquier cosa como solución, como dejar el sistema de aseguramiento de las EPS, seguramente concentrando más como propone Gaviria que las EPS sean más grandes, de las transnacionales, mantener o subir los aportes y bajar el POS, esa es la propuesta neoliberal de equilibrio de ver como se suben los ingresos ver como se mantienen las ganancias de las EPS, y como se bajan los derechos y quedo nivelado, y golpear la tutela y todas las cosas que les desfinancien el negocio. 

P14: 8. Transcripcion Jorge E. Robledo_ok.docx - 14:11 [Claro, porque la ganancia sale..]  (35:35)   (Super)
Códigos:	[modelo de salud actual] [modelo de salud futuro] 
No memos

Claro, porque la ganancia sale de la salud que se niega, es así de simple si usted mira la UPC, la ganancia no está formalmente dentro de la UPC, pero de ahí tiene que salir eso es una cosa muy torcida y nace con una lógica muy corrupta, porque la ganancia solo puede salir de la medicina que usted niegue, porque los costos de administración están incluidos en las cuentas pero la ganancia no, del derecho que se niegue. Se supone que la UPC está bien calculada, si eso estaría perfecto no habría de donde salir ganancia, solo negándole a alguien el servicio

P14: 8. Transcripcion Jorge E. Robledo_ok.docx - 14:12 [EL punto es este, cualquier si..]  (37:37)   (Super)
Códigos:	[modelo de salud actual] [modelo de salud futuro] 
No memos

EL punto es este, cualquier sistema de salud del mundo necesita gastar plata en cuatro cosas, medicinas, profesionales de la salud y  trabajadores, procedimientos quirúrgicos,  hospitalización  y administración. Usted no puede tener un sistema sin gerentes sin contabilistas. Estos cuatro costos son inexorables en cualquier sistema de salud del mundo, ¿Cuál es el problema con el sistema de aseguramiento? Es que se meten un quinto costo, que es la ganancia de las EPS que es distinto al costo de la administración, Palacino nos costaba un montón de plata como gerente de la EPS pero yo diría que se la pagamos, pero además el tipo hacia ganancias con eso, además de que asaltaba el sistema y el sistema esta modelado para esa ganancia lo que pasa es que esto es una cosa con un diseño fraudulento que ni siquiera eso lo deja claro desde el principio, es más la corte constitucional tiene una sentencia en la que le exige al gobierno determinar cuál es la plata de la administración porque ellos ya ante el acoso de este debate dicen no es que la ganancia sale de la administración para no insinuar que sale de quitar salud, entonces la corte constitucional le dice a Gaviria usted me tiene que determinar cuánto es lo que tiene que ser ganancia y eso nunca lo determino. Y el fraude grande de todos estos tipos que lo han seguido haciendo todos estos años los interventores de salud es robarse la plata mediante el truco y eso se los oriento Montealegre cuando fue abogado de salud, que es inventarse el cuento que de la UPC también se paga la construcción de clínica la compra de ambulancias, y se compran salas de cirugía pero resulta que eso no está dentro de la UPC y eso pasa directamente al capital de la EPS, porque aquí hay una cosa que es monstruosa, los recursos de salud son parafiscales que el ciudadano paga y se vuelven públicos pero con una destinación específica parecido a como el primer fondo que hubo en Colombia el fondo nacional del café, pero en el caso del fondo nacional del café que lo administran la federación de cafeteros y la federación cobra por esa administración tuvieron la delicadeza de separar las cuentas del fondo nacional del café y las cuentas de la federación de cafetera y sin embargo ha habido fraudes y esas cosas. Esto les han permitido mantener un revoltijos de plata y nadie sabe de donde es una cosa y la otra, pero todo eso es con este propósito de la ganancia, y no tuvieron la delicadeza de hacer eso separando unas cosas de otras, y fijando unos límites específicos sino que le dieron el permiso de manejar esa vaina como se les da la gana y con eso estamos arruinados pero sus patrimonios son inmensos, están arruinadas algunas pero usted va a ver esa gente que empezó esos negocios con la cedula y que hoy tienen como dice un amigo mío, edificios blancos de letras doradas, esos tienen unos patrimonios inmensos que han salido del negocio. En este mundo de trucos contables y en el caso de algunas instituciones que son de tipo cooperativo apareció una especie de crimen que es el gerente de la EPS crea las empresas que roban a las EPS, pero esas empresas si son personales. 

P14: 8. Transcripcion Jorge E. Robledo_ok.docx - 14:13 [Esa es la ley estatutaria, esa..]  (42:42)   (Super)
Códigos:	[modelo de salud actual] 
No memos

Esa es la ley estatutaria, esa es una ley calculada para el engaño. Esta ley tiene una historia que vale la pena contar, la idea inicial de Gaviria era que solo se presentaba la ley general no la estatutaria, y la lucha de los medios y la gente, genero una serie de presiones por las que a Gaviria le toco aceptar tramitar las dos leyes, al final la segunda general no se tramito, pero la estatutaria si y Gaviria descubrió de manera consciente y unos cuantos oportunista que le jalaron a eso, que el problema era como meter dentro de la ley algunos reclamos como una especie de fraude legislativo, que es meter unas cosas que al final no están diseñadas para cumplirse por ejemplo en esa ley que es una ley estatutaria se metió a la salud como un derecho fundamental. Simultáneamente el gobierno había hundido una reforma constitucional que decía que la salud era un derecho fundamental porque acuérdese que en Colombia la salud es un derecho fundamental no por el gobierno, sino por los fallos de la sentencia de la corte constitucional esa fue una ley muy diseñada para la trampa, una ley gatopardista una frase que yo uso mucho, el gatopardismo tiene que ver con una novela italiana de principios de siglo que se llama el gatopardo en la que hay una conversación entre un par de tipos de la elite Italiana que estan preocupados porque se les están saliendo las vainas de las manos y el pueblo esta alborotado, entonces en la conversación consiste en que alguno dice que aquí hay que cambiarlo todo para que todo siga igual,  y ese es un poco el juego de Gaviria con esa ley, introducir algunos cambios que iban a dejar la cosas iguales o empeorarlas pero con frases como el derecho fundamental a la salud, que fue lo que paso, que la corte constitucional le movió dos o tres cosas a esa ley y por eso es que quieren arrodillar a la justicia, entonces esta pelotera que hay en ese momento con la ley de equilibrio de poderes es porque no han podido arrodillar del todo a la justicia y eso lo han disfrazado de moralismo y de honradez, pero el lio que ha tenido el gobierno es que no han podido arrodillar del todo a la justicia, entonces la corte se le atraviesa y esa es la piedra de Gaviria, y dice es que la corte quiere que los colombianos coman langosta, es la frase que usa Gaviria y se mete por el camino para casi que no aprobar la ley, de no sancionarla y se acuerda que yo tuve una pelea grande diciendo que sancionen esa vaina, entones esto no se entiende bien sin este contexto que yo le estoy dando, Gaviria intento esa ley que estaba pensada para golpear la tutela. Pero los jueces le cambian tres cositas y queda jodido, ese es el secreto de eso

P14: 8. Transcripcion Jorge E. Robledo_ok.docx - 14:14 [Claro, es que el lio que han t..]  (44:44)   (Super)
Códigos:	[modelo de salud actual] 
No memos

Claro, es que el lio que han tenido en ese artículo que le voy a dar usted entenderá eso, la constitución del 91 es una constitución que tiene unos derechos, pero es por sobretodo la constitución del neoliberalismo del libre comercio, es la constitución de la ley 100, la ley 142 de los servicios públicos, de la ley 30 al golpe a las finanzas de las universidades públicas. Pero también es la constitución de la tutela y otros derechos, entonces desde que se aprueba la ley que es una constitución neoliberal, ha habido un forcejeo en Colombia quitándole a la constitución sus elementos democráticos y volviéndola cada vez más neoliberal y eso tiene que ver mucho con la salud, por eso en parte se aprueba la ley de sostenibilidad fiscal, y si usted mira se aprueba el cambio constitucional y se introduce el criterio de sostenibilidad fiscal que no es otra cosa que es impedir que los fallos de los jueces a favor de derechos sociales dañen las finanzas públicas, y si usted se fija en la ley de aplicación constitucional es clarísimo que se dirige contra la salud y contra los fallos de los jueces en salud y esa es la pelea en la que estamos, y usted se imagina la pelea con la corte constitucional. Santos no ha podido arrodillarla eso independiente si hay corruptos o no hay corruptos eso lo hay en todas partes, para poder lograr este propósito que es el propósito por el fallo de los jueces son los que agrandaron al POS, ósea se les daño el negocio porque aumentaron los derechos y los derechos riñen con las ganancias, entonces si ellos quieren mantener tasas de ganancias altísimas siendo los ingresos prácticamente iguales tiene que reducir los derechos ese es el pleito. 

P14: 8. Transcripcion Jorge E. Robledo_ok.docx - 14:15 [Es que cuando usted pone la ga..]  (46:46)   (Super)
Códigos:	[modelo de salud actual] 
No memos

Es que cuando usted pone la ganancia al mando que es la frase de Friedman hay una y solo una responsabilidades sociales, las empresas que ganan plata pues usted supedita la salud a eso, entonces cosas que son obvias, entonces en salud siempre tendrá que haber curación pero es fundamental que haya prevención y eso lo hace cualquiera, es mas eso lo hace uno en la casa y eso porque el asunto de ellos no es la salud, el asunto de ellos es el negocio y ellos le ganan a todo, entonces cosas como permitirles a las EPS que intermedien que hagan ganancia con la atención primaria en salud, eso es el colmo eso es una cosa que cualquier red pública hospitalaria, inclusive privada puede manejar eso con toda la suficiencia sin necesidad de hacer una intermediación financiera, es que no dejan volar ni un centavo es que el criterio neoliberal es que el estado no puede gastar ni un centavo que no le produzca ganancia antes. Ese es uno de los secretos de la tercerización, cuando a mí me contrata la universidad como profesor directamente es una relación entre la universidad y Robledo y a mí me pagan mi sueldo, no es que exigen meter un tercero que cobre un peaje entre la universidad y yo y así todo, es privatizarlo todo entonces eso no es sorprendente que eso pase en este sentido porque es su naturaleza los arboles de papaya no dan aguacates, ellos no ven la salud como un servicio ciudadano si no como un negocio por el camino que vamos va a llegar el día y eso está empezando a entrar en que la justicia también se paga, y que usted tenga más o menos justicia dependiendo si tiene más o menos capacidad de pago que es un poco lo que al final está sucediendo en salud, si usted tiene harta plata usted consigue la salud que sea, ya la idea del derecho que presupone una igualdad no existe se ha ido perdiendo.

P14: 8. Transcripcion Jorge E. Robledo_ok.docx - 14:16 [Si lo conozco pero allí hay un..]  (48:48)   (Super)
Códigos:	[modelo de salud actual] 
No memos

Si lo conozco pero allí hay un punto que es muy sencillo, ahí está la EPS como el cuento de Garzón y la EPS ahí. Entonces así no funciona porque ellos están haciendo es otra vaina, primero es increíble que se gasten 25 años en descubrir que la atención básica es importante, es que esto está lleno de mala fe, entonces montan eso pero con la EPS ahí, en la práctica saboteando eso entonces aquí empieza a pasar una cosa que usted tiene que reflexionar del criterio neoliberal de legislación y de actuación hay muchas cosa que empiezan a aparecer en las leyes, por necesidades políticas o por presiones ciudadanas pero la política al mando, ósea el grupo que manda en Colombia que son los mismos no está de acuerdo con lo que dice la ley, y entonces la actitud es violarla es interpretarla de una manera mañosa es no ser coherente en relación con las cosas que están ahí, y entonces ese es el cuento de esa frase famosa "no es que esa ley no pego" es la manera de decir que yo no la cumplo y aquí hay una ley que dice que tiene que haber voto electrónico desde 2014 y no se les da la gana y no va a haber y punto, porque si hay voto electrónico pierden. Dice la constitución que hay que separar los poderes pero este escándalo que hay ahora y por eso se los tumbo la corte constitucional es que esa reforma del equilibrio de poderes rompía la separación de los poderes, y arrodillaba a la justicia al ejecutivo y lo mismo pasa con estas normas de salud entonces la norma puede decir muchas cosas, pero ellos no están en eso, ellos están en otra cosa por ejemplo, yo lo demostré en dos debates, que la superintendencia de salud y la procuraduría cuando estalla el escándalo de Saludcoop les dicen dos cosa, uno ustedes no pueden seguir pagando con la plata de la salud unos edificios y otras cosas que dejen de robarse la plata y devuelvan la que ya se robaron, eso es lo que dice los dos fallos, no roben mas y devuelvan los robado, bueno el gobierno interviene a saludcoop y los tipos siguieron robándose la plata en beneficio de saludcoop, no devolvieron lo que se habían robado y como muchos del robo era pagando créditos de edificios y de cosas siguieron amortizando los créditos, siguieron robándose la plata de la UPC para pagar los edificios que eso es robarse la plata. Pero eso lo hacia Palacino y lo siguieron haciendo estos tipos el estado Colombiano. Sobre eso si usted busca en internet hay dos artículos dos debates míos. Por ejemplo allí se está probando una ley de licoreras que tiene que ver con salud, entonces la constitución dice que el monopolio es rentístico las industrias licoreras pero eso no le gusta a los tipos, no van a cambiar la constitución sino que van a aprobar una ley tramposamente redactada para violar la constitución y acabar con el monopolio rentístico, de los departamentos. La ley de Zidre, la misma cosa la ley dice que las tierras baldías del estado Colombiano tienen que ser en exclusividad para los pobres del campo, eso dice la constitución y en vez de cambiar la constitución aprueban una ley redactada de manera mañosa para violar la constitución y meternos el cuento de que no la violan, esto en el degeneramiento nacional es así que es lo mismo que le hacen a los ciudadanos en las EPS, usted tiene derecho a este medicamento pero tiene que hacer 5 viajes, usted tiene derecho a especialista pero la cita se la dan en 5 meses, que ese es una manera de gobernar en Colombia profundamente corrupta, yo me acuerdo que en Manizales había un funcionario público que hacia lo que se le daba la gana y un día alguien le hizo un reclamo y el tipo le dijo no es que es mejor pedir perdón que pedir permiso, esa es la mentalidad del mando. 

P14: 8. Transcripcion Jorge E. Robledo_ok.docx - 14:17 [Porque es evidente que ninguna..]  (50:50)   (Super)
Códigos:	[modelo de salud actual] 
No memos

Porque es evidente que ninguna EPS y ninguna clínica ni nadie atiende igual a los del subsidiado que a los del contributivo, en la práctica todo el mundo sabe que los derechos de salud son desiguales y todo el mundo actúa así,  en Colombia hay un cierto regreso a las condiciones medievales que ni siquiera el país es consiente, por ejemplo eso de la estratificación, ser de estrato 1, 2, 3 es como en el medioevo el conde el marques todo eso, es una manera de jerarquía social y la gente no se da cuenta de esa vaina, pero eso lo que presupone es una discriminación bárbara en la práctica, entonces si usted llega a pedir empleo y usted es de un barrio de estrato 2 tiene menos posibilidad de conseguirlos que siendo de un barrio de estrato 4, aquí nos dicen que todos los títulos son iguales pero no es lo mismo un título de Ingeniero de los Andes que un Ingeniero de una universidad de garaje y aquí es lo mismo cuando usted llega a urgencias y usted pasa su carnet usted de inmediato queda discriminado. Yo estoy seguro que si yo soy senador me atienden distinto si soy un ascensorista. Todo un sistema de discriminación pero no es raro esto, pero además esta impuesto por el propio sistema porque si usted es una IPS y le pagan más cumplido los de las EPS del contributivo, pues usted empieza a discriminar por ahí. Y hay un factor que puede ser cultural pues la gente del subsidiado, es gente con niveles culturales menores, que se sienten menos poseedoras de un derecho que se somete más, bueno las barreras de acceso en ellos son muchos más duras, lo de Bogotá es una monstruosidad, la gente vive en ciudad Bolívar y las EPS están en las 180, es todo un sistema inicuo es una vergüenza esta vaina, entonces esto lo que hace es confirmar toda esta cosa que estamos diciendo que esto es una cosa monstruosa en el sentido estricto de la palabra, todo eso es una especie de conspiración contra los más débiles y eso que a esto le falto una cosa, si usted a esto le introduce las prepagadas esto le da otro giro el verraco que es donde estamos los que nos podemos dar el lujo de estar en prepagada y estamos en un sistema más o menos aceptable, ahora nos han dado duro en la cara a mí me acaban de montar en el pago por mi y mi mujer 1 millón de pesos mensuales porque pasamos de los 65 y ahora la política es sacar, claro porque ahora me vuelvo muy costoso y cuando cumpla 70 me meten fácilmente 2 millones, si llego a los 75 me sacan porque me sacan más fácil antes que me muera. 

P14: 8. Transcripcion Jorge E. Robledo_ok.docx - 14:18 [Es así, es más yo tengo esta t..]  (52:52)   (Super)
Códigos:	[modelo de salud actual] 
No memos

 Es así, es más yo tengo esta teoría doctor, creo que hay un grupo grande de Colombianos que por dignidad ya no van a esos hp servicios. Yo no voy prefiero vender todo lo que tengo para no tener que ir a una EPS a mí no me ponen a hacer esa cola, esa vaina no me lo aguanto por eso estoy en una prepagada y si tengo que dejar de comer para estar en una prepagada entones lo hago. Entonces averigüe usted esto alguien me ha dicho que es mucho el número de gente que se está muriendo en sus casas y yo tengo la idea de que hay un sentimiento de dignidad, es que los pobres son pobres pero no carecen de dignidad, entonces yo estoy seguro que hay gente que dice que yo me muero pero yo no les hago a estos hp una cola desde las 3 de la mañana y punto y si yo no pago pues me muero y se acabó esta joda, eso debe estar pasando estoy seguro, porque esto es una cosa ignominiosa. 

P15: 9. Transcripcion Julian Duran_ok.docx - 15:11 [El problema de la sostenibilid..]  (67:67)   (Super)
Códigos:	[modelo de salud actual] 
No memos

El problema de la sostenibilidad financiera tiene varias causas, primero una falta de auditoria o de revisoría al sistema como tal, al sistema no se lo está controlando suficientemente no se le está haciendo una auditoria sobre cómo es su manejo financiero, esta debería ser muchísimo más estricta, entonces no sabemos a ciencia cierta cómo se está administrando cuales son los objetivos que realmente están siguiendo los administradores de estas entidades. Segundo pues tiene que ver de pronto con hechos de corrupción o desvíos de recursos que están haciendo los administradores de estos sistemas como en el caso de saludcoop que se desviaron los recursos hacia otros proyectos y también hacia el pago de altísimos honorarios a los ejecutivos, y por el otro lado también tiene que ver con un problema de gestión de la administración de los servicios de salud que ellos prestan que de pronto no están direccionados hacia la prestación de un servicio con eficiencia y de calidad 

P15: 9. Transcripcion Julian Duran_ok.docx - 15:17 [Pues el derecho a la salud est..]  (102:102)   (Super)
Códigos:	[modelo de salud actual] 
No memos

Pues el derecho a la salud está definido como otro derecho en la constitución política, pero en la práctica no se cumple con el actuar de las EPS como le dije anteriormente, debido a que estas no están orientadas realmente hacia la prestación del servicio, hacia la satisfacción del usuario, si no que ellas tienen otros objetivos que es maximizar rentas y ganancias,  maximizar contrataciones o proyectos con una dinámica que nosotros no vemos, pero ellos que están allí si se dan cuenta de la posibles fuentes de ventas, en el sistema de salud. El derecho a la salaud No se cumple en la práctica 

P15: 9. Transcripcion Julian Duran_ok.docx - 15:18 [El principal obstáculo es que ..]  (106:106)   (Super)
Códigos:	[modelo de salud actual] 
No memos

El principal obstáculo es que tiene un sistema de salud donde ese objetivo no se cumple porque está conformado por un conjunto de entidades las EPS, que no están direccionadas hacia lograr ese objetivo que es cumplir el derecho a la salud de las personas, sino que están entidades buscan otras metas y otros objetivos relacionados con la búsqueda de rentas o de ganancias y no se deja prestar un servicio de calidad y de satisfacción, digamos que toda la institucionalidad que existe en torno a la salud en Colombia no favorece o no permite que no se alcance ese objetivo ese derecho a la salud. 

P15: 9. Transcripcion Julian Duran_ok.docx - 15:19 [Pues la verdad va a ser cada v..]  (110:110)   (Super)
Códigos:	[modelo de salud actual] 
No memos

Pues la verdad va a ser cada vez más difícil cumplir ese objetivo porque si ya en la actualidad hay problemas en el futuro esos problemas van a acrecentar porque ya vimos que el aumento de la población mayor de los 60 años, una insostenibilidad financiera en los estados contables de estas entidades de salud, entonces hacia el futuro ese sistema de salud va a tender hacia el colapso 

P15: 9. Transcripcion Julian Duran_ok.docx - 15:22 [Puede que lo publicite que lo ..]  (122:122)   (Super)
Códigos:	[modelo de salud actual] 
No memos

Puede que lo publicite que lo diga pero no lo aplica, o lo aplica en una escala muy pequeña no lo masifica, en 20 años de pronto se puede empezar a implementar pero muy lentamente demasiado lento para las dinámicas que están presentando ósea lo que habíamos dicho que la población tiende a envejecer que el sistema de salud se está quedando insostenible, está presentando perdidas insostenibilidad la gente se está yendo hacia el régimen subsidiado y entonces esas políticas de salud preventiva deberían masificarse rápidamente pero no lo vemos, no vemos que se esté haciendo rápidamente.

P15: 9. Transcripcion Julian Duran_ok.docx - 15:24 [Lo que usted menciona ahí es u..]  (130:130)   (Super)
Códigos:	[modelo de salud actual] 
No memos

Lo que usted menciona ahí es un problema de desajuste entre la oferta y la demanda en los ámbitos locales, en ciertas regiones o en ciertas ciudades, digamos la oferta de salud que ofrece tanto el régimen subsidiado contributivo, de pronto no satisface la demanda que se da en esa misma región o en esa misma localidad entonces tenemos un problema entre la oferta y la demanda, en contextos locales

P15: 9. Transcripcion Julian Duran_ok.docx - 15:25 [Si es muy posible que eso se s..]  (133:133)   (Super)
Códigos:	[modelo de salud actual] 
No memos

Si es muy posible que eso se siga profundizando porque si la población continua aumentando y continua aumentando la población que más servicios de salud solicita, y si el régimen contributivo y subsidiado no se expanden entonces ese desajuste entre la oferta y la demanda va a seguir creciendo entonces no se ve una política o una estrategia general para seguir aumentando esas ofertas locales de salud, que sean acordes con esa demandas locales, no se ve que se esté ejerciendo esas políticas.

P15: 9. Transcripcion Julian Duran_ok.docx - 15:26 [Eso se debe a problemas de ofe..]  (137:137)   (Super)
Códigos:	[modelo de salud actual] 
No memos

Eso se debe a problemas de ofertas a que el régimen subsidiado no le ofrece todo lo que necesitan las personas, pues esa brecha puede seguir aumentando y es lo más posible porque el gobierno nacional no se ve que este aumentando los recursos en las cantidades necesarios y suficientes para que se amplié esa oferta de salud subsidiada, entonces es muy posible que ese desajuste y esa brecha siga aumentando.


P15: 9. Transcripcion Julian Duran_ok.docx - 15:27 [Exacto y no vemos que se hacen..]  (141:141)   (Super)
Códigos:	[modelo de salud actual] 
No memos

Exacto y no vemos que se hacen hospitales públicos todos los días, lo más posible es que se cierren los hospitales públicos no que se creen nuevos hospitales públicos, no creo que esa demanda se va a igualar porque no se ve una política, ni la voluntad política para lograrlo

P15: 9. Transcripcion Julian Duran_ok.docx - 15:28 [La cuestión es que el sistema ..]  (145:145)   (Super)
Códigos:	[modelo de salud actual] 
No memos

La cuestión es que el sistema de salud en una economía es un sector económico muy importante porque genera una gran cantidad de empleos, genera una gran cantidad de encadenamientos productivos , vemos que hay productores de fármacos, servicios auxiliares a los servicios de salud, entonces es una gran cadena productiva y es un sector muy importante en la economía, pero en Colombia no se ha logrado consolidar un sistema de salud que este orientado hacia el logro de los objetivos y el cumplimiento a los derechos de la salud, vemos usuarios que permanentemente están insatisfechos, vemos que el sistema de salud no ofrece toda la cobertura que se necesita y en general hay una insatisfacción en el sistema de salud entonces lo grave es que no  hay una voluntad política o que se estén haciendo cosas actualmente, políticas que sean ambiciosas para cambiar todo ese panorama.


P16: 10.Transcripcion Angela Tascon.docx - 16:10 [Se me paso en las primeras pre..]  (38:38)   (Super)
Códigos:	[modelo de salud actual] 
No memos

 Se me paso en las primeras preguntas pero se es un vacío brutal, no hay confianza ni entre las EPS e IPS, y lo que es peor no hay confianza dentro de las misas IPS que formamos un mismo grupo entonces como no hay confianza entonces yo escondo mis cosas no comparto básicamente de lo que nosotros más hablamos es la parte de tarifa, yo tengo una tarifa pero jamás logro poderlas compartir, no hay ese clima de confianza porque como el recurso es tan escaso entonces que todo el mundo está a la expectativa de que puedo coger, esa es una debilidad trasversal en el sistema.

P16: 10.Transcripcion Angela Tascon.docx - 16:11 [Me parece que es un derecho ba..]  (46:46)   (Super)
Códigos:	[Calidad en la atención : sistema de Gestión de la Calidad] [modelo de salud actual] 
No memos

Me parece que es un derecho bandera y que dijéramos todo el norte tiene que ser ese, el norte del sector tiene que ser ese porque realmente ahí está la igualdad ahí está la solidaridad, ahí está la oportunidad, a mí eso como teórico es excelente pero ahí hay un término muy importante que es la calidad y que de verdad dijéramos el gobierno exige bastante al respecto pero no está el medio para que se dé entonces por eso hay tantas cosas que es lo que uno ve en los noticieros, tantos eventos adversos tantas cosas porque no es prioritario y no se eso en que parte quepa pero por ejemplo la calidad que no debería de ser un costo, más bien un beneficio la calidad vale mucho porque el sector o el gobierno, no facilita entonces el hecho de que usted este certificado acreditado, el gobierno trata de sobresaltar eso, no tiene mucho respaldo porque no hay leyes que favorezcan eso que es lo que muchas veces se ha solicitado y pretendido que haya un tratamiento de tipo de impuestos, o de tipo de exportación de insumos o algo, para estas entidades que logran estos escalones de excelencia y de calidad. Entonces pues lastimosamente no es congruente la exigencia con las facilidades que se dan, pero para mí ese decreto transversal al secto

P16: 10.Transcripcion Angela Tascon.docx - 16:26 [Nosotros somos una institución..]  (106:106)   (Super)
Códigos:	[modelo de salud actual] 
No memos

Nosotros somos una institución muy pequeña, dentro del sistema somos demasiado pequeños sin embargo que siento yo a nivel de nosotros, que cuando las cosas se hacen con transparencia, cuando se maximiza la eficiencia de los recursos se puede y que si cada quien tuviese esa voluntad de prestar un buen servicio eso no riñe con que haya un buen manejo financiero y que hayan unas utilidades, de modo que si se puede, hay instituciones que nos está mostrando que si se puede, así sea con recursos escasos. 


P18: 11. Transcripcion Fabian Mendez.docx - 18:14 [Yo creo que para nada al contr..]  (51:51)   (Super)
Códigos:	[modelo de salud actual] 
No memos

Yo creo que para nada al contrario lo que tenemos es una desconfianza profunda entre todos los actores, la red esta desmembrada, cada quien logre defender su pedacito, y yo no creo que la confianza sea en este momento un valor que prevalezca en el sistema, y yo creo que ese es una de las raíces del sistema.

P18: 11. Transcripcion Fabian Mendez.docx - 18:15 [Por ejemplo las listas de paci..]  (55:55)   (Super)
Códigos:	[modelo de salud actual] 
No memos

 Por ejemplo las listas de pacientes de cáncer, son vistas en estos momentos como un asunto protegido por las EPS porque saben que son fuentes de recursos y la relaciones entonces se vuelven de desconfianza, usted tiene que ver como defiende su negocio los pacientes con ligas de usuarios para defender por vía jurídica sus derechos las IPS resguardadas en mecanismo jurídicos para atender y disminuir costos a como dé lugar, los profesionales de la salud con vinculaciones y con contratos y condiciones laborales, donde tampoco hay elementos de confianza, mas yo veía el otro día un indicador que usa una EPS para sus médicos generales es, el porcentaje de remisión a especialistas y son mecanismos punitivos más que de confianza en la capacidad resolutiva de sus médicos, es ver como restringen más con elementos económicos o fundamentas en elementos económicos, la posibilidad de que ese medico vaya a otro nivel a tratar de buscarle la solución a un paciente, todos los médicos generales tienen ahora un indicador que se les está revisando, y ellos no pueden remitir a especialistas a un número determinado de pacientes que le llegan a su consulta, pero más que fundamentado en un principio de eficiencia y en un principio de capacidad resolutiva, es fundamentado en un principio económico. Entonces yo diría que no la confianza se refleja en los mecanismos que tenemos que hoy prevalecen en un sistema donde las tutelas son en su mayor medida por asuntos del post, porque si existe un post un plan que cubre, porque las tutelas tendrán que ser por asuntos que están incluidos en el post. Entonces estamos en un asunto de desconfianza donde la gente, cada uno de los actores trata de defender su pedazo, de establecer barreras, de establecer mecanismos para defenderse jurídicamente, pero no está fundamentado en lograr mantener el derecho a la salud de la gente.

P18: 11. Transcripcion Fabian Mendez.docx - 18:16 [en un modelo como el actual no..]  (71:71)   (Super)
Códigos:	[modelo de salud actual] 
No memos

en un modelo como el actual no creo, y de hecho Santos mismo lo ha dicho en espacios y el ministro, como lo están planteando y lo que el derecho dice no es viable en un modelo como el actual, no todo lo que se están imaginando es inalcanzable. 

P18: 11. Transcripcion Fabian Mendez.docx - 18:20 [Pues usted ha mencionado varia..]  (87:87)   (Super)
Códigos:	[modelo de salud actual] 
No memos

Pues usted ha mencionado varias, hay barreras geográficas, económicas, cuando yo tengo mis papas que son usuarios del sistema y tienen un médico que es médico y eso les facilita a muchas cosas, pero de no ser por eso y mi papa siempre me dice hoy fui a consultar y solamente me podían atender por esto y para la otra consulta le tocaba pedir cita, hay barreras que impiden  tener una atención integral de la gente, somos registro dentro de un sistema que atender en un sistema de atención a una persona que es un número, peor no hay una visión integral de la salud. Entonces yo creo que las barreras salen y se inventan de múltiples formas con barreras administrativas, barreras para hacer cas por desgaste, barreras para acceder a los medicamentos, de hecho los que están ganados y los que están en el plan obligatorio, son negados porque la lógica es con esos recursos que se tiene por cada persona la EPS tiene que buscar la mayor eficiencia, y eso implica colocar barreras para no atención. y finalmente muchos prestadores terminan en esa cadena también, hablo de las IPS profesionales de la salud organizados tratando de funcionar en el sistema pero que saben que hoy atienden a una persona y que el resultado de esa atención si les va bien ,es pagado 6 meses después y eso es lo que uno ven en las EPS actuales, las EPS tratando de sobrevivir en un sistema que le colca barreras para la oportuna atención de le agente y para prestarles atención integral porque las EPS está siempre colocándoles cosas y barreas a la prestación, entonces finalmente también los prestadores terminan confabulados en un modelo en el cual tienen que ver como subsisten, porque en el mejor de los casos unos compañeros y colegas míos que trabajan en la IPS , y eso es tremendo cuando uno va y dice EPS tengo esta cuenta no nos han pagado 300 millones de pesos que nos deben desde hace 6 meses y las EPS llega a negociar y dicen les puedo pagar 30 millones y lo otro se lo doy definiendo y esto no se lo voy a pagar ya. Entonces hay una barrera para que la gente y quienes quieren hacer trabajo por lo menos en discapacidad, y ni siquiera es una IPS pequeñas, es una de las IPS más grandes de la ciudad para la atención de pacientes de ese tipo, y esta con esos problemas. Entonces hay barreras para los pacientes, para las IPS, barreras que se colocan en mucho paso del sistema para lograr la eficiencia económica. 

P19: 12. Transcripcion Fabio Osorio.docx - 19:6 [No, te digo la verdad yo creo ..]  (18:18)   (Super)
Códigos:	[modelo de salud actual] 
No memos

No, te digo la verdad yo creo que el gobierno se burla de los colombianos, no hacen sino sacar pañitos de agua tibia desde la ley 100 del 93, han sacado la 1122, leyes que de una u otra forma lo que hacen es dilatar la verdadera atención de fondo que es que haya un nuevo sistema de salud donde desaparezca el intermediario y se privilegie la atención, aquí en Colombia pueden sacar todos los programas que hayan en atención en salud, pero mientras en Colombia no se acabe la enfermedad que es la corrupción no va a poder llegar a ser efectiva en los colombianos uno lee en la 1122, en el decreto en salud, en el salud mental, en estas leyes lo que hacen es tapar la dificultad pero el problema de fondo sigue, siempre he dado un ejemplo es triste que acá hayan pacientes de primera categoría y de última categoría, dos ejemplos claros Juan Manuel Santos le dio cáncer lo vieron el lunes, lo atendieron el martes y lo operaron el viernes. Al vicepresidente Vargas lleras le dio un tumor en la cabeza y en menos de un mes ya tenía todo, acá hay pacientes en Colombia en mi HUV, que yo he atendido que llevan 2 3 años con un tratamiento y con una operación para que se los hagan oportunamente y con una quimioterapia cuando es cáncer, entonces no pueden haber próstatas de segunda, ni tumores de segunda, debe ser todo por igual independientemente del estrato social donde se encuentra el pacien

P22: 13. Transcripcion Maria Ines Pantoja.docx - 22:15 [Es que con el doctor Arias he ..]  (80:80)   (Super)
Códigos:	[modelo de salud actual] 
No memos

Es que con el doctor Arias he tenido muchísimas diferencias porque él defiende a las EPS a capa y espada y lo tiene que hacer porque por eso le pagan, pero el debería en un momento a otro dar la razón de que somos personas y no nos pueden tratar como artículos, y cuando ellos dicen que presto 50.000.000 de  citas, pero porque tantas, porque no han atendido a la gente con especialistas, porque no les han dado promoción y prevención antes, si a mí me da una enfermedad y me la atacan a tiempo, por ejemplo muchos pacientes que los pueden tratar a tiempo para que no llegue a ser crónico, ahí lo pueden sostener muchísimo tiempo y salen menos costoso para el sistema, pero como no lo atienden a tiempo llegan a crónicos y ahí si le cuesta un montón al sistema.

P22: 13. Transcripcion Maria Ines Pantoja.docx - 22:26 [Eso es un problema que el mini..]  (134:134)   (Super)
Códigos:	[modelo de salud actual] 
No memos

Eso es un problema que el ministro lo ve desde el punto de vista de la economía pero no lo ve desde el punto de vista del ser humano, ese es el problema.

P22: 13. Transcripcion Maria Ines Pantoja.docx - 22:27 [Es que es disintió si usted es..]  (138:138)   (Super)
Códigos:	[modelo de salud actual] 
No memos

 Es que es disintió si usted está en una empresa de productos de lo que sea, usted maneja diferente eso, pero usted está tratando seres humanos en el sistema de salud, no está frenteando ningún otro artículo y no nos puede manejar como clientes del sistema, pero uno al cliente lo cuida para que no se le vaya, acá no hay ese cuidado a las personas.

P22: 13. Transcripcion Maria Ines Pantoja.docx - 22:30 [La Organización Mundial de la ..]  (158:158)   (Super)
Códigos:	[modelo de salud actual] 
No memos

La Organización Mundial de la Salud dice que no solo los números y las estadísticas muestran la realidad que hay que acercarse a las personas para escuchar la realidad, pero la sola estadística no dice mucho porque más dice la vivencia de las personas. Al ministerio le mando un comunicado diciéndole eso porque le nos muestra cifras él dice de 47 millones de colombianos solo el 40% esta inconforme, y yo le digo señor ministro de los 40 millones de colombianos cuantos no se acercan a los servicios de salud, porque mucha gente no va

P22: 13. Transcripcion Maria Ines Pantoja.docx - 22:31 [Pero entonces condiciona a una..]  (166:166)   (Super)
Códigos:	[modelo de salud actual] 
No memos

Pero entonces condiciona a unas preguntas como le digo yo, por ejemplo ponen una palabra que no es usual para la gente, a una persona de un estrato 2 la llaman y le preguntan ella no sabe que es, entonces responde sí o no sin saber que están preguntando, y ellos no aceptan que bajarse el lenguaje a la población en general porque a usted le suena la pregunta muy infantil para usted que es una persona que conoce y que sabe, entonces esa gente no entendemos como podrá contestar la gente  porque son preguntas muy cerradas y que de acuerdo a eso van a clasificar las EPS póngase usted a pensar y las sanciones por ejemplo son, usted negó 20 servicios eso no significa para la superintendencia pero si usted negó 200 eso si significa, pero para nosotros significa como 200 como 20 o 1, a mi alguien me dijo usted porque pelea si usted está hecha, entonces le dije mire mientras haya una persona sufriendo en el sistema de salud yo no estaré hecha porque no pienso en mí, y pienso en la gente que sufre, y yo soy religiosa porque quiero a la gente y me gusta trabajar con la gente y yo puedo tener todo en el mundo pero en mi comunidad haya una sola persona sufriendo seguiremos luchando por esa persona, porque el ser humano vale, para ellos no vale. Y si vamos a los regímenes especiales eso es peor los regímenes especiales son barbaros, los que funcionan un poquito mejor son los de las universidades, por ejemplo la nacional tiene un muy buen servicio, pero lo que es maestros de escuelas eso es pésimo. Tengo un caso de una señora que le descubrieron un cáncer de páncreas en abril, después de mucha cosa le dieron una cita para el 23 de mayo, llego el 23 de mayo y el medico no estaba que hacia 2 semanas que no iban porque no le habían pagado, que la llamaban cuando el medico llegara, y es la hora que no la han llamado y ya es junio y tiene cáncer de páncreas. Y entonces no tienen sino un solo médico para atender este caso y no le dan a una persona una opción.


P22: 13. Transcripcion Maria Ines Pantoja.docx - 22:32 [Y la pobre señora y la hija su..]  (170:170)   (Super)
Códigos:	[modelo de salud actual] 
No memos

Y la pobre señora y la hija sufre, dice uno no puede ser, y ha habido muchísimos casos de los regímenes especiales como el de los maestros de la policía que están muy mal. Los del ejercito muy pocos, pero los de la policía en general nos tocó hacerle una tutela

P22: 13. Transcripcion Maria Ines Pantoja.docx - 22:33 [Al general Vera le toco hacer ..]  (173:173)   (Super)
Códigos:	[modelo de salud actual] 
No memos

Al general Vera le toco hacer tutela para que la EPS lo atendiera, para que le autorizaran un trasplante, lo que sí tienen ellos es que nos reintegraron un dinero que nosotros gastamos, con el general pero que otras EPS no reintegran absolutamente nada, pero nos tocó ponerle tutela. 

P23: 14. Transcripcion Mario Hernandez.docx - 23:14 [Pues haber yo he tenido una re..]  (62:66)   (Super)
Códigos:	[modelo de salud actual] [modelo de salud futuro] 
No memos

Pues haber yo he tenido una relación muy ambivalente con ese proceso de la ley estatutaria y así ha salido en los escritos que he hecho, porque si la corte constitucional hubiera sido más coherente con la jurisprudencia y con el bloque de institucionalidad, habría anulado, declarado inexequibles unas partes muy importantes de la ley estatutaria, primero porque la ley estatutaria en principio parte de la base que es el derecho fundamental a la atención en salud, no a la salud en un sentido amplio. Sin embargo la observación 14 del comité de derechos económicos, sociales y culturales de 2000, dice con toda claridad el derecho a la salud es el logro de mayor nivel de salud posible, en la población no solamente la atención a la enfermedad y entonces claro si asumimos eso seriamente, pues los componentes esenciales del derecho a la salud incluiría por lo menos esos 13 componentes que en la misma observación plantea y nosotros recogimos en el proyecto de ley que nosotros planteamos, es decir vida digna, libertaD y autonomía, incluso derechos sexuales, etc. en salud todo el tema de no discriminación, de morir dignamente, de no ser sometido a una serie de cosas, pero además agua potable, ambiente sano, educación suficiente, vivienda digna, trabajo digno, es decir una serie de condiciones para una vida saludable además de la atención a la enfermedad y la atención de la misma, y la participación en las decisiones individuales y colectivas, entonces eso que le presentamos a  congreso lo había podido revisar la corte diciendo lo podemos presentar a ella, si no que mire quedo demasiado limitado el derecho a la salud, y por otro lado cuando ya se empieza a definir que es de la atención a la salud que es lo que es derecho está claro que el artículo 15 dice, bienes y servicios, y tecnologías y dice con toda claridad y no serán cubiertos los servicios con recursos públicos que tengan estos seis criterios y la corte acepto eso y no discutió a fondo el tema de evidencia científica sobre seguridad eficacia y eficiencia y efectividad. Y entonces claro la corte no tiene por qué saberlo pero esa es la puerta de entrada para un gran dominio medico industrial para definir que entra y que no entra y el ministerio sigue muy confiado en que va a poder aplicar unas metodologías de cálculo de costo beneficio para tratar de excluir claramente lo que no sea de costo beneficio, pero no va a afectar y no está afectando lo que genera los altísimos costos que es lo que discutimos el día de hoy en la conferencia del doctor Holguín, es decir los derechos de propiedad intelectual que es realmente lo que esta generando estos sobrecostos enormes de los medicamentos y las tecnologías a través del sistema de derecho de propiedad intelectual. Entonces pues quedo limitado, el derecho de la salud quedo limitado en la ley estatutaria, ahora con las modulaciones que le hizo la corte al proyecto de ley que salió del congreso hay algunas posibilidades, por ejemplo el hecho de que no acepta la corte de que haya un plan explícito y además una zona de priorización y además un decreto de exclusiones, no la corte muy claramente dijo solo exclusiones y retiro todo lo demás. Entonces eso se nos devuelve a la sociedad Colombiana y al sistema diciendo ¿entonces es compatible lo del POS, un POS explicito? Pues no ya la ley estatutaria dijo que no, entonces como se sostiene eso, porque seguimos insistiendo en la relación UPC , POS, y eso hay que denunciarlo y hay que demostrarlo la incongruencia jurídico constitucional de esas dos cosas de la ley estatutaria y el modelo ley 100 con una  UPC y POS . Entonces por eso digo la ley estatutaria aun con sus limitaciones tiene una posibilidades que deberíamos explotar un poco más desde las sociedad civil porque no lo va a ser el gobierno, el gobierno cree que puede aplicar la ley estatutaria con la ley ordinaria que hay. 

PC: ¿Usted cuales cree que son esas posibilidades que tiene la ley?

MH: pues mire en principio esa idea de solo exclusiones, y además uno tendría que decir con un mecanismo participativo amplio con mucha discusión pública, pues también hay que exigirlo muy profundamente porque eso fue lo que dijo la corte. Segundo el tema de los obligaciones del estado y los elementos que debe garantizar el estado como componentes del derecho fundamental que lo dijo también hoy el doctor Holguín y que sale de la observacion 14, eso está en la ley estatutaria y todos los principios que ordenan como es que el estado debe garantizar eso, con integralidad que se le dejo todo un artículo, con oportunidad con afectación de los determinantes sociales de la salud con relación con los perfiles epidemiológicos y toda esa cosa, con eficacia pero sobre todo calidad, mucha calidad en la cosa oportunidad y calidad es lo que más se viola sistemáticamente en el sistema actual, entonces tomar esos principios y mostrar como el sistema actual no logra resolver por la vía en que está organizado, pues eso es una oportunidad. También el tema de la tutela, no tocar la tutela porque es el mecanismo fundamental para que proteger cualquier derecho, no dejarnos meter en el cuento en que es que ya la ley estatutaria dijo que era solo para lo que está incluido y no para lo que está excluido, cosas así. El tema de trabajo digno, hace mucho énfasis en el trabajo digno y obliga al gobierno a estabilizar y mejorar la calidad del trabajo en salud, el tema de medicamentos en toda la cadena de medicamentos debe haber una seria de políticas para disminuir el impacto en medicamentos, eso no se está haciendo realmente, el tema de las urgencias no se necesita autorización pero para toda la atención de urgencias, pero para eso también dice en la ley estatutaria debe haber una prioridad de la inversión en promoción y prevención y atención oportuna, es decir redes integradas que realmente respondan, entonces uno tendría que presionar a los actores actuales del sistema hacia esos ordenadores digamos de la ley estatutaria. El tema de la autonomía médica entendida como el criterio medico es el que debe prevalecer en las decisiones por encima del criterio financiero y administrativo eso esta suficientemente desarrollado por la sentencia C 313 del 2014, que es la sentencia de la corte que declaro la accesibilidad parcial, entonces hay muchas posibilidades ahí, y tendríamos que ponerlas a funcionar sistemáticamente, es lo que he estado impulsando en distintas organizaciones, el asunto está en cómo ordenar todo eso y ponerlo en una forma más organizada para que la sociedad colombiana y el estado colombianos se conmueva en el sentido de que ahí hay unas implicaciones muy fuertes de la ley. 

P23: 14. Transcripcion Mario Hernandez.docx - 23:19 [Porque creen en una cosa que s..]  (90:90)   (Super)
Códigos:	[modelo de salud actual] [modelo de salud futuro] 
No memos

Porque creen en una cosa que se llama economía de escala que pueda garantizar la rentabilidad de ese negocio, porque si no los tipos se les salen del negocio como lo han amenazado varias veces si uno oye los discursos del presidente de ACEMI dice no o me aumenta la UPC o me le salgo del negocio y le dejo sus afiliados y sus pacientes ahí y los atiende usted señor estado, es una amenaza permanente porque son ellos quienes dominan el asunto. Y por eso el gobierno dice no es que las EPS no están en discusión, dice si quitamos las EPS quien va a hacer eso, no hay institucionalidad para hacer eso. Ese es el problema gravísimo que tiene, con la destrucción de la institucionalidad pública solo quedamos en manos de la benditas EPS y son las reglas que ellas digan que nos van a obligar a hacer cosas.

P23: 14. Transcripcion Mario Hernandez.docx - 23:20 [Pues es que hay dos cosas, por..]  (94:94)   (Super)
Códigos:	[modelo de salud actual] [modelo de salud futuro] 
No memos

Pues es que hay dos cosas, por un lado se ha planteado es que los pobres no demandan mucho porque aprenden a sufrir cosas así, totalmente idealizada en cambio los que contribuyen y pagan mes a mes, tienden a pedir más. Eso es una lógica que viene hace rato los análisis de demanda de salud, el riesgo moral es que cuando yo estoy pagando todos los días quiero consumir eso que pague, en cambio si no pago no voy a consumir tanto de eso. Mire eso es una perspectiva demasiado individualista y demasiado ligada al egoísmo supuestamente natural de los modelos económicos, eso oculta lo que esta pasando realmente atrás y es que finalmente las formas como se han desarrollado esos dos regímenes hace que al pobre se le trate como a pobre, la información es pobre, la accesibilidad es pobre, las barreras de acceso cada vez son mayores y como el pobre tampoco reclama porque le reglan y si reclama de pronto se lo quitan, la lógica del subsidio de la calidad entonces va generando esta idea de que allá los pobres tienen menos y acceden menos, mientras que los del régimen contributivo se le tratan como medios pero todo el tiempo le están diciendo, sabe que si usted quiere mejorar la oportunidad la calidad, pague un poquito más, sigue siendo del régimen contributivo pero si paga una medicina prepagada lo atendemos adecuadamente. Y entonces alli se empieza a generar una demanda cada vez más frecuente más grande en el régimen contributivo, que en el régimen subsidiado, los de medicinas prepagadas son del régimen contributivo porque les obligan a pagar el régimen contributivo, pero usan más frecuentemente, entre más plata pongan más frecuentemente con mayor calidad con mayor oportunidad, con todo lo que usted quiera. Entonces por eso hay diferencias entre el régimen contributivo y el régimen subsidiado, el riesgo moral de la sobreutilización de cuando pago y la subutilización cuando no pago, que es donde nos han venido concentrando la explicación y yo creo que eso hay que estudiarlo con más profundidad. 


P23: 14. Transcripcion Mario Hernandez.docx - 23:21 [Claro que sí, esa es una buena..]  (98:98)   (Super)
Códigos:	[modelo de salud actual] 
No memos

Claro que sí, esa es una buena explicación, pero uno empieza a ver como la lógica esta de que el régimen subsidiado se atiende en la red pública y que el régimen contributivo entonces se atiende en la red privada pues resulta que está generando entonces unas cosas muy perversas, los pobres con ingresos muy bajos, pero que tienen un trabajito como celador o un trabajo como empleada doméstica, que entran al régimen contributivo aunque tengan su hospital ahí al frente el régimen contributivo no contrata con el hospital, y le está generando unas barreras adicionales con el sector privado, eso tampoco está bien estudiado. En Bogotá es impresionante, cada vez más el régimen contributivo es mucho más grande que el régimen subsidiado ya vamos en el 72% del régimen contributivo y en ciudad bolívar es como el 65% aunque allá están los pobres, porque como los obligan a vincularse al régimen contributivo con cualquier ingreso como independiente o como asalariado o con contratación flexible, informal de orden de servicios, en todo caso pagan pero los servicios no están allá, los obligan a irse hasta el norte tres y cuatro horas en buses y eso pues va desestimulando cada vez más la demanda y eso es régimen contributivo pero para pobres. 

P23: 14. Transcripcion Mario Hernandez.docx - 23:22 [Yo creo que en principio va a ..]  (102:102)   (Super)
Códigos:	[modelo de salud actual] [modelo de salud futuro] 
No memos

Yo creo que en principio va a continuar, es que finalmente al subsidiado se le trata y se le pone una cantidad de barreras que no creo que vayan a cambiar porque se supone que ahí hay menos plata, la UPC es un poco menor, no está ajustada a riesgos y eso lo han aprovechado bastante bien las EPS del régimen subsidiado, entonces no creo que estén presionando para que haya más demanda de servicios. Pero por otro lado también está el tema de la prevención de la promoción toda esta cosa, no creo que baje mucho la demanda y la utilización de servicios si sigue concentrado en esa perspectiva individualista tan fuerte, puede que logren las EPS bajar un poco la demanda del segundo y del tercer nivel, los tales servicios complementarios, como lo llaman con la estrategia de la medicina familiar que será el contenedor de ese ascenso, entonces es posible que baje la tasa de hospitalización que hoy es la más alta de américa latina, es posible que bajen algunas cositas por ejemplo, cesáreas pues si fuera coherente como dice la OMS debería ser el 15% máximo de los embarazos se complican, el resto debería ser parto normal y habría que estimular las condiciones y los medios y los recursos para que hayan partos normales, pero pues no, porque la plática se concentra en las cesáreas es mucho más fácil, la paga es mejor, las mismas señoras ya aprendieron a pedir eso como condición universal y pues eso tiene más riesgo para el niño, para la señora, para todo el mundo que un parto normal, pero bueno es la lógica del mercado la que impulsa eso. 


P24: 15. Transcripcion Martha Balbuena.docx - 24:3 [Si las cosas siguen así va a l..]  (14:14)   (Super)
Códigos:	[modelo de salud actual] 
No memos

Si las cosas siguen así va a llegar un momento en el que no hay salud y las IPS no podrían prestar salud. Colapsaría si sigue como esta, yo pienso eso porque vuelvo a lo mismo yo tuve un tiempo en el que trabajar con la secretaria de salud donde no existía la EPS no había un intervenirlo y pienso yo que en ese momento se hacía más cosas con el poco dinero que se tenía, en esa época a nosotros no nos quitaban la gente que estaba en el régimen contributivo, yo sé que era un ministerio o una nación paternalista, pero las personas que estamos como directores en esa época de las IPS  y que teníamos por ejemplo unos principios éticos, ese dinero que llego se utilizó en lo que debía utilizarse, que era esa parte de promoción en salud y diagnostico porque cuando llego ese dinero era para cubrir esas personas en salud pero si había otro problema quirúrgico que se pudiera hacer se hacía, y si no se remitía a otro nivel y allí igual se hacía pero no teníamos los intermediarios de ahora que son las EPS. El dinero del ministerio llegaba a las secretarias y a las decretarías llegaba a la IPS y nos hacían un control muy grande donde eso no tenía que fugarse para otras cuestiones que no fueran esa part

P26: 16. Transcripcion Senador Ospina.docx - 26:8 [En Colombia somos avanzados en..]  (34:34)   (Super)
Códigos:	[modelo de salud actual] [modelo de salud futuro] 
No memos

En Colombia somos avanzados en las leyes con dificultades de aplicación de la misma, la ley estatutaria en salud, y lo que tenemos nosotros en tema del derecho a alas salud como un derecho constitucional robusto y la manera como la corte y todos hemos protegido ese derecho, es de lo más avanzado que existe en Sudamérica y de lo más avanzad que existe en el mundo me parece a mí que se trata del punto a donde llegar y un punto de partida en temas de solides en principio del sistema, pero difícil de aplicar en la manera que tenemos diseñado el modelo

P28: 17. Transcripcion-Gustavo-Morales (1).docx - 28:11 [Bueno lo que tengo que decir d..]  (38:38)   (Super)
Códigos:	[modelo de salud actual] 
No memos

Bueno lo que tengo que decir de la circular 03 del 2013 que establece la metodología para el control directo de precios, es uno de los componentes de la política farmacéutica, mi primera respuesta a ala pregunta es que no se puede regular la política farmacéutica solo por ese punto porque hay 8. Entonces yo creo que un análisis de la política farmacéutica debe ver eso integralmente. Entonces con esas preliminares yo te diría que en un sistema de salud que principalmente depende de recursos públicos, por supuesto que debe haber algo como la circular 2013, que yo creo que está mal llamada como circular de precios porque en realidad si tú la lees con cuidado no es un control de precios de todo el mercado no te controlan lo que pagas en la droguería ni lo que el seguro privado le paga por un medicamento donde no hay recursos públicos. Es más bien el gran comprador de medicamentos que es el estado diciendo cuanto está dispuesto a pagar en el sistema público de salud que es el que paga. Entonces yo te diría que el mal llamado control de precios es inevitable en un sistema que depende de recursos públicos, y el circular de precios como lo está en la 03 es muy razonable, porque primero tiene unas reglas muy claras de que productos van a ser controlados y cuáles no, dependiendo de su papel en el mercado, y una vez acotado que productos van a controlar te dice que precios le vas a poner y la metodología es la referenciación internacional de precios que es el estándar mundial, porqué Colombia no puede compararse con estados unidos, ni con países europeos, pero tampoco se puede comparar con Camboya en el valor de los medicamentos. Entonces que exista un control de precio bajo la mecánica internacional es adecuado, no nos hemos opuesto a eso, el problema es que lo han aplicado muy mal la política se lanzó en 2013 y ese mismo año s de aplico, salió una lista de control de precios y les fregaron el precio y entonces hasta el diciembre de 2017 no se volvió a aplicar, no se usó la formula ni en 2014 ni en 2016. Y en el 2017 lo hicieron a las patadas tanto que lanzaron una circular para corregido los errores del 2017, lo que ha faltado es método gerencia y rigor, que es muy importante para la industria farmacéutica porque ellos hacen sus planes, con unos precios que esperan van a existir 	y no existen, entonces existen productos que están controlados que deberían estar controlados, productos que no deberían estar controlados pero que están controlados, productos que no se les ha actualizado el valor, hay un caos de implementación pero no de la polí

P28: 17. Transcripcion-Gustavo-Morales (1).docx - 28:13 [Esa es una preocupación que te..]  (46:46)   (Super)
Códigos:	[modelo de salud actual] [modelo de salud futuro] 
No memos

Esa es una preocupación que tenemos, lo que es bueno una filosofía de agregación donde por volumen se tiene a bajar el precio si eso se hace con transparencia con reglas claras y donde todos los posibles oferentes pueden ofertar, quien se va a oponer a eso. La duda que si tenemos en la implementación es si Colombia compra eficiente que es una entidad creada para comprar papeles y computadores y gasolina, tiene la experticia para manejar productos complejos como los medicamentos y la respuesta es que no. Si uno fuera a coger la política farmacéutica y dijera que está bien y que está mal la compra centralizada está bien, pero hay que tener cuidado con la implementación porque sobre todo cuando se trata de productos de alta complejidad, la sustibuilidad o lo que se llama la intercambiabilidad no es automática, tu puedes cambiar un computador Dell o Lenovo por un hp y no es mayor cosa lo que pasa, pero si cambias un producto de cáncer A por el producto B aunque sea el mismo principio activo puede haber riesgos, ese estudio previo es el que nos preocupa que no exista cuando Colombia compra eficiente se embarque en compras centralizadas.

P29: 18. Transcripcion Carlos Hernandez.docx - 29:10 [es un sistema que prometió ten..]  (49:49)   (Super)
Códigos:	[modelo de salud actual] [modelo de salud futuro] 
No memos

es un sistema que prometió tener múltiples planes, competidores en múltiples beneficios y lo que yo considero es que no se ha logrado eso, tenemos muchos actores muchas EPS pero que en la realidad la EPS más grande de país en este momentico se llama supersalud, en este momento debería de tener el seguro social, caprecom, cajanal y fondos especiales hoy tenemos supersalud, salud total y Sura. Pasamos de un oligopolio a otro oligopolio, porque hoy en día están intervenidas, la gran mayoría, caprecom, cafesalud Coomeva, SOS, están intervenidas, ósea el verdadero asegurador ahí se llama supersalud, y otras pocas tratando de tener los equilibrios pero esas otras no van ni quieren recibir más traslado de población , el sistema arranco en una competencia basada en competidores y hoy en día ningún asegurador quiere recibir lo que están dejando porque seguramente no son viables por su estrategia de localización y en la medida que cafesalud cierra y entrega municipios, Coomeva entrega municipios SOS entrega municipios, esos resultados de esas otras entidades se empiezan a deteriorar entonces estamos resolviendo al final de la corriente los temas estructurales, y en este sistema vienen padeciendo el sistema de salud, los prestadores las EPS que estaban equilibradas, y no hemos ajustado ni el modelo de intervención de riesgos y determinantes de la salud, no estoy haciendo modelos de intervención público más costos efectivo, y tampoco estoy exigiendo resultados en salud, solamente lo tenemos para un evento llamado enfermedad renal que es la única enfermedad que se paga por post y no hay más pagos por resultados, deberíamos de tener un sistema estar corrigiendo esto y pago por resultado de salud. 


P29: 18. Transcripcion Carlos Hernandez.docx - 29:11 [Ya está colapsado, el sistema ..]  (53:53)   (Super)
Códigos:	[modelo de salud actual] [Protección financiera : financiación del SGSSS] 
No memos

Ya está colapsado, el sistema en este momentico en 30 y pico de años, 5 años de crisis llego al punto de no retorno, en este momentico hay más recursos para pago directo de los no post, no hay compra de cartera para las EPS, ya la cartera de las clínicas paso de 120  a 160 días, en este momentico el incumplimiento de la situación financiera no lo cumple ninguno, este año varias aseguradoras están perdiendo en los resultados operacionales que nunca habían tenido y algunos cuyos resultados operacionales eran buenos, tampoco alcanzan a tapar el resultado personal. Este momento es el punto de quiebre de no retorno, y si el tema de la reforma tributaria no se hace y no se ajusta la UPC esto no tiene retorno.

P29: 18. Transcripcion Carlos Hernandez.docx - 29:14 [CH: Las políticas de salud deb..]  (65:65)   (Super)
Códigos:	[modelo de salud actual] [modelo de salud futuro] 
No memos

CH: Las políticas de salud deben de tener cimientos, es como la ley 10 y la ley 60 previos a los orígenes de la ley 100, si usted le dice al municipio que es responsable y no le da la plata, de qué sirve la responsabilidad si no va a haber la plata. Entonces está pasando con el MIAS es que el modelo lo exige, pero no ha cambiado la obligación de que las redes surgen de una ley que aún sigue existente, las SSS son de orden municipal y departamental, ósea que no tienen ninguna obligación de integralidad, cada S puede montar los servicios que quieran. Entonces esa S puede tomar la decisión de no tomar el parto porque no es rentable, la otra considera que es mejor la endoscopia, la otra S toma la decisión y la ley le obliga a usted a articularse con lo que tiene y con que es lo que tiene, tiene lo que es rentable, lo que es rentable no lo ofrece. Como puede haber modelos integrales de atención si hay procesos de atención que no son lucrativos y la gente dejo de hacerlos, ese traslado de lo no lucrativo lo harán algunos a costa de quebrarse o tener mala atención, entonces cual modelo en redes de atención cuando hay competencia departamentales, municipales y hay libertad de esclarecer servicios según su rentabilidad, por eso en la propuesta en el principio de la conversación dije esto no puede la plataforma es que la obligación de la red principal de atención es publica, y entonces el secretario de salud no va a convencer a un director de S si quiere o no hacerlo si no que él es la autoridad municipal y que va a decir partos acá no en todos los sitios con criterios administrativos eficientes, de alta calidad, partos de alto riesgo acá, acá esto lo otro. Pero hoy en día son las S  las hay municipales, departamentales, entonces no, el modelo integral de atención es esto lo que yo le ofrezco y señor aseguramiento privado sus pacientes asegurados por el nivel 1 le cuesta en promedio 80000 y reciben los mismos servicios todos, ya el resto de servicios que le va a costar el nivel 2 lo puede hacer por las otras redes, pero lo básico cuesta eso, no es que usted me monte aquí una IPS para decir que hace cosas, no yo asumí la responsabilidad total y tengo la plata. Y él va hacer esfuerzos para que esto se mantenga bueno, no va a montar IPS para tratar de hacer en escala que no logra ni va a ir a los barrios porque no tiene como hacerlo entonces no tiene realmente un modelo de intervención de riesgos colectivos eficientes. Por eso el debería dedicarse al riesgo secundario, ya tiene la enfermedad acá se trata de detectar temprano y yo lo voy a controlar a cuidar, yo recibo una prima por eso, pero hoy en día recibe toda la prima por todo y entonces se privilegia a lo final porque es lo que más exige lo más urgente y poco hago en esta capa, y no lo hago con lo público pero lo público lo hace mal porque cada vez una entidad hace lo que quiere, cada S uno pregunta dónde está la ley de referencia y contra referencia publica donde dice parto de segundo nivel, ortopedia de segundo nivel, no yo no hago ortopedia, partos. No hay un sistema de resultados con responsabilidades para decir porque uno va a una unidad de cuidados intensivos en Cali y encuentra niños con Lúes, que es sífilis congénita y son pacientes de medicina prepagada, pacientes de régimen contributivo que quiere decir eso, que no es un tema que la gente le esté costando montar sistemas de captación, seguimiento de riesgo y cada quinen hace como lo quiere, pero no están habiendo resultados ese modelo tiene que cambiar, no es posible y no es sostenible un modelo MIAS cuando las reglas de juego estructurales básicas no están establecidas. 

P29: 18. Transcripcion Carlos Hernandez.docx - 29:17 [En salud en comfandi hemos hec..]  (81:81)   (Super)
Códigos:	[modelo de salud actual] 
No memos

En salud en comfandi hemos hecho cosas pero no todos tenemos la obligación, nosotros tenemos grupos que trabajan con las comunidades, nosotros tenemos auxiliares centinelas que llaman a los pacientes y los buscan en la casa. Pero cuando vamos allá encontramos la causa del niño desnutrido es una madre drogadicta, la madre drogadicta no hay quien la reciba las dos niñas, no tienen escolaridad no hay cupos escolares, entonces la paciente con una enfermedad rara la atiendo desde el punto de vista médico, pero los problemas sociales no están resueltos nosotros como caja le podemos dar vivienda a esa familia, pero resulta que está afiliada a la EPS pero no tiene caja de compensación familiar y eso es otro tema. Entonces yo no puedo llegar a un determinante porque a pesar de que administro determinantes no cruzan, y el problema social sigue siendo grave, hay problema de alcoholismo, está dentro del post, hay un anciano abandonado hipertenso, no hay quien lo cuide el fallo de tutela es encárguese del paciente, pero está en su casa y no sabe que medicamente es. Entonces esos problemas no hay una oficina de bienestar social de presupuesto social, no hay hogares para los ancianos abandonados, y lo estoy viendo desde el punto de vista asistencial. Entonces la protección social no es seguridad social, la protección social es discapacitados, drogadictos, ancianos, madres cabeza de familia reciben subsidios que se convierten en plata y no intervenciones sociales y eso genera enfermedades. Nosotros estamos con una pedagogía, para esto y con esto damos pañales, ponemos cuidadores se hace esto, pero lo estructural no está resuelto, y uno dice las políticas deben llegar un ingreso mejor, la gente no puede hacer evasión cuando hay un nivel de pobreza los recursos yo tengo un sistema de protección social, el anciano lo incorporo y esto va a costar un poco menos y puede ser viable, y los impuestos podrían ajustarse para eso. Pero si yo consigo la tutela para esto sin resolver esto.


P29: 18. Transcripcion Carlos Hernandez.docx - 29:18 [Pero tienen que pagar, si a us..]  (85:85)   (Super)
Códigos:	[modelo de salud actual] 
No memos

Pero tienen que pagar, si a usted le dice un médico vea usted en Cali se gana 3 millones, en Tuluá se gana 3.5 y en vichada Guajira 6 millones, son decisiones que se vuelven públicas, que hoy en día están basadas en aseguramiento, y lo que hace el aseguramiento es contratar al hospital local, y el hospital atiende a todo el mundo, entonces el hospital no logra desarrollar estímulos para eso, si nosotros dijéramos todo parto en Colombia se va a pagar a 800.000 pesos el parto, y las cesáreas 600.000 pero no lo han dejado a la mano invisible del mercado entonces yo prefiero hacer cesárea y me voy rápido y cobro más, en cambio si el parto es la lógica para evitar complicaciones y eso debe ser 70/30, pues pongo la política para que eso que no hace el mercado usted lo define y le dice se paga a millón el parto y a 600.000 la cesárea y se ve que el comportamiento humano y la conducta cambia, esos son temas de políticas, y eso automáticamente se va revirtiendo, sino que hay demasiadas cosas y hay que hacer chek list, ah el estado dijo que va a intervenir poco porque si intervengo se me rebotan las sociedades se me rebotan, los médicos, las clínicas entonces hay que hacer esos esfuerzo

P30: 19. Transcripcion Felipe Garcia.docx - 30:1 [Yo tengo pues una opinión aunq..]  (7:7)   (Super)
Códigos:	[modelo de salud actual] [modelo de salud futuro] 
No memos

Yo tengo pues una opinión aunque no todos la comparten, el problema en Colombia se ha centrado en dos grandes talones de Aquiles, lo que llamamos el componente humano de lo que llamamos los prestadores en salud, y ese componente humano pasa por muchos casos una deficiente formación clínica, que hace inclusive que el mismo sistema empiece a fallar desde la base de poder valorar diagnosticar al usuario, si uno mira por ejemplo a partir de la ley 100 toda la parte de la EPS las IPS, se miraron siempre con una visión muy empresarial muy administrativa, y no se dieron cuenta que el sistema es tan complejo que no se pueden prevenir los costos a priori, pongo un ejemplo una persona que ha cotizado 3 meses o 1 año, y se va a cuidados intensivos 15 días cuando por lo menos son 3 millones de pesos diarios ya cuanto gasto, eso le crea una debilidad al sistema pero además de eso los que trabajan en esas unidades de cuidados intensivos no tienen como una formación suficientemente buena por lo menos en las clínicas de combate, no estoy hablando de los hospitales ni las clínicas elite,  para que ese paciente tenga un buen tratamiento y por lo menos se salve. Entonces ese es un problema que viene mucho de la formación del médico de la formación en salud es la calidad en formación que no voy a entrar a criticar, pero en general el sistema de educación en personal en salud, principalmente los médicos esta deficiente, salvo alguno ejemplos algunas universidades, algunas facultades, algunos programas de medicina, tienen como esa visión pero en general es una formación muy tecnológica, muy de tomar un cuestionario y decir ta ta ta, entones usted tiene esto, y están considerando que esa persona es un formulario, no es una maquina ella siente. Lo otro es el componente es el tipo financiero, es claro que en el mundo los sistemas de seguridad en salud no pueden autofinanciarse, primero porque hay diferencias en edad y las afecciones van cambiando, no es lo mismo de un niño o una persona de 18 años a una persona de 40 años que se llama la tercera edad y lo que se ha visto es que los costos de atención y los costos de mantenimiento y aun los costos de programas de prevención van aumentando en medida que aumenta la edad de la persona, y desafortunadamente muchos sistemas y muchas sociedades se están yendo a la pirámide invertida, se engruesan a la mitad pero también tienen un gran engrosamiento hacia la parte final, Colombia está en eso, esta caracterizada como una pirámide que tiende a hacer un país viejo, un país en el que la gran población está por encima de los 40, 50 años y eso también le trae costos muy fuertes a un sistema público de salud. entonces esos dos componentes provocan lo que para mí es una crisis, y la legislación actual no la va a resolver por dos razones, primero la formación tiene mucho que ver con un creciente resurgimiento en facultades y programas de medicina, con una formación no completa para casi satisfacer un mercado laboral médicos generales que vayan a las EPS, y lo otro los costos de atención básicamente día a día que se tecnifica la medicina los costos de equipos y los costos de laboratorios y exámenes vayan más grandes, porque la medicina obvio ha avanzado en términos de manejo, en términos de hacer más preciso el diagnostico que se hace, y entra los costos de toda esa gran tecnología que vamos a ver más adelante es muy grande. Desafortunadamente tiene que acudir a un sector llamado el privado que es el que va a invertir pero quiere recuperar también. 


P31: 20.Transcripcion Hellen Mendoza.docx - 31:5 [es que todo debe conservarse, ..]  (19:19)   (Super)
Códigos:	[modelo de salud actual] 
No memos

es que todo debe conservarse, hablemos uno por uno, cobertura el concepto de cobertura debe cambiar, nosotros los colombianos decimos y nos llenamos la boca diciendo que somos s uno de los pocos países del mundo con una cobertura casi del 100%, pero cuando nosotros miramos la cobertura y miramos a que tienen derecho nuestro pacientes vemos que una cosa es el contributivo y otra cosa es el subsidiado y a su vez una cosa es el subsidiado en las cabeceras municipales y otra cosa es el subsidiado en la red dispersa en los municipios y las veredas. Un paciente con un carnet de subsidiado puede tener acceso a una consulta de especialista y la pueden dar en 6 meses, entonces usted tiene una cobertura supuesta porque tiene un derecho a un servicio pero que no se está prestando efectivamente, entonces yo pienso que nosotros debemos definir lo que para nosotros es cobertura y que no sea una cobertura de tener el derecho si no que ese derecho de vedad sea efectivo. La unidad de pago por capitación tenemos que tenerla hay que recalcularla, la unidad de pago por capitación que hoy existe para solventar los pagos de salud no es suficiente, para poder garantizar la prestación del servicio. Capacidad instalada, nosotros en Colombia no tenemos camas suficientes para atender a la población y la distribución de la capacidad instalada a su vez en Colombia no está bien organizado, nosotros hoy hemos permitido que numerosas clínicas y hospitales en el país hayan cerrado la atención de maternidad y pediatría porque son los servicios que menos rentabilidad financiera presta, eso nos está llevando a un problema de salud publica, nosotros hoy no tenemos suficiente clínicas y hospitales donde se atienda estantes donde se atienda partos y población menor de 14 años, eso afecta más la capacidad instalada, si usted va hoy en Cali haber quien atiende pediatría va a ver que el Pareto de la población clínica Versalles y el club Noel, la gente está literal en los pasillos y el club Noel literal en los pasillos que tienen adentro del club Noel porque no hay donde ubicar a los pacientes, las maternas las atienden en los partos en la clínica Versalles, ya no hay más donde atender maternidad y acá no pasa nada, acá todas las clicas van cerrando los servicios menos rentables, hace más o menos 15 días renunciaron todos los pediatras de la clínica farallones, refiriéndose según ellos a dificultades irreconciliables con la alta dirección y no pasa nada y la población de Coomeva la  pediatría hoy no tiene donde atender. Y así fue pasando todo se acabó maternidad en remedios, se acabó maternidad en clínica de occidente, Imbanaco está orientado al mercado de la prepagada, fundación valle del Lili está orientada al mercado de alta complejidad como debe ser para una institución de este nivel, pero quien atiende a la mediana y a la baja, se nos olvidó entonces eso significa que no tenemos capacidad instalada para atender a lo que tenemos que atender. Sostenibilidad financiera pues se cae su peso en la unidad de pago por capitación no alcanza, hoy tenemos unas dificultad grande de cartera, las instituciones prestadoras de servicios de salud con corte a hoy 30 de abril no han recibido nada de pago en lo que lleva recorrido del año, nosotros tenemos una rotación de cartera fácilmente mas de 160 días, teniendo que hacer pago de proveedores y de absolutamente todo lo que tenemos que subsanar y no tenemos plata para soportar la prestación. Los modelos de prestación en salud usted como garantiza la atención de un modelo de atención en salud cuando se vuelve difícil garantizar la prestación, es muy complicado tenemos problemas de autorizaciones, tenemos problemas de temas de direccionamiento de algunas aseguradoras de algunos pacientes que tienen, entonces es complicado sin embargo las IPS en mi modo de ver están trabajando y han tratado en la medida posible la continuidad de los modelos de atención.


P31: 20.Transcripcion Hellen Mendoza.docx - 31:7 [Yo creo que la única saluda qu..]  (23:23)   (Super)
Códigos:	[modelo de salud actual] 
No memos

Yo creo que la única saluda que tenemos de verdad es hacer realidad el MIAS, es la única salida que tenemos, no se va a ver mañana tampoco pasado, pero si no arrancamos hoy no vamos a arrancar nunca, esta generación está en un modelo curativo y está siendo atendida por IPS donde el asegurador ha trasladado la gestión del riesgo y eso tiene incentivo a perderse cuando no se vigila de una manera adecuada, si nosotros iniciamos hoy la implementación del MIAS yo pensaría que en unos 20 años exagerando nosotros podemos tener una población mucho más sana. 

P31: 20.Transcripcion Hellen Mendoza.docx - 31:13 [HM: y si usted se va a eso y v..]  (47:47)   (Super)
Códigos:	[modelo de salud actual] 
No memos

HM: y si usted se va a eso y vamos un poquito mas y nos adelantamos un poquito el proyecto MIAS hoy la capacidad instalada de las instituciones de salud en colombiano, está instalada en la curación, entonces donde está la capacidad instalada para la prevención como el proyecto lo contempla, no esta no existe hay que montarla, la sostenibilidad financiera, estamos hoy en un punto de crisis del cual no sé cómo vamos a salir el modelo de atención en salud ahí está, estamos tratado lo humana mente posible y creo que nos ha ido bien porque a pesar de todo tenemos muy buenos indicadores, y un sistema que para mí es parcializado, cuando yo escuche en una entrevista que hizo el ministerio más o menos hace 3 semanas cuando estaba hablando y promocionando el libro que saco que se llama controversia, él decía que fuera del chiste el gobierno colombiano en cuestiones de salud había sacado una calificación muy positiva en los indicadores macro de gestión, y que estaba apuntándole de una forma bastante aceptada  a los compromisos que como gobierno se habían hecho a nivel internacional, y usted va y los mira y nos devolvemos a lo mismo, cobertura si maravilloso grande, esperanza de vida y es verdad desde el punto de vista macro usted dice acá no está pasando nada, pero cuando usted entra al fondo y le toca levantase la plata para pagar la nómina, cuando a usted le toca irse a sentar a la puerta de los bancos a rogar que lo atiendan cuando usted le toca pararse con el proveedor y decirle mira no te puedo pagar porque no tengo flujo de efectivo, cuando nos toca pagar todo más caro porque nos toca fiarlo, cuando llegamos a un momento literal no podemos funcionar porque no nos han pagado es cuando uno dice bueno a costa de que estamos nosotros garantizando el cumplimiento de los indicadores país. 

P31: 20.Transcripcion Hellen Mendoza.docx - 31:17 [HM: Si esto no cambia no hay f..]  (66:66)   (Super)
Códigos:	[modelo de salud actual] 
No memos

HM: Si esto no cambia no hay futuro, profe no tenemos con que pagar proveedores pasado mañana. Si esto no cambia lo veo colapsado, mire por ejemplo que paso el mes pasado no sé si el doctor Hernández le comento algo de esto en la entrevista que tuvieron pero usted vio que cambio el sistema de pago de los trabajadores independientes, cambio la forma en que se recauda la plata entonces ya los trabajadores independientes y las personas que cotizan ellas mismas ya no se paga mes anticipado sino mes vencido eso empezó a partir del 1 de marzo. Resulta que el sistema se compensa con la plata que entra porque cuando usted paga esa plata llega a la subcuenta y la subcuenta entonces hace la compensación y a cada EPS se le da su plata y a su vez la EPS  les paga a las IPS. Pues resulta que ese traslapo que hubo en los cambios de fecha de anticipado las EPS no recibieron plata y como las EPS no recibieron plata las IPS tampoco. 

P31: 20.Transcripcion Hellen Mendoza.docx - 31:18 [HM: Hay una migración en los t..]  (74:74)   (Super)
Códigos:	[modelo de salud actual] 
No memos

HM: Hay una migración en los temas de contratación y si usted me pregunta yo estoy de acuerdo, hay durante muchísimos años en Colombia hemos vivido con 2 modelos de contratación, un modelo de contratación de capitación, y un modelo de contratación por pago por eventos o pago por actividad, la capitación en su momento y como la vivimos todos durante muchísimos años, era un traslado del riesgo técnico y el riesgo primario, es decir la IPS se convertía en una EPS chiquita que le garantizaba a la EPS la atención de los 3 niveles de complejidad de un paciente y para la EPS no había problemas porque había un traslado de todo su riesgo tanto el técnico como el primar, entonces la EPS garantizaba solamente la plática para manejar el 4 nivel, una que otra exclusión del tercero y el manejo de la administración, durante todos esos años el peso recaía sobre las IPS. Con el cambio de las normas y con la obligatoriedad de la sola contratación de la cápita por complejidad para el primer nivel otra vez nosotros pasamos al famoso evento o pago por actividad, y se empezaron a trabajar otros modelos de pago prospectivos, en esos modelos de pago prospectivo entro el famoso pack, entro el PGP, dentro del pago están estos niveles de contratación, la cápita es un pago global prospectivo pero empezaron a haber formas diferentes de hacer estos contratos. Mientras todo eso maduraba hubo algunos modelos de contratación que fueron perversos para las IPS y eso contribuyo a que muchos tuviera un problema financiero aun mayor, hoy hay un desarrollo interesante y hoy los modelos d apago global y prospectivo no son de traslado al 100% del riesgo técnico y el riesgo primario sino que son rasgos compartidos, donde yo te traslado el riesgo técnico no te traslado el riesgo primario pero empezamos a mirar y construyamos la nota técnica en conjunto, de tal forma que si tú te quiebras yo te apoyo y si yo me reviento estamos trabajando para la misma vía. 

P31: 20.Transcripcion Hellen Mendoza.docx - 31:19 [HM: lo que pasa es que hay alg..]  (78:78)   (Super)
Códigos:	[modelo de salud actual] 
No memos

HM: lo que pasa es que hay algunas modalidades dentro de los modelos de pago prospectivos, que son los famosos pagos por desempeño, no es una modalidad de contratación perse, es un anexo a algún tipo de modalidad de contratación, por ejemplo usted va a coger una población de 50000 personas y usted va a hacer un modelo de contratación una cápita, y dentro de esa cápita le voy a paga a usted por la población que efectivamente tenga una citología vaginal, usted enloquece a hacer citología vaginales. Esto es puro modelo de agente principal, esto es pura teoría de ante principal entonces que pasa, cuando le estoy dando un incentivo por una cosa y no le doy por la otra siendo la otra probablemente igual o mejor, por ejemplo uno loco porque hoy en día los están pagando las EPS, la demanda inducida entonces centro todo mi personal a hacer citologías y pierdo la demanda inducida, no le paro bolas porque no me lo van a pagar adicional, como no me lo van a pagar adicional yo bajo la guardia y como bajo la guardia se me empieza a enfermar la población, entonces no es que la IPS  le castiguen por ser bueno, es que hay también teorías de agente principal. Entonces donde debemos mostos trabajar o cuales son los modelos ideales, en donde yo comparta el riesgo y donde yo pueda trabajar una nota técnica con la información sobre la mesa, donde el análisis actuarial sea un análisis compartido donde ambas empresas puedan trabajar en conjunto y si se puede. 

P31: 20.Transcripcion Hellen Mendoza.docx - 31:21 [HM: Los médicos cuando salió l..]  (97:97)   (Super)
Códigos:	[modelo de salud actual] 
No memos

HM: Los médicos cuando salió lo del mipres al principio todo el mundo estaba contento y yo veía a los colegas felices y yo no podía entender como un ser humano que ha hecho una universidad y haber salido de un pregrado tan difícil como es graduarse de médico, no entiende el fondo que tiene el mipres, él no te da el 100% de la autonomía eso es mentira el mipres no te está quitando a ti hacer un proceso de un formato lo único que cambia es que no lo haces a mano pero lo haces en el sistema el mipres te está diciendo que con tu código, y con tu nombre en línea el ministerio se va a dar cuenta quienes son los grande generadores del no por en este país. 

P32: 21.Transcripcion Oscar Gutierrez.docx - 32:1 [El tema de salud en Colombia e..]  (7:7)   (Super)
Códigos:	[modelo de salud actual] 
No memos

El tema de salud en Colombia está muy bien planteado dio cobertura teórica al 95%  de la población, pero en la práctica es inaplicable. 


______________________________________________________________________

Código: modelo de salud futuro {162-0}

P 8: 1. Transcripcion Martha Lucia  ospina.docx - 8:12 [Adicionalmente en el tema de p..]  (7:7)   (Super)
Códigos:	[modelo de salud futuro] 
No memos

Adicionalmente en el tema de prestación recuperaría la figura verdadera de la cápita como un modelo compartido y para eso habría que introducir cosas como el pago por desempeño y prohibiría obviamente ciertas prácticas, cuando logro óptimos resultados en desempeño por resultados en salud entonces lo renegocio y le quito la ganancia, y eso ha hecho que las IPS se defiendan y que las IPS no quieran trabajar por capitación solo por actividad, porque cada vez que lograban resultados, eficiencias les quitaban el ahorro y es injusto, entonces se desfiguro la figura de la cápita como esa figura de pago anticipado para garantizar la buena gestión y por eso paso lo que paso

P 8: 1. Transcripcion Martha Lucia  ospina.docx - 8:13 [Y recuperaría algo que se ha s..]  (7:7)   (Super)
Códigos:	[modelo de salud futuro] 
No memos

 Y recuperaría algo que se ha satanizado en el sistema que es la integración vertical otros países como Inglaterra promueven la integración vertical a mí me parece que debería existir pero basada en un certificado en no disponibilidad de oferta, porque en regiones apartadas no tengo la oferta y nadie la quiere poner y la EPS que está llevando no se puede integrar verticalmente porque está prohibido, entonces si yo tengo un certificado de que no tengo la oferta debería casi ni siquiera permitirse y obligarse a que haya integración vertical. Al prohibir la integración vertical, se prohibió por los intereses de las grandes clínicas que están adjudicadas en las ciudades, pero la integración vertical y los sitios donde no hay ofertan y en las zonas rurales dispersas debería ser promovida, es el único modo de tener una oferta en el país

P 8: 1. Transcripcion Martha Lucia  ospina.docx - 8:14 [Adicionalmente las capacidades..]  (7:7)   (Super)
Códigos:	[modelo de salud futuro] 
No memos

Adicionalmente las capacidades básicas de las entidades territoriales han desaparecido, las secretarias de salud son reducidas a morir y fuera de eso tienen unas plantas congeladas desde hace mucho tiempo, y la gestión no se ve porque cada vez que hay cambio, se barre con su cuadrilla  y eso barre con cualquier reconocimiento posible. De hecho cuando hicimos el plan de Ensanar, hay un capitulo que se llama fortalecimiento de la autoridad sanitaria, realmente se llamaba recuperación de la autoridad sanitaria pero no era correcto y lo cambiamos, si no se recupera la autoridad sanitaria local estamos fregados, y se necesita que haya de nuevo plantas de concursos de méritos, que no haya esa capacidad de contratación tan enorme para que las plantas existan de manera sólida pero con profesionales verdaderos, no plantas echas a punta de auxiliar, no es que el auxiliar no necesita trabajo, pero no es así eso sirve para politiquería y no para tener capacidades técnicas en un territorio. Entonces eso hace a no tener planta a no tener capacidad de un nivel técnico las entidades no tienen ningún tipo de control y no pueden hacer todo lo que realmente les toca, entonces podríamos hablar mucho mas de eso pero yo creo que eso del sistema habría que recuperar y que habría que modificar en general. 

P 8: 1. Transcripcion Martha Lucia  ospina.docx - 8:15 [como me lo imagino viendo como..]  (11:11)   (Super)
Códigos:	[modelo de salud futuro] 
No memos

como me lo imagino viendo como están las cosas. Pienso que como están las cosas es un sistema que tendrá cada vez consolidadas grandes IPS privadas pocas y grandísimas inmensamente ricas que ejercen una presión política importante y tienen una capacidad de negociación absoluta que serán las grandes ganadoras del sistema estoy hablando de enormes IPS privadas. Pienso que sobrevivirán algunos hospitales públicos con excelentes administraciones, y el resto entrara en una crisis cada vez mayores y tendrán que ser liquidados, la liquidación EPS llevara a una acumulación de deudas sin posibilidad de pago, lo que llevara a que digamos que hay unas pocas EPS que tal vez resulten de la unión de otras y con un impulso de capital puedan seguir viviendo bajo las mismas reglas de juego, pero quedaran acumulando deudas vieja que no pagaran nadie y llevaran a la quiebra a las IPS pequeñas, eso creo que pasara

P 8: 1. Transcripcion Martha Lucia  ospina.docx - 8:16 [Cual serie el sistema soñado e..]  (15:15)   (Super)
Códigos:	[modelo de salud futuro] 
No memos

 Cual serie el sistema soñado en el sistema soñado primero que todo haría un ejercicio muy juicioso de la UPC, lo que te dije ahora pero insisto porque es la fase del financiamiento con mayores variables de productividad con una mayor UPC que tuviera un R2 por lo menos del 30% hay información para lograrlo y que no sería una UPC fija, es decir que yo no fije la UPC el 28 de diciembre, si no que yo lo que haría en ese nuevo modelo mes a mes se corre la población del asegurador, es decir le corro la fórmula de la población de la aseguradora y le reconozco la plata, para que yo como asegurador tenga realmente incentivos verdaderos para gestionar el riesgo de mi población, es decir me están reconociendo mis enfermos renales, me están reconociendo el trabajo con los enfermos de cáncer, reconocen mi población mayor, es decir eso que hago para mantener los bien y tenerlos ahí es mas no tendría que empezar a sacar la población vieja, los tendría porque me los están reconociendo mes a mes y mantenerlos seria visible, lo que estoy visibilizando el riesgo poblacional y la UPC tendría esos reconocimientos pero además funcionaria el ajuste al post, como el que hace la cuenta de un puesto pero mejor, con más enfermedades. De modo que yo tengo esa población pero si hago las cosas bien recibo incentivos, esa es una visión súper dura para el estado pero no es imposible se puede hacer, ósea que tendría una unidad muy fuerte de aseguramiento que me de los reconocimientos mes a mes, entonces eso me da las reglas del juego claramente me dice que la población de Colombia, es visible y el asegurador que tenga poblaciones de alto riesgo peor que las mantenga bien y a tiempo, no se le compliquen no se le mueran recibe más plata, eso es alinear los incentivos para que sean virtuosos y no perversos,

P 8: 1. Transcripcion Martha Lucia  ospina.docx - 8:17 [resulta que uno puede sacar no..]  (15:15)   (Super)
Códigos:	[modelo de salud futuro] 
No memos

 resulta que uno puede sacar normas pero si los incentivos son contrarios la gente actuara de manera contraria y esto es una cosa loca porque una norma dice una cosa y otra, pero las circunstancias decide otro. Entonces al alinear eso la población tiene valía, si un señor de unos 70 años con múltiples enfermedades, si se mantiene bien recibe un dinero suficiente para poderlo manejar

P 8: 1. Transcripcion Martha Lucia  ospina.docx - 8:18 [claro, llevaría, y al tener es..]  (19:19)   (Super)
Códigos:	[modelo de salud futuro] 
No memos

claro, llevaría, y al tener ese reconocimiento yo como EPS que hago pues ahí si me volteo para meterle todo a la gestión de riesgo, porque primero me está viendo y luego me están reconociendo si lo tengo y lo tengo identificado no se me complica no s eme muere, entonces ahí el modelo voltea y adicionalmente ese sistema de pago tendría un reconocimiento especial por zonas es decir que tendría que tener una UPC diferente en zonas urbanas, en zona rural y en zona rural dispersa, yo me meto a estar en Tumaco bajo condiciones de Tumaco pero tengo un reconocimiento especial porque tengo unos desplazamientos costosos, tengo una serie de cosas que aunque se pensó en el pasado desapareció y en este momento seria visible pero no solamente que me den la plata, me dan unas reglas de juego y las reglas son las siguientes

P 8: 1. Transcripcion Martha Lucia  ospina.docx - 8:23 [Y recuperar esas figuras en es..]  (27:27)   (Super)
Códigos:	[modelo de salud futuro] 
No memos

Y recuperar esas figuras en esos modelos y en esos agentes comunitarios. La aseguradoras que quedaran necesariamente tendría que ser evaluadas por el consejo de estado y esas aseguradoras serían las encargadas de dar el valor agregado porque si hubiera un monopolio del estado, básicamente podríamos volver al mundo de que es malo pero es lo único que hay, no puede ser debe existir el mundo de lo diferencia de que haya un valor agregado. Lo que no se puede permito r de ninguna manera es que haya población en la misma región es decir la que está en el valle es solamente una, la que hay en Valle Cauca Nariño es una sola, pudiera responder por la región. Porque la atención primaria en salud está pegada a la región y poder interactuar con el departamento. En el modelo que te digo también habrá que recuperar la autoridad sanitaria y habría que recuperar las plantas de cargo de la secretaria de salud, rediseñando la secretaria de salud como debería ser hoy en día con los roles que debería tener no con los de 1980. Y eso digamos garantizaría el IBC el tema de habilitación de riesgos, el tema de emergencia sanitaria, todos los roles que no son capaces de cumplir porque cada secretaria tiene un diseño único que se lo inventan ellos, y en ese modelo del sistema de salud una cosa muy importante, los indicadores de desempeño serian diferenciales si es una IPC grande privada o una IPS grande publica o una pequeña IPS serían diferenciales, mala cosa lo que estamos haciendo todos en una misma cosa porque la IPS grande con ese dinero que tiene le es indiferente, hay prestadores pequeños muy buenos que hacen un esfuerzo enorme por hacer las cosas bien, pro no tienen la capacidad financiera para hacer todas esas cosas que se les pide ahí y están desapareciendo de regiones donde se necesitan. prestadores pequeños que van a hacer servicios por ejemplo en el norte del cauca que van a pequeños departamentos pero no pueden responder por ciertas características y ciertos indicadores que les piden a una IPC grande que es indiferente, entonces esa apreciación lo más delicado que yo veo en este momento es que los prestadores son una masa, pero no resulta que hay diferentes tipos de prestadores unos pequeños y unos muy ricos y cuando los miden con el mismo rasero el pequeño sale mal calificado y no es que sea malo, está en su contexto y es bueno para hacer lo que hace. Entonces habrá que hacer ejercicios diferenciales y en esos saldrían a la luz muchas coas, prestadores grandes que tienen unas prácticas muy complicadas en el sistema, tresnes que montan al paciente en una cadena de consumo pero nadie los detecta, pero si tuvieran una serie de indicadores diferentes que detectara que están haciendo, mucha mayor diferencia haría un tatequieto, haría las cosas bien  pero no serían los grandes ganadores y los inmensos ricos de este sistema, en general creo que eso establecería situación.

P 8: 1. Transcripcion Martha Lucia  ospina.docx - 8:24 [la salud es un concepto social..]  (35:35)   (Super)
Códigos:	[modelo de salud futuro] 
No memos

 la salud es un concepto social es un colectivo,

P 8: 1. Transcripcion Martha Lucia  ospina.docx - 8:25 [de salud tan bonita que tiene ..]  (35:35)   (Super)
Códigos:	[modelo de salud futuro] 
No memos

 de salud tan bonita que tiene la comisión de recursos humanos de la organización mundial de la salud, que dice que la salud pública es el compromiso de la sociedad con su ideal de la salud, es muy bonito porque el compromiso de la sociedad entendido como entidades y personas, es esa puesta que todos ponemos al ideal de salud, entonces no solo necesitamos salud, necesitamos pensión vivienda, calidad de comida, esa es la verdadera salud. p

P 8: 1. Transcripcion Martha Lucia  ospina.docx - 8:26 [pues le da el enfoque a acceso..]  (35:35)   (Super)
Códigos:	[modelo de salud futuro] 
No memos

 pues le da el enfoque a acceso a servicios de salud por un lado son como dos pensamientos encontrados, por un lado uno descansa en el estado y dice bueno por lo menos me la puso bajita, y lo digo por el plan de CENAE yo negocie con cada ministerio el plan de CENAE  y fue muy difícil, fue terrible cuando yo fui a hablar al ministerio de cultura con una paisana y le explique que en la gran consulta ciudadana había salido todo el tema de la cultura del colombiano del colombiano ventajoso, de traposo egoísta, esa esencia del egoísmo que teníamos que trabajar, me dijo no usted está equivocada yo no soy el ministerio de cultura, yo soy el ministerio del festival vallenato del festival de cine de Cartagena, del carnaval de Barranquilla entonces me quede aterrada y le dije es el ministerio del folclor, que es distinto y seguramente es muy importante en la cultura del folclor, y con eso uno sale muy aburrido y cuando me dijo no hable con el ministerio de educación y entonces donde esta esa esencia de ser colombiano, quien va a trabajar a apostarle por ese asunto de cultura, quien va a trabajar cuando 150000 colombianos dijeron que lo que teníamos era una deforestación impresionante en las ciudades, y con el estudio en deforestaciones nos damos cuenta que Colombia tiene 3.5 por 100000 personas cuando el mínimo indicado por mes es 10 y Nueva York tiene 50, nosotros somos un país deforestado no cuenta la selva ni nada solo las ciudades, pero no cuando uno ve lo normal que están haciendo cada vez 0 árboles, entonces donde está la coherencia de lo que la gente quiere con lo que se hace. Entonces si la ley estatutaria hubiera salido con la verdadera definición yo lo veo muy difícil, porque además una cosa terrible que es un error de la ley estatutaria le pone la tarea al sector salud, a uno no le pueden delegar responsabilidades sin responsabilidad eso es una locura, entonces como le dicen me hace el favor y hace esto cuando no tengo la capacidad de hacer vías de acceso para llegar a choco, yo no construyo carreteras entonces eso es una paradoja

P 8: 1. Transcripcion Martha Lucia  ospina.docx - 8:27 [que a 2023 la primera causa de..]  (55:55)   (Super)
Códigos:	[modelo de salud futuro] 
No memos

que a 2023 la primera causa de mortalidad en Colombia será el cáncer, no lo cardiovascular, el cáncer va a puntear en el 2023, pero salen otras nuevas y no transmisibles que no estábamos viendo que son las enfermedad mental suficientemente pesada para verla y se nos va a observar con el postconflicto se va a hacer invisible, y tenemos todo lo osteo muscular, todo lo osteomuscular esta por estilo de vida está dado por temas laborales y por unos bajos cobertura de ARL con una gran informalidad en el trabajo, pero timaban por la presión de la industria farmacéutica que hace que haya mucha formulación y mucho uso de antiinflamatorios no esteroideos y ese tipo de cosas para estas enfermedades. Seguimos teniendo presencia de enfermedades infecciosas y eventos de casa externa pero ya nuestra primera causa no es el homicidio como en el 2006, cayo a quinto puesto, entones que pasa porque sabemos que esto empeorara, primero por el envejecimiento poblacional, tenemos claramente y concentrada en algunas regiones como en valle del cauca, el eje cafetero, Tolima, Cundinamarca y Boyacá, la población más envejecida de Colombia. Ese envejecimiento acompañado de un mal estilo de vida, tenemos un bajo índice de actividad física el 26% en educación escolar y solo el 46% las personas de 18 a 60 años en Colombia tienen actividad física mínima para la edad, menos de la mitad de la gente. Tenemos todavía un 12% en consumo de tabaco, tenemos lo que más  me preocupa obesidad creciente, entre la encuesta de 2005 y 2010 hubo un crecimiento de promedio de perímetro de la cintura de 10 cm, esto significa que si nosotros ahora que entréguennos los resultados a final de año hay un crecimiento igual tenemos una epidemia de obesidad y ningún país del mundo ha logrado revertir la cifras de obesidad, las cifras de obesidad es irreversible para la población, ósea que hay que pararla como sea y tiene que ver con el cambio de habito, el tema de hábitos es tan grave espere le doy las cifras que eso es lo que viene, todo lo que te estoy diciendo haz de cuenta que es una receta de cocina que yo le echara cosas malas, el envejecimiento falta de actividad física y todo me va quedando la receta, al final el pastel que nos va a quedar es una carga de enfermedades crónicas inmanejable, pero como ve uno no tiene el cambio en su mano en el sector salud, el tema de dieta en un país donde son más caras las frutas y verduras que los carbohidratos, un país productor de frutas y verduras sin estaciones, entonces eso se habla con el ministerio de cultura algo tiene que pasar nosotros si no invertimos la dieta en los seibós de salud, un país que privilegia el empleo y se siente feliz por el empleo que da la industria tabacalera, el empleo que da la industria tabacalera no paga nunca las consecuencias que tenemos en los servicios de salud, hacemos la de el bobo, recibimos 3 pesos para gastarnos 3000 por acá, porque el estado en su globalidad no piensa en ese concepto de salud si no en manera fraccionada. Entonces eso le toca a un ministro de salud no es una tarea fácil yo he visto con mis propios ojos a dos ministros decirlo, esto es así pero no es un tema fácil, no es un tema que digieran fácilmente otros sectores y eso es muy difícil eso es una cosa que nadie se imagina esos lapus, y la gente es absolutamente liviana con las opiniones, por eso es muy injusto eso es una labor injusta. 

P 8: 1. Transcripcion Martha Lucia  ospina.docx - 8:28 [El plan decenal es un antes y ..]  (59:59)   (Super)
Códigos:	[modelo de salud futuro] 
No memos

El plan decenal es un antes y un después, es muy difícil sacarlo del sector salud, el plan decenal tiene el 70% de actividades fuera del sector salud, en Colombia 1 de cada 3 no consume frutas diariamente, 1 de cada 2 es inactivo físicamente, 1 de cada 8 fuma, 2 de a cada 10 consume gaseosa y refresco azucarados diariamente. Mire ese caldo de cultivo que va sumando, por eso cuando le dicen al ministro de las bebidas azucaradas, que pesar esa pelea perdida antes de empezar, si tiene más calorías una bandeja paisa. Es el contexto lo que va produciendo el daño en salud y la dieta ancestral y en todos los pueblos del mundo la dieta ancestrales nunca han causado obesidad en su población, la dietas ancestrales nunca y acá llevan más de 100 años consumiendo bandeja paisa la dieta ancestral no produce daño la prueba está en los mexicanos que cuando tiene la introducción de la dieta  tienen todo ese consumo de bebidas azucaradas empieza la obesidad tan aterradora, mientras que las generaciones anteriores que están pegados a la dieta ancestral mejicana son flacos. Ellos son el ejemplo vivo de lo que es la modificación de la dieta ancestral y nosotros podemos vivir siempre comiendo sancocho y nunca nos vamos a engordar porque es nuestra dieta ancestral, entonces esto es muy grave 1 de cada 3 personas tiene consumo perjudicial de alcohol. 1 de cada 3 personas en Colombia tiene hipertensión no sabe que tiene hipertensión. 1 de cada 3 personas con diabetes no sabe que tiene diabetes, 1 de cada 3 personas con hipertensión y que tiene tratamiento nunca logra adherencia al tratamiento, 1 de cada 3 personas no sabe porque se crea la diabetes, la gente cree que es por comer dulces, y de las cosas más tristes que muestrea que nosotros vamos para allá es la perdida de la lactancia materna, pasamos de tener 2 meses de lactancia materna que es bajísimo hoy en día tenemos 1.4 meses de lactancia materna eso es nada, y lo más tiste las poblaciones indígenas tienen menos de 1 mes de lactancia materna exclusiva, entonces como no vamos a tener enfermedades crónicas en el futuro es para allá que vamos, y no tenemos otro camino mientras se modifique esos determinantes y esos determinantes no estaban en el sector salud y este ministro en particular se ha dado peleas de política pública y las bebidas azucaradas que la gente no entiende que contexto, es lograr cambio grandísimos que terminen modificando factores sociales que llevan a que hay amenos daño en salud.

P 8: 1. Transcripcion Martha Lucia  ospina.docx - 8:29 [Es muy importante precisar ahí..]  (63:63)   (Super)
Códigos:	[modelo de salud futuro] 
No memos

 Es muy importante precisar ahí y esa pregunta ya me la han hecho, no es una mortalidad evitable por los servicios de salud, ósea que los servicios de salud no evitaron el 53% de las muertes, es una mortalidad evitable general es decir, son cosas que no debieron llegar a ocurrir pudieron modificarse pero incluso desde cosas anteriores y esa metodología de mortalidad evitable que no hacemos nosotros el proceso de metodología que se utiliza en el mundo muestra solo las causas, entonces llego a responder con datos resulta que los servicios de salud solamente y con esta grafica que es de un artículo e los años 76 pero que ha sido repetida varias veces y vuelve y sale, los servicios de salud solamente pueden modificar la mortalidad en un 11%, el estilo de vida puede modificar el 43% y el entorno el 19% entonces volvemos a lo que te estaba diciendo, si no modificamos desde los otros sectores el tema de actividad física, los ambientes para poder hacer actividad física, la seguridad que permita tener actividad física, el tema de acceso a alimentos, alimentos que realmente beneficien la salud y eso tiene que ser con el ingreso, posibilidad de garantizar seguridad financiera en ciertas familias hay grupos indígenas que tienen alimentación jerárquica, ósea come el papa los padres, la señora y los hijos del mayor a menor, ósea que el ultimo que es el que sale en la noticia, es el último que come. Y si financieramente no hay por el ministerio de comercio una garantía financiera de ingresos en esa familia porque el ingreso que digamos era de contrabando y se quitó, entonces uno piensa que ese niñito se va a salvar porque lo atienden en un servicio de salud, ósea el niñito con ese tipo de nutrición, es más cuando le empiezan a hacer rehabilitación nutricional se muere, el servicio de salud termina matándolo porque esa recuperación aguda no es buena para ese tipo de casos, entonces no es un problema de salud es un problema de seguridad financiera. Y entonces la respuesta es si se podría modificar en gran medida esa mortalidad evitable si comprendiéramos un concepto ampliado de salud y los demás sectores realmente aportaran para modificar la calidad de vida.

P 8: 1. Transcripcion Martha Lucia  ospina.docx - 8:30 [Yo creo que puede mejorar si a..]  (75:75)   (Super)
Códigos:	[modelo de salud futuro] 
No memos

Yo creo que puede mejorar si aumenta el poder resolutivo del nivel 1, si el nivel 1 absorbiera como tenia planeado el 80% de la demanda al especialista llegara solo lo que debe llegar, eso por un lado si realmente aumenta el poder resolutivo y el poder resolutivo aumenta en la medida que se modifique la habilitación y se mejore la calidad de la educación medica eso por un lado, y obviamente que hayan incentivos adecuados para que el modelo de nivel 1 sea más resolutivo, mejor pago y pueda hacer más cosas. Por otro lado también debe permitirse, promoverse ciertas prácticas que en otros países son excelentes, acá se hacen pero están satanizadas pero es perfectamente válido correcto aprobado que un especialista pueda hacer una consulta eficiente con 4 o 5 médicos generales que lo acompañen, de esa manera aumenta la capacidad de ver pacientes, telemedicina y espejo tiene 4 médicos generales y el atiende a esos que estañen simultaneo, ciertas estrategias. En Canadá esta súper probada la consulta para diabéticos grupal, y ya está inventado es solo hacerlo acá, entonces mientras sigamos empeñados en tener una consulta individual solo para mí y en el tiempo que yo quiero estamos equivocados porque el otro factor es que tenemos que tener unos niveles de tolerancia a la espera, las citas en otros países son lejos y eso se hace a nivel de urgencias a nivel de riesgo, a mí me llama la gente, es que me dio cita para dentro de 1 semana y que si lo puedo ayudar en que una cita en una semana es una cita lógica y bien pensada y tiene que  privilegiarse por riesgo no es lo mismo conseguir una cita para un paciente hemofílico con un hematólogo que conseguir una cita con un paciente con arterio… para un control tienen diferente tipo de urgencias, y de riesgo. Entonces si se ajusta ciertas cosas, el nivel de tolerancia de la gente, la capacidad resolutiva del nivel 1, las modalidades de consultas y la remuneración a los especialistas creo que se podría mejorar los tiempos, no solamente con un papel de la supersalud, que la cita que da a tantos días, eso no tiene lógica ni sentido n se puede lograr. 

P 8: 1. Transcripcion Martha Lucia  ospina.docx - 8:31 [Se demora más de una hora, per..]  (79:79)   (Super)
Códigos:	[modelo de salud futuro] 
No memos

Se demora más de una hora, pero si la capacidad resolutiva del nivel 1 y la oferta del nivel 1 fuera mayor mucha gente no se iría por urgencias, urgencias es el sifón donde los pacientes terminan yendo, y el triage en los servicios de urgencias realmente determina bien porque hay mucha gente que no debería estar en urgencias y esa gente termina esperando porque tampoco se pueden devolver. Ento

P 9: 2. Transcripcion Alex Duran_ok.docx - 9:5 [Entonces digamos que en el tem..]  (15:15)   (Super)
Códigos:	[modelo de salud futuro] 
No memos

Entonces digamos que en el tema financiero debe estar mediado por el resultado, ahí con el MIAS el país está dando un vuelco muy grande al pago por resultados, pero definitivamente, en el modelo de aseguramiento es importante que digamos a las EPS, a las IPS, o a quien opera el sistema de les exijan resultados, eso digamos es lo malo que a la gente no se le paga por resultados si no por actividades. Y dos en ese mismo estimulo financiero, está el tema de que sea lo financiero precisamente la razón de ser de hoy en día de las EPS y de algunas IPS, entonces hoy en Cali a pesar de cómo funciona el sistema, usted ve emporio de clínicas creciendo porque prima lo financiero por encima de la gente, e instituciones como por ejemplo, el Hospital Universitario, reventadas porque no prima otro tema que no sea lo financiero, entonces ese balance del sistema de no es tan cierto que estemos mal, están mal los que prima, que no son las personas los pacientes,  si ustedes revisa cuales son las 500 empresas que más han crecido en los últimos 20 años en el valle, ahí tenemos cinco clínicas de Cali, y están alli. Pero porque, porque si usted allá no consigna por delante no le atienden al paciente, entonces no es cierto que estamos en crisis

P 9: 2. Transcripcion Alex Duran_ok.docx - 9:7 [Entonces digamos hoy tiene que..]  (19:19)   (Super)
Códigos:	[modelo de salud futuro] 
No memos

Entonces digamos hoy tiene que cambiarse ese ideal que se tiene del sistema, ningún sistema en el mundo es 100%  cubierto de todo, no cubre todo en ninguna parte del mundo, ni en Estados Unidos, ni en Europa ni en ningún lado, todos tienen su límite, pero acá hemos vendido la idea de que todo se puede y eso en ultimas va a reventar el sistema.

P 9: 2. Transcripcion Alex Duran_ok.docx - 9:10 [Mire yo pienso que definitivam..]  (35:35)   (Super)
Códigos:	[modelo de salud futuro] 
No memos

Mire yo pienso que definitivamente el modelo de salud que le conviene no solo a Colombia si no a cualquier país del mundo es el que se centra en la gente, el que prima la persona, el que nosotros vamos donde la gente, el que la salud le llegue a la gente y no con prestación de servicios porque ese es un error grandísimo del actual sistema, nosotros podemos hacer muchas clínicas, podemos hacer puestos de salud lo que tu quieres pero el tema no está adentro de las instituciones, eso es un concepto errado de los servicios de salud, yo creo en la atención primaria en salud, a pesar de que usted bien sabe he sido netamente operativo y hospitalario, yo si pienso que a punta de pasta y a punta de consulta no vamos a resolver el problema del sistema, tenemos que estar allá y tenemos que hablar de salud de determinantes sociales, por ejemplo para mi empleo es una política de salud, seguridad el tema de autocuidado, eso es salud, pero no haciendo clínica ni hospitales ni clínicas de salud.

P 9: 2. Transcripcion Alex Duran_ok.docx - 9:11 [Unas pocas, no creo que las 50..]  (39:39)   (Super)
Códigos:	[modelo de salud futuro] 
No memos

Unas pocas, no creo que las 50 y ahí viene lo perverso del sistema porque digamos lo que se está haciendo es una selección natural de las EPS más fuetes financieramente,

P 9: 2. Transcripcion Alex Duran_ok.docx - 9:16 [se puede demorar en promedio 1..]  (79:79)   (Super)
Códigos:	[modelo de salud futuro] 
No memos

se puede demorar en promedio 1 año en hacerlo, porque como es un modelo para todo el sistema del territorio, entonces por ejemplo aquí, tenemos que ponernos de acuerdo con las EPS, tenemos que ponernos de acuerdo con los prestadores, y eso que suena así de sencillo pero no es fácil, yo pienso que en un año, yo pienso como el texto y de ahí a la implementación 2, 3 años más, el modelo no va a estar listo en el corto plazo. 

P10: 3. Transcripcion-Alexandra_Matallana_ok.docx - 10:8 [Todo depende de la cifras macr..]  (74:74)   (Super)
Códigos:	[modelo de salud futuro] 
No memos

Todo depende de la cifras macroeconómicas del país, porque si nosotros tenemos una alta tasa de desempleo pues obviamente el subsidiado va a aumentar, el contributivo va a disminuir, y el sistema se ve totalmente impactado por esa variable macroeconómica que le afecta directamente en el momento, digamos que al sistema de salud esa variable del desempleo la afecta en todo, pero  la de la informalidad también. Porque es digamos que esa es el financiamiento del sistema y si el financiamiento cada vez disminuye esa base esta totalmente desfinanciado, si el desempleo crece y si la informalidad crece, pues el sistema se desfinancia. 

P10: 3. Transcripcion-Alexandra_Matallana_ok.docx - 10:14 [no yo pienso que ahí se va a h..]  (125:125)   (Super)
Códigos:	[modelo de salud futuro] 
No memos

no yo pienso que ahí se va a hacer una depuración importante se van a capitalizar más fuertes y van a desaparecer las más debiles, y la idea es que pues si desaparezcan las que tengan que desaparecer y bueno, ya controlar a mas pocas de pronto el control pude ser mayor no estaría tan atomizado y digamos que los juegos numéricos no deberían ser de tantas, serian de menos;  yo creería que si van a desaparecer bastantes. 

P10: 3. Transcripcion-Alexandra_Matallana_ok.docx - 10:15 [Claro, lo que nosotros estamos..]  (137:137)   (Super)
Códigos:	[modelo de salud futuro] 
No memos

Claro, lo que nosotros estamos haciendo es apostándole todo al tema y nosotros como instituto nos subimos al bus del MIAS y vamos a empezar a hacer el MIAS en la Guajira y eso es lo que estoy trabajando en este momento, en la propuesta y yo si le apuesto a todo y le apuesto porque el país lo necesita porque la salud de los Colombianos lo necesita y esto no puede seguir así, esto ya no aguanta más la población ya no aguanta más, más carruseles de la muerte  y cada vez se entera uno más de ciudadanos del común que le pasan cosas , pero ya es gente muy cercana que las que tienen problemas, entonces yo creo que si hacemos el trabajo bien hecho si realmente nos metemos y nos sentamos con la comunidad a hacerlo de una manera diferencial porque no es lo mismo un modelo de salud en Bogotá que un modelo de salud en la Alta Guajira, o un modelo en Leticia que en Medellín entonces yo sí creo que si lo hacemos con juicio y nos metemos región por región, esto puede ser algo que cambie la estructura, ojala … yo si le estoy apostando todo a este tema.

P10: 3. Transcripcion-Alexandra_Matallana_ok.docx - 10:24 [No eso lo veo difícil, no se d..]  (216:216)   (Super)
Códigos:	[modelo de salud futuro] 
No memos

No eso lo veo difícil, no se digamos que no estoy estudiando en estos momentos ese tema de como prevén ese pedazo porque en los territorios, digamos no en la metrópolis es un poco más fácil ese tema pues porque hay muchas regionales que tienen su masa crítica hay y el resto van desapareciendo por mercado, pero estas grandes me parece un tema complicado que no se el ministerio en que estará pensando, como va a ser para hacer ese descreme también, no sé cómo lo estará pensando eso es un vacío que tocara preguntarlo porque no sé.

P11: 4. Transcripcion Armando Gonzales_ok.docx - 11:6 [Cambiar no, se debe financiarl..]  (21:21)   (Super)
Códigos:	[modelo de salud futuro] 
No memos

Cambiar no, se debe financiarlo. La financiación tiene también sus cuestiones porque no todo es dinero, recuerde que cualquier dinero por abundante que sea  tiene que tener una efectividad desde el punto de vista de la administración, la administración debe ser efectiva y hablar de efectividad no es sencillo, definamos que es  efectividad, efectividad es que uno utilice los recursos adecuados para tratar una enfermedad cualquiera, eficacia es que se logre el resultado, que yo le di el tratamiento y logre el resultado y las dos multiplicadas o sumadas, da la efectividad de la atención médica, es decir producir los actos médicos con costos muy racionales, pero que permitan cumplir con el objetivo de mejorar al paciente, eso es en términos generales lo que es eficiencia, eficacia y efectividad. Cuando usted ve esto, usted ve que por ejemplo que pasa cuando a un paciente que tienen que suministrarle un medicamento que cuesta una vez o  tres veces más, usted le da el medicamento que cuesta tres veces más, y obtiene la misma eficacia, eso se vuelve totalmente inefectivo, entonces la mayoría de las administraciones de cualquier empresa están caracterizadas porque no son altamente efectivas, altamente productivos en ese sentido, el vuelco que hay que dar es hacia el enfoque sistémico de la atención medica como el enfoque de cualquier otra empresa, que permita claramente prestar un servicio con el menor costo posible pero con la eficacia y con efectividad, eso es cuando uno aplica siempre el enfoque sistémico que hemos aplicado siempre, es yo tengo 5 componentes, tengo 1 proveedor, tengo una entrada, tengo un proceso, tengo resultados y tengo un cliente que sale satisfecho, entonces cuando yo tengo esos 5 componentes y me sale el paciente contento al menor costo posible, yo estoy siendo efectivo. Vamos a mirar si la mayoría de las instituciones de salud, están cumpliendo con ese enfoque sistémico para lograr una mayor productividad.

P11: 4. Transcripcion Armando Gonzales_ok.docx - 11:8 [Las dos cosas que te he dicho,..]  (29:29)   (Super)
Códigos:	[modelo de salud futuro] 
No memos

Las dos cosas que te he dicho, primero financiación y segundo que se hiciera un gran trabajo para lograr la efectividad del desempeño en todo el sistema de salud, yo creo que sin esas dos cosas no es posible, la una es casi imposible, y la otra pues también, pero si es, si usted no logra esa efectividad en los modelos de prestación del servicio de salud siempre vamos a tener un déficit muy grande en la prestación de los servicios. 

P11: 4. Transcripcion Armando Gonzales_ok.docx - 11:15 [La atención primaria en salud ..]  (93:93)   (Super)
Códigos:	[modelo de salud futuro] 
No memos

La atención primaria en salud lo hace el sistema, eso es lo que se ha definido desde el año 1990 desde esa celebre reunión en Rusia que la principal estrategia era la atención primaria. Pero tiene que ser una atención primaria que cuente con la facilidad para hacer las referencias necesarias de acuerdo al diagnóstico que se  le haga a cada paciente pero ahí es donde viene la dificultad, tu puedes tener la atención primaria en un puesto de salud y como es tu sistema de referencia y contra referencia para que el paciente solucione esa situación, si especialmente es de escasos de recursos, si tiene seguridad social, y si no tiene seguridad social entonces ahí empieza a fallar todo porque que le prestas la atención primaria en salud que no puede emigrar a los otros niveles de atención para resolver su situación, y te dicen tienes una neumonía no te puedo mandar aquí te voy a mandar a otro lado, y empieza el paseo. Yo lo veo muy difícil así, por eso yo siempre insisto que se necesita más desarrollo para que esta cosa funcione como debe ser y todos los pasos sean los adecuados en el proceso de atención de un paciente. 

P12: 5.Transcripcion Carlos Fajardo_ok.docx - 12:7 [El modelo es muy importante in..]  (30:30)   (Super)
Códigos:	[modelo de salud futuro] 
No memos

El modelo es muy importante interesante teóricamente, pero si vamos a poblaciones dispersas o a poblaciones como la costa pacífica, donde toda la población no está concentrada si no que existen poblados distantes no solamente por vía terrestre sino también por vía marítima, de dos a dieciséis horas, entonces no veo cómo se va a poder integrar cuando allá ni siquiera existe un servicio de salud, es muy difícil que el MIAS funciones con una población dispersa, posiblemente funcione en poblaciones donde haya mayor concentración por ejemplo Tumaco, podrá pero si tiene un buen sistema concerniente a lo que es el primer nivel, donde las actividades o las puertas de entrada de las enfermedades generales ahí se pueda captar y ahí se pueda hacer la mayoría de sus actividades de mejora de la salud, hoy que pasa, toda la población llega al primer nivel y eso es, remítala al segundo, entonces del primer nivel es casi nada lo que hacen, entonces se cápita se paga, pero se remite para un segundo tercer nivel.

P12: 5.Transcripcion Carlos Fajardo_ok.docx - 12:10 [reo que tiene que haber una ap..]  (42:42)   (Super)
Códigos:	[modelo de salud futuro] 
No memos

reo que tiene que haber una apertura mayor a la  libre oferta, para que las instituciones públicas, yo considero que en el sistema se les ha dado mucha importancia y demasiado proteccionismo en las instituciones públicas, pero no ha habido exigencias les permitan desarrollarse como tal, les han dado las herramientas pero lastimosamente por su formación burocrática o su formación política o su formación de intereses no le permiten que se desarrollen. 

P12: 5.Transcripcion Carlos Fajardo_ok.docx - 12:12 [Uno dice siempre cuándo va a t..]  (50:50)   (Super)
Códigos:	[modelo de salud futuro] 
No memos

Uno dice siempre cuándo va a todos los foros, los sistemas en el mundo y los sistemas de salud son muy complejos son de difícil manejo. Una de las condiciones que uno está mirando es que la población en Colombia en veinte años, va a tener una carga bastante fuerte de enfermedad  y van a ser las crónicas las enfermedades dominantes volviendo más costoso el sistema, entonces si el sistema en lo que respecta a la financiación continua igual, uno diría que en veinte años el sistema habrá colapsado, habrá que tener otro sistema o netamente privado, o netamente público. Netamente privado donde suceda como en Estados Unidos, que las personas que tienen plata son las que tienen acceso a salud y los que no tendrán unos limitantes como teníamos antes de la ley 100, donde las personas que no tenían plata pues tenían que vivir, de la caridad para curarse de una enfermedad, o de lo que el estado le pudiera brindar. Entonces yo lo que considero que, si no hay esos cambios del manejo de la población hacia la promoción y prevención, el sistema actual hoy es muy asistencialista, muy dado a curar a la enfermedad y muy poco hacia la promoción y la prevención de la enfermedad. Si no se cambia esas dos variables de la población y de la promoción y si no se da una buena atención pues el sistema volverá a ser de carácter público o privado, para que cada quien se pague hasta donde tenga sus capacidades. Lo cual sería grave porque sería un retroceso bastante grande.

P12: 5.Transcripcion Carlos Fajardo_ok.docx - 12:13 [Para mí el sistema preferido e..]  (54:54)   (Super)
Códigos:	[modelo de salud futuro] 
No memos

Para mí el sistema preferido es el que tenemos actualmente, el de aseguramiento con unas fuentes de financiación adecuadas, que estén dadas por dos condiciones, una un financiamiento que tenga el estado pero también que haya un financiamiento por parte del individuo, en las medidas de sus capacidades, el contributivo tiene a través de los pagos a través de las cuotas moderadoras, un financiamiento total, pero en el subsidiado no, es totalmente financiando por el estado. Hay muchas actividades del subsidiado que son exclusiones que no deberían ser financiadas por el estado, porque ahí entraría en esta parte a que haya verdaderamente una respuesta adecuada a esas necesidades. Y la la  parte corresponde al financiamiento y que este centralizado en su fuente  y la estrategia de atención sea la de promoción y prevención, porque si seguimos en un sistema existencialista morimos.


P12: 5.Transcripcion Carlos Fajardo_ok.docx - 12:16 [Si porque en esta situación de..]  (66:66)   (Super)
Códigos:	[modelo de salud futuro] [Protección financiera : financiación del SGSSS] 
No memos

Si porque en esta situación de tipo financiero no todas  van a poder cumplir sus estándares, o no van a poder lograr llenar el defecto que se calculó a diciembre de 2015 y que hay que cumplirlo en los sietes años. Hay bancos que no van a prestarles  a las EPS, entonces yo considero sobre todo que esas empresas pequeñas les va a ser muy difícil el cumplimiento, porque hoy para poder tener unos márgenes suficientes, y poder manejar las inestabilidades debe haber una gran población, si no es muy difícil con poblaciones pequeñas.

P12: 5.Transcripcion Carlos Fajardo_ok.docx - 12:27 [El gobierno hoy tiene toda la ..]  (106:106)   (Super)
Códigos:	[modelo de salud futuro] 
No memos

El gobierno hoy tiene toda la intención de poder prevenir lo que es el crecimiento exponencial que se está generando en las enfermedades crónicas, se sigan manifestando. Por eso esta hablando hoy del modelo del MIAS y de fortalecer todas las secciones preventivas que es donde el gobierno hoy tiene como ese enfoque, porque la pirámide hoy está al revés, todo lo queremos manejar desde la alta complejidad hasta una gripa la quieren manejar allá, un dolor de cabeza lo queremos manejar con el especialista, entonces no hay integralidad en atención como tal de la persona. Entonces el gobierno hoy está planteando que con el nuevo enfoque y con el manejo de ciertos modelos de riesgos en salud que existen como la diabetes, la hipertensión las cardiovasculares, la mortalidad infantil, la maternas, se dé un mejor tratamiento y que eso va a permitir que haya un cambio verdadero, eso se viene haciendo en el momento. 

P12: 5.Transcripcion Carlos Fajardo_ok.docx - 12:28 [Si se articula bien que es com..]  (110:110)   (Super)
Códigos:	[modelo de salud futuro] 
No memos

Si se articula bien que es como modelo que hoy está haciendo por ejemplo Bogotá que está articulando buscando que en el primer nivel se hagan nodos, entonces ya no existan 50 hospitales de primer nivel si no que sea un nudo norte, un nudo sur, este. Entonces eso va a permitir reducción de costos, pero igualmente las puertas de entradas esencialmente para poder proteger a la población en lo que concierne a las enfermedades que son precursoras. Entonces ahí es donde tiene que detectar si hay un cáncer incipiente, o si hay una diabetes incipiente o si hay hipertensión incipiente, porque ahí es lo que está pasando hoy, todo lo estamos llevando al segundo nivel. Seguimos en el mismo modelo y eso va a afectar, entonces yo diría que ese segundo modelo que esta presentando el gobierno de que haya una red de atención adecuada pero que se tenga una responsabilidad en cada una de ellas va a permitir mejorar el servicio. Que haya un primer nivel pero que sea resolutivo, porque hoy el primer nivel es el médico general dedicado a tramitar remisiones. Si ese primer nivel como está planteado en el MIAS es resolutivo y se extiende por todo el país eso funciona.

P12: 5.Transcripcion Carlos Fajardo_ok.docx - 12:29 [Hoy el gobierno está muy enfoc..]  (114:114)   (Super)
Códigos:	[modelo de salud futuro] 
No memos

 Hoy el gobierno está muy enfocado en eso, la implementación como tal porque en esto hay muchos intereses de diversas índoles, hay los intereses de las aseguradoras, de los prestadores públicos, del prestador privado y el interés del público en general. Entonces yo creo que mientras se articule todos estos intereses, en un promedio de 5 años esto estará funcionando, no antes. 

P12: 5.Transcripcion Carlos Fajardo_ok.docx - 12:30 [Yo creo que lo que plantee ant..]  (118:118)   (Super)
Códigos:	[modelo de salud futuro] 
No memos

Yo creo que lo que plantee anteriormente, la dispersión geográfica esa es una de las principales barreras de acceso sobre todo en regiones como  la costa pacífica, o en los sitios de la selva la Orinoquia, o en los lados de putumayo en la selva amazónica. Entonces el poder tener centro de atención, hoy no existen en esas ciudades en esas zonas, que garanticen la accesibilidad y el aseguramiento, uno porque no hay medios de transporte, dos porque no hay instituciones cercanas donde puedan ser atendidas las personas y tres porque hay mucho de los profesionales que no se arriesgan a ir a esas zonas. Entonces el acceso en total se puede decir que es mínimo, hay acceso cuando la persona sale por complicaciones muy difíciles de manejar y por eso acceden ya a los niveles de alta complejidades que son las zonas urbanas.

P12: 5.Transcripcion Carlos Fajardo_ok.docx - 12:19 [Yo estoy muy consiente que las..]  (145:145)   (Super)
Códigos:	[modelo de salud futuro] 
No memos

 Yo estoy muy consiente que las organizaciones y sobre todo las EPS tienen unos retos muy importante para poder visualizarse o para poder sostenerse en el tiempo y para poder trascender hacia los 20 años que es lo que queremos. Uno tienen que generar nuevas formas de administración en lo que corresponde a la gestión de riesgo, eso llevara  a que hay que cambiar las formas de contratación, hay que cambiar las formas de atención, que hay que articular la red, primaria con la red secundaria y que haya una articulación y que haya una red verdaderamente y que cumpla y que supla las condiciones básicas de una población y sea complementaria, porque hoy tenemos muy atomizado entonces una persona entro a una urgencia y ahí le dicen tenemos que tomarle la radiografía pero esa radiografía no se la tomamos aquí si no váyase a tal parte y de allá vengase de tal parte, entonces es un rodeo cuando debería ser una institución donde tenga sus examenes ahí tenga sus rayos x, su integralidad. Entonces en esa parte hay que cambiar y creo que para alla  vamos. Dos que las EPS, en el contributivo se vio mucho y para mi fue un factor de éxito que tengan su propio manejo de lo que es la atención primaria, que corresponde a toda la promoción y prevención que hoy lo tiene el contributivo como tal, pero que tengan instituciones desarrolladas para el acceso a través de la 1438 se dijo no y se puso unos limitantes, que máximo el 30% podrá ser integración vertical y se descartó la integración vertical, bueno si no se puede integración vertical entonces porque se permite que haya una Imbanaco una valle del Lili, cuando hay unas inversiones grandísimas en capital privado, y eso ha sido a través del mismo sistema que ha provisto para que se generen esas instituciones. Entonces las EPS deberían tenerlo y esa es una de las visiones que tengo. Un modelo que le permita también articular toda su población para poder ser atendida por ellas mismos, y no hoy o con ciertas instituciones que le garanticen integralidad en la atención, pero que es lo que nos dice la norma contrate con la red pública el 60% pero la red pública no me está siendo resolutiva entonces quedo en el peor de los mundos. Le pago pero no me da resultados, entonces si no puedo manejar esos directamente en aquellas partes donde veo que realmente no funciona sobre todo en esas zonas dispersas donde nadie le quiere meter plata ni nadie le quiere invertir, creo que eso mejoraría eso. Lo otro es el fortalecimiento financiero dentro de los 7 años y lo que se busca es que las EPS como tal como aseguradoras puedan tener una mejor sostenibilidad y un mejor soporte financiero que le permita garantizar y generar confianza en el sector, porque podemos decir que el sector tiene muchas desconfianzas, las EPS cogemos de las IPS, las IPS desconfiamos de las EPS, del estado entonces no hay factores de confianza que permitan generar verdaderos momentos en concertar en lograr mayores impactos en los desarrollos del sistema, entonces yo creo que más vamos para esa parte considero que es lo que uno buscaría dentro de este modelo


P13: 6 y 7.Transcripcion Diego Gomes y Gonzalo Gonzales_ok.docx - 13:7 [Yo creo que el futuro va a ser..]  (19:19)   (Super)
Códigos:	[modelo de salud futuro] 
No memos

Yo creo que el futuro va a ser parecido al de los otros países, salud nunca va a tener los recursos suficientes para dar todo de manera ilimitada, entonces el sector salud va a tener que controlar costos, a través de proveedores tal vez a través de usuario, en fin. Pero el futuro va a ser donde vamos a dar cuenta de lo que nos ha servido al sistema cuando no lo tengamos, eso es como el dolor. El sistema va a seguir teniendo problemas, se va a ir ajustando va a haber mucha presión para regresar a modelos de oferta de servicios, no de demanda diría yo, yo creo que en la actualidad se han venido logrando algunos equilibrios, pero hay que ir tomando medidas en medida de que se va prestando, porque el sistema se acomoda, usted regula un medicamento, entonces por otro lado le buscan la forma de cobrar más por la prescripción y se va a haciendo un sistema porque maneja tanto dinero y  hay mucho interés, entonces es un sistema así como la comida, el vestido, lo que todos necesitamos. El sistema no se va a acabar, se va a mantener pero se va a ir perfeccionando: 

P13: 6 y 7.Transcripcion Diego Gomes y Gonzalo Gonzales_ok.docx - 13:8 [Los diseñadores de política no..]  (21:21)   (Super)
Códigos:	[modelo de salud futuro] 
No memos

Los diseñadores de política no tienen mucho para dónde coger en diseño de sistemas, entonces el sistema al que están optando los países del mundo son los sistemas de aseguramiento, los sistemas de salud pública pagados por el estado están haciendo unas enormes crisis y tienen menores indicies de servicio, eficiencia y viabilidad, que los sistemas de aseguramiento. Los sistemas de aseguramiento como el de Alemania, o como suiza están marchando bien y Colombia es un sistema de aseguramiento que ha logrado generar atenciones a una fracción del costo, de los que encuentran los modelos de aseguramiento del mundo, es decir es más barato que Alemania o que Suiza, pero incluso mucho más barato que cualquiera de las pólizas privadas en Estados Unidos para dar otro ejemplo. Entonces no hay mucha opción en esa vía, se va a tener que consolidar el aseguramiento, puede tener dos posibilidades, una es de póliza abierta, otra de póliza regulada, nosotros tenemos un aseguramiento con póliza regulada, tampoco hay mucha opción para el país de optar por póliza abierta, o por póliza regulada, vamos a seguir con una póliza regulada por la UPC porque a otra cosa no se llega fácil desde el estado, esa póliza regulada puede tener unos incrementos, pero esos incrementos van a ser marginales nunca va a ser incremento del 20%, al contrario van a ser incrementos del uno o dos por ciento , del valor de la UPC de salud. Ese orden de cosas de que no y para donde coger por más de que haya presiones, o discusiones cualquier estudioso, técnico de salud, sabe que no hay otra opción para dónde coger, que estemos sobre el camino que es. Ahora, entonces no queda otra que es este camino que estamos recogiendo consolidarlo adecuadamente, ese camino de consolidación adecuada va a evolucionar a unos modelos de atención y modelos de gestión muy centrados en los resultados efectivos del paciente, con un costo razonable en el cual los riesgos son compartidos por todos. En este momento tenemos un modelo que lo único que corre el riesgo hoy es la EPS y ese modelo de que la EPS corre riesgo, lo que se ha llevado es a un acercamiento o una posición abusiva de los prestadores en el cobro por evento, se van a tarifas propias, se van a tarifas que ellos arbitrariamente imponen o a negociaciones extremadamente costosas que terminan incluso en vías judiciales, como la tutela. Lo que vamos  es a una autorregulación del sistema en el que van a empezar a aparecer agentes, que van a decir que asumen el riesgo de autorregular su costo para que sea viable, ese fenómeno de aparición de agentes que no van a cobrar por eventos, si no que van a cobrar integralmente asumiendo el riesgo y la responsabilidad de regular el costo, va a empezar a romper el mercado  va a empezar a generar unos modelos de autorregulación muy importantes en el sistema, yo creo que el modelo en el sistema se va  a consolidar y en 10 años vamos a estar diciendo, esta es la gran construcción de equidad social de Colombia como ya lo es de hecho en términos de atención, lo será pero ya en términos de que sea una construcción viable, muchos agentes van a tener que transformarse de lo que realmente son, no van a poder seguir siendo esos hospitales individuales, arrogantes que cobran unas tarifas abusivas al sistema si no unas redes integrales de atención, mucho más evolucionadas, mucho más juiciosas mucho más centradas en el paciente y vamos a seguir consolidando el sistema, se va a consolidar. 

P13: 6 y 7.Transcripcion Diego Gomes y Gonzalo Gonzales_ok.docx - 13:9 [Para completar algo que dice D..]  (23:23)   (Super)
Códigos:	[modelo de salud futuro] 
No memos

Para completar algo que dice Diego que me parece interesante, hay un estudio que hizo la universidad de Antioquia, el costo promedio en Colombia por persona es de 843 dólares, y en los países de cooperación por el desarrollo económico que son 22 países, el costo es de 3.405 dólares por persona, 4 veces menos que los otros países y tenemos cobertura plena, cosa que no ocurre en otros países, cosa que viene a otro tema. 

P13: 6 y 7.Transcripcion Diego Gomes y Gonzalo Gonzales_ok.docx - 13:10 [Cuando uno mira el sistema des..]  (31:31)   (Super)
Códigos:	[modelo de salud futuro] 
No memos

 Cuando uno mira el sistema desde el servicio de emergencia, uno mira que desde los 60 en el mundo los servicios de urgencia se vienen saturando, y eso le está ocurriendo a Colombia desde que yo Salí de medicina en el año 70, lo que uno ve ahí es que básicamente eso es una expresión no solamente local si no mundial, y la única respuesta que uno tiene es que es un modelo de atención que está llevando a eso, cuando uno mira el modelo de atención uno descubre que está fundamentado en 3 grandes pilares de alto costo. El pilar de especialistas, el pilar de medicamentos y el pilar de ayudas diagnósticas, todos esos 3 pilares han llevado a un modelo altamente costoso y no necesariamente más eficiente que un modelo donde los que trabajamos en una época como médicos generales, veíamos que funcionaba y que resolvía el 98% de los problemas médicos, entonces yo pienso que el sistema en todo el mundo y en Colombia sino modifica su modelo de atención medica, en el mundo no hay capacidad para resolver un problema de salud, donde realmente un médico familiar es el que centra toda la atención con el apoyo a los demás, no al contrario. Mientras no hagamos eso el sistema no va a ser viable. 

P13: 6 y 7.Transcripcion Diego Gomes y Gonzalo Gonzales_ok.docx - 13:11 [consolidación de un sistema pa..]  (33:33)   (Super)
Códigos:	[modelo de salud futuro] 
No memos

consolidación de un sistema para conservar la salud, para mantenerse feliz, generador de valores, generador de integración en la sociedad, que superemos un modelo que es de atención de la enfermedad, que lleguemos a un sistema donde el medico sea la última instancia y la forma de vida sea la primera instancia, y en esa forma de vida el medico ya no va a ser para curarle la enfermedad, si no para que usted cuide su salud, y ese es un sistema de salud centrado en mantener una población saludable, creo que debemos evolucionar a una sociedad que mire la salud de una manera más proactiva y más aceptando la dolencia, la enfermedad, natural y aceptando la muerte como algo totalmente natural, esperaría que lleguemos a esta decrépitamente aferrados a la vida sino aprender un poco de los elefantes y saber que también hay un tiempo para morir y un tiempo para ya entregarse a la naturaleza. Con la decrepitud que estamos teniendo hoy aferrándonos estamos es cada uno de nosotros, aplicando unos costos exagerados a la sociedad para tratar de salvar lo insalvable y atender lo no atendible, es aprender a vivir plenamente,  la vida, hasta que llegue el momento de ya no estar más.

P13: 6 y 7.Transcripcion Diego Gomes y Gonzalo Gonzales_ok.docx - 13:13 [El tema viene de la definición..]  (83:83)   (Super)
Códigos:	[modelo de salud futuro] 
No memos

El tema viene de la definición de que es salud, no hay un acuerdo mundial sobre que es salud y va a haber menos acuerdos de cuál es el derecho a la salud porque es muy individual, ósea para unas personas tener un ojo caído es algo natural, pero para otras no y entonces ahí comienza el concepto individual. Entonces yo pienso que el concepto de salud es un concepto que hay que manejarlo de tipo colectivo y de tipo individual, de tipo colectivo hay que pensar en la igualdad y para la igualdad no se puede gastar mucho en uno que no se justifique, y en otros dejarlo porque  estamos compitiendo por recursos. Entonces yo no me atrevería a decir, yo te podría recetar acá la definición de salud de la OMS pero los académicos los criticamos por X o Y valor, pero para mí es muy importante tenerla porque es una meta a lograr, sabiendo que llegar a eso es casi imposible, pero saber buscarla nos puede poner de acuerdo en el trabajo de la búsqueda de la salud.


P13: 6 y 7.Transcripcion Diego Gomes y Gonzalo Gonzales_ok.docx - 13:14 [Lo que no se da cuenta la gent..]  (93:93)   (Super)
Códigos:	[modelo de salud futuro] 
No memos

Lo que no se da cuenta la gente, es que el estado va a hacer eso a costa de sus propios recursos, el estado va a hacer eso como un cargo ineludible que se va a financiar de la extracción del estado de estos recursos, y ese comportamiento de tratar de hacer salud sin construir salud desde la propia gente, si no hacer salud a punta de medidas externas y no con una construcción de un comportamiento saludable, lo que va a generar es un costo que cada vez pasara  a ser mayores y entonces todos los individuos van a ver como se recortan las posibilidades para muchas más cosas, a costa de tener que pagar un sistema de salud que por su propia construcción y por su propia génesis es mucho más costosa que si todos partiéramos de que construimos un sistema de salud desde los deberes y no desde los derechos, ese es el gran error del sistema

P13: 6 y 7.Transcripcion Diego Gomes y Gonzalo Gonzales_ok.docx - 13:15 [Yo creo que la pregunta está m..]  (97:97)   (Super)
Códigos:	[modelo de salud futuro] 
No memos

Yo creo que la pregunta está mal hecha porque la salud no es un tema de se tiene o no se tiene salud, todos estamos parcialmente sanos, o parcialmente enfermos si nos vamos a un momento dado, entonces cuando uno plantea esa pregunta es, oiga es que todo el mundo tiene que tener salud, y resulta que todos tenemos salud y no salud simultáneamente, entones lo que va a darse la discusión es en cada individuo, oiga cual es la salud que usted cree que debe tener, entonces es una discusión que se va a tener colectiva y no es un problema que en 20 años vamos a atender, hay 3 cosas que en la vida el hombre da prioridad, primero su vida, segundo su parte religiosa y tercero a sus bienes, entonces ahí está el sector salud médico, el sacerdote y el abogado, esas son las prioridades que las personas consientes o inconscientemente manejan y ahora el economista con el abogado. Si uno mira salud dentro de esos conceptos, un dice claro todas personas que están intentando proteger su vida pasa por la salud, el tema es ¿qué es salud? para cada uno, que lo vimos ahora, hay que ponerlo como una meta pero sabemos que individualmente no hay necesariamente un acuerdo entre lo que es salud para usted o para mí, y cuando yo estoy protegiendo mi vida yo voy a buscar que se haga lo máximo para que mi vida se proteja, y ahí viene otro concepto y es que una cosa es prolongar la vida mediante los años o agregarle vida a los años que un concepto que manejamos, no basta decir viva usted más si no viva más pero con buena calidad de vida, y empieza la discusión, entonces a mí me parece esas preguntas si se van a garantizar el derecho a la salud en 20 años,  el derecho se va a garantizar, pero con las discusiones que se han ido evolucionando en 20 años al respecto. 

P13: 6 y 7.Transcripcion Diego Gomes y Gonzalo Gonzales_ok.docx - 13:16 [Yo pienso que sí, porque no ha..]  (101:101)   (Super)
Códigos:	[modelo de salud futuro] 
No memos

Yo pienso que sí, porque no hay que mirarlo solamente ahí, se soporta en una medicina familiar, o referenciada, donde una persona un médico y tal vez con su equipo va a mirar que le está ocurriendo a ese núcleo familiar, eso tiene el supuesto que atravesó del grupo familiar se van a coger los factores de riesgo primarios, la prevención y promoción, porque la familia vive en un entorno porque tiene una genética, tiene un estilo de vida. Segundo ese médico general va a ser como la torre de control de manejo suyo, ya no se tira a arriba donde un especialista entonces el especialista ve un pedacito y le pide todos los examemes que considera conveniente y después el paciente queda a su suerte a ver qué es lo que ocurre, eso es como pasar por un campo cuando empieza a llover, ver cuantas gotas de mala salud le cae a usted, cuando nosotros tenemos un médico familiar y con atención primaria en salud renovada, nosotros vamos a poder tener control inclusive en el gasto de salud y en la prevención y promoción de la salud, lo que llaman riesgo primario y vamos a poder tener control en el riesgo secundario. Yo pienso que debemos considerar un fracaso la hospitalización e inclusive llegar a los especialistas, si el médico general está bien capacitado puede disminuir mucho el tener que llegar a los especialistas y mucho tener que llegar a hospitalización.

P13: 6 y 7.Transcripcion Diego Gomes y Gonzalo Gonzales_ok.docx - 13:17 [Yo creo que no menos de 20 año..]  (105:105)   (Super)
Códigos:	[modelo de salud futuro] 
No memos

Yo creo que no menos de 20 años, porque no solamente es decir yo quiero esto, si no que el modelo hay que construirlo con hombres con personas, y lo que uno ve es que los que estaban formados en el siglo pasado tenían más enfoque en esta característica y lo mismos médicos tienen una ingerencia muy grande en la especialización, antes se hacía el diagnostico fundamentado en signos y síntomas, ahora el diagnostico se hace con pruebas diagnósticas, antes se hacía el diagnostico con signos y síntomas y se comprobaba con las ayudas diagnosticas ahora es a la inversa, son procedimientos que son muy costosos y que no siempre son mejores, porque cada vez que usted hace una prueba va aumentando el riesgo de falsos positivos, y al tener falsos positivos también puede tener un problema de salud, entonces lo que yo creo que hay es que cambiar toda una cultura y yo creo que eso no se da antes de 20 años, si se tuviera la gobernabilidad, y también lo voy a dudar porque la presión de los especialistas la presión de los suministros laboratorios, es muy grande y el sistema actual para ellos es más rentable, no para el sistema de salud entonces hacer ese cambio implica todo un cambio de cultura no solamente del sector sino de toda la sociedad, es reconocer que nosotros cuando gastamos 5 pesos acá, se lo estamos quitando a otro sector si nosotros aumentamos de forma ilimitada el gasto en salud se lo estamos quitando a educación y a otras cosas de bienestar, entonces ahí es cuando viene el tema que la igualdad hay que mirarla así. 

P13: 6 y 7.Transcripcion Diego Gomes y Gonzalo Gonzales_ok.docx - 13:18 [Es un cambio adecuado, yo creo..]  (109:109)   (Super)
Códigos:	[modelo de salud futuro] 
No memos

Es un cambio adecuado, yo creo que en un 50 o 60% antes de cuatro años, y apenas se pase una masa crítica inmediatamente se va a convertir en regla de operación.

P13: 6 y 7.Transcripcion Diego Gomes y Gonzalo Gonzales_ok.docx - 13:20 [Yo sí creo que hay barreras de..]  (119:119)   (Super)
Códigos:	[modelo de salud futuro] 
No memos

Yo sí creo que hay barreras de salud, cuando uno ve que hay pacientes que no pueden ingresar por ejemplo por urgencias porque no hay camas hospitalarias, y que empiezan a buscar por un sitio porque no se atendieron adecuadamente, una atención primaria. En el fondo el sistema tiene barreras concretas de acceso, yo pienso que si el modelo MIAS se monta, esas barreras van a disminuir, porque la puerta de entrada va a ser su médico familiar, va  a ser el abogado dentro del sistema, pero barreras las hay y siempre van a haber barreras porque siempre vamos a tener el tema de costos vs atención, pero yo diría que el sistema en la medida que tenga una forma racional un modelo de atención en salud, un modelo medico en atención en salud racional van a disminuir esas barreras, hay unas barreras culturales indudablemente, yo por lo menos tengo barreras culturales porque no hago uso del sistema, uno como médico tiene su relaciones, pero me preguntan a mi como entro yo al sistema, casi siempre por automedicación o porque el amigo me dice tal cosa, pero si me toca ingresar al sistema tengo desconocimiento en muchas cosa que la gente tiene que hacer y que está haciendo. Entonces yo pienso que todos esos procesos hay que simplificarlos, y en la medida que este el médico general manejando el paciente como una torre de control ya eso es un problema de conocimiento no está en mis manos, si no en el medico general y pasa por un sistema de información, otro de los grandes problemas que hay en el acceso es el sistema de información y si tuviéremos un sistema de información único, donde lo que me ve este médico aparece en mi historia médica y lo ve este otro y el otro tiene que creer en los exámenes que me hicieron acá, no solamente disminuiríamos los costos si no que no pondríamos a circular los pacientes a pedir un examen aquí y otro allá si no que se harían atencionespor integrales, el sistema tiene barreras.

P13: 6 y 7.Transcripcion Diego Gomes y Gonzalo Gonzales_ok.docx - 13:21 [Yo pienso que si tienen que di..]  (123:123)   (Super)
Códigos:	[modelo de salud futuro] 
No memos

Yo pienso que si tienen que disminuir, porque si el modelo se implanta va a haber una disminución, en otras palabras si yo tengo una amigdalitis para que me mandan a un otorrino si el médico general en el cual yo confió me la trata y al otro día estoy mejor. Ahí hay un problema bien grande a los 20 años y es que hay que reformar la formación en medicina que nosotros tenemos para que se ajuste a un modelo de estas características

P13: 6 y 7.Transcripcion Diego Gomes y Gonzalo Gonzales_ok.docx - 13:22 [En el MIAS aparece algo muy in..]  (127:127)   (Super)
Códigos:	[modelo de salud futuro] 
No memos

En el MIAS  aparece algo muy interesante y es que hay zonas del país que hay que manejar de forma distinta, cuando uno mira poblaciones como por ejemplo choco, o Quibdó, uno descubre que no es que hayan tenido recursos, si no que los recursos en mi concepto no se han utilizado correctamente, y utilizado adecuadamente no por corrupción sino también por modelo, me tocó trabajar en choco en atención primaria salud con modelos que ustedes tenían de Cinder con conductores de salud, con los holandeses allá y uno veía la transformación de la comunidad como con la atención primaria, se disminuía la mortalidad en niños, como se vacunaban, como se manejaba el saneamiento, y yo decía esto pues es un abismo y la pregunta es porque no lo podemos hacer nosotros en estas comunidades que lo requieren, usted tiene por lo menos en el valle de aburra, agua potable pero con toda la inversión que se ha hecho porque esa agua potable no funciona por ejemplo cuando la epidemia de cólera que me toco ayudarle al choco, y fuimos a aterrizar a rio sucio y vi un gran tanque de agua, aterrizamos y pregunto y abro la llave para lavarme las manos, para ver los pacientes que tenía y me encuentro que el acueducto estaba hecho pero se habían llevado las bombas, todavía no había funcionado un minuto y se habían robado las bombas, entonces uno pregunta el tema no es solo de recurso es tema de cultura, porque en el fondo es una cultura de manejo, estas comunidades requieren una atención distinta, yo pienso que tenemos que tener la capacidad dentro del sistema de casi manejarlo por oferta, mientras el MIAS madura y tiene una capacidad también de adaptación para que puedan hacerlo. Ósea el modelo no puede ser igual en todas partes, tiene que ser adaptado en las necesidades que tiene cada comunida

P13: 6 y 7.Transcripcion Diego Gomes y Gonzalo Gonzales_ok.docx - 13:24 [el dato que acá yo tengo no es..]  (135:135)   (Super)
Códigos:	[modelo de salud futuro] 
No memos

el dato que acá yo tengo no es de 5.9, es de 8.5 billones, a su vez las EPS tienen unos cuantos millones por cobrar a el estado de deudas que el estado le tienen a las EPS, pero todo eso en conjunto es un costo mayor de la salud que el que podríamos atender, una parte muy importante de ese costo que tienen muchos hospitales fue producido por tarifas propiciadas por eventos no POS que no eran justificados, o fueron producidos por medicamentos de alto costo que tenían un medicamento genético, entonces yo pienso que es un problema sistémico, muy grave en el cual las IPS tienen una parte muy delicada de responsabilidad, los jueces con sus tutelas tienen también una parte delicada de responsabilidad, y las EPS con sus malos modelos de gestión y sus prácticas corruptas, también tienen un grado muy delicado de responsabilidad.

P13: 6 y 7.Transcripcion Diego Gomes y Gonzalo Gonzales_ok.docx - 13:23 [Yo pienso que al sistema hay q..]  (135:135)   (Super)
Códigos:	[modelo de salud futuro] 
No memos

Yo pienso que al sistema hay que viabilizarlo, hay que hacer un aclaramiento de las citas, hay que inyectarla al país un flujo de caja y un aclaramiento de cuentas muy importante, eso puede no llegar a ocurrir, de eso no llegar a ocurrir y no darse ese aclaramiento de cifras, lo que va a terminar ocurriendo es que los hospitales van a tener que asumir esas deudas, las EPS efectivamente van a desaparecer y lo que vamos a ver es una rebaja real de los costos, de los hospitales vía producción de todo estos.

P13: 6 y 7.Transcripcion Diego Gomes y Gonzalo Gonzales_ok.docx - 13:25 [LA salud es como la comida, si..]  (139:139)   (Super)
Códigos:	[modelo de salud futuro] 
No memos

LA salud es como la comida, siempre la tenemos que tener y siempre vamos a buscar la forma de tenerla, de manera que hay que hacerlo. Yo pienso que el sistema está en desequilibrio por todo lo que se ha comentado pero hay también un responsable que no aparece, el sistema como sistema tiene una caja que se mueve y la quiebra de EPS en la cual el gobierno ha sido el socio mayoritario, ha recaído sobre esas instituciones, caprecom por ejemplo, quien paga eso pues las IPS, entonces ellas tienen que subir sus tarifas, para defenderse entonces los bancos le prestan a las IPS que hay que pagar intereses y eso vuelve a subir las tarifas, entonces eso es un espiral que se desencadena y alguien tiene que pararla, habrá una forma y es que se quiebre, entonces vienen unas nuevas, y los que están abajo van a financiera el sistema. En 20 años tenemos que resolver ese problema es como las contabilidades cuando están atrasadas, uno puede tomar la acción de ir hacia atrás hasta que se encuentre con el hoy, es una mala política, o desde hoy voy a actualizarlo  y más bien hacia otra poco a poco me voy actualizando, eso le va a pasar al sistema, el sistema tiene que poner un hoy, y tal vez irse para atrás lentamente para actualizar esas deuda.

P13: 6 y 7.Transcripcion Diego Gomes y Gonzalo Gonzales_ok.docx - 13:26 [Yo soy optimista con la salud ..]  (143:143)   (Super)
Códigos:	[modelo de salud futuro] 
No memos

Yo soy optimista con la salud en Colombia me parece que Colombia cuando se muestra hacia atrás que me toco todo el sistema años atrás, si no con otros países, Colombia está en mejor estado lo que si tenemos que hacer es un modelo más racional. Ósea el modelo debe tener un pensamiento más racional y creo que los MIAS pueden ser el camino, donde la racionalidad en los servicios de salud partiendo de la promoción y prevención, la atención primaria en salud a través de médicos familiares que sean quienes manejen las personas va a ser el futuro, pero como dije eso requiere un tiempo, el recurso humano hay que fundarlo con tiempo, hay que desarrollar la cultura dentro del sistema y se puede llevar a tener un sistema relativamente sostenible.

P13: 6 y 7.Transcripcion Diego Gomes y Gonzalo Gonzales_ok.docx - 13:27 [te lo digo en un altísimo porc..]  (151:151)   (Super)
Códigos:	[modelo de salud futuro] 
No memos

te lo digo en un altísimo porcentaje si, te lo digo por una EPS, que puede servir de modelo de revisiones. Hay una EPS que para nuestro efecto es un experimento controlado, un experimento donde puede aislar los demás efectos, y solo observar unos efectos específicos, si pudiéramos tener una EPS de la cual pudiéramos aislar los efectos de corrupción y solo nos podemos concentrar en los estrictos efectos de costo, y modelo de atención, si podemos tener una EPS donde además podamos tener efecto pasado, si no efecto presente, si podemos tener una EPS donde digamos se pusieron lo mejor de lo mejor para que la manejara, y si decimos que esa EPS tiene un hueco de 300.000 millones de pesos después de 3 años de operar y teniendo todos los datos porque estamos haciendo el plan de rescate. Entonces tú dices, todo lo que salga es por cuentas médicas, y en esas cuentas medicas hay unos patines que un juez obligo porque supuestamente al niño le mejoraba la forma de los pies si tenía patines de línea, y le obligaron al sistema para esos patines del niño. Y cantidad más de tutelas exóticas, además están las cuentas enorme de muchos hospitales departamentales ESE, ósea entidades sociales del estado que le cobraban y le siguen cobrando a tarifa soat o tarifa propia, y excelentes hospitales, los mejores hospitales del país o de los mejores hospitales del país a los cuales Savia le adeuda una enorme plata y ahora savia es la gran criminal porque les adeudan muchísimo dinero a ellos pero no son criminales por haberle cobrado a Savia tarifa propia, y tarifa soat, uno de los gerentes de esos hospitales dijo cuándo le recriminaban que porque cobraba acetaminofén a 1.500 pesos la tableta, cuando le costaba menos de 50 pesos, es que nuestro hospital es de 5 estrellas, es que no hay hospital de 5 estrellas, hay salud y hay costos de la salud, si usted quiere cobrar las 5 estrellas cóbrelas en el sistema privado, peor no en un sistema público de salud, entonces te lo digo, la razón de la quiebra de Savia son todo el sistema de hospitales. Entonces ese experimento controlado te lo dice así claramente son los hospitales los responsables del sistema de salud.


P14: 8. Transcripcion Jorge E. Robledo_ok.docx - 14:3 [No, yo creo que es clarísimo q..]  (11:11)   (Super)
Códigos:	[modelo de salud futuro] 
No memos

No, yo creo que es clarísimo que las cosas del sistema de salud se van a empeorar, en el corto plazo es evidente, va a aumentar el desempleo y va a aumentar la pobreza, pero en los próximos 4 – 5 años las cosas se van a empeorar, porque paso la bonanza minera no se hizo lo que se tenía que hacer, están profundizando libre comercio, luego viene problemas de desempleo, de pobreza y de informalidad mayores, y eso va a repercutir en el sistema de salud en el horizonte de corto plazo de aquí al 2020, después no sé qué pueda pasar. Entonces por ese lado es evidente lo que usted señala del envejecimiento del país es un hecho cierto, y hay otro hecho que también es bien cierto y es la tendencia de creciente costos en la salud, precisamente por el envejecimiento, en el caso de la salud los desarrollos tecnológicos se terminan convirtiendo en nuevos costos de salud, entonces los problemas de las finanzas, de las EPS o del sistema de salud, se van a complicar, van a haber menos gente en el contributivo y más pobres y más necesidades en el subsidiado

P14: 8. Transcripcion Jorge E. Robledo_ok.docx - 14:4 [Si porque va a aumentar el des..]  (13:13)   (Super)
Códigos:	[modelo de salud futuro] 
No memos

Si porque va a aumentar el desempleo y van a caer los salarios. Y hagamos una anotación, por el lado de reducir la corrupción y todas las practicas indeseables de la EPS etcétera, en eso no se está haciendo nada, entonces en los próximos años eso va a aumentar y eso también agrava el funcionamiento del sistema de salud

P14: 8. Transcripcion Jorge E. Robledo_ok.docx - 14:5 [Ellos en cobertura formal podr..]  (15:15)   (Super)
Códigos:	[modelo de salud actual] [modelo de salud futuro] 
No memos

Ellos en cobertura formal podrán mantener el indicador muy alto porque además esto es un régimen en el que estamos hoy montados sobre la publicidad y sobre el engaño. Los carnet son baratos de entregar, el problema es que todos sabemos las barreras de acceso, entonces usted tiene carnet y él lo protege de todo pero inclusive ya empieza a haber problemas de acceso en las prepagadas, esto se está degenerando rápido más todavía, entonces lo que va a pasar más adelante, el punto es que las barreras de acceso van a hacer cada vez mayores, porque pues no puede ser de otra manera porque las medidas que deberían tomarse, como por ejemplo acabar con la intermediación de las EPS, esa esta claro que no la toman bajo ninguna consideración, eso lo tiene claro el Banco Mundial, toda la banda neoliberal de la cual hace parte Alejandro Gaviria (Ministro de Salud)  entonces no hay otra manera de equilibrar un sistema desequilibrado que  es reduciendo los servicios a la gente. Lo ha dicho Gaviria en cierto sentido en todos los tonos él dice que el POS es demasiado costoso, todos los que son los derechos superan la capacidad financiera del sistema, es su gran teoría lo que nunca explica es que si eso es así, porque se les permite las ganancias a las EPS y porque se le permiten todos los fraudes a las EPS, porque si falta plata lo menos seria pedirle la coherencia de reducir los costos y unos de los costos principales de este asunto son las ganancias, que son distinta a los costos de administración 

P14: 8. Transcripcion Jorge E. Robledo_ok.docx - 14:6 [Claro, aquí no más el magister..]  (17:17)   (Super)
Códigos:	[modelo de salud futuro] 
No memos

Claro, aquí no más el magisterio y las fuerzas armadas no tienen EPS, ahora funciona muy mal pero por otras razones pero se demuestra que la administración del sistema de salud  no requiere de poder hacer ganancias con el pretexto de la administración. Aquí el magisterio y las fuerzas armadas y varias universidades la nacional por ejemplo y Ecopetrol funcionan sin EPS, eso no es una necesidad técnica, es una necesidad técnica que tenga administración, pero no que haya ganancia que es como los bancos que le ganan la plata que le dan los Colombianos y eso no tiene por qué existir. Hay muchos países del mundo que eso no existe.

P14: 8. Transcripcion Jorge E. Robledo_ok.docx - 14:10 [Yo no he mirado eso con deteni..]  (29:29)   (Super)
Códigos:	[modelo de salud futuro] 
No memos

Yo no he mirado eso con detenimiento, pero yo creo que puede ser las dos cosas juntas uno lo ha dicho Gaviria que quiere EPS más grandes y este es un negocio que en Colombia tiene una novela. En cierta medida todo este modelo de privatización, y es eso lo que yo estaba mirando aquí, un proyecto de investigación de la universidad de los andes, dice "la globalización ha llevado a las naciones industrializadas del occidente presionadas por la competencia japonesa y asiática a abrir nuevas ramas de acumulación de capital telecomunicaciones, energía y servicios, agua y recolección de basuras especialmente" Esto le cabe a salud también, obviamente es lo mismo y dice "esto y la caída de las tasas de ganancias en los países del norte, mejoro las condiciones para rentabilidad de inversión en nuestros países, de esta manera se creó el contexto político y para la intervención del capital nativo y extranjero en esta rama de la actividad". O sea el problema de los ricos del mundo es que tienen un exceso de ahorro que llaman los economistas, ganan demasiada plata y el problema de los pobres es ver que tan endeudados, en cambio los ricos es ver qué hacer con los que ganaron. Parte del asunto es ese y eso lo ha planteado Gaviria, él dice debemos hacer EPS más grandes, entonces ese tipo de medidas de las que estamos hablando en parte apuntan a achicar el número de oferentes que están dentro de este contexto, pero además no resuelve el problema, el problema necesitara de otras soluciones, pero bueno van avanzando en la dirección que les interesa

P14: 8. Transcripcion Jorge E. Robledo_ok.docx - 14:11 [Claro, porque la ganancia sale..]  (35:35)   (Super)
Códigos:	[modelo de salud actual] [modelo de salud futuro] 
No memos

Claro, porque la ganancia sale de la salud que se niega, es así de simple si usted mira la UPC, la ganancia no está formalmente dentro de la UPC, pero de ahí tiene que salir eso es una cosa muy torcida y nace con una lógica muy corrupta, porque la ganancia solo puede salir de la medicina que usted niegue, porque los costos de administración están incluidos en las cuentas pero la ganancia no, del derecho que se niegue. Se supone que la UPC está bien calculada, si eso estaría perfecto no habría de donde salir ganancia, solo negándole a alguien el servicio

P14: 8. Transcripcion Jorge E. Robledo_ok.docx - 14:12 [EL punto es este, cualquier si..]  (37:37)   (Super)
Códigos:	[modelo de salud actual] [modelo de salud futuro] 
No memos

EL punto es este, cualquier sistema de salud del mundo necesita gastar plata en cuatro cosas, medicinas, profesionales de la salud y  trabajadores, procedimientos quirúrgicos,  hospitalización  y administración. Usted no puede tener un sistema sin gerentes sin contabilistas. Estos cuatro costos son inexorables en cualquier sistema de salud del mundo, ¿Cuál es el problema con el sistema de aseguramiento? Es que se meten un quinto costo, que es la ganancia de las EPS que es distinto al costo de la administración, Palacino nos costaba un montón de plata como gerente de la EPS pero yo diría que se la pagamos, pero además el tipo hacia ganancias con eso, además de que asaltaba el sistema y el sistema esta modelado para esa ganancia lo que pasa es que esto es una cosa con un diseño fraudulento que ni siquiera eso lo deja claro desde el principio, es más la corte constitucional tiene una sentencia en la que le exige al gobierno determinar cuál es la plata de la administración porque ellos ya ante el acoso de este debate dicen no es que la ganancia sale de la administración para no insinuar que sale de quitar salud, entonces la corte constitucional le dice a Gaviria usted me tiene que determinar cuánto es lo que tiene que ser ganancia y eso nunca lo determino. Y el fraude grande de todos estos tipos que lo han seguido haciendo todos estos años los interventores de salud es robarse la plata mediante el truco y eso se los oriento Montealegre cuando fue abogado de salud, que es inventarse el cuento que de la UPC también se paga la construcción de clínica la compra de ambulancias, y se compran salas de cirugía pero resulta que eso no está dentro de la UPC y eso pasa directamente al capital de la EPS, porque aquí hay una cosa que es monstruosa, los recursos de salud son parafiscales que el ciudadano paga y se vuelven públicos pero con una destinación específica parecido a como el primer fondo que hubo en Colombia el fondo nacional del café, pero en el caso del fondo nacional del café que lo administran la federación de cafeteros y la federación cobra por esa administración tuvieron la delicadeza de separar las cuentas del fondo nacional del café y las cuentas de la federación de cafetera y sin embargo ha habido fraudes y esas cosas. Esto les han permitido mantener un revoltijos de plata y nadie sabe de donde es una cosa y la otra, pero todo eso es con este propósito de la ganancia, y no tuvieron la delicadeza de hacer eso separando unas cosas de otras, y fijando unos límites específicos sino que le dieron el permiso de manejar esa vaina como se les da la gana y con eso estamos arruinados pero sus patrimonios son inmensos, están arruinadas algunas pero usted va a ver esa gente que empezó esos negocios con la cedula y que hoy tienen como dice un amigo mío, edificios blancos de letras doradas, esos tienen unos patrimonios inmensos que han salido del negocio. En este mundo de trucos contables y en el caso de algunas instituciones que son de tipo cooperativo apareció una especie de crimen que es el gerente de la EPS crea las empresas que roban a las EPS, pero esas empresas si son personales. 

P15: 9. Transcripcion Julian Duran_ok.docx - 15:3 [La realidad parece mostrar que..]  (15:15)   (Super)
Códigos:	[modelo de salud futuro] 
No memos

 La realidad parece mostrar que no se está preparando porque como dice usted ese aumento de la población mayor de 60 años va a generar una gran presión va a aumentar la presión en el sistema nacional de salud y eso requeriría que en este momento se estuvieran implementando nuevas estrategias para enfrentar eso, pero no se ven esas estrategias, por ejemplo en una medicina más orientada  hacia la prevención pero no vemos que se estén implementando esas estrategias actualmente

P15: 9. Transcripcion Julian Duran_ok.docx - 15:21 [Pues la verdad falta mucho par..]  (118:118)   (Super)
Códigos:	[modelo de salud futuro] 
No memos

Pues la verdad falta mucho para que se implemente porque no se ve una voluntad política de los gobiernos ni de los ministros de salud en aplicar este tipo de estrategias, desconozco porque razón pero si se nota una falta de voluntad en aplicar este tipo de políticas.

P16: 10.Transcripcion Angela Tascon.docx - 16:4 [Si es bueno por lo menos regul..]  (11:11)   (Super)
Códigos:	[modelo de salud futuro] 
No memos

Si es bueno por lo menos regula un poquito el mercado, si dijéramos que tuviese al menos un piso tarifario pero actualizado porque si hay unas partidas pero están completamente desenmarcadas de la realidad, estamos hablando de un piso tarifario 2001, entones me parece que está muy bien estructurado fue muy bien hecho pero está totalmente desactualizado, pero es un norte que nos ayuda a fijar las tarifas en este momento.

P16: 10.Transcripcion Angela Tascon.docx - 16:5 [Se deben cambiar no podría dec..]  (15:15)   (Super)
Códigos:	[modelo de salud futuro] 
No memos

Se deben cambiar no podría decir exactamente qué, pero si buscar las herramientas para frenar la tutela, en el sentido que se volvió una herramienta dijéramos corrupta, entonces usted en tutela para que le den pañales, en tutela para que le den el medicamento que tiene que ser traído de otra parte, es decir son una cantidad de necesidades que la tutela misma ha creado facilitándole a la gente que hace perverso el sistema. Entonces yo diría que la tutela es una herramienta y es buena pero los que favorecen la tutela que son los jueces no tienen ni idea entonces a usted le favorece positivamente una tutela para una prótesis de pene para una persona de 80 años, entonces ve uno cosas demasiado absurdas que han sido favorecidas por este elemento que podría ser útil pero que llego a un punto de distorsión total de mal uso. La parte de la participación de los médicos a mí me parece que el medico es el actor principal del acto asistencial como tal, y el medico como tal ha perdido muchísima facilidad en su labor porque está limitado por tiempo, por los mismos elementos del sistema como lo son los medicamentos, que esa seria también otra parte importantísima que el sistema tiene que cambiar, mejorar la parte de controlar los laboratorios en los costos, ellos manejan todo y eso que ya con las últimas reformas se ha logrado controlar un poquito más el mercado, yo pienso que este ministro ha trabajado bastante en eso y se ha logrado pero sabemos muy bien que países como Brasil manejan medicamentos de alto costo como el sida, mucho más baratos que los que se manejan acá. Entonces yo diría que el control a los laboratorios esos contratos gigantes internacionales que manejan los laboratorios tienen mucho poder, y mientras no se controle eso sale más barato como exponía un alto ejecutivo que le costaba más barato comprar en Francia que acá. 

P16: 10.Transcripcion Angela Tascon.docx - 16:7 [LA verdad si seguimos como est..]  (22:22)   (Super)
Códigos:	[modelo de salud futuro] 
No memos

LA verdad si seguimos como estamos yo veo al sistema muy perverso, muy deteriorado precisamente esta tendencia que ya casi estamos en el 100% de la universalidad, pero mal financiada hace que las instituciones que realmente prestan el servicio como lo son las clínicas y los hospitales, cada vez están más deterioradas porque cada vez el sistema pide más y da menos, entonces digo que si seguimos en este camino, con una intervención del gobierno muy por encima yo diría que en 10 años si seguimos a este ritmo lo veo peor. Pues colapsar no tanto porque cuanto llevas con este tema y dijéramos se buscan herramientas y se ponen paños de agua tibia como fue el pago del fosiga, o los tiempos que le ha dado el gobierno a EPS como Coomeva, que le ha dado 7 años de gracia para que se estabilice, pero si no hay control yo diría que vamos a seguir en el mismo tema.  Colapsar no porque el sistema lo que está amparando es el derecho constitucional a la vida y a la salud, como le parece que eso colapsara pero si muy deteriorado si seguimos como estamos

P16: 10.Transcripcion Angela Tascon.docx - 16:8 [Pues dijéramos un sistema muy ..]  (26:26)   (Super)
Códigos:	[modelo de salud futuro] 
No memos

Pues dijéramos un sistema muy racional donde le manejo de los recursos fuera como paralelo en como dijéramos directo a esta universalidad, entonces más apoyado en estadística científicas, para poder decir que las enfe3rmedades de alto costo fueran bien manejadas, entonces yo vería un sistema ideal como maneja un banco sus recursos que los cuida que les hace seguimiento, entones un sistema así tratando de cumplir el propósito de atención universal. 


P16: 10.Transcripcion Angela Tascon.docx - 16:9 [Lo que yo creo en este momento..]  (30:30)   (Super)
Códigos:	[modelo de salud futuro] 
No memos

Lo que yo creo en este momento resulta de que si es posible ya hay intereses muy externos dignos que hay grupos externos que quieren estar aquí, eso quiere decir que el sistema bien manejado si es un sistema productivo entonces yo digiera que en 10 años si el gobierno no se pone las pilas y los colombianos no nos ponemos las pilas grupos externos nos van a manejar la salud, porque ya tenemos grupos fuertes manejando la salud aquí ya tratando de meterse y de invertir, eso me cuestiona a mi mucho porque digamos yo que estoy acá y trabajando con pesos y estirándolos, entonces uno dice como hacen, pero es porque ellos tienen una visión macro donde van a manejar y a controlar, yo digo que el problema principal es la falta de control, la terceriarizacion la vuelta que da el recurso, entonces yo creo que de verdad que el sistema es viable, el sector siempre y cuando tenga unos buenos administradores, como decían ahora respecto de esta reforma tributaria por ahí una congresista la escuche, ponía cifras que controlaban la corrupción y demás, son muchos mayores que los 3 puntos que estoy buscando con el IVA, yo digo que el problema inmenso de nuestro país es la corrupción y la politiquería. 

P16: 10.Transcripcion Angela Tascon.docx - 16:12 [No para nada, es que todo eso ..]  (50:50)   (Super)
Códigos:	[modelo de salud futuro] 
No memos

No para nada, es que todo eso se logra con recursos, entonces el gobierno en este momento no está en capacidad y a futuro debe ser posible porque ese decreto es un deber ser, y el deber ser es una prioridad es transversal es en valores es el deber ser. Entonces yo lo que pienso es que el gobierno no facilita no tiene las herramientas para que ese decreto y ese deber ser se diera completo, porque ahí hay unas palabras claves, y que son profundas que exigen un trabajo arduo. 

P16: 10.Transcripcion Angela Tascon.docx - 16:14 [Yo diría que sí, yo estoy muy ..]  (58:58)   (Super)
Códigos:	[modelo de salud futuro] 
No memos

Yo diría que sí, yo estoy muy de acuerdo en que la promoción y la prevención es la base de todo y si esa se expande vamos a tener gente más saludable a pesar de que hemos mejorado muchísimo en la parte del estándar de vida, hemos subido muchísimo, pero nos falta mucho.


P18: 11. Transcripcion Fabian Mendez.docx - 18:4 [Yo creo que necesita una refor..]  (7:7)   (Super)
Códigos:	[modelo de salud futuro] [Que debe conservarse del sistema de salud] 
No memos

Yo creo que necesita una reformulación de fundamentos, yo creo que en labores de inspección de vigilancia y control, todo el asunto de la vigilancia de la calidad como esta formulado, es decir si uno mira el sistema en términos de cómo esta formulado hay elementos que son válidos pero la intermediación no estoy de acuerdo en la existencia de un modelo como el actual con intermediarios, creo que ha sido muy negativo para el sistema, creo que un sistema fundamentado en relaciones económicas no es lo que el país necesita, en ese sentido yo cambiaria casi todo cambiaría el sistema por un sistema único de salud, por encuestas manejado por el estado, los privados podrían tener un espacio pero siempre bajo la regulación y control del estado como su principal ente rector y no dejándole muchas de esas funciones como ha ocurrido en los privados

P18: 11. Transcripcion Fabian Mendez.docx - 18:5 [Estamos en una coyuntura muy p..]  (11:11)   (Super)
Códigos:	[modelo de salud futuro] 
No memos

Estamos en una coyuntura muy particular hoy en el país con todo lo que significa los acuerdos del estado de las FARC y lo que viene hacia adelante puede ser determinante. Creo que el sistema no aguanta más en términos de viabilidad financiera creo que ya está mostrando bastantes varias décadas de estar intentando un modelo lo arreglamos por un lado, lo arreglamos por acá pero no muestra señales. Y necesita una reformulación, pensar en el MIAS en las políticas de tratar de volver a la salud pública a la atención primaria desde este modelo, creo que va a ser muy complicado y va a ser visible las contradicciones que tiene alrededor de que no es un modelo que garantiza el derecho a la salud como un asunto fundamental. Entonces en principio se escucha muy bien es volver a la atención primaria, pero yo creo que puede ese sistema la implementación del MIAS, y de la política puede hacer que se hagan visible las contradicciones que tiene y que vaya a la postre a la necesidad de pensar una reformulación, no creo que el modelo aguante más los asuntos de corrupción que tenemos, la iliquidez del sistema lo que estamos viendo cada vez más, los problemas de falta de acceso real porque lo que tenemos es una población cubierta entre comillas pero sin real acceso. Es un sistema que como yo lo veo en la coyuntura actual del país de una situación política que puede y ojala si uno es optimista significar cambios significativos en como esta formulado actualmente, entonces yo esperaría que habrán cambios.

P18: 11. Transcripcion Fabian Mendez.docx - 18:9 [Yo no creo porque hay por supu..]  (19:19)   (Super)
Códigos:	[modelo de salud futuro] 
No memos

Yo no creo porque hay por supuesto políticas formuladas y el plan decenal habla de esto de la importancia de las crónicas y aun el MIAS y el país habla de la importancia de las crónicas, pero esto implica un cambio en la forma en como enfrentamos los problemas de salud más allá de los curativos, hacia la promoción. Entonces yo creo que el asunto necesita un cambio dramático para que realmente el derecho a la salud y lo que la ley estatutaria dice sea realmente real. Porque hay mucha letra muerta muchas cosas que decimos que escribimos, pero que a la postre no se concreta para los derechos fundamentales para la población.

P18: 11. Transcripcion Fabian Mendez.docx - 18:10 [Cuando hablamos de un sistema ..]  (23:23)   (Super)
Códigos:	[modelo de salud futuro] 
No memos

Cuando hablamos de un sistema único, con un estado que retome su función de rector y garante de la salud de la población, donde haya  posibilidad de participación de privados pero bajo reglas muy claras de prestación de los servicios y donde prime el derecho a la salud sobre las relaciones económicas. Ese escenario ideal implica tener un estado que piense la salud y la educación como requerimientos fundamentales para buscar justicia social.

P18: 11. Transcripcion Fabian Mendez.docx - 18:11 [Estamos en un péndulo, pero do..]  (27:27)   (Super)
Códigos:	[modelo de salud futuro] 
No memos

 Estamos en un péndulo, pero donde ni aun como me decía un político español, me decía ni la derecha española concebiría un sistema tan privatizado como el colombiano, es decir ni el PP se la jugaría por un modelo como el que tenemos acá en Colombia. 

P18: 11. Transcripcion Fabian Mendez.docx - 18:13 [Si ese es el panorama que nos ..]  (35:35)   (Super)
Códigos:	[modelo de salud futuro] 
No memos

 Si ese es el panorama que nos han pintado que parece que viene, tenemos demasiadas EPS y la política actual apunta hacia allá que queden menos y que esas menos en manos de unos pocos, entonces supuestamente el sistema de vigilancia va a funcionar mejor, la inspección en vigilancia va a funcionar mejor y eso va a lleva que mejor su desempeño. Las raíces bases del sistema donde digamos las acciones de promoción y prevención están en un segundo nivel, lo que usted mira ahora cómo es posible que en un sistema se está creando cada vez más IPS de alta tecnología y lo fundamental no haya quien lo cubra, que el hospital Universitario este en estragos. Yo creo que se está aceitando la maquinaria para sacarle más provecho a unos recursos que si hay muchos, y si cada vez vamos a tener y hay muchos recursos pero con una visión curativa y donde la salud pública no juega un papel fundamental. 

P18: 11. Transcripcion Fabian Mendez.docx - 18:17 [Hay algo central y es que noso..]  (75:75)   (Super)
Códigos:	[modelo de salud futuro] 
No memos

Hay algo central y es que nosotros tenemos unas cohortes de población que son el resultado de unas condiciones de desigualdad muy grandes, cuando hicimos esta encuesta en 2015 en el año pasado, incluimos población mayor de 60 años, estamos hablando de gente que nació antes de 1955, y tuvimos gente hasta después de los 90 ósea que estamos hablando que nació entre 1920, 1915 y 1955. Esa población es la que forma esa cohorte y va ingresando cada vez más, ahí va a haber un cambio importante en esa cohorte, pero ahora mismo tenemos todos estos que nacieron antes del 55 que van a seguir envejeciendo, porque entre los 60 y los 70 todavía tenemos un grupo de gente que están activos social y laboralmente, después de los 70 es más complicado el asunto y esos van a ser cada vez una proporción más grande de gente que tienen niveles educativos muy bajos, su ocupación es de trabajo informal y hoy todavía siguen trabajando, no por placer si no por necesidad.  Entonces estamos con una población que tienen en su curso de vida una acumulación de factores asociados de riesgo que van a  llevar a una carga muy grande, y esta población no tiene las condiciones básicas que un estado en seguridad social le haya permitido construir, las salidas que están dando el estado son menores, no sé si usted conoce el programa mayor que le da 160 pesos cada dos meses a un anciano. Son mecanismos remediales insuficientes, y en términos en derecho tenemos una población muy vulnerable, con una cantidad de riesgos acumulados durante toda la vida, con un nivel educativo bajo, pero además súmele a esto unas redes sociales, porque es un país que paso de ser rural, muy rápidamente a ser urbano vivíamos antes en espacios donde las redes sociales apoyaban más al adulto, lo que encontramos en la encuesta fue a adultos mayores viviendo en ciudades grandes uno de cada cinco habitantes mayores de 60 años en Colombia, fue desplazado en algún momento del campo a la ciudad, y lo que es más impresionantes es que 9 de cada 10 de esos tuvo moverse nuevamente, hay un asunto de repitibilidad muy grande en gente que fue afectada por el conflicto. Entonces que va a pasar con esta población que está aislada viviendo del rebusque y con bajo nivel educativo, la respuesta en términos de salud para esa población es un reto muy grande con costos enormes para el sistema de salud y yo no veo como los van a suplir.

P18: 11. Transcripcion Fabian Mendez.docx - 18:18 [En principio lo que está en el..]  (79:79)   (Super)
Códigos:	[modelo de salud futuro] 
No memos

En principio lo que está en el papel es digamos un cambio en la dirección positiva, de volver a pensar después de estar en un sistema completamente centrado en la atención, en la medicación esta, vuelve y dice hay que pensar en la promoción a la salud, hay que pensar integralmente. Pero yo veo dificultades tremendas en como esto se operiviza en el sistema y en cómo está construido. Yo no creo que para las EPS sea de interés porque su interés es fundamentalmente el lucro, desarrollar actividades con un enfoque de territorio y entonces lo que a la postre vamos a ver es más recursos que llegan a las EPS y donde se cree que esto se va a resolver con médicos familiares, es una visión de la atención primaria donde lo intersectorial no muestra un papel central esa concepción de la atención primaria en programas creo que es una visión parcializada de lo que es la atención primaria, y yo no creo que las EPS puedan ser actores reales porque no son actores que trabajan con una visión de territorio. Entonces yo creo que allí es donde el sistema tiene un diseño una concepción que hace que a pesar de lo que se formules y aparezca en el papel real seguro en el territorio va a ser muy difícil que un sistema además que no garantiza esos derechos los vea efectivo. Implicaría un cambio muy sustancial en la forma como funciona actualmente el sistema.

P18: 11. Transcripcion Fabian Mendez.docx - 18:19 [Yo creo que se necesitaría un ..]  (83:83)   (Super)
Códigos:	[modelo de salud futuro] 
No memos

Yo creo que se necesitaría un cambio político importante, a pesar de mi escepticismo yo creo que uno tiene que dentro de este mundo manejar una ventana de optimismo porque si no apague y vámonos. Yo defiendo y creo que hay unas cosas positivas dentro del modelo, pero creo que para poder que el MIAS sea una realidad hay que hacer un cambio sustancial en términos internos de la rectoría y la forma de cómo está organizado el sistema

P18: 11. Transcripcion Fabian Mendez.docx - 18:21 [Yo confió en que seamos lo suf..]  (91:91)   (Super)
Códigos:	[modelo de salud futuro] 
No memos

Yo confió en que seamos lo suficientemente inteligentes en el país para entender que la salud es un bien necesario y fundamental para el desarrollo, y aun con los modelos de desarrollo económico, tener una población sana, tener una población que tenga real acceso a los servicios de salud, va a permitir que se logren avances aun en lo económico y bajo ese principio y bajo un cambio político importante en el país donde otros actores entran a jugar se pueda poco a poco ese derecho a la salud de la ley estatutaria hacerlo real, porque si yo no confiara en que esto no va a cambiar de manera positiva pues sería casi que vámonos pal monte otra vez

P19: 12. Transcripcion Fabio Osorio.docx - 19:4 [Las EPS si no pudieron entrar ..]  (11:11)   (Super)
Códigos:	[modelo de salud futuro] 
No memos

Las EPS si no pudieron entrar en casi 20 años, no van a poder entrar en 10 años más son creadas de la misma ley 100 del 93, las EPS fundamentan la atención en un pago mensual que va al trabajador o al estado por una UPC o por una unidad de capitación por cada afiliado que tenga la EPS le dan una plata. Las EPS deben de desaparecer del sistema de salud, son las intermediarias que es donde la plata se pierde. Elk ministerio de salud debe de girarle directamente a las clínicas y hospitales el dinero por la atención de pacientes, yo defenderé la tesis que las EPS deben de desaparecer, casos como CAPRECOM, como CAFESALUD, como SALUDCOOP son una vergüenza nacional porque jugaron con la salud, privilegiaron lo económico a los social. Las EPS con el tiempo que les den si no pudieron en 20 años, ya no podrán en 10 porque fueron el fortín politiquero de muchos congresistas de Bogotá que hicieron sus negocios con la salud de la gente que tristemente confiaron en ellos y las EPS fueron la caja menor de muchos políticos en Colombia.

P19: 12. Transcripcion Fabio Osorio.docx - 19:5 [Si la salud en Colombia se ded..]  (14:14)   (Super)
Códigos:	[modelo de salud futuro] 
No memos

 Si la salud en Colombia se dedica a la atención y no maneja la prevención y no maneja la promoción en salud vamos a tener muchas dificultades acá en Colombia esperamos que se enferme la gente para atenderla, acá no hay promoción ni prevención, en Colombia hay nivel de atención 1, 2 3  y especializado que es el 4, si el del nivel 1 hay promoción para la diabetes que está matando mucha gente, si hay promoción para la hipertensión, para los embarazos, para suicidio, seguramente los niveles 2 y 3 no tendrían tanta demanda. Hoy en Colombia se descuida esa promoción y prevención por eso muchas veces nuestros adultos mayores se están enfermando con mucho más riesgo y la enfermedad es difícil de controlar porque no se detectó a tiempo, peor aún somos s un país con mucha violencia y tristemente los adultos mayores están enterrando sus hijos o a sus nietos debido a la gran violencia que hay, si no hay políticas públicas de atención en salud dese de lo preventivo y lo promocional, no vamos a poder tener respuesta cuando nos llegue tanto enfermo de tan complicada enfermedad de 4 subgrado de patología que va q ser muy difícil de ,mantener el sistema de salud con los gastos que hay y que acá en Colombia para que te den un medicamento te dan siempre el genérico y el genérico de una u otra forma hace que la enfermedad se combata no con tanta efectividad, por un medicamento comercial quien casi que lo obligan a comprarlo para que tengan una recuperación más rápida en su enfermedad. 

P19: 12. Transcripcion Fabio Osorio.docx - 19:7 [Si acá en Colombia no se acaba..]  (22:22)   (Super)
Códigos:	[modelo de salud futuro] 
No memos

Si acá en Colombia no se acaba con la politiquería en los hospitales y clínicas de salud, no se acaba con la corrupción en salud, ningún sistema de salud va a aguantar semejante forma de robarse los recursos en salud. Es increíble que saludcoop siendo una EPS grande a nivel nacional e internacional haya pensado más en construir canchas de golf, equipo de futbol en tener acciones en México que invertir en sus afiliados, dentro de 10 años si no hay de ahora una reforma estructural una  reforma que en verdad privilegie la atención no va a pasar nada, los enfermos van a seguir llegando y la población, porque la salud aunque no sea un negocio hay que cobrar por la atención en pacientes, la salud como negocio debe privilegiar lo social no lo económico, la salud en Colombia dentro de 10, 20 años si no se estructura desde ahora vamos a llegar a lo mismo, el gobierno dice es que ya aseguramos a la gente, pero señor gobierno la aseguración y el sistema de salud no es un carnet, es una atención todos pueden tener un carnet, pero si no hay atención no va a haber buen aseguramiento y si no hay promoción ni prevención dentro de 10 años estaríamos con muchas dificultades en la atención a la población en Colombia. 


P19: 12. Transcripcion Fabio Osorio.docx - 19:8 [En el sistema de salud de la l..]  (26:26)   (Super)
Códigos:	[modelo de salud futuro] 
No memos

En el sistema de salud de la ley 100 se habla de equidad de igualdad de honestidad de oportunidad, para mi sigue siendo letra muerta si no hay un control de los recursos del estado a los hospitales el sistema de salud que yo sueño para mi país, es que los recursos del estado lleguen directamente a los hospitales que no haya tramitología ni intermediación, cuando estos recursos lleguen a los hospitales que tengan gerentes que piensen en no robarse los recursos que no hagan corrupción con el gobierno de turno sea alcaldía o gobernación, para desviar los recursos oportunos que lleguen a los hospitales, si hay un buen gerente en salud que piense en salud uy no en negocio y que esos recursos sean vigilados por los entes de control no por asustadurias, que solamente sacan informes y no proceden, si no como entes de control como la procuraduría que sancione a los malos dirigentes públicos para que haya un buen control, con la contraloría que le haga un seguimiento a los recursos para que si el acetaminofén vale 500 pesos se pague 500 no 5000 para que se roben 4500, unos buenos entes de control unos buenos administradores en salud y que los dineros lleguen oportunamente a los hospitales creo que ese es el sistema de salud que yo sueño. Y más aún un sistema de salud humanitario que el trabajador de salud sienta que esa persona que está siendo atendida sea como su familiar, que haya un respeto por el paciente, que entienda que no es una maquina es un paciente que necesita ayuda, y que así se muera por la enfermedad tenga una muerte digna y que el sistema de salud sea humanizado y no mecanizado. A un médico de hoy en día le dan 20 pacientes para que atienda en 2 horas, es imposible que un paciente tenga la posibilidad para que su médico lo trate con tiempo y con oportunidad para que lo trate con eficacia y tiempo, en el tratamiento de la enfermedad. 

P19: 12. Transcripcion Fabio Osorio.docx - 19:9 [Totalmente de acuerdo, yo sigo..]  (30:30)   (Super)
Códigos:	[modelo de salud futuro] 
No memos

Totalmente de acuerdo, yo sigo pensando que el ministerio de salud en Bogotá tiene unas secretarias en los departamentos y una secretaria de salud en las ciudades que esos dineros vayan directamente a los hospitales que solo la secretaria le haga seguimiento y la entrega pero cuando se le mandan a una EPS pero cuando se lo mandan a una EPS para que se lo manden a los hospitales ahí es donde está el hueco, esas EPS juegan con los recursos caso caprecom, saludcoop, calisalud, yo siempre lo he sostenido si los dineros llegan directamente a los hospitales van a tener mejor control pero con un intermediario es donde se pierden. De Bogotá mandan 5000 millones para el hospital pero cuando llegan a la EPS ya van 3000, y cuando llegan a el hospital van 1000

P19: 12. Transcripcion Fabio Osorio.docx - 19:14 [Va a seguir igual si no hay so..]  (70:70)   (Super)
Códigos:	[modelo de salud futuro] 
No memos

Va a seguir igual si no hay soluciones de fondo la ley 100 es una colcha de retazos, una cantidad de leyes que sacan para tapar los huecos si acá no hay una reforma estructural desde la ley 100, esa tiene que desaparecer tiene que haber un nuevo sistema de salud que apremie la oportunidad en la atención a pacientes, si desde ahora no hacemos algo a mayor tiempo mayor población,

P19: 12. Transcripcion Fabio Osorio.docx - 19:17 [Yo pienso que nosotros podemos..]  (82:82)   (Super)
Códigos:	[modelo de salud futuro] 
No memos

Yo pienso que nosotros podemos hacer historia, hacer que cambie, podemos entregarle a nuestros hijos un sistema de calidad en salud bacano uno poder entregar a las personas que seguirán en su ciclo de vida, que los que estamos ahora hicimos algo para que eso mejorara, por eso el llamado que yo hago independientemente de la forma de pensamiento o la ideología, hagamos respetar el derecho a la salud, es un derecho irrenunciable, usted tiene que movilizarse socialmente, siempre doy este ejemplo también acá la selección Colombia gana y salen todos, cierran un hospital y no pasa nada, acá en Colombia es triste que haya una feria de Cali y se llene más de 40 cuadras, pero acá se muere un niño por el sistema de salud y los únicos que pelean son las familias, entonces yo pienso que debemos ser humanizados y solidarios con el valor que me mostraste de salir a exigir el mismo derecho para todos independientemente de la situación

P22: 13. Transcripcion Maria Ines Pantoja.docx - 22:5 [Yo no creo porque mire, el min..]  (11:11)   (Super)
Códigos:	[modelo de salud futuro] 
No memos

 Yo no creo porque mire, el ministerio de salud en este momento crítico, no está proyectando nada porque está apagando incendios, en este momento están tratando de reglamentar la ley estatutaria, con resoluciones con decretos, pero yo no creo que el sistema esté preparado para recibir una población del 21% de la población envejecida como dice usted, con la demanda en salud que son más exigentes y vamos a estar más enfermos porque la vejez lleva a un desgaste físico entonces yo creo que el sistema de salud no está preparado para eso, el sistema de salud en este momento como esta tan quebrado está tratando de remendarse esos quiebres que tiene el sistema de salud, pero no hay una proyección a futuro y nosotros no se la vemos con este sistema de salud, donde el negocio predomina, la ganancia y la corrupción es muy difícil que se prepare para un futuro.

P22: 13. Transcripcion Maria Ines Pantoja.docx - 22:8 [Usted ve como las EPS, están d..]  (23:23)   (Super)
Códigos:	[modelo de salud futuro] 
No memos

Usted ve como las EPS, están debiéndole a los hospitales, una cantidad de dinero impresionante, entonces empezando por eso que es lo económico, por ahí ya las EPS no pueden responder porque hubo corrupción porque los dineros de la salud los utilizaron para otras cosas, los utilizaron para hacer fincas, para hacer edificios, clínicas, pero no para atender a las personas. Otro factor los laboratorios, la industria farmacéutica, no tenían control de precio y cada uno cobraba lo que quería por los medicamentos, y si usted oyó en todo el problema del control de precios, veíamos que los precios de Colombia eran inmensamente grandes frente a los países de américa latina y el mundo, porque se conseguían los medicamentos a otro precio. Entonces pues el ministro creemos que ha acertado en el control de precios, pero el control de precios solo se está haciendo en un parte de la cadena, no en toda la cadena de distribución de los medicamentos, entonces le hemos dicho al ministro que eso hace falta, porque no podemos dejar al libre albedrio, ya salió de la industria salió del laboratorio y de aquí para allá los precios pueden aumentar lo que quieran. No han bajado y no los quieren bajar los laboratorios. 

P22: 13. Transcripcion Maria Ines Pantoja.docx - 22:11 [En la reunión de Cali de la co..]  (29:30)   (Super)
Códigos:	[modelo de salud futuro] 
No memos

En la reunión de Cali de la comisión séptima, la del gobernadora le dijo a las EPS que si no le pagaban a los hospitales del valle, ella iba a coger el régimen subsidiado, y la presidente de Gestar salud, le contesto que primero los departamentos le pagaran 250 mil millones que les deben a las EPS, y el gobierno le dijo que el departamento no podía coger por nada del mundo el régimen subsidiado.
MP: Es que la 1479 tiene 3 modalidades, pero son dos las más importantes el departamento puede atender directamente, darle a la persona hacer el medicamento o todos los medicamentos del no POS, directamente ellos asumirlo, o también pueden decirle a la EPS que les presten el servicio y que el ente territorial le paga a la EPS, pero las EPS con  los entes territoriales, cada uno escogió una modalidad, por ejemplo Cundinamarca escogió que ellos mismos asumen dar el servicio, pero como la red pública de hospitales esta tan débil, se ha debilitado tanto, entonces nos preguntamos donde van a atender a esas personas de procedimientos de alta tecnología que no tienen donde atenderlo, entonces el departamento tendría que contratar con una red privada para que le atiendan a la gente, por eso nosotros fomentamos mucho y le decimos al ministerio hay que fortalecer la red pública, porque usted ha visto como se han cerrado de hospitales y todo lo que ha sucedido, entonces con la eliminación del CTC, solo se eliminan para el régimen contributivo mas no para el subsidiado entonces ahí los del subsidiado siguen con la misma barrera. Lo que nos decía la viceministra ayer que estuvimos con ella era que los departamentos pueden asumir la plataforma del ministerio, para prestar el servicio directamente por la plataforma, porque es una plataforma donde el medico formula y da la formula y ya no tiene necesidad de autorización para el no POS, pero solo para el régimen contributivo y los más vulnerables que son del subsidiado siguen en la mismas. Hay una gran diferencia, nosotros le vamos a hacer la consulta a la corte, porque es que la corte también sus sentencias ha dicho que los departamentos deben tomar sus responsabilidades, pero yo lo que pienso es porque no una plataforma común y que los departamentos giren al fosyga, o paguen de alguna manera pero es imposible por la ley, y la ministra decía seria lanzar al congreso la reforma de esa ley que los entes territoriales tienen que responder por el NO POS.


P22: 13. Transcripcion Maria Ines Pantoja.docx - 22:13 [Y sabe que es importantísimo l..]  (39:39)   (Super)
Códigos:	[modelo de salud futuro] 
No memos

Y sabe que es importantísimo la humanización del sistema de salud, eso es mortal eso es una inhumanizacion, y uno dice como a otro ser humano uno lo puede atropellar de esa manera. El hospital santa clara, ir a ese hospital donde la gente está tirada en el piso en cartones, eso es inhumano, pero como humaniza un sistema de salud usted, es muy difícil, yo le decía en naciones unidas que nos llamaron yo les decía, para que las EPS vuelvan a ser lo que deben ser, se necesitan 3 años de retiro espiritual muy profundo para que tomen conciencia de las aberraciones que tienen. Y me dijo el defensor del pueblo, yo me uno a este para hacerles el retiro. Es totalmente difícil porque tienen el negocio en su cabeza, entonces yo digo que la UPC alcanzaría si se gasta para lo que debe ser. Porque hacen edificios, porque tienen con qué, porque compran fincas, mire los que tienen empresa de turismo, los que tienen universidades, sanitas es una, tiene universidad, vende apartamentos y tiene agencia de viajes. Pero de sanitas de la EPS, yo hice un derecho de petición cuando me di cuenta que sanitas tenia empresa de turismo, estaba vendiendo apartamentos, eso nos pertenece a los usuarios porque somos los que estamos pagando para que se nos preste el servicio. Pero deberían decir esto lo están haciendo con esto y estoy con esto. 

P22: 13. Transcripcion Maria Ines Pantoja.docx - 22:16 [Yo no sé, yo digo que la esper..]  (84:84)   (Super)
Códigos:	[modelo de salud futuro] 
No memos

 Yo no sé, yo digo que la esperanza no se puede perder, pero es que como le digo sobre esta base quebradiza es como imposible construir algo nuevo, yo creo que es totalmente difícil o casi imposible, porque si le pongo esto más pesado acá a una cuestión se me va ir al piso, en la construcción no más si no tiene buena base no me va sostener. El problema del sistema es de fondo, estructural, acá nos encontramos con un paciente que hace 6 meses está en la clínica, con tutela le pidieron que le dieran hospitalización en casa, que le dieran una enfermera, prefirieron tenerlo en el hospital 6 meses, cuanto le cuesta una hospitalización en el hospital, en desde darle una hospitalización en la domiciliaria con una enfermera de 12 horas, y eso le mejora la calidad de vida. Y tutela desacato de todo, una persona en esta situaciones un hospital en 6 meses, en vez de darle lo que necesita en su casa, que muera dignamente, es que esto es totalmente aberrante. Mandan a una persona para Bogotá, le pagan el pasaje, le pagan estadía y no le dan la cita, entonces una persona la tienen acá gastando en un alberque 100.000 pesos diarios, un mes haga la cuenta cuanto le da, y la cita le costaba 80 mil pesos los exámenes 250 mil pesos, cuanto boto la EPS por negligencia de no dar las ordenes, es que ni siquiera por sentido de economía lo están haciendo, y que piensan es que ni siquiera en la plata, si yo pienso en el dinero digo hagamos rapidito para que me cueste menos, pero ni siquiera eso, yo no entiendo como dejan a esta persona hospitalizada 6 meses por no darle un servicio en casa, eso no tiene sentido. Pero es que si yo no administro lo poquito que me dan no va a alcanzar para nada, con tutela y con todo y no le han dado salida del hospital hasta que le den hospitalización domiciliaria. Y así hay muchos casos.

P22: 13. Transcripcion Maria Ines Pantoja.docx - 22:17 [Acá lo que se atiende es la en..]  (88:88)   (Super)
Códigos:	[modelo de salud futuro] 
No memos

Acá lo que se atiende es la enfermedad, no se atiende la prevención entonces la gente lo que busca es que me curen y llegamos y encontramos cantidad de barreras de acceso porque uno llega a la cita 20 días después y resulta que el médico no tiene el médico o el especialista porque solo hay un especialista porque no hay cita no hay agenda no hay nada entonces en todo ese lapso de tiempo la gente se va empeorando en sus salud y llega el momento en que la persona llega a la muerte y la mortalidad, pues es muy alta frente a enfermedades que son prevenibles y no hay una educación para un autocuidado, por ejemplo las personas diabéticas, si las tienen dentro de un programa donde les enseñan como alimentarse como vivir, esas personas van a sostener buena salud dentro de su condición de enfermedad, dentro de mi condición como trasplantada vivo bien, vivo con calidad de vida, pero si en las personas no se les da esa atención integral para que la persona desarrolle capacidades dentro de su misma enfermedad y hay una cosa que yo pienso es que uno debe conocer su cuerpo y sus síntomas, tiene que escuchar las alarmas que le da el cuerpo, si a mí me dan unas picada en la cabeza es por algo, y yo tengo que ponerle cuidado y cuando vea que eso es como muy seguido tengo que buscar al médico, pero la gente dice que eso pasa y no hay problema, porque no hay educación para tener esas capacidades que usted dice de yo escuchar mi cuerpo y de tener hábitos saludables y uno no necesita mucho dinero para eso. Acá hemos tenido experiencias de personas trasplantadas que han mejorado su entorno con lo poquito que tienen, pero han aprendido como cuidarse y están vivos y están bien. Por ejemplo hay una señora que vive en Patio bonito, ellos tenían la calle sin pavimentar con charcos, por esos zancudos y eso, la tarea de nosotros fue ir a mirar el sitio y mirar como creábamos para esa persona algo mejor, el piso era muy húmedo el baño era un baño sin baldosa y sin nada. Entonces en su casa se le puso en la pieza a la señora se le puso una plataforma con maderita para que ella no tuviera contacto con el piso, con pedazos de baldosa se recogieron y el señor esposo le arreglo el baño con eso, y le quedo un baño muy bonito donde ella podía estar y los charcos le enseñamos que le echaran ACPM para u que se murieran los zancudos y ella vive ahí ya han mejorado mucho, pero en esas condiciones estuvo ella, y si uno le enseña a la gente ciertas técnicas que no son muy costosas y se las apoya en eso de educación, la gente aprende y va haciendo. Nosotros tenemos un programa que se llama AQUIRO y empoderamiento, donde le enseñamos a la gente a defender sus derechos, a alimentarse a es

P22: 13. Transcripcion Maria Ines Pantoja.docx - 22:18 [Mire eso de sectorizar las pat..]  (95:95)   (Super)
Códigos:	[modelo de salud futuro] 
No memos

Mire eso de sectorizar las patologías a mí me parece muy grave, porque agrupan unas patologías y las patologías que el ser humano sufre son tan variable tan diferente cada ser humano que a mí me da angustia de que por ejemplo, si yo soy diabético y de diabetes ya pase a enfermo renal crónico entonces yo tengo que pasar de esta agrupación a otra y es que el ser humano no lo pueden partir, porque es integral y yo me puedo enfermar de muchas cosas, por ejemplo yo puedo tener trasplante de hígado y puedo estar mal de los riñones puedo estar con una artritis, entonces no sé cómo harán conmigo para meterme en todos esos grupos, no entiendo cómo pueden hacer. Es que fraccionar el ser humano es difícil y acá fracciona por patologías, eso lo quería hacer el ministro Palacios cuando la emergencia social, él nos llamó y dijo mire yo les armo un plan para ustedes para ustedes los enfermos hepáticos y renales y yo le dije es que nosotros no velamos solo por este grupo, nosotros velamos por la atención de los colombianos. Pero en el MIAS yo veo que han implementado eso, al meternos como en grupos, pero la idea de Palacios era esa, yo estoy acá como trasplantado y acá me dan un presupuesto, lo que hicieron con las huérfanas, las huérfanas tienen un presupuesto aparte, y eso me parece muy grave porque cuando uno tiene una enfermedad huérfana y tiene que pasar a otro grupo, entonces quedo en el aire

P22: 13. Transcripcion Maria Ines Pantoja.docx - 22:19 [el MIAS se pudiera caer si se ..]  (99:99)   (Super)
Códigos:	[modelo de salud futuro] 
No memos

el MIAS se pudiera caer si se demanda, porque la ley estatutaria le dijo que la participación durante todo el proceso, ninguna norma del país puede salir sin participación ciudadana. Y el MIAS lo hicieron sin ninguna participación y hablando con el doctor Ruiz muchas veces y le dijimos muchas veces que estuvimos en reunión con él, ellos creen que nosotros no podemos participar y de hecho si podemos participar porque somos los que conocemos la realidad de la gente. Por ejemplo en la eliminación del POS hemos estado muy pendientes de que haya una verificación de que el servicio se ha prestado, no simplemente una autorización. El problema está en que de verdad se goce del servicio. 

P23: 14. Transcripcion Mario Hernandez.docx - 23:5 [La tendencia es más bien a tra..]  (11:11)   (Super)
Códigos:	[modelo de salud futuro] 
No memos

La tendencia es más bien a tratar de ponerle un criterio de sostenibilidad a largo plazo que tiene tres elementos, y así es que entiendo yo la manera en que este gobierno en particular está tratando de prepararse para eso que es lo que han venido diciendo en los últimos días con este asunto del informe del envejecimiento de la población que acaba de salir, y con la política integral en salud. Cuál es la lógica que quiere manejar el gobierno para el mediano y largo plazo, primero ser muy fuertes en la decisión de las exclusiones, que queda excluido del sistema de seguridad social en salud y que será cubierto por las familias según su capacidad de pago, ahí seguramente habrán unos subsidios para los viejos pobres que demuestren estar en la peor condición en la extrema pobreza y el resto de acuerdo a su capacidad de pago verán como hacen, las familias tendrán un gasto adicional para eso insisten mucho en el deber del pago adicional a través de la ley estatuaria del artículo 10, y por otro lado una serie de incentivos positivos y negativos para el autocuidado que dicen también es una ley de la ley estatutaria, entonces van a trasladar fuertemente a las familias la responsabilidad por sus viejos y cada uno por sí mismo hasta viejo, de manera que si usted fumo 20 años y luego está reclamando oxígenos se le va a decir usted es culpable de su problema, entonces usted haga su pago o usted compre su oxigeno cosas de ese estilo, esa es la primera estrategia. La segunda tiene que ver con esta cosa de alinear los incentivos a través de un fortalecimiento de las EPS va a haber menos EPS, con unas redes integradas seguramente de manera vertical pero disfrazado con una integración comercial con un gran operador de muchos prestadores de segundo primero y tercer nivel, en territorios esa es la segunda estrategia muy fuertes que las EPS sean menos tengan monopolio, tengan económia de escala muy fuerte y tengan integración vertical con las redes. Y en tercer lugar ellos suponen que si simplemente la gente se cuida pues va a tener condiciones, pero se hará una cosa intersectorial con una platica que existe en salud que se llama plan de intervenciones colectivas para tratar que desde ahí se hagan unas cosas de promoción a la salud, a prevención a la enfermedad y se le diga a todos los demás sectores como es que tendrían que afectar las condiciones de vida a los famosos determinantes sociales, para evitar que la gente se enferme, ese es el proyecto, pero insisto en que esto desborda el asunto porque la lógica del mundo alrededor del tema de la atención medica es una lógica de acumulación de capital que se llama complejo médico industrial y financiero de la salud con una protección de la propiedad intelectual que hacen los grandes monopolios transnacionales y son los que imponen finalmente los precios y  por tanto los costos de servicio de salud en cada país definidos desde afuera definidos desde afuera, así esta de claro la cosa y como los viejos nos enfermamos cada vez más y somos grandes consumidores de esos bienes y servicios estimulados por la gran industria farmacéutica y la gran industria de equipos de tecnologías médicas, pues esto va a ser cada vez más impagable así de sencillo. El gobierno cree que puede aumentar un poco el valor de la UPC aumentado la cotización de los más ricos y aumentando los impuestos en algunas cosas muy puntuales, eso no les va a funcionar simplemente porque no se está afectando la causa de los sobre costos mundiales de la atención médica y al contrario se está generando una forma muy tímida, de acomodar el sistema actual a esa condición. Entonces en conclusión esa necesidad cada vez mayor de una atención más organizada para adultos mayores, pues va a ser poco resuelta o nada resuelta de parte del sistema

P23: 14. Transcripcion Mario Hernandez.docx - 23:10 [Yo creo que con esta política ..]  (30:30)   (Super)
Códigos:	[modelo de salud futuro] 
No memos

Yo creo que con esta política de atención integral van a llegar a ese tipo de acuerdo en uno o dos años, porque la reforma tributaria se viene este próximo semestre y se demorara 6 meses o hasta 1 año de pronto y ahí se prometió que se aumentaba la UPC, entonces bueno el siguiente elemento es como disminuimos los actores que no sea 60 y pico EPS si no que queden 10 o 15 y se encarguen de los dos y las territorializamos, ese negocio esa articulación esa negociación entre gobierno como regulador y con EPS como articuladores con los prestadores. 

P23: 14. Transcripcion Mario Hernandez.docx - 23:11 [Yo pienso que la tendencia com..]  (34:34)   (Super)
Códigos:	[modelo de salud futuro] [Protección financiera : financiación del SGSSS] 
No memos

Yo pienso que la tendencia como va es más hacia una disminución de actores y entonces son unos oligopolios o monopolios, en aseguramiento que manejan los tres regímenes prácticamente y hagan entonces sus ajustes de cuentas pero con economía de escala y por eso funcionara con un tipo de gestión del riesgo en salud similar al de káiser permanente, y quien lo está asimilando es SURA, como las EPS más eficiente supuestamente en incorporar eso y como es la cosa, pues simplemente si yo tengo 10 EPS, pues esas 10 EPS para todo el país están territorializadas, de manera que se distribuyen las zonas dispersas, primero una para cada zona dispersa Guajira por ejemplo, uno que maneja régimen subsidiado contributivo todo, después en las zonas rurales podría ser también pero repartiéndose una o dos máximo para poder articular con las grandes ciudades, y en las grandes ciudades van a terminar seguramente territorializando por localidades comunas o lo que sea, es decir, esa tendencia permite claramente que logren la economía de escala que dicen y disminuiría sus pérdidas, pero en esa reflexión no está claro porque pierden las EPS, porque y por donde se esta yendo la plata, y yo creo que ese análisis lo debería hacer un organismo de control, la contraloría, la procuraduría; el mismo Ministerio y la Superintendencia, no lo han querido hacer porque esa plata se ha ido a otras inversiones como saludcoop se demostró claramente que 1.4 billones de pesos se habían ido a cosas que no tenian nada que ver con la salud, inversiones en México, villa Valeria, cancha de golf, equipos de futbol, eso tiene algo que ver con la salud?  Y son recursos de destinación específica, entonces claro perdidas. Entonces como tengo que gastar todos los días de la UPC, entonces no tengo de donde pagar las deudas, así nos dice el ministro que son las cosas, entonces por eso tendría que capitalizarse para pagar las deudas y seguir pues no, les van a ir quitando las deudas diciendo que no son demostrables esas deudas al año ya se pierden, de manera que los prestadores si sufren la carga de esa deuda mientras las EPS van entrando a una lógica de oligopolio para recapitalizarse y manejar todo el negocio 

P23: 14. Transcripcion Mario Hernandez.docx - 23:13 [Claro al tratar de identificar..]  (42:42)   (Super)
Códigos:	[modelo de salud futuro] 
No memos

Claro al tratar de identificar primero como eran los costos de transacción derivados de esa intermediación financiera de las EPS, pues teníamos cifras muy desiguales en un momento dado hasta la superintendencia empezó a hablar de 50%, la contraloría de 34%, los otros no es que los costos no están realmente identificados, el ministerio dice que el 16%, bueno esas son cifras pero aun así uno podría decir, mire si uno compara esos costos de administración de los recursos con Inglaterra por ejemplo, o con Costa rica empieza uno a encontrar que las diferencias son enormes, entonces uno retira esos costos de transacción de esa manera y se concentra en una forma de atención pública territorial mucho más regulada en el sentido de que no tendría ánimo de lucro alguno, tendría que sr muy ligada a las necesidades de la población, hay una forma de calcular eso que permitirá bajar fuertemente los costos de transacción, ese es el primer elemento. Pero el segundo no es que solamente es en administración de la plata, si no en la forma en que el estado logra disminuir el impacto del gran complejo médico-industrial, sobre los costos de la operación del sistema, por ejemplo medidas de producción del medicamento es esenciales, producción nacional y ojala publica, eso ha demostrado que baja los costos enormemente, la formulación de medicamentos genéricos con un trabajo muy fuerte de política pública con los formadores con los médicos, hombre no se va a formular medicamento de marca compras conjuntas de ciertos medicamentos, obligando a las casas farmacéuticas transnacionales a bajar los costos, bueno una serie de mecanismos que se pueden hacer para bajar el costo de la atención. Y el tercer elemento es el que es muy difícil de medir el de invertir seriamente, en prevención, invertir en formas de vida saludables que implica transformar la manera en que estamos acabando con el agua,  como estamos acabando con el ambiente, como estamos generando condiciones de trabajo espantosas, que generan enfermedad y que eso no lo atiende ningún sistema de aseguramiento y eso tiene que ver con una política pública, de intersectorialidad para transformar realmente las condiciones de vida, entonces son muchos frentes los que hay que hacer y no solamente el tema de cuanto me cuesta cada píldora y como calculo la población que tengo que atender, que es un cálculo que se puede hacer que ya mas o menos existe, pero si uno no afecta todos estos otros generadores de sobre costos innecesarios en un sistema pues obviamente no va a transformar. 

P23: 14. Transcripcion Mario Hernandez.docx - 23:14 [Pues haber yo he tenido una re..]  (62:66)   (Super)
Códigos:	[modelo de salud actual] [modelo de salud futuro] 
No memos

Pues haber yo he tenido una relación muy ambivalente con ese proceso de la ley estatutaria y así ha salido en los escritos que he hecho, porque si la corte constitucional hubiera sido más coherente con la jurisprudencia y con el bloque de institucionalidad, habría anulado, declarado inexequibles unas partes muy importantes de la ley estatutaria, primero porque la ley estatutaria en principio parte de la base que es el derecho fundamental a la atención en salud, no a la salud en un sentido amplio. Sin embargo la observación 14 del comité de derechos económicos, sociales y culturales de 2000, dice con toda claridad el derecho a la salud es el logro de mayor nivel de salud posible, en la población no solamente la atención a la enfermedad y entonces claro si asumimos eso seriamente, pues los componentes esenciales del derecho a la salud incluiría por lo menos esos 13 componentes que en la misma observación plantea y nosotros recogimos en el proyecto de ley que nosotros planteamos, es decir vida digna, libertaD y autonomía, incluso derechos sexuales, etc. en salud todo el tema de no discriminación, de morir dignamente, de no ser sometido a una serie de cosas, pero además agua potable, ambiente sano, educación suficiente, vivienda digna, trabajo digno, es decir una serie de condiciones para una vida saludable además de la atención a la enfermedad y la atención de la misma, y la participación en las decisiones individuales y colectivas, entonces eso que le presentamos a  congreso lo había podido revisar la corte diciendo lo podemos presentar a ella, si no que mire quedo demasiado limitado el derecho a la salud, y por otro lado cuando ya se empieza a definir que es de la atención a la salud que es lo que es derecho está claro que el artículo 15 dice, bienes y servicios, y tecnologías y dice con toda claridad y no serán cubiertos los servicios con recursos públicos que tengan estos seis criterios y la corte acepto eso y no discutió a fondo el tema de evidencia científica sobre seguridad eficacia y eficiencia y efectividad. Y entonces claro la corte no tiene por qué saberlo pero esa es la puerta de entrada para un gran dominio medico industrial para definir que entra y que no entra y el ministerio sigue muy confiado en que va a poder aplicar unas metodologías de cálculo de costo beneficio para tratar de excluir claramente lo que no sea de costo beneficio, pero no va a afectar y no está afectando lo que genera los altísimos costos que es lo que discutimos el día de hoy en la conferencia del doctor Holguín, es decir los derechos de propiedad intelectual que es realmente lo que esta generando estos sobrecostos enormes de los medicamentos y las tecnologías a través del sistema de derecho de propiedad intelectual. Entonces pues quedo limitado, el derecho de la salud quedo limitado en la ley estatutaria, ahora con las modulaciones que le hizo la corte al proyecto de ley que salió del congreso hay algunas posibilidades, por ejemplo el hecho de que no acepta la corte de que haya un plan explícito y además una zona de priorización y además un decreto de exclusiones, no la corte muy claramente dijo solo exclusiones y retiro todo lo demás. Entonces eso se nos devuelve a la sociedad Colombiana y al sistema diciendo ¿entonces es compatible lo del POS, un POS explicito? Pues no ya la ley estatutaria dijo que no, entonces como se sostiene eso, porque seguimos insistiendo en la relación UPC , POS, y eso hay que denunciarlo y hay que demostrarlo la incongruencia jurídico constitucional de esas dos cosas de la ley estatutaria y el modelo ley 100 con una  UPC y POS . Entonces por eso digo la ley estatutaria aun con sus limitaciones tiene una posibilidades que deberíamos explotar un poco más desde las sociedad civil porque no lo va a ser el gobierno, el gobierno cree que puede aplicar la ley estatutaria con la ley ordinaria que hay. 

PC: ¿Usted cuales cree que son esas posibilidades que tiene la ley?

MH: pues mire en principio esa idea de solo exclusiones, y además uno tendría que decir con un mecanismo participativo amplio con mucha discusión pública, pues también hay que exigirlo muy profundamente porque eso fue lo que dijo la corte. Segundo el tema de los obligaciones del estado y los elementos que debe garantizar el estado como componentes del derecho fundamental que lo dijo también hoy el doctor Holguín y que sale de la observacion 14, eso está en la ley estatutaria y todos los principios que ordenan como es que el estado debe garantizar eso, con integralidad que se le dejo todo un artículo, con oportunidad con afectación de los determinantes sociales de la salud con relación con los perfiles epidemiológicos y toda esa cosa, con eficacia pero sobre todo calidad, mucha calidad en la cosa oportunidad y calidad es lo que más se viola sistemáticamente en el sistema actual, entonces tomar esos principios y mostrar como el sistema actual no logra resolver por la vía en que está organizado, pues eso es una oportunidad. También el tema de la tutela, no tocar la tutela porque es el mecanismo fundamental para que proteger cualquier derecho, no dejarnos meter en el cuento en que es que ya la ley estatutaria dijo que era solo para lo que está incluido y no para lo que está excluido, cosas así. El tema de trabajo digno, hace mucho énfasis en el trabajo digno y obliga al gobierno a estabilizar y mejorar la calidad del trabajo en salud, el tema de medicamentos en toda la cadena de medicamentos debe haber una seria de políticas para disminuir el impacto en medicamentos, eso no se está haciendo realmente, el tema de las urgencias no se necesita autorización pero para toda la atención de urgencias, pero para eso también dice en la ley estatutaria debe haber una prioridad de la inversión en promoción y prevención y atención oportuna, es decir redes integradas que realmente respondan, entonces uno tendría que presionar a los actores actuales del sistema hacia esos ordenadores digamos de la ley estatutaria. El tema de la autonomía médica entendida como el criterio medico es el que debe prevalecer en las decisiones por encima del criterio financiero y administrativo eso esta suficientemente desarrollado por la sentencia C 313 del 2014, que es la sentencia de la corte que declaro la accesibilidad parcial, entonces hay muchas posibilidades ahí, y tendríamos que ponerlas a funcionar sistemáticamente, es lo que he estado impulsando en distintas organizaciones, el asunto está en cómo ordenar todo eso y ponerlo en una forma más organizada para que la sociedad colombiana y el estado colombianos se conmueva en el sentido de que ahí hay unas implicaciones muy fuertes de la ley. 

P23: 14. Transcripcion Mario Hernandez.docx - 23:15 [No yo creo que el estado colom..]  (70:70)   (Super)
Códigos:	[modelo de salud futuro] 
No memos

No yo creo que el estado colombiano ya adopto una posición y como digo no hay una correlación de fuerzas que obligue a un cambio estructural del sistema y la verdad es muy sencilla, es simplemente es un estado regulador que trata de ajustar los contratos entre agentes de mercado y que trata de ofrecer unos incentivos para que cada uno de ellos se porte bien, en garantizar lo que dice el plan obligatorio de salud que es hasta dónde quiere llegar, y sacara muy pronto las exclusiones diciendo de eso no me encargo yo, esa es su manera de garantizar el derecho otra cosa es que sea conveniente y que sea la mejor manera desde el punto de vista de la jurisprudencia internacional. 

P23: 14. Transcripcion Mario Hernandez.docx - 23:16 [Por dos razones fundamentales,..]  (74:74)   (Super)
Códigos:	[modelo de salud futuro] 
No memos

Por dos razones fundamentales, primero porque el aseguramiento individual se centra fuertemente casi exclusivamente en el tema de la atención de la enfermedad y precisamente como el tema va concentrado los costos de esa atención en enfermedades crónicas, que implica cada vez más complejidades y más inversiones científico-tecnológicas, pues el mercado hala hacia allá está llevando las cosas hacia allá, y por eso los agentes todos prestadores aseguradores farmacéuticas pacientes, todo el mundo se concentra en ese alto costo de atención crónica, entonces claro ese modelo se concentra allí y descuida la prevención descuida la promoción, casi que hay que darles plata de antemano para que hagan prevención que es lo que va a hacer el gobierno para que hagan una gestión del riesgo en salud individual y no solo gestión financiera, del riesgo financiero mejor dicho que es lo que han venido haciendo. Ahí está la aspiración del gobierno, pero no se va a poder precisamente porque la concentración de los intereses de los recursos está en esa punta del sistema. Pero por otro lado se supone que hay unos programas que van a afectar las causas de las enfermedades antes de que se presenten, pues resulta que para eso se necesita afectar las condiciones de vida de la gente y no solamente el comportamiento individual, pues la lógica del aseguramiento se centra en la prevención y promoción a través del cambio de comportamiento de riesgos de los individuos porque todo lo demás se supone que eso lo hace el estado, pero el estado tiene otro problemita clave, y es que destruyo su capacidad institucional para hacer salud publica bien hecha, destruyo. Los municipios no tienen secretarias de salud no tienen laboratorios de vigilancia, acabaron con el área ,no tienen programas no tienen como incidir en la atención individual y con la poquita plata de intervenciones colectivas se ha dedicado a  hacer contratos para charlitas bobas y refrigerios, y se roban la plata, pero porque se destruyó la institucionalidad y la responsabilidad pública en los territorios y ahora el gobierno nacional viene a decir que por favor coordinen con las EPS de sus territorios señores. Entonces está apelando a la buena voluntad cuando destruyo la institucionalidad publica para hacer buena prevención y buena promoción, y claro le está diciendo a las EPS yo le doy más platica si usted logra gestionar el riesgo en salud de sus afiliados de los individuos y claro cuando lo hace la EPS lo hace como hacen las aseguradoras, veo como cambio el comportamiento del individuo y le pongo también incentivos a ese comportamiento, positivos y negativos. Yo sí creo que eso no es factible y no va a afectar realmente las causas de la cronificacion, la cantidad de enfermedades que existen en este momento prevenibles y mortalidad evitable que tenemos en el país

P23: 14. Transcripcion Mario Hernandez.docx - 23:17 [Pues es que mire a pesar de qu..]  (82:82)   (Super)
Códigos:	[modelo de salud futuro] 
No memos

Pues es que mire a pesar de que define la 1438 la atención primaria en salud tomando lo de la APS renovada, y toda esa cosa en el modelo colombiano donde se centra en la atención individual donde es el aseguramiento lo que prima, el aseguramiento de mercado con competencia regular en esas condiciones la atención primaria se transforma, se convierte en una estrategia de prevención individual, insisto en eso, yo le hago el control  a mi afiliado si no es mi afiliado yo no le hago ningún control es que esa es la lógica del aseguramiento, y pues trato de que no se enferme ese afiliado en concreto, ese es el límite de la atención primaria ahí. Ah no pero será familiar y comunitaria, comunitaria ni que nada un asegurador cada vez que se da cuenta que está generando externalidades incluso para una cantidad de competidores, pues va a decir no que pena yo hasta allá no llego, eso que lo haga el estado y quien es el estado, una cosa abstracta por allá porque no hay estado en el nivel territorial, ni en el municipio ni en el departamento, ahí hay unas cosas totalmente marginadas, entonces claro se va a convertir la atención primaria en una herramienta para disminuir los costos de atención y punto. 

P23: 14. Transcripcion Mario Hernandez.docx - 23:18 [Es un modelo de articulación d..]  (86:86)   (Super)
Códigos:	[modelo de salud futuro] 
No memos

Es un modelo de articulación de las distintas estrategias para tratar de alinear los incentivos de los distintos agentes, pero con obviamente el poder cada vez más concentrado en las EPS que serán menos y más grandes, y son los grandes ordenadores que seguirán allí que se supone garantizarían la gestión del riesgo.

P23: 14. Transcripcion Mario Hernandez.docx - 23:19 [Porque creen en una cosa que s..]  (90:90)   (Super)
Códigos:	[modelo de salud actual] [modelo de salud futuro] 
No memos

Porque creen en una cosa que se llama economía de escala que pueda garantizar la rentabilidad de ese negocio, porque si no los tipos se les salen del negocio como lo han amenazado varias veces si uno oye los discursos del presidente de ACEMI dice no o me aumenta la UPC o me le salgo del negocio y le dejo sus afiliados y sus pacientes ahí y los atiende usted señor estado, es una amenaza permanente porque son ellos quienes dominan el asunto. Y por eso el gobierno dice no es que las EPS no están en discusión, dice si quitamos las EPS quien va a hacer eso, no hay institucionalidad para hacer eso. Ese es el problema gravísimo que tiene, con la destrucción de la institucionalidad pública solo quedamos en manos de la benditas EPS y son las reglas que ellas digan que nos van a obligar a hacer cosas.

P23: 14. Transcripcion Mario Hernandez.docx - 23:20 [Pues es que hay dos cosas, por..]  (94:94)   (Super)
Códigos:	[modelo de salud actual] [modelo de salud futuro] 
No memos

Pues es que hay dos cosas, por un lado se ha planteado es que los pobres no demandan mucho porque aprenden a sufrir cosas así, totalmente idealizada en cambio los que contribuyen y pagan mes a mes, tienden a pedir más. Eso es una lógica que viene hace rato los análisis de demanda de salud, el riesgo moral es que cuando yo estoy pagando todos los días quiero consumir eso que pague, en cambio si no pago no voy a consumir tanto de eso. Mire eso es una perspectiva demasiado individualista y demasiado ligada al egoísmo supuestamente natural de los modelos económicos, eso oculta lo que esta pasando realmente atrás y es que finalmente las formas como se han desarrollado esos dos regímenes hace que al pobre se le trate como a pobre, la información es pobre, la accesibilidad es pobre, las barreras de acceso cada vez son mayores y como el pobre tampoco reclama porque le reglan y si reclama de pronto se lo quitan, la lógica del subsidio de la calidad entonces va generando esta idea de que allá los pobres tienen menos y acceden menos, mientras que los del régimen contributivo se le tratan como medios pero todo el tiempo le están diciendo, sabe que si usted quiere mejorar la oportunidad la calidad, pague un poquito más, sigue siendo del régimen contributivo pero si paga una medicina prepagada lo atendemos adecuadamente. Y entonces alli se empieza a generar una demanda cada vez más frecuente más grande en el régimen contributivo, que en el régimen subsidiado, los de medicinas prepagadas son del régimen contributivo porque les obligan a pagar el régimen contributivo, pero usan más frecuentemente, entre más plata pongan más frecuentemente con mayor calidad con mayor oportunidad, con todo lo que usted quiera. Entonces por eso hay diferencias entre el régimen contributivo y el régimen subsidiado, el riesgo moral de la sobreutilización de cuando pago y la subutilización cuando no pago, que es donde nos han venido concentrando la explicación y yo creo que eso hay que estudiarlo con más profundidad. 


P23: 14. Transcripcion Mario Hernandez.docx - 23:22 [Yo creo que en principio va a ..]  (102:102)   (Super)
Códigos:	[modelo de salud actual] [modelo de salud futuro] 
No memos

Yo creo que en principio va a continuar, es que finalmente al subsidiado se le trata y se le pone una cantidad de barreras que no creo que vayan a cambiar porque se supone que ahí hay menos plata, la UPC es un poco menor, no está ajustada a riesgos y eso lo han aprovechado bastante bien las EPS del régimen subsidiado, entonces no creo que estén presionando para que haya más demanda de servicios. Pero por otro lado también está el tema de la prevención de la promoción toda esta cosa, no creo que baje mucho la demanda y la utilización de servicios si sigue concentrado en esa perspectiva individualista tan fuerte, puede que logren las EPS bajar un poco la demanda del segundo y del tercer nivel, los tales servicios complementarios, como lo llaman con la estrategia de la medicina familiar que será el contenedor de ese ascenso, entonces es posible que baje la tasa de hospitalización que hoy es la más alta de américa latina, es posible que bajen algunas cositas por ejemplo, cesáreas pues si fuera coherente como dice la OMS debería ser el 15% máximo de los embarazos se complican, el resto debería ser parto normal y habría que estimular las condiciones y los medios y los recursos para que hayan partos normales, pero pues no, porque la plática se concentra en las cesáreas es mucho más fácil, la paga es mejor, las mismas señoras ya aprendieron a pedir eso como condición universal y pues eso tiene más riesgo para el niño, para la señora, para todo el mundo que un parto normal, pero bueno es la lógica del mercado la que impulsa eso. 


P23: 14. Transcripcion Mario Hernandez.docx - 23:23 [pues la verdad es que no es po..]  (106:106)   (Super)
Códigos:	[modelo de salud futuro] 
No memos

pues la verdad es que no es por una falta de ejemplos en el mundo, al contrario si uno incluso tomando ese informe de la OMS del desempeño de los sistemas de salud en el mundo, en donde salió tan bien librada Colombia porque el modelo de evaluación fue tomado del pluralismo estructuralizado, eso sí se los mostré claramente a Juan Luis Londoño y me dijo que yo estaba exagerando y que estaba con un problema de timing, pero ese es el punto aun en ese informe se muestra con claridad que tienen mejor resultados en salud aquellos sistemas que son de fondo publico único, como Inglaterra, como Canadá, como los países nórdicos, y gastan mucho menos que los que tienen un predominio de mercado como Estados Unidos y otros. Entonces la apuesta de este sistema es hacer la mejor articulación entre estado y mercado, supuestamente la gran frase de Santos, el uno y el otro hasta donde sea necesario, es como la idea fundamental. Pues no, si uno va a Costa Rica, tiene un sistema de seguridad social, una caja de seguridad social con 85% de cobertura y tiene todavía una cantidad de gente que no entra por una lógica de lo formal y lo informal en el trabajo, y aun así han logrado por otros sistemas resolver. Pero ese modelo de una caja única de seguridad social , que se baja y se administra en el nivel territorial, tratando de hacer atención primaria, articular incluso con el tema de salud pública, una serie de mecanismos para tratar de articular eso, y responder a las especifidades de la población del territorio, eso ha sido mucho ms eficaz que los otros modelos que hemos venido desarrollando, tanto el mercado de Estados Unidos como este de competencia regular que tiene Colombia, entonces se puede demostrar eso, se pueden traer los ejemplos, entones no es por falta de ejemplos, ni falta de iniciativas ni de propuestas, es porque la estructura de un sistema de salud de un país, depende de la correlación de fuerzas y como se da eso en las trayectorias históricas de cada país, y eso entones define hacia dónde va mi prospectiva teniendo en cuenta los 20 y pico de años que lleva este modelo, es que se va a consolidar y cada vez más va ser utilizado por el gran complejo médico-industrial y por los organismos internacionales, como el ejemplo de cobertura universal en salud, con una buena combinación de estrado-mercado, así lo dicen la OMS, todos estos organismos nos muestran como el gran ejemplo, porque es bastante funcional a la acumulación de capital en ese sector, nacional y transnacional y al mismo tiempo logra contener las inequidades y los impactos fuertes que tiene sobre la sociedad, con el régimen subsidiado con unas mínimas regulaciones, con esa regulación de la competencia que es la lógica UPC, POS;  esos son regulaciones de mercado. Entonces como contiene e impulsa el capital, pues es el modelo perfecto, cambiar eso es muy difícil- 


P23: 14. Transcripcion Mario Hernandez.docx - 23:25 [Yo creo que sí, las están incl..]  (110:110)   (Super)
Códigos:	[modelo de salud futuro] 
No memos

Yo creo que sí, las están incluso liquidando golpeando y toda esa cosa, la superintendencia ya fue a la Guajira, y dijo si 10 no me cumplen de las 17, y las van a eliminar para irlas concentrado hasta quedar con una, para que sea esa 1 que atienda a todos. Pues dicen que la indígena, para régimen subsidiado, y para el régimen contributivo van a poner a la caja de compensación que manejan los políticos allá, entones es la forma de disminuir, pero igual están molestando para liquidar un montonón de estas y si se sale el negocio entones si quiere continuar, capitalice a otra grande y venga volvamos a hacer socios, cada uno está armando su gran grupo económico, Luis Carlos Sarmiento Angulo ya quiere invertir ahí, y ya está ofreciendo para comprar cafesalud, ya tiene todo lo de pensión ya tiene todo lo de cesantías, tiene todo el sector financiero, tiene un poconon de negocios en todo lado entonces ya casi va a entrar

P23: 14. Transcripcion Mario Hernandez.docx - 23:26 [Pues la única forma de ir cons..]  (118:118)   (Super)
Códigos:	[modelo de salud futuro] 
No memos

Pues la única forma de ir construyendo algo distinto es si se ponen al debate público el tema de salud de otra manera, y cosas como la ventana de oportunidad que se abre con el tal acuerdo de paz y el post acuerdo pues tendrían que discutir cual es el lugar de la salud en la construcción de la paz, la necesidad de que la academia se lance mucho más a la discusión pública, la articulación entre diferentes organizaciones sociales que puedan efectivamente aglutinar unas necesidades de cambio, porque es la única forma, es la movilización social la única que podría transformar el estado de cosas. 


P24: 15. Transcripcion Martha Balbuena.docx - 24:2 [En relación con las políticas ..]  (7:7)   (Super)
Códigos:	[modelo de salud futuro] 
No memos

En relación con las políticas de seguridad social en salud, pienso que cambiaría todo empezando por la conceptualización del equipo de salud, porque nosotros hablamos de un sistema de seguridad social en salud, donde están las entidades prestadoras de servicio, las EPS yo sé que eso no se puede cambiar pero al menos que se disminuyeran a 2 o 3, pero que realmente cumplieran con el objetivo que tiene una empresa prestadora de servicios, porque la empresa prestadora de servicios se confiera como la intermediaria entre el ministerio y esta IPS. Pero que es lo que nosotros estamos viendo algunas de las personas que tomamos parte de las juntas directivas, la IPS presta servicio de salud pero la EPS no le paga con oportunidad, es una de las cosas es que se conserve esas EPS pero que su forma de ética fuera realmente pagar cuando se cobre el servicio que estén bien diligenciadas, eso sería uno

P24: 15. Transcripcion Martha Balbuena.docx - 24:4 [LA estrategia de atención prim..]  (18:18)   (Super)
Códigos:	[modelo de salud futuro] 
No memos

LA estrategia de atención primaria como se concibió en algunas instituciones, en ese momento se empezó a conformar equipos para hacer toda esta parte de promoción y prevención de la enfermedad pero cuando se iniciaron esos equipos se hablaba básicamente solo del auxiliar de enfermería, no era el equipo como lo concibo yo, un equipo donde este el medico el médico familiar, todo el equipo de salud como la terapista la enfermera, el odontólogo, la trabajadora social, que realmente puedan impactar en un barrio en una comuna, pero que el gobierno no sé si habrá entendido que solo con 2 o 3 personas no se puede hacer y tampoco pueden crear un solo equipo para 20000 habitantes, entonces tiene que ser y uno tiene los parámetros por cada equipo de atención primaria ellos podrían atender tantas personas.

P24: 15. Transcripcion Martha Balbuena.docx - 24:5 [En cuba uno se queda aterrado ..]  (22:22)   (Super)
Códigos:	[modelo de salud futuro] 
No memos

En cuba uno se queda aterrado de que el CDR es por manzana, dígame usted cuantas familias estarían allí y no son familiar grandísimas ósea que es mucho más poquito. Acá pues podrían llegarse a eso pero que implicaría, todo un cambio total de este régimen.  La ley lo manda pero no se hace, llevamos 5 años y no se ha hecho

P24: 15. Transcripcion Martha Balbuena.docx - 24:6 [No, Lo que pasa en este moment..]  (26:26)   (Super)
Códigos:	[modelo de salud futuro] 
No memos

No, Lo que pasa en este momento es la necesidad es el crear, ese médico familiar y crear ese médico familiar con otras profesiones, pero como se llega a aplicar y los costos que van a hacer de pronto dicen que es muy costoso no vamos por allí, entonces volemos a lo mismo, no es que esté hablando que la promotora no debe estar se acabó pero hacia algo, el auxiliar de enfermería también pero en ese momento era el médico y la enfermera ahora en este momento que uno ve en algunas comunas de Cali, entonces ya está el odontólogo, la enfermera el terapeuta, el médico general, pero tienen una población tan grande que no son capaces de cubrirla. Ahí yo pienso que cuando el ministerio o la secretaria se une a la parte académica podría al menos formarse un equipo mayor que podríamos empezar muy despacio, porque el grupo académico esta por un periodo porque tiene un relevo de un mes, los otros estrían continuamente, pero si se lograra hacer esa integración para que la academia entre comillas pudiera seguir como lo hacemos nosotros con los internos en las instituciones de salud, que eso se continuara en lo que nosotros vamos a hablar de atención primaria es importante

P24: 15. Transcripcion Martha Balbuena.docx - 24:7 [Yo diría, empezaría lo que ust..]  (50:50)   (Super)
Códigos:	[modelo de salud futuro] 
No memos

 Yo diría, empezaría lo que usted me dijo una vez, cogería un grupo de una población y empezaría a medir con las familias a ver cuáles son los resultados que uno sabe que epidemiologicamente eso en un año podemos ver un cambio y de ahí yo empezaría inicialmente en los lugares más apartados que hemos trabajado, yo lo trabaje en Jamundí mucho tiempo, en las veredas y trabajando medico promotoras y enfermeras y no más, y mire usted el cambio que hubo , eso fue en el año 83, 84. 

P24: 15. Transcripcion Martha Balbuena.docx - 24:11 [Yo digo si a usted no lo busca..]  (78:78)   (Super)
Códigos:	[modelo de salud futuro] 
No memos

Yo digo si a usted no lo buscan, hay que ir allá entonces si tenemos médicos que están dentro de la institución que digo yo dejaría en la institución a el especialista el otro médico al médico general lo pondría con un equipo a ir allá, a las instituciones va a llegar lo que tiene que llegar el medico está en el barrio y el habitante sabe que va a llegar entonces no viene acá y no va a congestionar, mire doctor que en estos días fui al valle del Lili, se abrió a atender a todo el mundo pero está peor que el universitario, ahora hay muy poquita gente no hay forma y en el valle del Lili está lleno porque hicieron muchos contratos pero no están dando una atención oportuna. Están en los corredores los pacientes. Entonces yo digo si lográramos es decir que las IPS, pero eso es una parte de educación tanto para toda la gente de salud como para la comunidad se acuerda cuando estaba lo de remisión que la gente llegaba a cualquier parte pero luego se educó, pero después eso se fue perdiendo porque aparecieron los demás y decían vaya a este sitio y usted  o este otro y eso se volvió, cuando estaba ese régimen de remisiones era bueno, hay mucha congestión porque la gente le falta educación. 

P24: 15. Transcripcion Martha Balbuena.docx - 24:12 [Si cada persona hace las cosas..]  (94:94)   (Super)
Códigos:	[modelo de salud futuro] 
No memos

Si cada persona hace las cosas bien como las tiene que hacer nosotros cada uno está cuidando lo que estamos haciendo, eso es otra cosa que trabajamos mucho para poder entender que toda la calidad del sistema sea bien, pero eso depende de cada uno de nosotros por eso digo que las auditorias para ver si se está trabajando bien, estoy de acuerdo con todos los proceso pero eso depende de cada uno de nosotros y la gente tiene que estar muy metida para poder saber qué es lo que tiene que hacer. Por ejemplo si tengo que hacer la misión de un paciente a un quirófano están todos los procesos y eso es calidad del proceso para que todo salga bien pero si no lo conozco, no lo hago bien entonces vuelvo a lo mismo cada vez que implementemos calidad en lo servicios de salud en cualquier parte tenemos que capacitar a la gente, para que la gente lo que le toque hacer lo haga bien porque somos como una cadena donde un eslabón se parte y se dañó todo ese círculo.

P24: 15. Transcripcion Martha Balbuena.docx - 24:13 [Si cuesta, pero si nosotros em..]  (98:98)   (Super)
Códigos:	[modelo de salud futuro] 
No memos

Si cuesta, pero si nosotros empezamos acá en la universidad por lo menos es lo que es esta mirando, las políticas institucionales están diciendo necesitamos mejorar investigación donde nosotros nos están evaluando y yo le decía doctor usted nos está diciendo que tenemos que hacer investigación pero para hacer tenemos que partir de que hay gente que le gusta y otros que no les gusta entonces empecemos a escoger y capacitemos la gente que le gusta y esa gente será un motor para los demás, Pero no puedo meter a todos que tienen que hacer investigación y escribir y publicar, primero capacitémonos y luego ver que eso va a mejorar. Entones yo pienso que hay que capacitar primero a las personas que la gente participe en esos procesos cuando la gente ha participado la gente no entiende que le van a imponer y que el gobierno no quiere que salgamos de esto, y de pronto las personas que queremos hacer algo, lo que usted me decía yo me puse a pensar, yo voy a estar en la comuna 18 y pues de ahí tiene que salir un proyecto de investigación, donde no lo podemos dejar en Cali. 

P26: 16. Transcripcion Senador Ospina.docx - 26:4 [Se debe cambiar la relación qu..]  (11:11)   (Super)
Códigos:	[modelo de salud futuro] 
No memos

 Se debe cambiar la relación que hoy se concentra en salud entre unos prestadores de salud y unos administradores o afiladores de usuarios, porque esto es una relación que es meramente basada de una forma económica en quien le presta el servicio a la comunidad y en quien afilia a la continuidad no tiene en cuenta los entornos ambientales n las acciones territoriales ni las acciones preventivas, no las acciones de promoción de hábitos saludables, ni la intersectorialidad como herramienta. Un sistema de salud que se base entre prestadores de servicios y afiladores es un sistema de salud como camino al fracaso.


P26: 16. Transcripcion Senador Ospina.docx - 26:5 [Yo trataría de entender que el..]  (15:15)   (Super)
Códigos:	[modelo de salud futuro] 
No memos

 Yo trataría de entender que el sujeto es un sujeto que tiene un entorno social donde desarrolla su vida, es un sujeto que tiene un territorio donde vive, es un sujeto que se encuentra en una transición demográfica y no se puede perder este bono que la transición demográfica nos posibilita tener en términos de productividad entendería que estamos en un tema de cambio climático y de nuevas enfermedades transmitidas por sectores que aplican y entendería que no podemos estar sujetos a tener en el sistema un medio económico sino que estaremos sujetos a preservar la vida de los ciudadanos. Bajo esa logia que haría, buscaría que las empresas promotoras de salud segmentaran territorialmente sui trabajo, buscaría que la relación entre empresas promotoras de salud y prestadores de servicio fuera mucho más estrechas, e incluso pensaría en integraciones integrales buscaría que la tarea de los entes territoriales en términos de intersectorialidad obligara a las EPS  participar de la misma, buscaría una regulación nacional en cuanto a tarifas, de tal forma que no haya una explosión de tarifas, si no por el contrario un uso eficiente del recurso, y buscaría que por ley una mayor proporción de recursos se adelantara efectivamente en términos de prevención de enfermedad y promoción de salud.

P26: 16. Transcripcion Senador Ospina.docx - 26:6 [El sistema, los gastos en salu..]  (19:19)   (Super)
Códigos:	[modelo de salud futuro] 
No memos

El sistema, los gastos en salud no son gastos ocasionales sino que son gastos recurrentes, eso significa que uno no resuelve la crisis de la salud con la implementación de un recurso extraordinario en una ocasión y no dejarlo de prestar. En ese sentido se tiene que ser más eficiente con los recursos que hoy se tienen y se deben de identificar fuertes alternativas de crecimiento progresivo para las futuras demandas en servicio de salud, para yo tratar de evitar el colapso y entendiendo que las EPS tienen hoy grandes pérdidas trataría de cambiar la relación que hoy existe donde le ciudadano está sujeto a una sobreoferta de servicios de prestador y a una restricción de servicios por parte de la EPS. Entonces para que esa relación se cambiara yo tendría que adelantar toda una acción pedagógica y una acción de control para evitar la sobre oferta de servicios a lo que está sujeto el paciente. Lo que yo entiendo es que como todas las entidades tienen ánimo de lucro como los prestadores de servicio como las EPS, cuando el paciente llega al hospital o la clínica la clínica satura al paciente de servicios sin importar los costos de los mismos porque tiene un interés económico en esta acción, pero como la EPS tiene ánimo de lucro restringe servicios para tratar de sobrevivir en el sistema. Sacar a los ciudadanos de esas, demanda un rediseño institucional que debe adelantarse. Pero además de eso buscaría que la capitalización de las EPS se puedan hacer con cargo a recursos hoy existentes en todo el sistema, si permitimos que hospitales y crónicas sean partícipes de EPS y sean parte de ese patrimonio yo podría solventar la ecuación financiera que el ministerio propone. También trataría de evitar que los costos en la prestación de servicios estén cada vez más altos y para eso haría una política nacional de formación de talento humano médico, o de profesionales asociados a la salud para tratar de reducir la escasa oferta que existe en ciertas especialidades. Todo ello para tratar de una forma macroeconómica reducir las presiones económicas que tiene el sistema por falta de cirujanos cardiovasculares, o neurocirujanos o pediatras con incentivos a aquellos especialistas que vayan a trabajar en la periferia, y adicional mente trataría al máximo de brindar importaciones directas de medicamentos e insumos reduciendo el arancel a 0 para poderlos proveer a la prestación.

P26: 16. Transcripcion Senador Ospina.docx - 26:7 [Lo hice más o menos con este s..]  (23:23)   (Super)
Códigos:	[modelo de salud futuro] 
No memos

Lo hice más o menos con este sentido, gran cantidad de cirugía estética y plástica realizada en condiciones no apropiadas, le generaba al hospital universitario grandes costos en las complicaciones, este alto costo en la atención de las complicaciones al interior del hospital me decía que los recursos del hospital no solamente se tiene que evaluar en términos de los ingresos sino que también en términos de reducir los gastos y por eso si hiciéramos cirugía estética y plástica y de buena calidad podríamos reducir lo que nos está a estallando el hospital dado que al hospital universitario publico era donde trasladaban a todos estos pacientes con estas complejidades. También lo hice entendiendo que en el sistema de seguridad social en salid, no está la cirugía estética y plástica y ella podría generar un flujo de caja distinto al sistema, entonces pensé si pudiéramos montar un modelo estandarizado de cirugía plástica a bajo costo podríamos encontrar una participación de recursos que no van a llegar por la manera que el sistema funciona, y también lo hice entendiendo que un hospital que forma talento humano también tiene que darse la oportunidad de proveer a los de menos recursos de ese tipo de proyectos, la sumatoria de estas 3 tareas nos ha dado la oportunidad de efectivamente ser razonable mente exitosos y nos ha dado a entender que el sistema del hospital funciona, lo hice también con otro tipo de subespecialidades con una orientación parecida. Cuando también se hizo un aprovechamiento mejor de los recursos mejor que podríamos capturar por accidente de tránsito, entonces se hizo un proyecto denominado trauma para que pudiéramos atender con oportunidad al accidentado y facturarle satisfactoriamente a los aseguradores. La sumatoria de estos recursos más el aprovechamiento de lo que prestábamos a las EPS nos permitió tener un hospital estable.


P26: 16. Transcripcion Senador Ospina.docx - 26:8 [En Colombia somos avanzados en..]  (34:34)   (Super)
Códigos:	[modelo de salud actual] [modelo de salud futuro] 
No memos

En Colombia somos avanzados en las leyes con dificultades de aplicación de la misma, la ley estatutaria en salud, y lo que tenemos nosotros en tema del derecho a alas salud como un derecho constitucional robusto y la manera como la corte y todos hemos protegido ese derecho, es de lo más avanzado que existe en Sudamérica y de lo más avanzad que existe en el mundo me parece a mí que se trata del punto a donde llegar y un punto de partida en temas de solides en principio del sistema, pero difícil de aplicar en la manera que tenemos diseñado el modelo

P26: 16. Transcripcion Senador Ospina.docx - 26:9 [Yo creo que parte del éxito de..]  (38:38)   (Super)
Códigos:	[modelo de salud futuro] 
No memos

Yo creo que parte del éxito de una sociedad es tener referenciado y escrito lo que son los fundamentos y si una sociedad tiene escrito a nivel constitucional unos derecho de salud como los que nosotros tenemos, necesariamente van a ser cumplidos, van a haber procesos de transición difícil de leer pero todos los días creo que la sociedad colombiana va a aplicar mayor cantidad de recursos para el éxito cierto de este derecho a la salud. 

P26: 16. Transcripcion Senador Ospina.docx - 26:10 [Si, es decir yo creo que en el..]  (42:42)   (Super)
Códigos:	[modelo de salud futuro] 
No memos

Si, es decir yo creo que en el papel es una estrategia muy interesante, creo que recuperar la estrategia de atención primaria en salud como herramienta es ir pensando distinto a lo que anteriormente existía y si me preguntan a mi si es un avance frente a lo que anteriormente existía, yo lo veo así, anteriormente ni siquiera existía un bosquejo de ese otro, se trata de identificar muy bien los aliados territoriales para ir implementando esta estrategia.


P26: 16. Transcripcion Senador Ospina.docx - 26:11 [Yo creo que uno de los problem..]  (46:46)   (Super)
Códigos:	[modelo de salud futuro] 
No memos

Yo creo que uno de los problemas es que el gobierno está contra el tiempo, una de las dificultades que se tiene es que la liquides del sistema agota al sistema, y que tocara tomar medidas drásticas para hacer un alto en el camino y reorientar hacia el MIAS lo que es el nuevo enfoque en salud en Colombia.

P26: 16. Transcripcion Senador Ospina.docx - 26:12 [Reorganizar los presupuestos, ..]  (50:50)   (Super)
Códigos:	[modelo de salud futuro] 
No memos

Reorganizar los presupuestos, orientar por ley mayor presupuesto para la atención primaria en salud, rediseñar la relación entre la academia y los prestadores para formar más talento humano de manera inmediata, precisar la nueva categoría que deben tener las nuevas empresas promotoras de salud, rediseñar las relaciones que se tienen con los entes territoriales, territorialsacion de la salud es fundamental.

P26: 16. Transcripcion Senador Ospina.docx - 26:13 [En este momento nosotros estam..]  (54:54)   (Super)
Códigos:	[modelo de salud futuro] 
No memos

En este momento nosotros estamos en algo que se llama una transición demográfica que nos da un bono pensional, eso significa que todavía no estamos en una población mayoritariamente envejecida y tampoco estamos en una población mayoritariamente joven y menor de edad, nos encontramos con un engrosamiento de la pirámide poblacional donde la mayor proporción se encentra en hombres y mujeres jóvenes, dado que esa mayor proporción se encuentra en hombres y mujeres jóvenes, ellos todavía no son altamente demandantes de servicios de salud, si no que al contrario pueden proveer de fuerzas productiva y de recursos a la sociedad colombiana en su conjunto, la sociedad debe entender este momento que se está atravesando para no solamente aprovecharlo en términos de pensiones futura, si no para aprovecharlo en términos de ahorro e inversión efectiva en salud. Estoy seguro que si se adelanta esto de esa manera la situación va a funcionar.

P26: 16. Transcripcion Senador Ospina.docx - 26:16 [Ese es un tema muy contradicto..]  (70:70)   (Super)
Códigos:	[modelo de salud futuro] 
No memos

Ese es un tema muy contradictorio pero yo tengo que plantearlo así, debe ser políticos pero entendido en lo que significa la palabra político, no politiquero el término que se hable de político es que un gran gerente de hospital debe ser un aglutinador de toda la sociedad alrededor de la institución que lidere, que tenga la capacidad de liderar y de encontrarse con la academia y de liderar y encontrarse con el cliente interno trabajador, profesionales de la salud y encontrarse con las autoridades municipales y departamentales para financiación del mismo y una gran capacidad de negociación frente a las empresas promotoras de salud. Si un gerente es un politiquero que va orientado a la burocratización de la institución a la corrupción como camino al clientelizacion de la institución, acabara con el hospital de manera inmediata, pero si se trata de un técnico o tecnólogo que no lea la capacidad de dialogo como herramienta podrá hacer bien la tarea pero no tendrá los socios para poder liderar el hospital a un buen puerto. Los directores de hospitales pueden y deben ser por concurso esto está muy bien, pero no sirven si son por concursos amañados o por si adicionalmente no responden a lo que deben ser las directrices de la autoridad local. Uno preferiría que hospitales de primer nivel, segundo y tercero, sean elegidos por la autoridad local para que los mismos hagan el matrimonio indisoluble que conlleva realmente la prestación de servicio de salud.

P28: 17. Transcripcion-Gustavo-Morales (1).docx - 28:6 [ahora lo que vamos a debatir e..]  (15:15)   (Super)
Códigos:	[modelo de salud futuro] 
No memos

ahora lo que vamos a debatir es que vamos a segura salud con un sistema de aseguramiento, los sistemas de salud del mundo originales al menos aseguraban a aquello que es un riesgo financiero para las personas y las familias, la enfermedad catastrófica, el accidente, la enfermedad que limita tu trabajo. Quizás el aseguramiento no deba seguir protegiendo las grupas, ni los embarazos ni la torcida del tobillo, porque eso no es tal vez un riesgo que signifique catástrofe financiera para las familias, entonces yo creo que un sistema de aseguramiento con una precisión mejorada de que estamos asegurando es el camino a seguir.

P28: 17. Transcripcion-Gustavo-Morales (1).docx - 28:7 [Yo creo que uno, debe cambiars..]  (19:19)   (Super)
Códigos:	[modelo de salud futuro] 
No memos

Yo creo que uno, debe cambiarse la injerencia de las entidades territoriales en el sistema de salud, porque salvo una o dos, nuestras entidades territoriales no tienen la fortaleza técnica ni financiera para administrar los aspectos del sistema que le han pedido que administren. Dos debe acabarse la injerencia política en el sistema de salud, y acá hablo con un poquito de autoridad moral porque la superintendencia financiera ha logrado estar blindada de la presión política, porque la superintendencia de industria y comercio ha estado blindada contra la política y en cambio la supersalud no, porque los políticos todavía ven la supersalud como un lugar donde pueden ir a pedir puestos y contratos. Eso no tienen ningún sentido, entonces yo creo que las injerencias de los políticos deben acabarse en la supervisión y en la prestación la mayoría de los hospitales públicos siguen liderados por los clanes políticos de las regiones y eso hace que no funcione el sistema de salud. Yo diría que esas son las dos cosas principales que deben cambiar. Y tal vez si me permites una más, creo que por lo menos hay que abrir el debate de la integración vertical. Acá se ha estigmatizado la integración vertical, pero si ves, por estigmatizar una integración vertical estamos abriéndole por la puerta de atrás a otras, restringimos que las EPS fueran dueñas de IPS, pero en cambio hay IPS que están siendo dueñas de EPS, y que mayor integración vertical que la que vemos en Bogotá donde todo el régimen subsidiado esta administrado por el distrito y además son los dueños de los principales centros de prestación del servicio. Entonces cuando hablamos de integración vertical de que estamos hablando, ese tema lo tiene que discutir el país

P28: 17. Transcripcion-Gustavo-Morales (1).docx - 28:8 [Pues yo puedo decir que en est..]  (23:23)   (Super)
Códigos:	[modelo de salud futuro] 
No memos

Pues yo puedo decir que en este 2018 arrancamos de un buen punto de partida, una cobertura del 98% un consenso general donde son más los avances que los retrocesos, un reconocimiento internacional por lo menos de la OSD de que nuestro sistema es el mejor de américa latina, es un buen punto de partida para mejorar lo mejorable, por supuesto hay algunas propuestas policitas que quieren tirar todo por la borda y arrancar de 0, eso sería un error histórico, yo creo que hay que construir sobre el aprendizaje y tenemos ventajas en comparación con el sistema gringo, ellos no tienen cobertura universal y le apostaron al aseguramiento privado, nosotros ya estamos en cobertura universal y yo creo que ahora, regulemos los entes privados con fuerte regulación para que esa cobertura universal se traduzca en la prestación del servicio. Entonces en los próximos 20 años veo un sistema de salud donde puede abrase un poco más a la competencia privada, dejarlos que innoven en prestación, en calidad y veo un sistema que va a enfrentar tres desafíos muy importantes. Los 3 desafíos que yo veo, son uno muy pronto va a tener que resolver sus temas de financiación para que sea estable, siempre va a haber un déficit no hay un solo sistema de salud 100% sostenible en el mundo, pero lo que no puede pasar es que la financiación básica del sistema este dependiendo del ciclo económico, entonces ahí hay un problema por resolver. El otro es como vamos a integrar la inteligencia artificial al sistema de salud, big data, análisis epidemiológicos, pronósticos, bueno todo lo que la Inteligencia Artificial pueda hacer. Tres, veo un sistema de salud donde los hospitales van a dejar de ser los centros del sistema los sistemas de salud hoy en día se han construido muy en torno a la idea de hospital, y alrededor de eso se ha construido el sistema, pero la tecnología va a desbaratar el concepto de hospital como lo vemos hoy en día, lo que no vemos es cuál es el sistema. Mira el sistema como Kaiser permanente que se usa en Estados unidos, ya el 60% de las consultas médicas se hacen por correo electrónico. Entonces ya no necesitas consultorios y no los necesitas en gran calidad, y finalmente como lo decía ahora creo que si abrimos las puertas para la innovación y no solo la innovación para las tecnologías, es muy importe y lo vamos a hablar después, sino la innovación en la prestación vamos a tener un sistema muy distinto y la gran pregunta que hay que hacerse es esta, como hacemos que el campesino de Tumaco o el campesino, coxca en bolívar tenga acceso a los servicios de salud, mínimos, básicos, dignos y de calidad. Ese problema ya no lo tienen los que habitan en Cali, o en Medellín o en Bucaramanga, mal que bien que les toca hacer fila y esperar van a hacer atendidos. El problema es el que vive en boca grande en Tumaco, ese muchacho allá pescador o viejo de 60, el tema que el puesto de salud le queda de 4 o 5 horas, no será que si invertimos un poquito en tecnología satelital e internet ese señor no va a tener acceso a salud sin necesidad de tener que desplazarse, y ese es un tema de infraestructura, pero no hospitalaria que es de lo que todo el mundo habla, sino en infraestructura digital para la salud, esas son como las grandes macro tendencias que veo en el sistema de salud.


P28: 17. Transcripcion-Gustavo-Morales (1).docx - 28:9 [Yo promovería un sistema de sa..]  (27:27)   (Super)
Códigos:	[modelo de salud futuro] 
No memos

Yo promovería un sistema de salud donde todos los colombianos sin distinción se les garantiza unas prestaciones básicas principalmente orientadas a evitarle el riesgo catastrófico. Eso puede implicar que a muchos colombianos se les reduzca su plan de beneficio, pero se le ofrezca algo al resto de colombianos. En estos momentos el habitante de la clase media baja de Bogotá tiene acceso a un sistema muy generoso de salud, pero el campesino del Guaviare no tiene absolutamente nada, como hacemos para igualar eso es el gran desafío, veo un sistema donde haya competencia entre las EPS, hoy en día no la hay, hoy en día a todas le pagamos lo mismo para que ofrezcan el mismo plan de beneficio y no distinguimos entre calidad del servicio. Porque sanitas recibe lo mismo que Medimas, cuando sabemos la diferencia de calidad del servicio es distinta. Entonces yo creo que ahí se debe trabajar por resultado. Y veo un sistema de salud donde estamos pensando en volvernos que no todo venga de afuera, en este momento todos los dispositivos, todos los medicamentos viene de afuera, acá se acabó la política industrial de dispositivos y medicamentos entonces quisiera ver como en Brasil o en Méjico, una política que promueva los medicamentos desde adentro entonces ese sería mi sistema soñado.

P28: 17. Transcripcion-Gustavo-Morales (1).docx - 28:12 [Depende de que pedazo de la po..]  (42:42)   (Super)
Códigos:	[modelo de salud futuro] 
No memos

Depende de que pedazo de la política estamos hablando, el pedazo de control  de precios que mencionamos anteriormente contribuye de manera importante, hay productos que se les baja el precio en un 80% eso está bien porque no, pero como bien lo decías al formular la pregunta, todo lo que se saque en esa metería te va a resolver el 13% del problema incluso hay un dato más agudo, el 13% son la totalidad del medicamentos pero los medicamentos innovadores de alto costo que son a quienes están dirigidas estas políticas de control de precios no representan mas del 3% de gasto en salud, entonces todos estos esfuerzos validos solo se refieren al 3%. Yo lo que creo es que hay que continuar con esa política, con otro brazo de la política que se llama la política de compras centralizadas que es muy positivo, con la política de evaluación tecnología para que el sistema no esté pagando cosas que no sirven, todo eso está bien pero se ha llegado a un extremo donde se empezó a cuestionar las patentes, pero lo que yo creo es que el país debe decir ahora y el 87% del gasto restante que vamos a hacer con eso, este documento que te doy son nuestras propuestas para el 87% restantes, muchas de las cuales ya hemos mencionado aquí y otras que te van a abrir el apetito también. 

P28: 17. Transcripcion-Gustavo-Morales (1).docx - 28:13 [Esa es una preocupación que te..]  (46:46)   (Super)
Códigos:	[modelo de salud actual] [modelo de salud futuro] 
No memos

Esa es una preocupación que tenemos, lo que es bueno una filosofía de agregación donde por volumen se tiene a bajar el precio si eso se hace con transparencia con reglas claras y donde todos los posibles oferentes pueden ofertar, quien se va a oponer a eso. La duda que si tenemos en la implementación es si Colombia compra eficiente que es una entidad creada para comprar papeles y computadores y gasolina, tiene la experticia para manejar productos complejos como los medicamentos y la respuesta es que no. Si uno fuera a coger la política farmacéutica y dijera que está bien y que está mal la compra centralizada está bien, pero hay que tener cuidado con la implementación porque sobre todo cuando se trata de productos de alta complejidad, la sustibuilidad o lo que se llama la intercambiabilidad no es automática, tu puedes cambiar un computador Dell o Lenovo por un hp y no es mayor cosa lo que pasa, pero si cambias un producto de cáncer A por el producto B aunque sea el mismo principio activo puede haber riesgos, ese estudio previo es el que nos preocupa que no exista cuando Colombia compra eficiente se embarque en compras centralizadas.

P28: 17. Transcripcion-Gustavo-Morales (1).docx - 28:14 [La gente no capta y no tiene p..]  (50:50)   (Super)
Códigos:	[modelo de salud futuro] 
No memos

La gente no capta y no tiene porqué captarlo porque es un tema muy sofisticado que las declaratorias de interés público son para un efecto especifico, para declarar invalida la patente eso es lo que hace, es decir las declaratorias de interés público no son una cosa retorica como decir esta carretera es de interés público, por supuesto que lo son, pero en medicamento por normas andinas y por tratados que ha suscrito Colombia las declaratorias de interés público, su único efecto es que la superintendencia de industria y comercio, debe entonces autorizar que terceros sin el visto bueno del titular de la patente, comercialicen o produzcan ese producto patentado, básicamente es una expropiación de la patente, lo que dice la norma que para expropiar una patente se necesita una declaratoria de interés público y la declaratoria de interés público sirve para eso, para nada más. Entonces con eso en mente la pregunta que hay que hacerse es si la existencia de patentes es la causa del problema que se quiere solucionar. Normalmente los gobiernos alegan que quieren bajar el precio, por eso hace una declaratoria de interés público y luego expropian la patente. Pero lo que termina pasando en áreas como la hepatitis C donde hay competencia, es que no es que se baje los precios, además tenemos precios contralados, lo que termina pasando es que se vuelve un favor a las empresas locales o a los importadores de genéricos, o de biosimilares no aprobados para que entren al mercado, pero el precio es el mismo por eso hay un techo y todos se pegan de ahí, nadie va a ser tan bobo de cobrar menos, nada ganas con cobrar 85 cuando puedes cobrar 10. entonces me ese sentido nosotros creemos que una declaratoria de interés público manda un mensaje muy negativo de que aquí no se respetan las patentes, y no vas a conseguir un resultado de bajar el precio y por eso es que nos parece que no cala.

P28: 17. Transcripcion-Gustavo-Morales (1).docx - 28:15 [En alguna época existía la pre..]  (54:54)   (Super)
Códigos:	[modelo de salud futuro] 
No memos

En alguna época existía la preocupación de que el control de precios iba a espantar la entrada de productos innovadores ya se ha demostrado que no pasa, si el laboratorio sabe a qué atenerse a la hora de precios traerá el producto lo importante es que sepa cuál es la regla, pero la existencia de la política no ha ahuyentado la innovación, que le está ahuyentando hoy en día, esto que hablábamos en una pregunta anterior de una incertidumbre si en este país se respeta o no la patente, y otra política muy importante de la que no hemos hablado es la puerta de entrada de productos farmacéuticos, que esta consignada en un artículo que es el 72 de la ley 1753 del 2015 que es el plan de desarrollo, nosotros lo demandamos la corte en parte nos dio la razón. Ese artículo lo que dice es que no puede entrar ningún medicamento a Colombia, ósea como entra un producto a Colombia con un registro sanitario que lo da la invita y ese registro lo da después de un análisis de seguridad y eficacia, el producto es seguro y hace lo que dice y no te mata, todas las invimas del mundo hacen eso. Pero en Colombia a raíz de esta política que esta consignada en el artículo 72  se dijo un momentico, antes de que haya permiso sanitario debe haber un acuerdo por el precio, si no hay acuerdo en determinación del precio el producto no va a entrar a Colombia, y uno entiende que el sistema de salud no quiera pagar un producto hasta que no se defina el precio eso está bien, pero si no tiene registro sanitario no lo puede pagar nadie, entonces nuestro argumento fue oiga si la persona quiere pagar de su bolsillo o tenga un seguro que lo compre porque no deja que lo adquiera, otra cosa es que el sistema decía no cubrirlo, pero usted no le prohíba a la persona de su bolsillo o por otros sistemas adquirir el producto. Entonces ese tema si puede espantar la llegada de innovación porque la gente no va a hacer el esfuerzo de conseguir un permiso sanitario cuando sabe que no se lo van a dar hasta que no hasta un acuerdo de precio. El ministro rivera ha tenido muchos aciertos, pero yo creo que él ha montado un discurso que básicamente, parece decir que la innovación es la culpable de todos los problemas de sostenibilidad del sistema, pero resulta que, por lo contrario, el próximo 22 de marzo vamos a tener un evento donde vienen varios profesores académicos del exterior a enseñarnos como la innovación bien implementada al sistema puede reducir los costos en salud de manera considerable. Si tú le das a un enfermo de hepatitis C esta droga milagrosa que la cura, eso cuantas entradas a emergencia te van a ahorrar, la cosa es que el costo de esta innovación sea razonable para que sea más el beneficio del costo. Pero esa política de fortalecimiento a entradas de medicamento es lo que está verdaderamente está espantando la entrada de innovación en Colombia. 

P28: 17. Transcripcion-Gustavo-Morales (1).docx - 28:16 [A mí me parece que la ley esta..]  (74:74)   (Super)
Códigos:	[modelo de salud futuro] 
No memos

A mí me parece que la ley estatutaria se equivocó en la definición en derecho a la salud, porque dijo que era el derecho acceder a la prestación de servicios de salud, si yo fuera sido Dios o legislador por un día diría que el derecho a la salud es el derecho a que todos podemos hacer lo necesario para tener buena salud, en lugar de gastarnos tanta plata en médicos deberíamos gastar la plata publica en gimnasios y en buena alimentación para los niños eso sería más barato y mejoraría la salud de los colombianos, ósea la asociación entre derecho y salud y prestación medica es lo que me parece problemática, nos lleva a parte curativa. Yo perdí 25 kilos los últimos 6 meses me siento mucho mejor de salud, mis años de salud han mejorado sustancialmente, sabe cuántas veces fui al médico por eso ninguna. Me parece que la ley estatutaria tiene una aproximación demasiado limitada y circunscrita de lo que constituye la salud humana porque para la ley estatutaria la salud humana es poder acceder a un médico y eso está bien, pero eximio al estado y a la sociedad  de toda la responsabilidad de lo demás, nutrición infantil, agua potable, buen ambiente nada de eso está relacionado con el derecho a la salud tal y como lo definición la ley estatutaria tu y yo que somos estudiados sabemos que esas son variables de la salud, pero la ley estatutaria los quito del mapa., entonces si yo tengo el mejor hospital del mundo a dos cuadras de la casa pero si la ciudad esta contaminada el estado podrá decir yo estoy cumpliendo. Pero el otro es este, eso es la crítica a la ley estatutaria pero par que todo no sea negativo que tiene una cosa muy buena tiene un procedimiento técnico y participativo para definir que está cubierto por el sistema. Eso está bien y eso ha funcionado, está en las primeras de cambio, pero va funcionando bien ya vamos por la tercera ronda, eso es positivo. Pero lo tercero es esto y lo más hereje de todos, yo creo que es un error definir la salud como un derecho fundamental entras en una condición muy complicada, sobre hasta dónde llega el derecho a la salud y haya donde no, yo pongo siempre en mis conferencias este ejemplo, nunca se ha dicho que el servicio de energía electica sea un derecho fundamental, sin embargo, este país paso de 25 años de tener una cobertura del 52% a tener una cobertura del 95% en el servicio de energía eléctrica. Y no fue necesario decir que eso era un derecho fundamental, simplemente se dijo que era un deber del estado prestar ese servicio, el estado lo ha logrado con dificultades, pero lo ha logrado. En cambio, si tú dices que la salud es un derecho fundamental, los jueces se pueden meter, y la intromisión de los jueces y la intromisión de los jueces ha sido de los grandes problemas en el sistema de salud. 

P28: 17. Transcripcion-Gustavo-Morales (1).docx - 28:18 [Pues es una lástima que se hay..]  (82:82)   (Super)
Códigos:	[modelo de salud futuro] 
No memos

Pues es una lástima que se haya retirado el gran líder que era el viceministro Fernando Ruiz se haya retirado porque eso quedo medio expósito allá en el ministerio, pero yo tengo fe y esperanza que el próximo gobierno lo rescate, tiene que ser el primer día de nuevo ministro de salud, que vamos a hacer con el MIAS así que yo creo que este funcionando antes de un año. Yo soy un optimista. 

P28: 17. Transcripcion-Gustavo-Morales (1).docx - 28:19 [Yo lo que creo es esto, el dis..]  (86:86)   (Super)
Códigos:	[modelo de salud futuro] 
No memos

Yo lo que creo es esto, el discurso oficial en los últimos 8 años ha sido que la incorporación en tecnología en salud ha sido el problema del sistema de salud, como lo vimos a lo largo de la charla, una incorporación ordenada en las tecnologías no sería el problema, sino que puede ser una buena parte de la solución. Y yo creo que toca poner el foco en corrupción en ineficiencias, hasta donde vamos a cubrir y hasta donde no pero las tecnologías en salud, no solo las tecnologías que tú y yo tenemos en la casa sino las tecnologías farmacéuticas, puede ser la solución a muchos problemas del sistema si la cogemos con inteligencia, con control de precio con compra centralizada, pagando solo lo que toca, lo que pasa es que ante la incapacidad administrativa del estado lo que se hizo fue que crear el sofá por la ventana, es decir que no entren para no lidiar con eso, que bueno sería que el estado decidiera que entren y vemos como las aprovechamos al máximo para hacer más eficiente el sistema. Esa sería mi reflexión final.

P28: 17. Transcripcion-Gustavo-Morales (1).docx - 28:20 [Yo creo que ahí no hay mucho q..]  (90:90)   (Super)
Códigos:	[modelo de salud futuro] 
No memos

Yo creo que ahí no hay mucho que hacer desde el punto de vista política pública, porque eso llego, eso va a pasar es como oponerse a Uber, pero la gente lo va a seguir usando porque se siente más cómoda y segura. Cuando las aseguradoras vean que les va mejor dándole a cada usuario un celular para que haga sus consultas por las pantallas, pues los hospitales van a tener que repensarse totalmente.

P29: 18. Transcripcion Carlos Hernandez.docx - 29:1 [Yo quisiera rescatar política ..]  (11:11)   (Super)
Códigos:	[modelo de salud futuro] 
No memos

Yo quisiera rescatar política perdidas y preservar algunas, yo rescataría que el sistema requiere de una asistencia pública en todo el país de base de asistencia publica no privada, es decir que el sistema que teníamos antes de un sistema de salud para los municipios en cada hospital en cada vereda en cada sitio, tuviéramos una responsabilidad pública, esa responsabilidad privada realmente no se ha dado, y se ha dado solamente en las grandes poblaciones por descremo, entonces no es lógico que cuando se hace descreme una le vaya bien en lo privado y a otra privadas les vaya mal y que al final la asistencia pública lo dejen lo no rentable y no haber intervenido de manera anticipada los riesgos, entonces considero que el sistema público debe tener un sistema nacional , publico para la baja complejidad igual en todos los municipios de responsabilidad pública, como el sistema de prestación y de aseguramiento de servicios básicos, y una segunda capa que son servicios más programados más previsibles basados en prima, en una prima como el aseguramiento hoy pero para eventos no básicos y para intervención de riesgos prevenibles, me explico cuando usted le dice a la población en Cali usted se encarga de cuidar  la población para bajar los riesgos y evitar que los pacientes tengan complicaciones un modelo en teoría lo dice, nadie lo hace en la práctica porque los competidores de ese mercado realmente no van a los barrios y si nadie con sus aseguradoras van a ir tocando puerta a puerta preguntando si es de Coomeva si es de Comfenalco, es un sistema totalmente ineficiente y si lo hace la asistencia pública responde por eso los riesgos básicos, lo hace un equipo que sea necesario sin diferenciación, en la segunda capa para riesgos quirúrgicos ya problemas existentes la gente tendría dos opciones una prima en que el estado paga como si fuera el subsidiado y tiene una red pública privada, sin diferencia y obviamente se competiría para que la gente vaya al mejor sitio posible con igualdad de condiciones y una prima para aquellos que tienen recursos económicos más alta, si quiere salirse de esa red vaya a  una atención más confortable, contribuyendo a esos planes gente con capacidad económica no como hoy en día clase media alta no paga nada para un riesgo diferencial y exige más que la gente de menor recursos. Entonces en un sistema así como lo estoy planteando yo preservaría lo que fue el sistema nacional de salud  con una red integrada entre salud ambiental y salud asistencial y riesgos colectivos preventivos lo integraría a lo que tuvimos antes, y una población que paga por una prima de riesgos quirúrgicos hospitalarios, que tendrán diferenciales públicos privados según la capacidad económica y tendrán mayor nivel de aporte a lo que hoy en día estamos haciendo alusión a un sistema diferencial por ingreso. 

P29: 18. Transcripcion Carlos Hernandez.docx - 29:4 [Si es la responsabilidad públi..]  (22:22)   (Super)
Códigos:	[modelo de salud futuro] 
No memos

Si es la responsabilidad pública para los problemas de los determinantes sociales como las otras entidades en lo básico, para asegurar temas estructurales hasta una capa de detección temprana de riesgos, y eso es público en todo el país. El privado muy poco ha hecho para intervenirlo y dejar lo privado con una competencia en prestación y en primas de aseguramiento para controlar los costos donde ellos van a competir intensamente pero todo lo efectivamente intervenido se está resolviendo con elementos de comportamiento y determinantes sociales.

P29: 18. Transcripcion Carlos Hernandez.docx - 29:5 [Lo que si es necesario en Colo..]  (30:30)   (Super)
Códigos:	[modelo de salud futuro] 
No memos

 Lo que si es necesario en Colombia es que lo público que fue el temor y sigue siendo el temor de mucha gente, de vuelva transaccional político y entonces en la transacionalidad político un sistema público donde la gente sin merito o con méritos pero devolviendo favores en términos burocráticos, pues sería una gran pérdida porque si se vuelven cargos y elementos electorales no llegaran los servicios a la población y hubo una época en el país sobre todo en nuestra región donde ser director de una unidad regional de salud era por mérito y estaba ligado a las universidades y había indicadores de comparación y sus estándares eran importantes a nivel de referencia internacional, pero hoy por hoy ocupar un cargo público no interesa de donde es, que está formado ni hay responsabilidad con resultados en muchos de los territorios. 

P29: 18. Transcripcion Carlos Hernandez.docx - 29:8 [la gente ha pensado que en la ..]  (42:42)   (Super)
Códigos:	[modelo de salud futuro] 
No memos

 la gente ha pensado que en la telemedicina hay experiencias buenas, y facilitar el acceso con unos profesionales, pero cuando uno habla de esto hay varios enfoques, uno es la rentabilidad otro es la venta de tecnología para uno y no para pocos, entonces yo lo superaría de la siguiente forma. Lo que necesitamos primero es generar comunidades virtuales, grupos de pacientes que entre ellos intercambien experiencias, uno de los aprendizajes en educación en salud no es la formulación del médico si no como aprende el paciente. Eso no es solamente de bajo costo si no que genera mejor adherencia y credibilidad y genera menor demanda de servicio, Colombia debería lograr que sus entidades tengan comunidades de pacientes, donde los aprendizajes y los estos sean compartidos rápidamente para generar hábitos y comportamientos, y adherencia en tratamientos protocolizados para aquellos eventos que son altamente estandarizados. Segundo es un tema de apoyo a comunidades distantes facilitando la comunicación y seguimiento del paciente donde los aplicativos como tú lo dices bien sean por una imagen o por un sensor que está reportando síntomas y objetivando la atención sin generar riesgo se pueda orientar la conductas. Tercero lo que necesitamos es la formación de equipos de salud en el conocimiento para facilitar y asegurar el conocimiento de las tecnologías aplicadas al sector salud, no es el aparto de glicemia en si el que te va a determinar, es un químico farmacéutico el que debería estar ajustándole las dosis a una cohorte de pacientes que están descontrolados, ósea de medicalizar el acto médico no entregándolo a un equipo, estenme expertos formados para la tecnología para que con la tecnología y los pacientes y sus familia generen casos de atención mucho más activos que un acceso al endocrinólogo el manejo la preparación de alimentos en la casa, el manejo de las dosis de la insulina para un paciente diabético no es un asunto del endocrinólogo, es un asunto de un equipo de salud que está más cercano utilizando una tecnología pero la tecnología es el proceso de atención, el grupo de profesionales y el equipo generando compromisos y adherentes, muchas veces tomamos de referentes internacionales google health, google health va a sacar todos los aparatos que te va a haber un diagnóstico de todas las cosas, y eso se va a vender como se vende hoy en día Netflix como se vende DIRECTV para monitorearte y decirte como estas, eso es una transacción comercial y tu va a tener un acceso y te van a decir eso. Pero ese es un elemento económico importante al que van a poder acceder pocos. Lo que nosotros es demostrarle al país, es como genero conciencia de comportamientos y hábitos, como traslado un concepto sub especializado a un concepto de un profesional en unas áreas que acompañan y garantizan el éxito del tratamiento y yo le traslado a algunos que tiene la capacidad de inquirir esos sensores y esos monitores para todo los órganos que hoy en día se está llevando, ese es el proceso que va a ser muy importante en esta escala. Peros seguramente llegara rápidamente al tema de la genética, al llevar a participar nosotros, cuál de esas cohortes del paciente su familia independiente de sus hábitos va a tener estos riesgos de enfermedad y con eso se pueda generar entonces una ambientación de planificación general basad en epigenetic para decir no queremos tener más riesgos de estas enfermedades raras, usted debe trabajar un programa a planificación familiar, ósea que hoy por hoy en todo lo que hemos mencionado no hay clínicas, no hay hospitales, lo que hay es comunidades de pacientes que cuando se salen de esos estándares van a estar acá y vamos a buscar con tecnología evitar que los pacientes que tienen riesgos genéticos para reproducir enfermedades no generen ese riesgo y se pueda dividir esa cohorte, pacientes y enfermo. La tecnología ayuda, pero ayuda mucho más la generación de conciencia y de comportamiento con equipo s de salud trabajando con las comunidades. Eso es lo que quisiera yo primero, poco a poco. 

P29: 18. Transcripcion Carlos Hernandez.docx - 29:9 [Se requiere primero una políti..]  (46:46)   (Super)
Códigos:	[modelo de salud futuro] 
No memos

Se requiere primero una política de país, de decir que toda la historia clínica tiene código H7 para que todo viaje y para que haya un depositario único y que la historia clínica la cargue el paciente en su dispositivo y eso es un tema que no debe ser voluntario si no debe ser una política de cumplimiento para todo y eso no se ha logrado hacer y eso ayudaría mucho para que no tengamos que repetir exámenes y tardar la atención para volver a realizar cosas ya hechas, dos hace falta una obligación de tener una plataforma para garantizar el acceso a una citas del punto de vista un hobb de citas y que cada uno no tenga diferentes mecanismo, eso se puede hacer también por política en regiones como una central de ambulancias, todo eso se puede hacer. Lo que no le puedo trasladar a la tecnología es lo que realmente necesita el paciente y es ahí donde yo tengo diferencia, pero google earth esta encontrado un mecanismo de presión para que se utilice y entre rápidamente cuando tenga su desarrollos pero lo básico no se ha resuelto, incluyéndonos a nosotros, muchas veces el paciente está pidiendo una cita para algo que no puede ser una enfermedad, piden citas para saber si el dolor que está sintiendo era el esperado o no o si se puede tomar otro medicamento y eso no requiere una cita médica si no un cercanía con el medico que ya lo vio y que lo conoce, no tiene que volverlo a examinar. Muchas veces llaman para saber dónde le van a retirar el yeso y están pidiendo citas porque no le dijeron donde le tienen que retirar el yeso, muchas veces llaman para preguntar si pueden tomarse una copa de vino o no para algo y eso no es una cita médica. Con esto quiero decir que hemos vuelto y queremos vía transaccional a acercar el acto médico a alguien que lo que requiere es una orientación, a alguien que lo requiere es un acceso a un portal donde debería estar la información de lo que es y lo que debería de ser, a que no le ha llegado una capacitación básica de cómo manejar su enfermedad. Entonces cuando nosotros estamos diciendo que deberían de tener facilidad de hacer agendamiento web, eso está muy bien cuando está claro, cuando toda la cadena antes del acto médico se está cumpliendo, es decir yo tengo un sistema de salud donde la población se auto diagnosticara se autocontrol, mide su peso, su talla, sabe cómo está su presión arterial, tiene un dispositivo donde puede ir a un supermercado y tomarse un analgésico y saber cómo está su glicemia o la entidad le asegura al diabético su seguimiento y lo único que necesita saber es si debe o no ir a urgencias, pero nuestro sistema lo que ha hecho es una poca presencia en generar capacidades al seguimiento, no necesariamente con tecnología, tengo un sistema donde la puerta de entrada es por urgencias, el 70% de los pacientes demandan los servicios por urgencias debía ser el 80% de consulta externa, y le estoy entregando a la consulta externa cosas que son de enfermería de nutrición de psicología, cuando la gente debería tener ese conocimiento básico claro, y un tema de esos seguramente va a requerir muchas menos transacciones web médicas y va a tener comunidades externas interactuando entre ellas dividido en la presión de prestación intramoral y especializada cuando hacemos fallado en toda la cadena, si nosotros actuamos en esta cadena seguramente esa medicina especializada y la tecnología, claro hoy en día se hace cirugía robótica de un país a otro y se hace la cirugía, pero cuantos deberían estar en cirugía, cuantos recién nacidos deberían estar en cuidados intensivos, si nosotros entendemos que la tecnología es necesaria deberíamos hacer esfuerzo para que no tengamos grandes consumidores de tecnología cuando nosotros debemos intervenir en los riesgos antes, y por eso en la lógica que menciono es que si estamos mal en plataformas virtuales, estamos mal repitiendo procesos de atención con alto costo, pero también no nos vamos a enloquecer haciendo esto cuando hemos dejado de hacer muchas cosas y los recursos deberían de estar previamente. Una forma de decírtelo es a mí no me sirve que yo recato muy bien a la paciente embarazada de alto riesgo y la saco adelante con un año de 600 Gramos, y aquí eso es motivo de orgullo en américa latina tener ese tipo de entidades la pregunta es y usted que hizo como clínica para que esa cantidad de niños que llegan allá, usted como entidad tan importante no dijo de que ciudad eran, donde no le pidieron un control prenatal, porque no le hicieron un programa de planificación familiar, porque ese paciente llego allá. No el orgullo es que acá rescato la gente súper bien a un costo de individuo alto, no importa que salga con limitación de su capacidad intelectual. Cuando uno es responsable como organización de entregar salud uno deberían de optimizar la tecnología pero también para informar donde están las fallas de nosotros para que esto no vuelva a suceder. Eso ha sido un tema histórico que en nosotros como país deberíamos de tener apuestas tecnológicas pero con la exigencia que de esas personas que lo están usando intervengan orienten y ayuden a buscar las causas estructurales para que ese tipo de situaciones no se vuelvan a suceder. 

P29: 18. Transcripcion Carlos Hernandez.docx - 29:10 [es un sistema que prometió ten..]  (49:49)   (Super)
Códigos:	[modelo de salud actual] [modelo de salud futuro] 
No memos

es un sistema que prometió tener múltiples planes, competidores en múltiples beneficios y lo que yo considero es que no se ha logrado eso, tenemos muchos actores muchas EPS pero que en la realidad la EPS más grande de país en este momentico se llama supersalud, en este momento debería de tener el seguro social, caprecom, cajanal y fondos especiales hoy tenemos supersalud, salud total y Sura. Pasamos de un oligopolio a otro oligopolio, porque hoy en día están intervenidas, la gran mayoría, caprecom, cafesalud Coomeva, SOS, están intervenidas, ósea el verdadero asegurador ahí se llama supersalud, y otras pocas tratando de tener los equilibrios pero esas otras no van ni quieren recibir más traslado de población , el sistema arranco en una competencia basada en competidores y hoy en día ningún asegurador quiere recibir lo que están dejando porque seguramente no son viables por su estrategia de localización y en la medida que cafesalud cierra y entrega municipios, Coomeva entrega municipios SOS entrega municipios, esos resultados de esas otras entidades se empiezan a deteriorar entonces estamos resolviendo al final de la corriente los temas estructurales, y en este sistema vienen padeciendo el sistema de salud, los prestadores las EPS que estaban equilibradas, y no hemos ajustado ni el modelo de intervención de riesgos y determinantes de la salud, no estoy haciendo modelos de intervención público más costos efectivo, y tampoco estoy exigiendo resultados en salud, solamente lo tenemos para un evento llamado enfermedad renal que es la única enfermedad que se paga por post y no hay más pagos por resultados, deberíamos de tener un sistema estar corrigiendo esto y pago por resultado de salud. 


P29: 18. Transcripcion Carlos Hernandez.docx - 29:14 [CH: Las políticas de salud deb..]  (65:65)   (Super)
Códigos:	[modelo de salud actual] [modelo de salud futuro] 
No memos

CH: Las políticas de salud deben de tener cimientos, es como la ley 10 y la ley 60 previos a los orígenes de la ley 100, si usted le dice al municipio que es responsable y no le da la plata, de qué sirve la responsabilidad si no va a haber la plata. Entonces está pasando con el MIAS es que el modelo lo exige, pero no ha cambiado la obligación de que las redes surgen de una ley que aún sigue existente, las SSS son de orden municipal y departamental, ósea que no tienen ninguna obligación de integralidad, cada S puede montar los servicios que quieran. Entonces esa S puede tomar la decisión de no tomar el parto porque no es rentable, la otra considera que es mejor la endoscopia, la otra S toma la decisión y la ley le obliga a usted a articularse con lo que tiene y con que es lo que tiene, tiene lo que es rentable, lo que es rentable no lo ofrece. Como puede haber modelos integrales de atención si hay procesos de atención que no son lucrativos y la gente dejo de hacerlos, ese traslado de lo no lucrativo lo harán algunos a costa de quebrarse o tener mala atención, entonces cual modelo en redes de atención cuando hay competencia departamentales, municipales y hay libertad de esclarecer servicios según su rentabilidad, por eso en la propuesta en el principio de la conversación dije esto no puede la plataforma es que la obligación de la red principal de atención es publica, y entonces el secretario de salud no va a convencer a un director de S si quiere o no hacerlo si no que él es la autoridad municipal y que va a decir partos acá no en todos los sitios con criterios administrativos eficientes, de alta calidad, partos de alto riesgo acá, acá esto lo otro. Pero hoy en día son las S  las hay municipales, departamentales, entonces no, el modelo integral de atención es esto lo que yo le ofrezco y señor aseguramiento privado sus pacientes asegurados por el nivel 1 le cuesta en promedio 80000 y reciben los mismos servicios todos, ya el resto de servicios que le va a costar el nivel 2 lo puede hacer por las otras redes, pero lo básico cuesta eso, no es que usted me monte aquí una IPS para decir que hace cosas, no yo asumí la responsabilidad total y tengo la plata. Y él va hacer esfuerzos para que esto se mantenga bueno, no va a montar IPS para tratar de hacer en escala que no logra ni va a ir a los barrios porque no tiene como hacerlo entonces no tiene realmente un modelo de intervención de riesgos colectivos eficientes. Por eso el debería dedicarse al riesgo secundario, ya tiene la enfermedad acá se trata de detectar temprano y yo lo voy a controlar a cuidar, yo recibo una prima por eso, pero hoy en día recibe toda la prima por todo y entonces se privilegia a lo final porque es lo que más exige lo más urgente y poco hago en esta capa, y no lo hago con lo público pero lo público lo hace mal porque cada vez una entidad hace lo que quiere, cada S uno pregunta dónde está la ley de referencia y contra referencia publica donde dice parto de segundo nivel, ortopedia de segundo nivel, no yo no hago ortopedia, partos. No hay un sistema de resultados con responsabilidades para decir porque uno va a una unidad de cuidados intensivos en Cali y encuentra niños con Lúes, que es sífilis congénita y son pacientes de medicina prepagada, pacientes de régimen contributivo que quiere decir eso, que no es un tema que la gente le esté costando montar sistemas de captación, seguimiento de riesgo y cada quinen hace como lo quiere, pero no están habiendo resultados ese modelo tiene que cambiar, no es posible y no es sostenible un modelo MIAS cuando las reglas de juego estructurales básicas no están establecidas. 

P29: 18. Transcripcion Carlos Hernandez.docx - 29:15 [Yo haría el sistema público na..]  (69:69)   (Super)
Códigos:	[modelo de salud futuro] 
No memos

Yo haría el sistema público nacional para la atención básica en captación de riesgos y en atención   de servicios básicos, de responsabilidad pública sin aseguramiento privado. Y esa plata suficiente llegaría a los entes territoriales con una clara obligación que la dirección nacional y una ley de fuerza constitucional obligaría que las necesidades de salud de causas estructurales sean aprobadas en comités de planeación local y departamental donde los factores determinantes de la enfermedad sean resueltos. Me explico, yo le doy vueltas al tema que hoy en día lo vemos como prestación y los determinantes por otro lado, si yo tengo una comunidad de nutrición con problemas odontológicos, respiratorios, no puedo darle plata porque eso cuesta esa plata la atención de ellos, no yo les tengo que decir al ente territorial o local que hay que mejorar las condiciones de empleo, hay que darles subsidios por producción de alimentos actividades básicas para generar la intervención de problemas nutricional y no darle plata para la nutrición como un tema de complemente de atención a asistencia médica como una consulta de nutrición o como una hospitalización como una enfermedad diarreica, el plan de municipio yumbo o Jamundí, es un plan para producción de alimentos, un subsidio alimentario una práctica para la generación de empleo de ingresos y condiciones de vivienda para gente que se enferma de tuberculosis o desnutrición, no es una plata para que el hospital de la unión reciba la plata para que atienda desnutrido con tuberculosis, lo que necesita esta país es que si la necesidad y los factores determinantes de las enfermedades tienen la obligación los entes territoriales de presentar el plan de intervención estructural y apropiar los recursos para eso , y para la asistencia en una forma gradual se le darán los recursos de prestación, en aquellas poblaciones que son distantes de los centros esa entidad será aseguradora y prestadora, como ha pasado con antiguos territorios nacionales allá no hay oficinas de EPS, allá lo único que existe es la institución de salud, pero debe haber una institución social que resuelve los problemas sociales económicos de esa población, entonces eso es lo que estoy considerando. Una vez que se presentan estos elementos esta lógica de ente territorial, ya la enfermedad, apendicetomía, vesícula, infarto, paciente fumador, en ese nivel habrán unas prestaciones que sumaran un asegurador público para una segunda capa y aseguradores privados que van a competir para garantizar que eso que no resuelve lo básico, van a tener entidades idóneas, de calidad y controladas a un costo menor que lo que es hoy y que a la final están yendo a un nivel de alta complejidad generando una rentabilidad a muy pocas clínicas, porque lo rentable es lo complejo lo intervenido, entonces un sistema cuya pirámide de costo debería de ser así, esta invertida, usted va encontrar que hoy en día las clínicas la que son viables y rentables están concentrando a pacientes muy complejos a un alto nivel de rentabilidad a un costo enorme y los otros soportan costos altos y bajos márgenes están muy mal económicamente y la gente demanda muy poco oportunamente porque el asegurador no tiene la capacidad económica porque en el modelo no ha obligado a que la puerta de entrada es muy fácil y hay compromisos territoriales de mayor alcance, el tema ambiental el tema de alimentación, temas que son estructurales hoy en día se resuelven con plata para cuidados intensivos para drogadictos, y los temas estructurales ya están resueltos. 

P29: 18. Transcripcion Carlos Hernandez.docx - 29:19 [Por ejemplo ese tema de la cul..]  (93:93)   (Super)
Códigos:	[modelo de salud futuro] 
No memos

Por ejemplo ese tema de la cultura ciudadana de que la gente consuma productos saludables marcados con rojo, amarillo y verde. Como una política de esas sin tener que ver el tema asistencial está diciendo vea consuma dieta saludable, políticas que como gobernante se toma. 


P30: 19. Transcripcion Felipe Garcia.docx - 30:1 [Yo tengo pues una opinión aunq..]  (7:7)   (Super)
Códigos:	[modelo de salud actual] [modelo de salud futuro] 
No memos

Yo tengo pues una opinión aunque no todos la comparten, el problema en Colombia se ha centrado en dos grandes talones de Aquiles, lo que llamamos el componente humano de lo que llamamos los prestadores en salud, y ese componente humano pasa por muchos casos una deficiente formación clínica, que hace inclusive que el mismo sistema empiece a fallar desde la base de poder valorar diagnosticar al usuario, si uno mira por ejemplo a partir de la ley 100 toda la parte de la EPS las IPS, se miraron siempre con una visión muy empresarial muy administrativa, y no se dieron cuenta que el sistema es tan complejo que no se pueden prevenir los costos a priori, pongo un ejemplo una persona que ha cotizado 3 meses o 1 año, y se va a cuidados intensivos 15 días cuando por lo menos son 3 millones de pesos diarios ya cuanto gasto, eso le crea una debilidad al sistema pero además de eso los que trabajan en esas unidades de cuidados intensivos no tienen como una formación suficientemente buena por lo menos en las clínicas de combate, no estoy hablando de los hospitales ni las clínicas elite,  para que ese paciente tenga un buen tratamiento y por lo menos se salve. Entonces ese es un problema que viene mucho de la formación del médico de la formación en salud es la calidad en formación que no voy a entrar a criticar, pero en general el sistema de educación en personal en salud, principalmente los médicos esta deficiente, salvo alguno ejemplos algunas universidades, algunas facultades, algunos programas de medicina, tienen como esa visión pero en general es una formación muy tecnológica, muy de tomar un cuestionario y decir ta ta ta, entones usted tiene esto, y están considerando que esa persona es un formulario, no es una maquina ella siente. Lo otro es el componente es el tipo financiero, es claro que en el mundo los sistemas de seguridad en salud no pueden autofinanciarse, primero porque hay diferencias en edad y las afecciones van cambiando, no es lo mismo de un niño o una persona de 18 años a una persona de 40 años que se llama la tercera edad y lo que se ha visto es que los costos de atención y los costos de mantenimiento y aun los costos de programas de prevención van aumentando en medida que aumenta la edad de la persona, y desafortunadamente muchos sistemas y muchas sociedades se están yendo a la pirámide invertida, se engruesan a la mitad pero también tienen un gran engrosamiento hacia la parte final, Colombia está en eso, esta caracterizada como una pirámide que tiende a hacer un país viejo, un país en el que la gran población está por encima de los 40, 50 años y eso también le trae costos muy fuertes a un sistema público de salud. entonces esos dos componentes provocan lo que para mí es una crisis, y la legislación actual no la va a resolver por dos razones, primero la formación tiene mucho que ver con un creciente resurgimiento en facultades y programas de medicina, con una formación no completa para casi satisfacer un mercado laboral médicos generales que vayan a las EPS, y lo otro los costos de atención básicamente día a día que se tecnifica la medicina los costos de equipos y los costos de laboratorios y exámenes vayan más grandes, porque la medicina obvio ha avanzado en términos de manejo, en términos de hacer más preciso el diagnostico que se hace, y entra los costos de toda esa gran tecnología que vamos a ver más adelante es muy grande. Desafortunadamente tiene que acudir a un sector llamado el privado que es el que va a invertir pero quiere recuperar también. 


P30: 19. Transcripcion Felipe Garcia.docx - 30:3 [Mire hay unos estudios, que co..]  (15:15)   (Super)
Códigos:	[modelo de salud futuro] 
No memos

Mire hay unos estudios, que comienzan a decir que el sistema no es sostenible en 10 años, la crisis que viene va a ser más profunda de una atención pública, de un sistema público, entones yo pienso que tarde o temprano tenemos que ir a un sistema mixto que es como la tendencia mundial, estaba para algo peor la inversión privada también va a hacer algo, esos sistemas publico privado en Colombia no han tenido mucho éxito. 


P30: 19. Transcripcion Felipe Garcia.docx - 30:4 [Yo pienso que un sistema de sa..]  (23:23)   (Super)
Códigos:	[modelo de salud futuro] 
No memos

Yo pienso que un sistema de salud tiene que tener 3 grandes cualidades, primero cobertura, y la cobertura con calidad, y tercero que pueda moverse, que pueda tener una capacidad de movimiento lo suficientemente buena para que los usuarios escojan su atención. Entonces en ese sentido el sistema es costoso si lo va a pagar el estado, el estado guste o no le guste no puede y mucho más el estado colombiano no puede costear 100% el sistema de salud, entonces para mi debería hacerse un sistema estratificado que en parte lo está en este momento, con la cuota moderadora con todo ese tipo de copagos, está siendo estratificado entonces yo estoy de acuerdo el sisben ha sido excelente hasta que llegaron los políticos y lo amasaron y le hicieron todo lo que quisieron, no vamos a hablar de eso, pero ese era un modelo interesante, porque estaba sobre los estratos sociales de menor ingreso y de menor capacidad, y un aspecto del sisben es que cogió zonas rurales porque desafortunadamente el fuerte del servicio está en las ciudades, la calidad del servicio en atención en salud está en ciudades, en las zonas rurales es casi cero, entonces para mí el sistema tiene que estar en dos planos, en uno de una faja poblacional que cada día va a ser menor y que de alguna manera no puede pagar por un servicio lo suficientemente bueno y de calidad, y ahí el estado si tiene que meterle toda la inversión, hay otra faja que es la del medio que es la que estaría en la mitad, ella está ofreciendo algunos servicios públicos pero también tiene que estar en un régimen semiprivado, y yo pienso que se debe tener un servicio mixto, después de los niveles 4 5 y 6 se debe hacer un servicio mi

P30: 19. Transcripcion Felipe Garcia.docx - 30:5 [Pero en una coinversión es que..]  (27:27)   (Super)
Códigos:	[modelo de salud futuro] 
No memos

Pero en una coinversión es que el sistema por donde lo mire, yo no soy economista, por donde lo mire el sistema se tensa en esa fibra y el gran problema es que los que están abajo se están consumiendo, ellos no pueden pagar, por eso digo gratuidad en los estratos más bajos, porque ahí es donde los problemas de salud pública son mucho más preocupantes, no hay estructura sanitaria, una estructura de vivienda insuficiente, asignación, entonces yo diría que ahí es donde más riesgo hay para enfermedades para una cantidad de efectos en la salud, entonces en estas yo diría 1, 0 y 2, es donde está la debilidad que el sistema no ha podido ir allá, les obligan a pagar unas cuotas entonces yo si diría eso debería ser gratis, es el deber del estado ya más adelante hay que hablar de cómo hacer la cofinanciación, teniendo como base que el estado no puede pagar y no tiene plata para pagar un servicio netamente público. 

P30: 19. Transcripcion Felipe Garcia.docx - 30:17 [Ahí me metió en un lio a mi po..]  (77:77)   (Super)
Códigos:	[modelo de salud futuro] 
No memos

Ahí me metió en un lio a mi porque yo estoy con las EPS no como están concebidas, como unidades operativas de salud que es otra cosa, una unidad operativa de salud es una unidad administrativa jurídica, con capacidad clínica médica de soporte, con capacidad suficiente tecnología para detectar diagnosticar y con una capacidad para mantener la salud de sus afiliados, eso para mí es una unidad, llámelo EPS, IPS son unidades entonces el sistema tiene que tener unidades, y esa unidades no tienen que estar aisladas, hoy en día las redes son las que hacen las fortalezas. Mire porque están quebradas las EPS, están aisladas porque yo tengo es que ver como cuido mi capital y el retorno de capital y la sostenibilidad financiera, una unidad de estas no necesita preocuparse por sostenibilidad financiera porque esta poyada por otras, ese sería un sistema para mí de unidad operativa que no haya una competencia si no una complementariedad, que esa unidad sea más experto en la parte cardiológica que la parte gástrica, y esas dos se complementan y se comparten fortalezas se comparten diagnósticos, especialistas. ese sería un sistema que administrativa mente sería más eficiente porque que haría, el recurso profesional lo estaría manejando de manera global integral y segundo el manejo del paciente se vuelve más abierto, por especialistas y eso es una tendencia de los grupos de especialistas entones que hacen las EPS, vaya consulte con especialista de pulmones en tal grupo, ya casi que tienen contrato con esos especialistas ese es el segundo nivel de atención ya esos médicos generales le dicen usted tiene un problema de pulmones vamos a remitirlo al especialista x o Y y el pulmologo no está en la EPS él se hace outsourcing y contrata especialistas. entonces allí le quitaría ese problema a las EPS porque serian unidades operativas especializadas, entonces el paciente está en el sistema y circula dentro del sistema, entonces eso significa para mi administrativamente más eficiente, entonces usted lo maneja como un sistema integrado se complementa, la medicina en estos momentos ningún momento se las sabe todas con todo el respeto, porque ha avanzado tanto la tecnología que el concepto médico para resolver un problema necesita un especialista, el medico egresado de la universidad es un médico que básicamente mira la epidermis y a duras penas puede ir a la dermis, y hace un aplacamiento de los órganos internos no puede ir mas allá, le falta conocimiento. Entonces que tiene que hacer, las famosas especializaciones, eso hace que esa persona sepa mucho de cardo, de pulmones, para poder manejar las enfermedades complejas que hoy en día vienen siendo eso las enfermedades, porque nuestro alimento tiene una cantidad de éxito y por eso es la fuerza del cánce

P30: 19. Transcripcion Felipe Garcia.docx - 30:21 [Porque es costoso. Más que la ..]  (101:101)   (Super)
Códigos:	[modelo de salud futuro] 
No memos

Porque es costoso. Más que la atención primaria en salud, porque implica tomar desde bien temprano a esa persona y hacerle una serie de seguimientos, se llama atención primaria es eso para prevenirle o decirle en que momento va a tener usted problemas, es que la atención primaria no es hasta los 6, 7 años para mí la atención primaria es la primera fase para determinar si usted va a estar enfermo o no. Entonces el concepto de atención primaria se vuelven en el concepto de atención básica, yo cuando tenía 5 años, tenía un estado metabólico diferente al que tengo ahora, me lo atendieron y puede ser que mucho que no me atendieron puede ser lo que me está pasando ahora, los efectos a largo plazo de salud se dan desde que usted está en el vientre materno, y eso se llama epigenetica su genoma lo pueden remarcar para que se vea enfermo y después sienta las afecciones aun ya en el útero en el vientre materno, entonces que es atención primaria. El derecho a la salud debe existir como norma política constitucional tiene que ser, todo sistema mundial tiene que conocer el derecho integral a la salud como lo maneja es otra cosa. 

P30: 19. Transcripcion Felipe Garcia.docx - 30:23 [FG: Entonces ese sistema tiene..]  (112:112)   (Super)
Códigos:	[modelo de salud futuro] [TECNOLOGIA] 
No memos

FG: Entonces ese sistema tiene el gran defecto de digamos esa administración del sistema esta corrupta, en ciertas partes es un sistema tan factual que esta al dominio de lo comercial es un sistema que no va a funcionar. Entonces yo pienso que el sistema integrado de salud no sé si pueda funcionar como tal a mí me da cosita hablar de ese tema integral en salud, porque si fuera integrado tendría que colocar las medicinas que llamamos no tradicionales vs las occidentales alopáticas que llamamos tradicionales. Yo no soy como tan simplista pero creo que las tendencias se tienen que ver, relacionada mente ambas subsisten ambas sociedades están, toda sociedad está enferma toda comunidad humana va a estar enferma, si uno mira las estadísticas de cáncer a los 60 va a tener cáncer de mama, pero que se debía que la tecnología puede detectar cáncer de mama hasta edad muy bajita, sale la famosa mamografía, entonces ya el cáncer de mama se vuelve muy frecuente, será que estaba así, será que siempre ha habido así grandote y por la tecnología diagnostica lo subimos, esa es la pregunta. O será que precisamente está yendo hacia allá, que los ambientes lo está tocando tanto. Tal vez en los canceres gástricos hay una explicación, por la dieta y ahí sí puedo creer que el cáncer gástrico haya aumentado, bien sea por la parte de enlatados. El de próstata por ejemplo, yo el otro día estaba hablando con un estudiante de doctorado que es urólogo, es que el cáncer de próstata tiene que aumentar, como así que factores hay para que haya cáncer de próstata aparte de la edad. 

P30: 19. Transcripcion Felipe Garcia.docx - 30:28 [Por eso a mí no me han podido ..]  (155:155)   (Super)
Códigos:	[modelo de salud futuro] 
No memos

Por eso a mí no me han podido definir que es atención primaria en salud, y tengo muchos médicos que son médicos de medicina familiar es aceptarle al niño si va a tener neumonía, si va a tener problemas pulmonares, si va a tener asma. Inclusive la misma organización de la salud no ha podido definir que es atención primaria en salud, esa atención a la primera infancia perfecto, ahí no lo dicen la atención prenatal pero no es primaria, es una fase, cada fase de la vida del hombre es primaria cuando le dicen a la mujer la menopausia eso cambio completamente la salud de la mujer le quito una función que tenía antes, ahora le reorganiza todas las hormonas y todo eso y hay personas que se suicidan por depresión menopaúsica, entonces que es atención primaria, a que estamos atendiendo allí lo que pasa es que se ha medido la protección más materno infantil hasta los 7 años, eso es lo que se ha definido como atención primaria, de ahí para adelante no sé qué es atención primaria. 

P32: 21.Transcripcion Oscar Gutierrez.docx - 32:11 [OG: Suficiente no pero debe se..]  (40:40)   (Super)
Códigos:	[modelo de salud futuro] 
No memos

OG: Suficiente no pero debe ser el primordial, el que marca el liderazgo, no es un sistema preventivo, no curativo que no responda a la demanda del paciente enfermo sin o que responda a la posibilidad que el paciente se enferme, el único país en américa que probablemente tiene algo parecido es cuba, porque es pobre por el mismo sistema de pobreza que tiene y cuba se dedicó desde principios de la revolución a dos cosas, a educar a la gente y a darle salud, pero no tiene recursos para dar salud entonces ellos detectaron desde muy temprano que la mejor estrategia era coger al paciente cuando apenas se estaba enfermando y crearon los médicos primarios, las facultades de medicinas, yo no me dejaría tratar de un médico cubano para una enfermedad pero como sistema es el sistema que tiene la mejor atención materna, en atención infantil y en tratamientos de las enfermedades crónicas, usted sabe por ejemplo que los cubanos fuman tabaco desde la mañana y hasta que están niños sin embargo las complicaciones respiratorias que tienen las controla mejor que por ejemplo en Colombia que por ejemplo el fumador o exfumador llega al médico cuando ya está arrastrándose al cajón, en cambio allá el medico de cuadra va periódicamente y esperan que no llegue a un punto de no retorno. En cuba hay 500 personas por cada médico en Colombia es 1 medico por cada 10000, pero en Colombia no va a ser posible porque los médicos ahora la medicina dejo de ser un arte una vocación, no es que el médico no pueda gastar plata pero la plata no puede ser la primera preocupación del médico, como pasa ahora y lo que es peor la actitud tanto de la gente como de los médicos ha cambiado, ahora es perfectamente aceptable deseable que la única función del médico sea tener un mercedes Benz, con todas las arandelas, ya el éxito de los médicos jóvenes se mide por el carro donde andan y me llama mucho la atención, las conversaciones entre los médicos es de donde pagan mejor y que tienen que hacer por levantar plata, entraron en un mercado y el mercado de la salud esta para eso. Cuando yo estudie acá se medía la vocación y se admiraba a los médicos y si el medico tenia platica pues era muy bien ganada, ahora nadie le cuestiona al médico como se gana esa plata operaciones que no necesitan. 

______________________________________________________________________

Código: Protección financiera : financiación del SGSSS {52-0}

P10: 3. Transcripcion-Alexandra_Matallana_ok.docx - 10:9 [La proyectaron dejando todos l..]  (78:78)   (Super)
Códigos:	[Protección financiera : financiación del SGSSS] 
No memos

La proyectaron dejando todos los supuestos iguales, nada va a cambiar. 

P10: 3. Transcripcion-Alexandra_Matallana_ok.docx - 10:10 [Pues que pienso, que la UPC cl..]  (86:86)   (Super)
Códigos:	[Protección financiera : financiación del SGSSS] 
No memos

Pues que pienso, que la UPC claramente no está alcanzando, no es suficiente. 

P10: 3. Transcripcion-Alexandra_Matallana_ok.docx - 10:11 [Grave que tiene el sistema per..]  (89:89)   (Super)
Códigos:	[Protección financiera : financiación del SGSSS] 
No memos

Grave que tiene el sistema pero además también hay trabajos como en un círculo vicioso porque un sistema desfinanciado tampoco puede tener tarifas que sean consecuentes, pero pienso que es insuficiente. 

P10: 3. Transcripcion-Alexandra_Matallana_ok.docx - 10:12 [Como le decía anteriormente a ..]  (105:105)   (Super)
Códigos:	[Protección financiera : financiación del SGSSS] 
No memos

 Como le decía anteriormente a mí me preocupa eso, que el sistema esta desfinanciado y me preocupa que se siga desfinanciando por eso, por la política económica del país. 

P10: 3. Transcripcion-Alexandra_Matallana_ok.docx - 10:13 [si, es de pensar que las EPS e..]  (109:109)   (Super)
Códigos:	[Protección financiera : financiación del SGSSS] 
No memos

si, es de pensar que las EPS estén trasladando, siempre le han trasladado a las IPS, con esa forma de contratación pues lo hace, el riesgo de salud ¿no? 

P10: 3. Transcripcion-Alexandra_Matallana_ok.docx - 10:18 [i claro, claramente es lo que ..]  (177:177)   (Super)
Códigos:	[Protección financiera : financiación del SGSSS] 
No memos

i claro, claramente es lo que yo te digo dentro del flujo financiero del sistema claramente hay unos problemas muy grandes y además digamos que el core de la aseguradoras no es mejorarle la salud a sus usuarios porque ellas son entes financieros, y su core no es cuantos pacientes tengo sanos, es cuánta plata ganar si soy eficiente en mi gestión … tampoco digo que vayan a perder, pero miremos como esta ese flujo y para donde se está yendo la plata del sector, la plata del sector es para atender a la salud y a la enfermedad del colombiano, pero para donde se está yendo no lo sé, pero se está escapando. 

P11: 4. Transcripcion Armando Gonzales_ok.docx - 11:4 [La unidad de pago por capitaci..]  (13:13)   (Super)
Códigos:	[Protección financiera : financiación del SGSSS] 
No memos

La unidad de pago por capitación que básicamente puede ser que la del  régimen contributivo digamos que es aceptable, pero el problema no es el valor de pago por capitación, lo que afecta realmente es la financiación del sistema, por ejemplo el régimen subsidiado que todo es financiado por el gobierno, es una plata demasiado importante si tú tienes que cubrir 21 o 24 millones de personas con régimen subsidiado todo por el gobierno eso ya da un desnivel económico muy grande, que el gobierno tiene que solventar pero que no está en capacidad de solventarlo en su totalidad;  lo cual crea un desequilibrio económico muy grande que impide que las cosas marchen como deberían marchar, es muy bueno que un país que tiene 96% de cobertura como muy pocos países en el mundo lo tienen, entonces cuando se logró eso se debió acompañarse también de los mecanismos de financiación para para hacerlo una realidad.

P11: 4. Transcripcion Armando Gonzales_ok.docx - 11:7 [Ya se lo dije varias veces, mi..]  (25:25)   (Super)
Códigos:	[Protección financiera : financiación del SGSSS] 
No memos

Ya se lo dije varias veces, mientras no se logre la financiación del sistema de salud el futuro es muy incierto, es que usted nos ve bien a nosotros pero vaya mire el resto de entidades de salud como están, cantidades de situaciones muy complejas, pero porque la culpa aquí es un problema de financiación  y eso es un problema estructural, porque el gobierno por mas que quisiera contar con todos los recursos no va a poder contar con todos los recursos económicos, pero hay un problema igual o mayor que es la inefectividad de la atención médica o la inefectividad en cualquier empresa ,entonces mire como seria yo siempre he llegado a la conclusión, de que la única forma de que estos países subdesarrollados logren una efectividad en todos sus procesos y logren mucho mayor productividad va a tener que ver con el nivel del desarrollo del país, y el nivel del desarrollo del país no va con una sola cosa, ahí tres cosas que están unidas que son salud, educación y empleo y entonces cuando usted ve ese desequilibrio que hay, entonces usted ve como hace para tener una salud completa si las otras dos variables importantes como el empleo, y la educación no se están dando, o no se dan en la medida que se tienen que dar. Lo que quiero decir es que en un país subdesarrollado es muy difícil tener servicios desarrollados y si se da, se dan excepcionalmente, entonces digamos que la salud aquí está dada por unas clínicas muy importantes, fundación Valle del Lili, Clínica de Occidente, Centro Médico Imbanaco, pero el resto como están eso crea unos desequilibrios muy grandes, hay un sector que puede funcionar bien por x o y motivos que son bien difícil de definir, gran admiración tengo yo por la fundación valle del Lili, pero muchísima pero porque somos tan poquitas las que tenemos ese grado de desarrollo y el resto del sistema de salud está muy subdesarrollado.  Y todos tenemos el mismo problema de los recursos económicos, entonces no tiene la suficiente efectividad en las cosas que realiza, y entonces ahí lo único que se procesa es desperdicios y eso perpetúa una atención que no sea la más adecuada porque hay una gran cantidad de desperdicios que no se logran resolver. 

P11: 4. Transcripcion Armando Gonzales_ok.docx - 11:9 [Eso no tiene otra alternativa,..]  (37:37)   (Super)
Códigos:	[Protección financiera : financiación del SGSSS] 
No memos

Eso no tiene otra alternativa, o se capitalizan o se desaparecen porque la misma superintendencia de salud no las puede mantener en funcionamiento, ellas necesariamente tienen que hacer una capitalización. Coomeva hizo un programa a 7 años, y ahora con esto de cristus se puede acelerar un poquito el programa pero ese programa que hizo la EPS a 7 años es un programa muy completo para en 7 años tener las condiciones ideales, aunque me parece mucho tiempo esperar 7 años hasta que EPS pueda brindar los servicios que debe estar prestando desde ya. Pienso que ellos con cristus van a acelerar muchísimo ese proceso y obviamente la situación de EPS Coomeva cambie radicalmente. Y es un orgullo para nosotros las personas del valle porque Coomeva de las 17 empresas que tiene, la única que tiene una situación difícil es la EPS, el resto esta súper bien. 

P11: 4. Transcripcion Armando Gonzales_ok.docx - 11:10 [Yo creo que en un momento dado..]  (41:41)   (Super)
Códigos:	[Protección financiera : financiación del SGSSS] 
No memos

 Yo creo que en un momento dado lo que requieren es capitalizarse y ahí diría uno que en medio que se haga, lo importante es que lleguen recursos frescos que permitan la sobrevivencia de estas entidades.

P11: 4. Transcripcion Armando Gonzales_ok.docx - 11:11 [o que es más importante que es..]  (69:69)   (Super)
Códigos:	[Protección financiera : financiación del SGSSS] 
No memos

o que es más importante que es una financiación más adecuada no se ha podido lograr nada. La UPC nunca se logró aumentar sabiendo que la UPC es insuficiente para toda la prestación, es más llega el famoso no POS y queda dentro de la misma UPC, una modificaciones mínimas, entonces tenías unas prestaciones así (pequeñas)  y las volviste de este grande, dentro de una UPC muy baja para dar cobertura a todo eso, entonces ese es el problema más grande que pueda tener por lo tanto las EPS tienen que tratar de trabajar mucho políticamente, pero no han logrado nada porque lo dijo el ministro de hacienda y él es clarísimo que no puede aumentar la UPC porque si la aumenta se desequilibra todo el sistema económico del sistema. 


P11: 4. Transcripcion Armando Gonzales_ok.docx - 11:18 [Pero como se va a solucionar, ..]  (100:100)   (Super)
Códigos:	[Protección financiera : financiación del SGSSS] 
No memos

 Pero como se va a solucionar, si es que no hay recursos económicos para eso, yo no le veo solución, tampoco tan dramático pero realmente no la veo con ese faltante no aparece, lo último que leí en portafolio hace tres o cuatro meses, es que el sistema de salud en este momento gastaba 44 billones de pesos al año, y que se requerían mínimo 55 billones al año, ósea que hay un déficit de 11 billones de pesos, entonces de donde va a salir esa plata.

P11: 4. Transcripcion Armando Gonzales_ok.docx - 11:19 [Usted no me ha hecho una pregu..]  (104:104)   (Super)
Códigos:	[Protección financiera : financiación del SGSSS] 
No memos

Usted no me ha hecho una pregunta que debería hacer, qué es: ¿ porque nosotros sobrevivimos en un esta situación con comprometido? Y eso se debe a que nosotros atendemos el 57% de pacientes de las aseguradoras, y un 43% de particulares. Esa mezcla nos da a nosotros la solvencia financiera para poder cumplir con la mayoría de compromisos con nuestros grupos de interés, con los seis grupos de interés que tiene cualquier empresa. Eso no quiere decir que no tengamos dificultades, y que a proveedores que se les venía pagando a 50 días, haya que empezar a diferirle un poquito más, pero todavía continuamos siendo de las mejores del país al cumplir con esos compromisos. Entonces esa mezcla que nosotros tenemos entre particular que muy pocas entidades los tienen, nos permiten una solvencia financiera que nos permite cumplir con todos los compromisos con los grupos de interés, esa es la situación, ahora en la medida que nosotros estamos creciendo y que llegamos a una clínica con 360 camas, ese grupo de aseguradoras va a aumentar la saturación y ahí podríamos nosotros a empezar a tener dificultades porque ya no podríamos atender la misma solvencia, porque no es que nos disminuyan los privados, si no que nos aumentan los otros lo cual nos disuelve el porcentaje de los privados.

P12: 5.Transcripcion Carlos Fajardo_ok.docx - 12:5 [Consideramos que si no hay nue..]  (18:18)   (Super)
Códigos:	[Protección financiera : financiación del SGSSS] 
No memos

Consideramos que si no hay nuevos impuestos no hay nuevos ingresos, el sistema como tal va a colapsar, porque hoy no está permitiendo que los recursos verdaderamente alcancen, hoy  aparte de lo de salud, se está accediéndose a insumos, se está accediendo a multivitamínicos, se está accediendo a transporte, se está accediendo a situaciones que no son totalmente enmarcadas dentro del campo de la salud, pero que dada la tutela y dada las condiciones de la población de muchas necesidades básicas, se está abusando del sistema, para poder cubrir ese tipo de necesidades.

P12: 5.Transcripcion Carlos Fajardo_ok.docx - 12:14 [Yo creo que sí, este decreto e..]  (58:58)   (Super)
Códigos:	[Protección financiera : financiación del SGSSS] 
No memos

 Yo creo que sí, este decreto es una forma de poder purgar y poder estabilizar financieramente el sistema, uno, entonces las empresas que hoy tienen capacidad de recibir una  inyección de capital para poder salir adelantes, eso les va a permitir ser más eficientes. Y dos a través de unos nuevos modelos de contratación unos modelos de gestión, donde el riesgo en salud va a poder permitir con más herramientas para mejorar sus indicadores en lo que respecta  cierta estabilidad del sistema como tal de la población a la cual están asegurando. Entonces yo creo que el 2702 es una gran oportunidad, la vemos así para la organización Emssanar, hay unos inversionistas muy interesados en poder decir cuánto requieren ustedes aproximadamente nuestro defecto son de 400 millones, de los cuales estamos pendientes de una financiación, la valoración de la empresa la tendremos como en 96.000 millones entonces serian como 50.000 millones es lo que nos faltaría aproximadamente que debemos   buscar a través de inversionistas. Y más de un inversionista nos dice que nos ponen todo, pero no queremos que todo esté en un solo inversionista, si no que haya una diversidad de inversionista. Es más difícil por la formación de la alianza publico privada, pero se ha propuesto con el mismo departamento del valle si Dios quiere. Pero se parte de inversionistas, muy interesados en este modelo. 

P12: 5.Transcripcion Carlos Fajardo_ok.docx - 12:15 [Exactamente, estamos en ese pr..]  (62:62)   (Super)
Códigos:	[Protección financiera : financiación del SGSSS] 
No memos

Exactamente, estamos en ese proceso y creo que a finales de agosto ya tendremos el cambio, hoy somos una empresa solidaria en salud y pasaríamos a ser una sociedad anónima. Para poder tener el vehículo que haya inversionistas en el sector que ya los tenemos bien previstos e identificados. 


P12: 5.Transcripcion Carlos Fajardo_ok.docx - 12:16 [Si porque en esta situación de..]  (66:66)   (Super)
Códigos:	[modelo de salud futuro] [Protección financiera : financiación del SGSSS] 
No memos

Si porque en esta situación de tipo financiero no todas  van a poder cumplir sus estándares, o no van a poder lograr llenar el defecto que se calculó a diciembre de 2015 y que hay que cumplirlo en los sietes años. Hay bancos que no van a prestarles  a las EPS, entonces yo considero sobre todo que esas empresas pequeñas les va a ser muy difícil el cumplimiento, porque hoy para poder tener unos márgenes suficientes, y poder manejar las inestabilidades debe haber una gran población, si no es muy difícil con poblaciones pequeñas.

P12: 5.Transcripcion Carlos Fajardo_ok.docx - 12:17 [Si puede ser un poquito más o ..]  (70:70)   (Super)
Códigos:	[Protección financiera : financiación del SGSSS] 
No memos

Si puede ser un poquito más o menos, pero tiene que ser empresas con musculo financiero y fuertes, porque dentro del decreto esta poder tener las reservas que teníamos, antes dentro de las reservas aproximadamente es tener en caja 1.5 el promedio de facturación que tiene la EPS esa plata tiene que estar ahí, que en caso de algún problema  la EPS tiene esa plata en reserva y los puede pagar tranquilamente para evitar hoy los desbalances que se generan en los procesos de caja entre IPS y EPS

P12: 5.Transcripcion Carlos Fajardo_ok.docx - 12:22 [Si se da un cumplimiento a lo ..]  (130:130)   (Super)
Códigos:	[Protección financiera : financiación del SGSSS] 
No memos

Si se da un cumplimiento a lo que esta establecido en la 2702 en los  aspecto al segmento financiero y en la última ley que la firmo también el presidente sobre nuevos recursos de fortalecimiento al sector salud, creo que eso va a evolucionar mejor. Hoy se hizo un cambio muy sustancial en lo que respecta al financiamiento del sector  salud, ya lo manifesté anteriormente, faltan recursos, pero igualmente se ha buscado blindar para que esos recursos sean manejados más eficientemente a través del cumplimento de el plan de mejoramiento institucional y otro a través de unos impuestos que pueden generar  la bolsa que hoy se genera para poder cumplir con todo lo concerniente a la salud del pueblo colombiano, sea de mayores ingresos, entonces considero que si se dan esas perspectivas a pesar de que hoy uno tiene una economía en desbalance pero que  Colombia afortunadamente ha podido sortear adecuadamente esta situación sobre todo con la caída del precio del petróleo, le ha permitido que con esas inyecciones tener una estabilidad y creo que el sistema puede sostenerse en esas condiciones. No a mejorar si a sostenerse, los flujos si porque hay muchas medidas, por ejemplo que pasaba en el 1997 cuando no había giro directo, cuando la plata llegaba directamente a las alcaldías, se quedaba ahí y esa plata se perdió y los recursos no llegaban a las IPS, ahí sí que peor. Se quita al municipio y ahí hay flujo directo, eso mejoro mucho. Hoy están diciendo, a las instituciones que capitalizan, inyecten capital, instituciones como las EPS, busquen mejoramiento continuos en contratación, en sistemas de manejo de riesgos hay unas nuevas variables, esto va a generar que en el futuro la relación EPS IPS sea mejor en la parte de flujo y caja.

P12: 5.Transcripcion Carlos Fajardo_ok.docx - 12:20 [En los análisis que se hacen c..]  (138:138)   (Super)
Códigos:	[Protección financiera : financiación del SGSSS] 
No memos

En los análisis que se hacen cuando sale la parte de la tutela y del no POS, el régimen subsidiado era sostenible, si le quitamos el componente no POS el régimen subsidiado es sostenible, pero si hoy el componente no POS o la implementación de la  ley estatutaria donde da una cobertura mayor  muchas de las situaciones, creo que el sistema como tal no va  a ser sostenible, va a tener el mismo problema que tiene el régimen contributivo, es más la experiencia nuestra nos está diciendo hoy el régimen contributivo es más sostenible, nosotros estamos manejando dentro de lo que es movilidad el régimen contributivo, estamos llegando a acerca de 16.000 personas, que es el régimen contributivo y el régimen contributivo nos da proyecciones o mejores resultados financieros que el régimen subsidiado, porque tenemos unos componentes que uno es la vía capitación que es lo del primer nivel y como lo dijimos anteriormente el primer nivel cuando cápita paga, pero las acciones que se hacen ahí no impactan en los resultados de mejoramiento de salud de la población, porque toda esa población va a ir al segundo o tercer nivel y ahí va a ser más costoso. Dos porque hay muchas de las enfermedades hoy crónicas que han seguido creciendo y eso hace que haya más canceres que haya más niños con problemas genéticos, mas situaciones de enfermedades precursoras como el diabetes, entonces yo le diría, antes de que el no POS no era una variable que afectaba financieramente el subsidiado es viable o si se quita el no POS podría decir que es todavía financieramente viable. El contributivo si tiene la parte de transacciones económicas que es lo que a veces afecta considerablemente en la liquides o en la parte financiera del régimen contributivo. 

P13: 6 y 7.Transcripcion Diego Gomes y Gonzalo Gonzales_ok.docx - 13:12 [Las EPS no están ganando plata..]  (37:37)   (Super)
Códigos:	[Protección financiera : financiación del SGSSS] 
No memos

Las EPS no están ganando plata, las EPS están en un desequilibrio económico, solamente 1 o 2, en los últimos 2 años han tenido muy cerca el punto de equilibrio. Las exigencias que hay de captación son de tal tamaño que nadie se le va a medir a un negocio a capitalizar lo que está solicitando cuando no está dando retorno. Entonces yo pienso que eso va a reventar, ahora se ha hecho el primer esfuerzo en este primer año, que es un esfuerzo casi que diría yo de contabilidad pero en el segundo o en el tercer año, a menos que hayan entidades externas a Colombia que este interesadas en comprar las EPS, van a tener capacidad para la capitación que requieren. Porque la capitalización es, capitalización y cubrir las deudas que tienen, no es solamente la capitalización son dos grandes cargas, financieras que tiene, de manera que yo creo que muy bien pensado el tema de la capitalización porque así es que se debería manejar un negocio de aseguramiento, pero creo que después de 20 años en 7 años no se logra eso. 

P15: 9. Transcripcion Julian Duran_ok.docx - 15:4 [Si, eso podría generar una may..]  (19:19)   (Super)
Códigos:	[Protección financiera : financiación del SGSSS] 
No memos

Si, eso podría generar una mayor presión en eses sistema de salud, y va a agravar la situación que actualmente tiene. 

P15: 9. Transcripcion Julian Duran_ok.docx - 15:9 [Pues mire las EPS del régimen ..]  (59:59)   (Super)
Códigos:	[Protección financiera : financiación del SGSSS] 
No memos

Pues mire las EPS del régimen contributivo  vienen presentando unos supuestos pérdidas  y eso en el largo plazo se está acumulando y va a llegar a un punto en el cual eso va a generar una situación insostenible, y respecto al régimen subsidiado no conozco en detalle cómo es su situación financiera

P15: 9. Transcripcion Julian Duran_ok.docx - 15:10 [entonces también registra esa ..]  (63:63)   (Super)
Códigos:	[Protección financiera : financiación del SGSSS] 
No memos

entonces también registra esa pérdida acumulada en sus estados financieros y con el problema creciente que cada vez tienen más afiliados, ósea las afiliaciones están aumentando en ese régimen y eso entonces también va a generar una mayor presión sobre sus finanzas y entonces también entraría en crisis igual que el régimen contributivo. 

P15: 9. Transcripcion Julian Duran_ok.docx - 15:12 [arece ser que no se van a capi..]  (71:71)   (Super)
Códigos:	[Protección financiera : financiación del SGSSS] 
No memos

arece ser que no se van a capitalizar porque no se ve una mayor presión política y de fiscalización que las obligue a eso, las EPS parece en cierto sentido que no tuvieran control


P16: 10.Transcripcion Angela Tascon.docx - 16:6 [En lo financiero yo soy partid..]  (18:18)   (Super)
Códigos:	[Protección financiera : financiación del SGSSS] 
No memos

En lo financiero yo soy partidaria de algunas propuestas que ha hecho la dirección de las asociaciones clínicas y hospitales y es centralizar la disponibilidad de los recursos con mucho control. De hecho por eso algo que se ha logrado ahora y que ha mejorado un poco ahora los recursos que son más públicos como el del régimen subsidiado han tenido un poquito más de control más sin embargo sabemos que una tercera parte de los recursos se pierden en el camino, entonces yo diría que la descentralización de los recursos con unos bancos, digamos muy bien empoderado del manejo del dinero seria, porque plata si hay lo que pasa es que está mal direccionada y mal manejada.

P16: 10.Transcripcion Angela Tascon.docx - 16:18 [Pues nosotros particularmente ..]  (74:74)   (Super)
Códigos:	[Protección financiera : financiación del SGSSS] 
No memos

Pues nosotros particularmente en esta instituciones tamos preocupados porque por ejemplo nos están pagando diciembre del 2014, entonces el flujo ahí tiene que hacer uno milagros tiene que obviar algunas cosas, sacrificar más capacitación en el personal que redundaría en un mejor servicio asistencial, empezar a sacrificar esas cosas que no son vitales entonces el oxígeno ni los insumos, pero podría uno aportarle mas al crecimiento de la gente pero como el recurso es tan escaso si no hay una reforma de verdad, estaba pidiendo nuestro presidente de clínicas y hospitales que subieran si quiera 1 puntico del IVA, vamos a ver si llegan esos recursos pero como estamos lo veo muy complicado. 


P16: 10.Transcripcion Angela Tascon.docx - 16:19 [Lo veo muy complicado porque n..]  (78:78)   (Super)
Códigos:	[Protección financiera : financiación del SGSSS] 
No memos

Lo veo muy complicado porque no llega, porque cuando le dicen a uno 90 días, son mínimo 120, porque son 90 días en el momento en que usted radica la factura más o menos por decir un promedio pero resulta que en la radicación de la factura se le van otros 30 días, el sistema le permite a usted devolver la factura a los 30 días máximo entonces se la devuelven al día 29, entonces ya vamos en 150 entonces yo lo veo muy complicado porque yo tengo la sensación que en muchos casos y en muchas EPS, el oficio es más o menos parecido a lo que yo creo en las aseguradoras, dijéramos de autos y todo siempre es buscando donde está la falla en la petición para no pagarle, entonces en este tema en que la factura va y vuelve se pueden pasar dos o tres meses más, entonces mientras el sistema no mejore todas estos caminos que hay estos atajos, que hay para la consecución del recurso yo lo veo muy complicado, muy difícil crecer es difícil ofrecer servicios con oportunidad y con calidad mientras los recursos no fluyan. 

P16: 10.Transcripcion Angela Tascon.docx - 16:20 [Pues fue muy bueno pero descon..]  (82:82)   (Super)
Códigos:	[Protección financiera : financiación del SGSSS] 
No memos

Pues fue muy bueno pero desconozco como hacen para que ese giro directo ya no sea tan directo porque es que estamos sufriendo directamente, se supone que uno hace un convenio y unas cuotas de pago y que son por giro directo pero las EPS tienen el poder de reportar las facturas y los servicios para que eso tenga un giro directo, entonces resulta que yo estoy detectando que la EPS no está reportando lo que debe recortar para que a uno le llegue el giro directo, entonces cuando usted esta esperando e hizo un convenio para que su giro directo sea 100 millones y de pronto le llegaron 30, pero porque ellos no han reportado, no es que el fosiga no haga el giro directo, es que la EPS no ha reportado lo que le deben a usted girar y no sabemos ya al interior, por eso le digo que la falta de control, definitivamente las EPS tienen el poder porque uno sabe que les llega plata y no le llega a uno, porque no sé, le pagan a otros, pero el giro directo fue una herramienta muy buena pero me parece que las EPS tienen el poder de manejarlo, alterarlo entonces no le giran a usted lo que le quedaron de girar.

P16: 10.Transcripcion Angela Tascon.docx - 16:21 [Pues yo creo que desde que hay..]  (86:86)   (Super)
Códigos:	[Protección financiera : financiación del SGSSS] 
No memos

Pues yo creo que desde que haya voluntad de poder se puede, pero no es fácil porque hay mucha intermediación, hay mucho poder arribar el gobierno mismo es claro en que casi la tercera parte de los recursos no se sabe pa donde se van y es una cantidad, entonces el gobierno puede todo pero programado con controles con gente transparente, con gente ética, que eso es lo que nos falta mucho. Yo creo que hay voluntad al menos con este ministro uno ve que hay voluntad pero que dijéramos las barreras invisibles son muy fuertes, uno ve que la norma, esos decretos que hay de los derechos a la salud que son constitucionales, eso es voluntad porque no baja, por las barreras invisibles politiqueras no dejan bajar el recurso ni las leyes, pero voluntad si hay. 


P18: 11. Transcripcion Fabian Mendez.docx - 18:6 [Creo que el sistema no aguanta..]  (11:11)   (Super)
Códigos:	[Protección financiera : financiación del SGSSS] 
No memos

Creo que el sistema no aguanta más en términos de viabilidad financiera creo que ya está mostrando bastantes varias décadas de estar intentando un modelo lo arreglamos por un lado, lo arreglamos por acá pero no muestra señales. Y necesita una reformulaci

P18: 11. Transcripcion Fabian Mendez.docx - 18:12 [No creo.]  (31:31)   (Super)
Códigos:	[Protección financiera : financiación del SGSSS] 
No memos

No creo.

P18: 11. Transcripcion Fabian Mendez.docx - 18:23 [Es una cadena de sucesos ligad..]  (100:100)   (Super)
Códigos:	[Protección financiera : financiación del SGSSS] 
No memos

Es una cadena de sucesos ligados y ya hablábamos antes de las barreras y le mencionada yo antes como pensaba yo que los prestadores y los profesionales de la salud son otras de las víctimas de este modelo que se han visto reducidos proletarizados en un sistema o construyen cooperativas o empiezan a trabajar. La deuda que hoy tienen las IPS es más grande que la deuda de 5 años, yo no le veo a menos que ocurra esa capitalización, que hablábamos antes que esa deuda pueda ser reducida, porque lo que yo veo con indicadores o con la experiencia de ver los colegas que están en el sistema atendiendo en el negocio de la prestación de servicios, es que la situación está cada vez más crítica, cada vez más las brechas y las distancias entre prestación, la mitad la glosa y finalmente el pago esa de la prestación de los servicios cada vez se extiende más y las deudas son cada vez más grandes, uno lo ve y no estamos hablando de IPS pequeñas, de IPS grandes. 

P22: 13. Transcripcion Maria Ines Pantoja.docx - 22:10 [uera de eso el problema grave ..]  (27:27)   (Super)
Códigos:	[Protección financiera : financiación del SGSSS] 
No memos

uera de eso el problema grave del régimen subsidiado en este momento no es el POS sino lo  que  tienen que pagar los entes territoriales con la resolución 1974, eso ha sido una barrera total para los pacientes, porque las EPS venían recobrando el NO POS  pero entonces el fosiga, estaba teniendo una responsabilidad que no era de él, que era de los entes territoriales, pagar el no POS, entonces el ministerio quiso pues organizar esto, pero los entes territoriales no han tomado esto como una responsabilidad, hemos discutido mucho ese decreto porque vemos que hay una gran desigualdad, en los planes de beneficio que tanto pidio la 760 de la corte, entonces nosotros vemos que hay una brecha y estamos bregando para que se derogue ese decreto. El problema es la cuestión de fuentes de pago, en el régimen contributivo lo paga el Fosyga, y en el régimen subsidiado lo tienen que pagar los entes territoriales, el departamento, las secretarias de salud que tienen un rubro para eso. 

P22: 13. Transcripcion Maria Ines Pantoja.docx - 22:12 [Yo pienso que la UPC se hace b..]  (34:34)   (Super)
Códigos:	[Protección financiera : financiación del SGSSS] 
No memos

Yo pienso que la UPC se hace base a unos valores y costos del año anterior, eso tiene en cuenta muchas variables si la UPC de verdad no alcanzara, yo creo que las EPS no se comprometerían a prestar el servicio si no fuera suficiente si a mí me dicen, mire usted tiene que dar 50 huevos al desayuno y me dan para 20 yo digo que pena yo no los puedo dar porque usted solo me está dando para 20, entonces es algo que las EPS aceptan seguir porque es un negocio y ellos mire que con la contención del gasto no mas, en estos momentos cafesalud, la contención del gasto que ha tenido cafesalud, capital salud, Coomeva, con esa contención del gasto la UPC tiene que alcanzarles. mire cafésalud  desde que paso la administración a la nueva IPS cafesalud hace cuatro meses no le presta servicio a muchas personas eso es una contención del gasto y eso es un ahorro que ellos están haciendo. A los trasplantados a los enfermos de sida, dice uno como puede ser. Ellos han hecho una contención de gasto muy grande, yo discutía con el ministro un día que hicieron un evento, el decía que los pacientes éramos los que teníamos estancado el sistema de salud, que abusábamos, entonces yo le dije señor ministro usted es economista,  y usted tiene que tener una visión muy clara y mostrarnos cuanto han despilfarrado y desviado las EPS de Los dineros que les han dado en salud, cuanto los laboratorios han cobrado más por medicamento, la intermediación cuanto se ha llevado, la contención cuanto se ha llevado y los pacientes cuanto hemos abusado, cuando haya un panorama económico completo que usted lo puede hacer como economista, ese día si nosotros tomamos conciencia de eso, pero mientras usted diga que somos los pacientes, usted le está faltando a la verdad y a la ética, y me dijo que usted tiene toda la razón, con nosotros ha cedido muchísimo en el sentido de aceptar muchas sugerencias, de contactarnos con los técnicos que está trabajando, es una persona que ha dado un vuelco, paso de profesor universitario a ministro pues eso lo entiende uno. 

P22: 13. Transcripcion Maria Ines Pantoja.docx - 22:24 [o están recuperando con la con..]  (127:127)   (Super)
Códigos:	[Protección financiera : financiación del SGSSS] 
No memos

o están recuperando con la contención de gasto, pero con esa contención de gasto van a tener más gente enferma, eso es un círculo vicioso. Usted no le prestó atención a esta población porque quiso contener el gasto para superar un poco el déficit, resulta que esta población se le va a agravar y cuando esta población llegue a una urgencia u hospitalización esa contención del gasto, se le va a ir allá.  Yo pienso que la sostenibilidad financiera de las EPS está muy difícil, lo que uno dice porque una pérdida de esas, yo lo que no entiendo porque pierde y pierde y siguen sosteniéndose allí. 

P22: 13. Transcripcion Maria Ines Pantoja.docx - 22:25 [Es que el dinero se va para ot..]  (130:130)   (Super)
Códigos:	[Protección financiera : financiación del SGSSS] 
No memos

 Es que el dinero se va para otras cosas, si vemos en saludcoop todo lo que sucedió y que no ha sucedido nada para muchos, es que ese problema político es inmensamente grande, usted cree porque sacaron a la contralora, porque el fiscal general de la nación saco a la contralora. Cuando ella empezó a tocar a saludcoop, cuando ella empezó a hacer que le confiscaran los bienes a Palacino fue cuando él le busco el quiebre a la contralora, para que no continuara porque ella dio la orden, el superintendente nos decía que eso es un problema. Pero de todas maneras vemos como eso se ha convertido en una mafia, el sistema de salud es otro sistema de mafia de los que existen en Colombia, entonces si usted denuncia a las EPS, usted es el malo del paseo empiezan a buscarle el quiebre por algún lado entonces aquí esto no le veo yo futuro, pero mire este es el déficit, pero este déficit ellos se los han trasladado a las IPS porque ellos les venden a las IPS el dinero, entonces las IPS no nos están dando atención porque las EPS no les han pagado, entonces hacen eso por evento y por evento es imposible recuperar, porque si usted tiene convenio con red usted le puede costar una cita 40mil pesos pero usted por evento tiene que pagarla completa, 200 mil pesos, váyase usted a exámenes procedimientos, por evento y así están contratando en este momento, es la única manera que está haciendo contratación, entonces quien va a poder recuperar el dinero así, en vez de haberles dicho a las EPS mire yo tengo un convenio con la santa fe, y le debo entonces yo le voy a pagar de esta manera porque soy consciente que debo pero nunca dejo cerrar el contrato no dejo que mi convenio se acabe, pero esto es tanto volumen que ya el convenció se acabó y no hay condiciones de pago a las IPS le han dicho le pago la mitad ,le pago la cuarta parte, en unas IPS dicen pero como, y otras acceden a recuperar algo.

P22: 13. Transcripcion Maria Ines Pantoja.docx - 22:28 [Es que como se capitaliza algu..]  (142:142)   (Super)
Códigos:	[Protección financiera : financiación del SGSSS] 
No memos

Es que como se capitaliza alguien que debe tanto, ellos deben mucho dinero a la IPS entonces lo que pensamos nosotros que están haciendo es que están cogiendo la UPC de ahora para pagar un poco de deudas, pero no están utilizando la UPC para los que deben usarla, porque Coomeva está conteniendo gasto impresionando, cafesalud y capitalsalud que conocemos acá en la realidad más. 

P22: 13. Transcripcion Maria Ines Pantoja.docx - 22:29 [Yo dudo porque usted cree que ..]  (150:150)   (Super)
Códigos:	[Protección financiera : financiación del SGSSS] 
No memos

Yo dudo porque usted cree que el grupo económico va a entrar a perder, con todas las deudas que hay, no porque nadie compra algo tan malo, yo no voy a comprar algo malo, nosotros pensamos que están poniéndole una cara más bonita para que la compren. Y después van a venir nuevamente los problemas y es que mire en Colombia no tenemos la capacidad e tener una estructura tan grande, son 7 millones de personas, si no tienen ni siquiera una oficina en ciertos lugares, para atender a la gente usted cree que tienen la capacidad de manejar unos volúmenes de gente tan grande, yo no creo. Para urgencias solo tienen un sitio aquí, la 109 aunque las urgencias uno puede ir a cualquier parte, pero que hacen en las urgencias en los hospitales privados, lo mandan a la 109 asinando completamente, mire lo que hemos bregado para que le den una cita a una persona y la atiendan.


P23: 14. Transcripcion Mario Hernandez.docx - 23:8 [Pues digamos comparativamente ..]  (22:22)   (Super)
Códigos:	[cobertura] [Protección financiera : financiación del SGSSS] 
No memos

Pues digamos comparativamente es bajo con respecto a otros países entonces ha generado otra forma de control del gasto que afecta obviamente la oportunidad la calidad la pertinencia, todo y pues claro uno tiende a pensar entonces aumentemos la UPC para que aumente otra cosa, pero si la lógica de la regulación del mercado de aseguramiento en Colombia consiste en mantener un mismo valor por persona a cambio de un paquete de servicios pues el asegurador trata todo el tiempo de no gastarse ese valor, es decir hace una cantidad de mecanismos para recibir la UPC pero no gastársela en la prestación directa de los servicios, eso es lo que explica realmente todas las barreras de acceso que hay, toda la falta de oportunidad que hay, toda la falta de calidad, porque ahí se concentra su esfuerzo de rentabilidad, mientras eso no cambie auméntele la UPC todo lo que quiera y eso es como echar cada vez más en un saco roto, unas venas abiertas ahí tras hundir a la gente. Entonces sinceramente esa lógica es la que debemos discutir a fondo porque a mi juicio eso siempre mantiene una perspectiva de control del gasto, que se distancia fuertemente de las necesidades de la población, claro no puede controlar el gasto pero en función de necesidades reales epidemiológicas demográficas etc., pero no en función de necesidades de mercado y de rentabilidad de los otros sectores que se lucran con la atención en salud. 


P23: 14. Transcripcion Mario Hernandez.docx - 23:9 [No pues ahí las del régimen su..]  (26:26)   (Super)
Códigos:	[Protección financiera : financiación del SGSSS] 
No memos

No pues ahí las del régimen subsidiado han insistido que ni siquiera el tema es del exactamente el mismo valor se haga un ajuste más claro de la UPC, según los riesgos como así lo tiene el régimen contributivo, es decir en el régimen contributivo es un promedio del UPC, pero se les está pagando más por el niño menor de 1 año, por el mayor de 65, por la señora gestante, mientras que en el régimen subsidiado no hay ese ajuste a riesgo en salud, ellos están diciendo por lo menos ajuste y eso va a tener que pasar, va a haber un ajuste pero el gobierno va a decir yo los ajusto pero la misma EPS se encarga de régimen contributivo y de régimen subsidiado y permite la comunicación entre los dos, ese es el negocio que más o menos van a hacer.

P23: 14. Transcripcion Mario Hernandez.docx - 23:11 [Yo pienso que la tendencia com..]  (34:34)   (Super)
Códigos:	[modelo de salud futuro] [Protección financiera : financiación del SGSSS] 
No memos

Yo pienso que la tendencia como va es más hacia una disminución de actores y entonces son unos oligopolios o monopolios, en aseguramiento que manejan los tres regímenes prácticamente y hagan entonces sus ajustes de cuentas pero con economía de escala y por eso funcionara con un tipo de gestión del riesgo en salud similar al de káiser permanente, y quien lo está asimilando es SURA, como las EPS más eficiente supuestamente en incorporar eso y como es la cosa, pues simplemente si yo tengo 10 EPS, pues esas 10 EPS para todo el país están territorializadas, de manera que se distribuyen las zonas dispersas, primero una para cada zona dispersa Guajira por ejemplo, uno que maneja régimen subsidiado contributivo todo, después en las zonas rurales podría ser también pero repartiéndose una o dos máximo para poder articular con las grandes ciudades, y en las grandes ciudades van a terminar seguramente territorializando por localidades comunas o lo que sea, es decir, esa tendencia permite claramente que logren la economía de escala que dicen y disminuiría sus pérdidas, pero en esa reflexión no está claro porque pierden las EPS, porque y por donde se esta yendo la plata, y yo creo que ese análisis lo debería hacer un organismo de control, la contraloría, la procuraduría; el mismo Ministerio y la Superintendencia, no lo han querido hacer porque esa plata se ha ido a otras inversiones como saludcoop se demostró claramente que 1.4 billones de pesos se habían ido a cosas que no tenian nada que ver con la salud, inversiones en México, villa Valeria, cancha de golf, equipos de futbol, eso tiene algo que ver con la salud?  Y son recursos de destinación específica, entonces claro perdidas. Entonces como tengo que gastar todos los días de la UPC, entonces no tengo de donde pagar las deudas, así nos dice el ministro que son las cosas, entonces por eso tendría que capitalizarse para pagar las deudas y seguir pues no, les van a ir quitando las deudas diciendo que no son demostrables esas deudas al año ya se pierden, de manera que los prestadores si sufren la carga de esa deuda mientras las EPS van entrando a una lógica de oligopolio para recapitalizarse y manejar todo el negocio 

P23: 14. Transcripcion Mario Hernandez.docx - 23:12 [Claro lo que dice es que una c..]  (38:38)   (Super)
Códigos:	[Protección financiera : financiación del SGSSS] 
No memos

Claro lo que dice es que una cosa es lo contable y otra cosa es lo real y como las deudas aparecen por allá 5 años atrás, entonces yo las tengo que ir eliminando entonces en principio uno puede decir no esas deudas ya no cuentan y por eso puede darme utilidad y obligan entonces a disminuir a disfrazar en la parte contable lo que realmente ha ocurrido que es una extracción de rentas sistemáticas de parte de muchos actores. Entonces lo que hay es un gran engaño sinceramente, tendría uno que meterse a una investigación muy profunda de cómo esta estructurada el plan único de cuentas pero con toda la realidad del mundo, con facturas en mano cosa que nadie ha hecho, y cuando empezó a hacerlo la contraloría fíjese en lo que se metió la contralora Morelli termino perseguida por el fiscal que era el abogado de saludcoop, entonces bueno es decir aquí lo que tenemos es unas cajas negras enormes que no nos dejan entender que es lo que está pasando realmente y un esfuerzo de ajuste por la vía del oligopolio. 

P28: 17. Transcripcion-Gustavo-Morales (1).docx - 28:10 [Sin duda va a haber un proceso..]  (30:30)   (Super)
Códigos:	[Protección financiera : financiación del SGSSS] 
No memos

Sin duda va a haber un proceso de consolidación, ya vimos a Aliansalud y su grupo que lo acaba de comprar United Health, Coomeva entro a una alianza con los cristo, acá va a haber una entrada de jugadores extranjeros y una consolidación, la idea es que queden unas 5 o 7 aseguradoras muy sólidas que compitan en todo el país, y se tiene que acabar la odiosa distinción entre subsidiado y contributivo ya se está acabando de hecho, eso se tiene que acabar porque los colombianos son todos iguales, el ministro de salud lo definió muy bellamente de que estamos hablando cuando hablamos que la salud es un derecho fundamental nadie ha tratado de intentar una definición y el después de tanta reflexión lo logra, eso quiere decir que no se admiten desigualdades en el servicio de salud, el servicio en el que tienes derecho tu o yo tiene que ser el mismo que recibe un campesino del Guaviare, en eso consiste el derecho fundamental, entonces financiera mente eso significa que eso tiene que acabarse esa distinción entre EPS que castiga a los pobres y les ofrece una mejor cobertura y les ofrece una  menor UPC. Yo creo que las EPS serias se han dado cuenta que el negocio es rentable, cuando hacen gestión de riesgo, cuando invierten en tecnología y prevención y cuando invierten en prevención y eso solo lo pueden hacer las EPS con musculo financiero entonces lo que veo es una consolidación. Pero dicho eso me parece muy positivo eso que ha identificado el ministro en fortalecerse, porque ahora si van a ser aseguradoras de verdad, sino atienden el riesgo de sus afiliados tienen que liquidarse o desaparecer, pero si lo atienden tiene todo el derecho de tener una ganancia. 


P29: 18. Transcripcion Carlos Hernandez.docx - 29:3 [Eso ayuda mucho para ser viabl..]  (19:19)   (Super)
Códigos:	[Protección financiera : financiación del SGSSS] 
No memos

Eso ayuda mucho para ser viable y financieramente un sistema de salud, un país que pagan todo no importa lo que se cobre pues hoy en día están consumiendo 22% de su producto interno bruto, pero cuando uno define una prima, la prima debe de ajustarse según los riesgos no según los recursos,  entonces lo que tiene Colombia es que tiene una UPC prima plana para todo el país y no lo ajusta según el nivel de riesgo de la población, quiere decir eso que si estoy en Bogotá y mi nivel de enfermedad es distinto a la región tropical o a la región pacifica, esa UPC es la misma, no tiene diferencia regionales ni por concentración de riesgos solamente se ha ajustado por población, porque de Cali es más cara Medellín y Bogotá, pero no por concentración de riesgos, no porque hay mayor enfermedad, entonces la prima debe ajustarse para entender las necesidades de la población pero para no para atenderla como enfermedad, las necesidades de la población para prevenirla en sus factores estructurales por eso menciono que yo no debería estar ajustando la prima por ejemplo en esta región, porque tenga mas niños en cuidados intensivos, no yo debería de tener una estructura básica de prestación y aseguramiento publico para que los niños que nacen desnutridos no nazcan desnutridos y eso se tiene que hacer con condiciones de pobreza de empleo y control prenatal, no es un tema de EPS ni de control prenatal, es un tema de pobreza alimentación y de control prenatal, son los tres factores, entonces cuando le encargamos los temas de costo en salud a una UPC que es igual en todo el país, y que no conoce esos sistemas estructurales estoy enfocando un problema de salud de una manera distante a las causas estructurarles entonces si hay un tema de distancia entre la necesidad y el valor de la UPC. 

P29: 18. Transcripcion Carlos Hernandez.docx - 29:6 [CH: Hasta el momento ninguna q..]  (34:34)   (Super)
Códigos:	[Protección financiera : financiación del SGSSS] 
No memos

CH: Hasta el momento ninguna quiere capitalizar, el país tomo una serie de decisiones a través de su gobierno y le decimos el país porque todos pasivamente participamos de esto. Se tomó la decisión de hacer una cobertura universal pasar del 40 al 70 al 98% de la población igualada, decidió igualar los planes de beneficio del régimen subsidiado igual al contributivo, pero no ajusto a tiempo el valor de la prima, con eso condeno a muchos entre territorial a mucha aseguradora del régimen subsidiado a tener una crisis financiera enorme. Pero cuando también al mismo tiempo permitió que las EPS no tuviesen margen de solvencia ni las condiciones de una verdadera aseguramiento, todas las entidades financieras deben tener un capital de respaldo para y eso no se exigió. Además permitió en la contabilidad adelantar ingresos para cerrar un año bien, es decir les permitió a las entidades disimular los estados financieros en el papel, y modifico el plan de beneficio sin adicionar recursos, desde la sentencia t760 cargo una responsabilidad que administrativa y financieramente le cuesta mucho y además no paga todas las prestaciones del post, se establece una UPC para el post pero él no post crece se estimula la industria farmacéutica para que se lucre más de las entidades no post y ese costo queda extra contable en los estados financieros y no se reconoce esa deuda y a medida que se ha ido reconociendo el ministro y hay expertos que hablan de 12 billones de pesos de déficit, provocado por una parte aparte de la corrupción, segundo que lo ha intervenido ese gobierno en los últimos años, la financiaciones de medicamentos con precios liberados más costosos teniendo la fábrica en Colombia, que los demás países del mundo. Tres por una libertad para exigir derechos que no estaban en el plan de beneficio donde los jueces fallan individualmente para exigir servicios que no estaban en el post, cuatro un registro financiero falso de la realidad financiera, no es un resultado d los tres años, es un acumulado del faltante financiero que arranco desde 2008 y cambios en el estado de negocio donde el ajuste de UPC no es equilibrado para asumir los costos del envejecimiento, cuando las aseguradora tiene una prima una contingencia y si el riesgo crece se ajusta a la prima. Esto ha sido un tema que durante muchos años, los riesgos y los beneficios crecen y la prima no se ajustó, los únicos dos momentos en que se ha ajustado ha sido estos dos años, pero es un esfuerzo que ha hecho este ministro para poder empezar a tapar el faltante de cuatro cinco años atrás. Este país por otros temas tiene mas población pobre en el subsidiado que la que debería tener, la población pobre se citaban en los parques y terminaron mucha gente entre médicos en el sisben, hay profesionales en el sisben que trabajan y son formales. El ultimo estudio de planeación que presento simón Gaviria demuestra que hay un exceso en la base de población que está en el régimen subsidiado que no debería estar en el régimen subsidiado, y adicionalmente a eso el modelo de financiamiento para esa cobertura gradual era el 70% de la población trabajando informalmente y el 30% no, y la informalidad se ha mantenido en el mismo nivel en más del 50% de la población, entonces financieramente el faltante no es de estos 3 años es reconocer en los estados financieros reales, que todavía no han revelado todas las EPS, y ese faltante financiero es el que hoy en día hay que pagar en los próximos 10 años para tapar el hueco a la fecha, no considera eso los costos del envejecimiento, las nuevas enfermedades y las nuevas tecnologías.

P29: 18. Transcripcion Carlos Hernandez.docx - 29:11 [Ya está colapsado, el sistema ..]  (53:53)   (Super)
Códigos:	[modelo de salud actual] [Protección financiera : financiación del SGSSS] 
No memos

Ya está colapsado, el sistema en este momentico en 30 y pico de años, 5 años de crisis llego al punto de no retorno, en este momentico hay más recursos para pago directo de los no post, no hay compra de cartera para las EPS, ya la cartera de las clínicas paso de 120  a 160 días, en este momentico el incumplimiento de la situación financiera no lo cumple ninguno, este año varias aseguradoras están perdiendo en los resultados operacionales que nunca habían tenido y algunos cuyos resultados operacionales eran buenos, tampoco alcanzan a tapar el resultado personal. Este momento es el punto de quiebre de no retorno, y si el tema de la reforma tributaria no se hace y no se ajusta la UPC esto no tiene retorno.

P30: 19. Transcripcion Felipe Garcia.docx - 30:26 [FG: Porque quien presiona en e..]  (127:127)   (Super)
Códigos:	[Protección financiera : financiación del SGSSS] 
No memos

FG: Porque quien presiona en el sistema es supuestamente el curador, el mago que va a curar el que te provee el elixir hablando así en términos metafóricos para que te cures, no es el medico ni la EPS es la farmacéutica y es quien presiona políticamente. 


P31: 20.Transcripcion Hellen Mendoza.docx - 31:2 [o, acuérdese profe que cuando ..]  (11:11)   (Super)
Códigos:	[Protección financiera : financiación del SGSSS] 
No memos

o, acuérdese profe que cuando comenzó el post se había hecho un cálculo para el UPC que la hizo el ministro que en paz descanse y se hizo en base a unos servicios iniciales, con el paso del tiempo fueron añadiendo cosas al post tecnología, servicios, y los ajustes de la UPC que se hicieron durante un periodo importante en la historia del sistema de seguridad social en salud en Colombia, no fueron lo suficientemente fuertes como para sostener toda la tecnología y todas las inclusiones que el post tuvo, nosotros llevamos 3 años si mi memoria no falla en donde hemos tenido unos ajustes de la UPC importantes para el aseguramiento, pero incluso con los ajustes que se han hecho ahora, los servicios prestados la cobertura que hay y la UPC existente pienso yo que no es sostenible, esa UPC para todo lo que se brinda con un post. 

P31: 20.Transcripcion Hellen Mendoza.docx - 31:3 [sted tiene hoy una cobertura, ..]  (15:15)   (Super)
Códigos:	[Protección financiera : financiación del SGSSS] 
No memos

sted tiene hoy una cobertura, El sistema colombiano es el sistema con menor gasto de bolsillo del mundo nuestro sistema cubre el 100% de todo porque lo que no cubre el post lo cubre la tutela y lo que no cubre la tutela lo cubre el no post, y lo metemos por CTC por MYPRES por como usted lo quiera llamar pero hoy un colombiano tiene acceso a pañales a acceso de servicios públicos, a pañitos húmedos a crema, a pago de empleada de servicio, y a una cantidad de cosas que no están directamente relacionadas con la prestación de servicio prese, entonces usted no puede tener la prestación de un universo con un presupuesto finito, es absolutamente imposible usted lograr que ha una interrelación entre lo prestado y lo efectivamente pagado, eso es lo que tiene a un pie de un colapso. 

P31: 20.Transcripcion Hellen Mendoza.docx - 31:6 [La unidad de pago por capitaci..]  (19:19)   (Super)
Códigos:	[Protección financiera : financiación del SGSSS] 
No memos

La unidad de pago por capitación tenemos que tenerla hay que recalcularla, la unidad de pago por capitación que hoy existe para solventar los pagos de salud no es suficiente, para poder garantizar la prestación del servicio. Capacidad instalada, nosotros en Colombia no tenemos camas suficientes para atender a la población y la distribución de la capacidad instalada a su vez en Colombia no está bien organizado, nosotros hoy hemos permitido que numerosas clínicas y hospitales en el país hayan cerrado la atención de maternidad y pediatría porque son los servicios que menos rentabilidad financiera presta, eso nos está llevando a un problema de salud publica, nosotros hoy no tenemos suficiente clínicas y hospitales donde se atienda estantes donde se atienda partos y población menor de 14 años, eso afecta más la capacidad instalada, si usted va hoy en Cali haber quien atiende pediatría va a ver que el Pareto de la población clínica Versalles y el club Noel, la gente está literal en los pasillos y el club Noel literal en los pasillos que tienen adentro del club Noel porque no hay donde ubicar a los pacientes, las maternas las atienden en los partos en la clínica Versalles, ya no hay más donde atender maternidad y acá no pasa nada, acá todas las clicas van cerrando los servicios menos rentables, hace más o menos 15 días renunciaron todos los pediatras de la clínica farallones, refiriéndose según ellos a dificultades irreconciliables con la alta dirección y no pasa nada y la población de Coomeva la  pediatría hoy no tiene donde atender. Y así fue pasando todo se acabó maternidad en remedios, se acabó maternidad en clínica de occidente, Imbanaco está orientado al mercado de la prepagada, fundación valle del Lili está orientada al mercado de alta complejidad como debe ser para una institución de este nivel, pero quien atiende a la mediana y a la baja, se nos olvidó entonces eso significa que no tenemos capacidad instalada para atender a lo que tenemos que atender. Sostenibilidad financiera pues se cae su peso en la unidad de pago por capitación no alcanza, hoy tenemos unas dificultad grande de cartera, las instituciones prestadoras de servicios de salud con corte a hoy 30 de abril no han recibido nada de pago en lo que lleva recorrido del año, nosotros tenemos una rotación de cartera fácilmente mas de 160 días, teniendo que hacer pago de proveedores y de absolutamente todo lo que tenemos que subsanar y no tenemos plata para soportar la prestación. Los modelos de prestación en salud usted como garantiza la atención de un modelo de atención en salud cuando se vuelve difícil garantizar la prestación, es muy complicado tenemos problemas de autorizaciones, tenemos problemas de temas de direccionamiento de algunas aseguradoras de algunos pacientes que tienen, entonces es complicado sin embargo las IPS en mi modo de ver están trabajando y han tratado en la medida posible la continuidad de los modelos de atención.


P32: 21.Transcripcion Oscar Gutierrez.docx - 32:5 [OG: No y ese es un problema y ..]  (20:20)   (Super)
Códigos:	[Protección financiera : financiación del SGSSS] 
No memos

OG: No y ese es un problema y es que se volvió un sistema muy costoso de operar, demasiado compartido donde la relación costo beneficio, se diluye en tantas partes que al final el beneficio que se obtiene se obtiene a un costo muy superior, la sumatoria de los costos no se traduce en sumatoria de beneficios, entonces este sistema como tal es inviable

______________________________________________________________________

Código: Que debe conservarse del sistema de salud {18-0}

P 8: 1. Transcripcion Martha Lucia  ospina.docx - 8:19 [No pero me iría por las asegur..]  (23:23)   (Super)
Códigos:	[Que debe conservarse del sistema de salud] 
No memos

 No pero me iría por las aseguradoras, y porque no me iría por un sistema único de salud, porque no me gustaría tener un monopolio no por el estado ni por nadie, quisiera tener competencia porque las evaluaría por resultados, pero adicionalmente ellas estarían en regiones ofertarían por regiones estarían por un tiempo y la gente misma podría decir si la aseguradora le funciona o no.

P 9: 2. Transcripcion Alex Duran_ok.docx - 9:3 [El modelo de aseguramiento deb..]  (7:7)   (Super)
Códigos:	[Que debe conservarse del sistema de salud] 
No memos

El modelo de aseguramiento debe conservarse,

P 9: 2. Transcripcion Alex Duran_ok.docx - 9:4 [la figura de aseguramiento, el..]  (7:7)   (Super)
Códigos:	[Que debe conservarse del sistema de salud] 
No memos

la figura de aseguramiento, el tema de la equidad, creo que es un valor del sistema, y el tema de conservar el gasto de bolsillo que tiene el sistema.

P11: 4. Transcripcion Armando Gonzales_ok.docx - 11:3 [Yo lo miro un poquito diferent..]  (9:9)   (Super)
Códigos:	[Que debe conservarse del sistema de salud] 
No memos

Yo lo miro un poquito diferente, todo esto tiene que ver con la ley 100 de 1993, donde se amplía la cobertura que era del 15% a más del 90% y hoy en día es una cobertura casi universal con el 96%. 

P12: 5.Transcripcion Carlos Fajardo_ok.docx - 12:3 [De los elementos o de las vari..]  (7:7)   (Super)
Códigos:	[Que debe conservarse del sistema de salud] 
No memos

De los elementos o de las variables que actualmente el sistema tiene, uno que debe conservarse es el aseguramiento como tal porque eso permite que toda la población tenga acceso a un sistema de seguridad social, hoy Colombia tiene cerca de un 96%  o 97% en el aseguramiento, sin distingo de rezos, credos o razas.

P13: 6 y 7.Transcripcion Diego Gomes y Gonzalo Gonzales_ok.docx - 13:3 [Yo pienso que la cobertura uni..]  (7:7)   (Super)
Códigos:	[Que debe conservarse del sistema de salud] 
No memos

Yo pienso que la cobertura universal, me parece que el país dio un gran salto al tener la posibilidad de que todas las personas tengan una seguridad social en salud ese primer elemento, el segundo elemento es la equidad, que las personas puedan recibir lo que requiere, tercero algo que nos ha concretado es el tema de la gestión del gobierno, es muy importante que trabajemos en promoción y prevención, especialmente para trabajar los riesgos primarios y todo el tema de atención secundaria 

P13: 6 y 7.Transcripcion Diego Gomes y Gonzalo Gonzales_ok.docx - 13:4 [El modelo de aseguramiento de ..]  (9:9)   (Super)
Códigos:	[Que debe conservarse del sistema de salud] 
No memos

El modelo de aseguramiento de cobertura universal, esa ha sido una conquista espectacular, una construcción social importantísima para la sociedad colombiana, la posibilidad de tener cobertura total de aseguramiento, modelo de aseguramiento con cobertura total

P16: 10.Transcripcion Angela Tascon.docx - 16:3 [Yo diría que la universalidad ..]  (7:7)   (Super)
Códigos:	[Que debe conservarse del sistema de salud] 
No memos

Yo diría que la universalidad que es un logro muy importante, pero no aposta dijéramos de la parte privada por decirlo de alguna manera, si no que el gobierno tiene que adoptar esa universalidad pero por si mismo, no asegurándose en otros sería una parte.

P18: 11. Transcripcion Fabian Mendez.docx - 18:4 [Yo creo que necesita una refor..]  (7:7)   (Super)
Códigos:	[modelo de salud futuro] [Que debe conservarse del sistema de salud] 
No memos

Yo creo que necesita una reformulación de fundamentos, yo creo que en labores de inspección de vigilancia y control, todo el asunto de la vigilancia de la calidad como esta formulado, es decir si uno mira el sistema en términos de cómo esta formulado hay elementos que son válidos pero la intermediación no estoy de acuerdo en la existencia de un modelo como el actual con intermediarios, creo que ha sido muy negativo para el sistema, creo que un sistema fundamentado en relaciones económicas no es lo que el país necesita, en ese sentido yo cambiaria casi todo cambiaría el sistema por un sistema único de salud, por encuestas manejado por el estado, los privados podrían tener un espacio pero siempre bajo la regulación y control del estado como su principal ente rector y no dejándole muchas de esas funciones como ha ocurrido en los privados

P19: 12. Transcripcion Fabio Osorio.docx - 19:3 [La salud en Colombia se volvió..]  (7:7)   (Super)
Códigos:	[Que debe conservarse del sistema de salud] 
No memos

 La salud en Colombia se volvió un negocio desde la ley 100 del 93 yo creo que tiene que haber otro sistema de salud, otras normas que lo rigen porque la ley 100 del 93 se volvió una colcha de retazos de otra resoluciones de otros decretos que fueron modificando lo que hicieron en la ley 100. La ley 100 del 93 para mi está pensada en la economía y en el negocio  no en lo social y en la prestación de un servicio que fundamente el derecho a la vida como lo consagra la constitución, yo considero que debe haber una estructura de fondo no pañitos de agua tibia para que este sistema de salud entienda que debe ser visto con una rentabilidad social en salud y no con una rentabilidad económica financiera como lo hacen ahora las diferentes EPS, los diferentes centros hospitalarios que privilegian el cobro antes de la atención. En este HUV primero se atiende y después se cobra que es la gran diferencia de la clínica priva, que en ella si tu no pagas no te van a atender acá se te atiende y cuando se le va a cobrar a la EPS o a la gobernación es un problema tenaz, porque no le pagan esos dinero y por eso la crisis de salud pública está más afectada por un hospital de una clínica privada porque ellos piensan en el negocio y no en el beneficio de la salud

P24: 15. Transcripcion Martha Balbuena.docx - 24:8 [hay que tener el ministerio y ..]  (54:54)   (Super)
Códigos:	[Que debe conservarse del sistema de salud] 
No memos

hay que tener el ministerio y las EPS digamos se conservaría pero como hacemos para que la gente realmente si haga lo que debe hacer, ahí entra algo muy importante que aquí la justicia a este señor que se robó una pasta de Maggie terrible y todos los ladrones de cuellos blanco que tenemos siendo doctores y con títulos, porque está totalmente esa parte ética perdida

P26: 16. Transcripcion Senador Ospina.docx - 26:3 [Se deben de conservar las EPS ..]  (7:7)   (Super)
Códigos:	[Que debe conservarse del sistema de salud] 
No memos

Se deben de conservar las EPS siempre y cuando se constituyen no como solamente pagadores o aseguradoras, sino que se constituyen como organizaciones que conformen y consoliden capital social las EPS son nuevas instituciones que tienen una capacidad afiladora una capacidad de llevar el registro de los individuos, el conocimiento de los mismos. Si las mismas evolucionaran a entidades gestoras efectivas adelantando construcción de asociatividad colectivo, capital social, las mismas tendrían futuro. Se debe mantener lo que han ganado los hospitales en relación a hospitales  clínicas en relación a las estructuras de costos, a los manejos eficientes de la prestación del servicio y adicionalmente la ley debiese mantener los capítulos gruesos que diferencian el tema pensional con el tema de riesgos laborales con el tema de salud.

P28: 17. Transcripcion-Gustavo-Morales (1).docx - 28:4 [el principio de participación ..]  (15:15)   (Super)
Códigos:	[Que debe conservarse del sistema de salud] 
No memos

el principio de participación privada, yo creo que con una fuerte regulación y con unas reglas muy claras como ya se ha visto en estos 25 años de existencias del sistema, al sistema y a los colombianos les va mejor cuando hay participación privada, que cuando todo está en manos del estado e incluso voy a decir más, esa participación privada puede ser mejor cuando el privado tiene ánimo de lucro que cuando no lo tien

P28: 17. Transcripcion-Gustavo-Morales (1).docx - 28:5 [Fíjese que las grandes crisis ..]  (15:15)   (Super)
Códigos:	[Que debe conservarse del sistema de salud] 
No memos

 Fíjese que las grandes crisis de las EPS que la ha habido y han sido gravísimas, han sido de entidades privadas sin ánimo de lucro, saludcoop, algunas cajas de compensación, las EPS indígenas, etc. En cambio, las EPS que salen constantemente mejor calificadas en el ranking del propio ministerio de salud son las EPS que se han conformado a sí mismas como EPS con ánimo de lucro, SURA, sanitas, SOS, salud total, entonces hay que desestigmatizar el ánimo de lucro y aportarle más al sector privado y no nos puede dar pena decir que la salud es un negocio, lo ha sido toda la vida para las clínicas privadas, lo ha sido toda la vida para los médicos, también lo tiene que ser para las aseguradoras. Lo que pasa es que eso tiene que venir acompañado de una muy fuerte regulación que es en lo que estamos muy débiles. Eso sería una cosa que yo conservaría y fortalecería, y la otra es el sistema de aseguramiento, yo creo que hay países que han logrado a partir de un sistema de impuestos generales ofrecerles a sus ciudadanos un sistema de salud más o menos sólido, y ese podría ser un camino, pero tenemos que tener una tasa de tributación del 60 o 50% que hoy en día no la tenemos y no se ve políticamente viable. El sistema de aseguramiento me parece que es una de las formas más eficaz que la humanidad ha descubierto para redistribuir los riesgos sociales,

P30: 19. Transcripcion Felipe Garcia.docx - 30:2 [G: Haber conservar, la ley est..]  (11:11)   (Super)
Códigos:	[Que debe conservarse del sistema de salud] 
No memos

G: Haber conservar, la ley estatutaria está intentando rescatar un sistema que era interesante, y era que antes de la ley 100 la medicina era inequitativa estaba restringida a una población, que vamos a llamar elite y la otra era atendida algo así que llamemos urgencia, pobreza todo este tipo de cosas, lo importante del sistema es conservar un sistema donde haya prestadores de servicio en salud  eso si es importante mantenerlo porque están creando una red, por ejemplo algo que hay que conservar y creo que debe conservarse es el sisben o por lo menos la atención a los estratos más bajos, eso debería conservarse pero en términos de calidad está disminuyendo. Entonces para mi conservar eso es importante una estructura que ya se creó a nivel de asociaciones llamémosla EPS o IPS que ya le dan una estructura a la salud en Colombia, eso sí rescataría. Como hacer que esas EPS si juegan en un sistema articulado es otro problema, rescataría eso y la labor que ha hecho el estado de mantener ciertos niveles de atención en los estratos más bajos, pero con calidad pero yo si rescataría eso del sistema. Y la ley de talentos hay que conservarla, que es la ley que pone a  que usted que ha prestado el servicio de salud debe estar capacitado para eso, hay que conservarlo porque eso le daría la calidad al servicio de salud en Colombia. Yo creo que esas tres grandes estructuras jurídicas deberían conservarse. 

P31: 20.Transcripcion Hellen Mendoza.docx - 31:1 [todo los elementos que usted m..]  (7:7)   (Super)
Códigos:	[Que debe conservarse del sistema de salud] 
No memos

 todo los elementos que usted menciona debe conservarse, porque los elementos que usted menciona hacen parte de un todo nosotros necesitamos que la cobertura siga y que sea una cobertura real, no solamente una cobertura de tener un carnet, una cobertura que debe ir orientada a que la persona tenga accesibilidad, la unidad de pago por capitación es el mecanismo que tenemos de reconocimiento de los servicios prestados la capacidad instalada que es indispensable para poder garantizar la cobertura, la sostenibilidad financiera porque sin esto no hay forma de continuar ni la prestación ni el aseguramiento, los modelos de atención en salud porque son las formas que tenemos de hacer la prestación del servicio y debe haber un sistema de gestión de la calidad que garantice que la atención que estamos dando tenga la cobertura que necesita en una capacidad instalada y suficiente en donde la unidad de pago por capitación sea lo suficiente mente fuerte para que nos garantice una estabilidad financiera para todos, entonces todos los elementos que están ahí deben ser conservados

P32: 21.Transcripcion Oscar Gutierrez.docx - 32:3 [OG: No sabría que conservaría,..]  (15:15)   (Super)
Códigos:	[Que debe conservarse del sistema de salud] 
No memos

OG: No sabría que conservaría, lo que le podría decir s lo que paso, la salud es una función del estado, debe ser una función del estado y en algún momento cuando se creó el seguro social, él se creó en el 52, pero basado en el modelo ingles de aseguramiento, el problema es que en ese momento lo hizo el ministerio de trabajo no el ministerio de salud, para los trabajadores, y la idea era cobertura universal entonces uno de los elementos que yo preservaría del sistema actual es la cobertura universal. Que pasó en el antiguo sistema que como todo lo que pasa en Colombia se corrompió, no había sistema más corrompido y costoso que el seguro social, un poquito menos que el sistema dependiente del gobierno las secretarias o el ministerio de salud, se corrompió y se desvirtuó. Entonces el gobierno de la época, encontró desde la época de López Michelsen que dos sistemas paralelos de salud eran muy costosos y solucionaban pesimamente el problema de salud del pueblo colombiano y desde esa época empezaron a tratar de compaginar los dos sistemas de salud paralelos, ISS, y el sistema de ministerio de salud pero el problema es político de poder, el ministro de trabajo no se iba a dejar quitar el ascendiente que tenía sobre la clase trabajadora, en esa época las sindicales obreras eran muy poderosas, entonces tuvieron que idearse una maroma para acabar con el seguro social y fue crear u n ministerio transitorio que se llamó ministerio del trabajo y la seguridad social, aboliendo el servicio el ministerio de salud y aboliendo el ministerio de trabajo, entonces unificando los dos ya no tendríamos dos sistemas, y entonces eso le permitió tener recursos para extenderlo, pero vino la reforma que para poder hacer eso necesitaban una estructura el costado no lo tenía, entonces hizo algo que me pareció muy hábil pero no sé si estaba bien, que fue entregarle a la empresa privada, tercierizo en ese momento la prestación del servicio de la salud, y entonces usted tenía que meterse en una empresa privada y el gobierno dijo yo le doy la salud pero vigilo que se la den, y yo más bien le administro, usted me da la paga a mí y yo le pago a él y después de un tiempito cuando ya tenía bajo su mano el control volvió y separo, ministerio de trabajo y ministerio de salud, pero ya lo unifico y dejo pensiones en una parte, y la prestación de salud en otra. Entonces usted me dice wau rescataría, uno la posibilidad de primero la unificación de 2 sistemas o 4 sistemas, lo que pasa en México, hay 5 o 6 sistemas paralelos a cuan más ineficiente y más costoso que se presta mucho mas para la corrupción, que se aceptaría, la unificación de un sistema que bueno o malo da la posibilidad de hacer cobertura universal.

P32: 21.Transcripcion Oscar Gutierrez.docx - 32:4 [Dos rescataría que la població..]  (16:16)   (Super)
Códigos:	[Que debe conservarse del sistema de salud] 
No memos

Dos rescataría que la población por primera vez acepto que la salud es un derecho no una conquista, es un derecho nato, es que el cambio de personalidad es impresionante, yo me acuerdo cuando era pequeño y las empleadas del servicio se enfermaban y las mandaban para la casa,  y entonces usted va al sanjuán de Dios y usted ve nada, y la gente se enfermaba y nada, s usted era sindicalizado el seguro que era una clínica de primer mundo mejor que cualquiera. Y si era de una hernia iba donde el chamán, el taita el curandero y si no se dejaba morir. La salud es un derecho y pelea y tiene tantas razones en rebotarse cuando el servicio es malo porque como eso no es una empresa privada usted tiene que dar un buen lucro.

______________________________________________________________________

Código: Sin duda va a haber un proceso.. {0-0}
______________________________________________________________________

Código: Situación de la salud: causas de mortlidad {10-0}

P28: 17. Transcripcion-Gustavo-Morales (1).docx - 28:17 [Lo que pasa es que la mayoría ..]  (78:78)   (Super)
Códigos:	[Situación de la salud: causas de mortlidad] 
No memos

Lo que pasa es que la mayoría de las enfermedades crónicas no transmisibles, se originan en la propia conducta personal la gran mayoría de los tipos de cáncer, por ejemplo, el de pulmón porque la gente fuma, la obesidad produce todo tipo de problemas metabólicos, entonces yo te diría que la razón es porque el sistema está mirando la enfermedad no la prevención. Ahora yo no tengo la varita mágica para evitar que un sistema vea la prevención, pero le tengo mucha fe a la tecnología digital, una tecnología que permita monitorear tu peso tu ritmo cardiaco diariamente conectados a unos monitores en algún centro de salud de tu aseguradora, eso tiene que funcionar tarde que temprano y esta empezando a funcionar en algunos lugares del mundo. Entonces la respuesta corta a tu compleja pregunta es esta, el sistema está montado sobre incentivos para remunerar la atención a la enfermedad no la atención a la prevención, y yo creo que la prevención se va a volver un gran negocio cuando implementemos la tecnología digital, y ese es el gran desafío del sistema.


P29: 18. Transcripcion Carlos Hernandez.docx - 29:7 [Nosotros hemos crecido, lo que..]  (38:38)   (Super)
Códigos:	[Situación de la salud: causas de mortlidad] 
No memos

Nosotros hemos crecido, lo que se ha proyectado es que en los próximos 10 años vamos a tener el 23% de la población envejecida, y cuando uno tiene eso en términos económicos hay una cosa que se llama la enfermedad de Baumol, una enfermedad derivada de generarle a la población una mayor expectativa de vida, en la medida que los países logran que sus habitantes tengan mejor expectativa de vida, está generando un incremento de 8 veces el costo de salud por haberle dado mayor expectativa de vida, porque se produce eso porque si la gente antes vivía 60 y un infarto a los 60 tiene un costo distinto que un infarto a los 80, cuando usted tiene un infarto a los 80 le falla el riñón, le falla el cerebro y las lesiones de ese infarto tienen un costo 8 veces más que las lesiones que ocasiona un infarto en la edad más temprana, el costo del envejecimiento es un costo que todos los países han venido padeciendo y buscando mecanismos de intervención, un sistema que solamente le da una prima plana, que no tiene en cuenta el riesgo y encima de eso el envejecimiento, esta trasladando ese riesgo financiero a la prestación y la prestación a las condiciones de atención de la población y al final del día el impacto es en la calidad de vida de la gente que han atendido y en los profesionales que les han precarizado sus ingresos. Hay experiencias donde la gente en la medida que tiene más edad comienza a consumirte ciertos servicios que no deberían de ser hospitalarios y deberían de ser ambulatorios e incluso en el hogar, hoy en día más del 90% de los pacientes que deberían morir en su casa con condiciones dignas, mueren en unidades de cuidado intensivo a un costo enorme sin generar valor agregado.


P30: 19. Transcripcion Felipe Garcia.docx - 30:18 [Después lo coronario usted com..]  (81:81)   (Super)
Códigos:	[Situación de la salud: causas de mortlidad] 
No memos

 Después lo coronario usted come mucha grasa mucha cosa, entonces son problemas del entorno y si usted lo maneja en un sistema integral con especialistas esta mejor manejado. 

P31: 20.Transcripcion Hellen Mendoza.docx - 31:10 [Porque uno mira las edades de ..]  (27:27)   (Super)
Códigos:	[Situación de la salud: causas de mortlidad] 
No memos

Porque uno mira las edades de la gente, la población colombiana donde está, el Pareto de la población colombiana esta, nosotros tenemos un peso importante entre los 45 y 49 años, tenemos una población envejecida pero ha ido creciendo pero también nos están creciendo los niños, adonde vamos nosotros a impactar con el MIAS, a la población más joven en los niños y no los adolescentes, el viejito que tiene la hipertensión o diabetes el adulto que tiene el cáncer, ahí no podíamos impactar con el MIAS como si podremos impactar en un niño que está en crecimiento y desarrollo, y el que esta arrancando hoy, en 20 años va a ser un adulto joven sin hipertensión, sin diabetes, sin enfermedades asociadas a riesgo cardiovascular, porque desde pequeñito los hemos cogido y les hemos enseñándolos hábitos de prevención. Nosotros hoy no estamos acostumbrados a cuidarnos, tomamos trago, fumamos, trasnochamos, comida chatarra, porque asumimos que tenemos el derecho a enfermarnos y la clínica tiene la obligación de atendernos porque la EPS tener que pagar, nosotros hoy no estamos diciendo que si nosotros garantizamos nuestro autocuidado, no va haber rentabilidad financiera, pero va a haber rentabilidad social

P32: 21.Transcripcion Oscar Gutierrez.docx - 32:2 [OG: Ese es el temas que tengo ..]  (11:11)   (Super)
Códigos:	[Situación de la salud: causas de mortlidad] 
No memos

OG: Ese es el temas que tengo con ese proyecto de medicina regenerativa porque lo que estamos buscando es soluciones radicales, si nosotros lográramos encontrar la solución a la diabetes, las compañías farmacéuticas que venden droga implemento cuidado a los diabéticos, entrarían en proceso de quiebra, teniendo en cuenta que el 8%  de la población colombiana es diabética, el 15% de la población Mexicana es diabética, el 8.1%  de la población brasileña es diabética, el mercado es gigantesco, usted es ingeniero y conoce lo que eso implica industrial. Este es el problema real de lo que se viene para el futuro. Usted me está preguntando o quiere hablar sobre el 2030 y este es el problema actual del mundo.

P32: 21.Transcripcion Oscar Gutierrez.docx - 32:6 [El cuadro de la salud cambio p..]  (20:20)   (Super)
Códigos:	[Situación de la salud: causas de mortlidad] 
No memos

El cuadro de la salud cambio porque la demografía del país cambio, entonces yo acá le tengo la cuestión de la declaración de Cristian Grant, los ancianos viven demasiado y esto es un riesgo para la economía global, tenemos que hacer algo ya. Entonces de una demografía de niños, una pirámide de base ancha estamos pasando a una pirámide angosta o a una pirámide rectangular y casos como el de la universidad del Valle, pirámides invertidas o lo que está pasando en los países desarrollados, Japón hoy en día el 30% de la población tiene más de 60 años, en Europa en promedio mas del 27% de la población europea tiene mas de 60 años, usted sabe que hay pueblos en Europa, ciudades en España que han tenido que cerrar escuelas porque no hay niños. Entonces la prevalencia de las enfermedades están cambiando de infeccionas a traumáticas, a enfermedades crónicas no transmisibles que son las que vienen con la edad, muchas de las cuales ni siquiera sabíamos que existían las enfermedades de la degeneración no sabemos ni siquiera cómo se maneja, la manejamos como pensamos que manejamos a otro viejito hace años, pero cuando tengamos 10% de la población con enfermedades cardiacas que vamos a hacer, o 15% de la población total con enfermedades pulmonares. Entonces el enfoque ya no es salud preventiva para que a uno no le dé una infección ahora la prevención esta en evitar que el viejo se enferme tan pronto como podamos, porque no le vamos a  la alargar la vida, la idea es alargarle el estado de bienestar para que se enferme el día antes de morirse. 

P32: 21.Transcripcion Oscar Gutierrez.docx - 32:7 [OG: Mire esto se debió haber p..]  (24:24)   (Super)
Códigos:	[Situación de la salud: causas de mortlidad] 
No memos

OG: Mire esto se debió haber previsto hace 48 años cuando yo entre a la universidad, el crecimiento poblacional de Colombia estaba alrededor de 6 7% anual, eso lo podría usted averiguar, las familias tenían 7, 8, 10 hijos yo vengo de una familia de 10 hijos. Y había esa policía de poblar el país porque poblar el país traería desarrollo, cuando yo entre a la universidad las dos ciudades de máximo crecimiento en Latinoamérica era sao paulo y Cali, y alguien preveo que eso que estaban haciendo era insostenible, porque el país no tenía la capacidad para sostener educar y darle salud a este gentío, entones aun en contra de la oposición de la iglesia católica vino profamilia que es una entidad privada pero alentada por el gobierno que empezó las campañas de control de la población y han sido muy exitosas porque en este momento el crecimiento de la población en Colombia está alrededor del 2.1%  que es exactamente el de mantenimiento de la población. Pero no estamos creciendo, porque nacen menos niños, que los que se mueren viejos, porque simultáneamente a diferencia de los países escandinavos o Japón, nosotros saltamos a una expectativa de vida de 48 años los hombres 50 las mujeres, a 76 o 78  los hombres, 80 las mujeres y eso que todavía estamos con la secuela de la violencia, lo que mata gente es la violencia interpersonal que es producida por las tenciones sociales de la urbanización acelerada no selectiva. Ahora que estábamos viendo esto del postconflicto que ha creado tanta expectativa tanta emoción, tanta cosa me puse a ver las estadísticas del salvador que vino el presiente a la firma y que a boca llena decía 25 años a fin de la guerra y este año el salvador bate el record de muertes violentas, por encima de lo que tenía durante el punto máximo de la guerra civil, 120 muertos por cada 100000 habitantes. Entonces el conflicto en Colombia no mata tanta gente como la intolerancia interpersonal, eso me asusta porque eso tiene incidencia sobre las expectativas del tipo de enfermedad que vamos a ver en los próximos 10 año

P32: 21.Transcripcion Oscar Gutierrez.docx - 32:8 [: Uno lo que llamamos enfermed..]  (28:28)   (Super)
Códigos:	[Situación de la salud: causas de mortlidad] 
No memos

: Uno lo que llamamos enfermedades crónicas no transmisibles las producidas por el envejecimiento, cardiovascular pulmonar endocrina y del aparato locomotor y mentales. Va haber una que otra infecciones pero poquito, trauma va a aumentar violentamente no sé si por violencia pero si por accidentes. Esta mañana oía que Cali esta prácticamente acogotada por la cantidad de accidentes de los motociclistas 8 a 10 diarios con lesionados, y lesiones de accidente de tránsito las secuelas pueden durar años. 

P32: 21.Transcripcion Oscar Gutierrez.docx - 32:9 [OG: Seguida por la violencia y..]  (32:32)   (Super)
Códigos:	[Situación de la salud: causas de mortlidad] 
No memos

OG: Seguida por la violencia y va a seguir durante mucho tiempo, pienso yo si el gobierno o los estadistas no asumen que el problema no es acabar con el conflicto tienen que hacer una reforma estructural como la que se quiso hacer hace 40 años pero las fuerzas como usted dice no las favorece. 

P32: 21.Transcripcion Oscar Gutierrez.docx - 32:10 [OG: En los países desarrollado..]  (36:36)   (Super)
Códigos:	[Situación de la salud: causas de mortlidad] 
No memos

OG: En los países desarrollados estamos viendo un fenómeno que no vamos a ver todavía nosotros que es el cierre de hospitales. La cuestión es esta el sistema tiene que caminar hacia buscar a las personas hasta su sitio de vivienda de trabajo para hacerle mantenimiento, si usted tiene un carrito viejo usted le va a dar más mantenimiento, pero nosotros tenemos un sistema que la mayor  parte de mantenimiento es a los nuevos porque venimos de la época de las enfermedades infecciosas y a los viejos los consultan cuando ya se les cayó el motor. 

______________________________________________________________________

Código: TECNOLOGIA {17-0}

P30: 19. Transcripcion Felipe Garcia.docx - 30:8 [Yo pienso que hoy en día la te..]  (38:38)   (Super)
Códigos:	[TECNOLOGIA] 
No memos

Yo pienso que hoy en día la tecnología médica y la tecnología en general, va muy rápida y se vuelve obsoleto rápidamente, entonces si uno quiere, lo importante es que el medico de consulta, el medico diagnostica pero hoy en día el medico tiene una serie de ayudas diagnósticas, la tecnología, y no solamente la tecnología de tipo administrativa, la tecnología de laboratorios, de imágenes, la tecnología productiva y ahora podemos hablar una cuarta que se llama la medicina personalizada, cada individuo es un mundo, cada individuo merece un tratamiento único, es como la tendencia mundial, las tecnologías se van haciendo cada día más finas casi que a nivel de célula únicas, y eso lógicamente trae que el diagnostico sea más certero y por lo tanto el tratamiento va más seguro eso es lo que se espera que una tecnología médica haga. Cualquiera adelanto en tecnología tiene que ser más fino, que permita al médico darle un diagnóstico más certero para hacer entonces un tratamiento más efectivo. En los marcadores es donde yo siempre he dicho Colombia está atrasada, el sistema nuestro está muy atrasado, por ejemplo acá se compra mucho la parte de aparatos de resonancia magnética, la parte de los Tags, la tecnología computacional axial, en cuatro dimensiones, y eso es muy bueno pero rápidamente eso se va quedando atrás, ahora hablamos del test de tesla, resonancia magnética 20 tesla, pero ya están saliendo de 30, 40 tesla que quiere decir eso, que son energía mas penetrantes y te permiten ver mejor tu interior y al verte más detalladamente el interior te permite ver más detalladamente donde está la afección y por lo tanto puedes ir a hacer un tratamiento y un diagnóstico más rápido. En tecnología básicamente si me preguntan que tecnologías, las tecnologías diagnósticas, y las tecnólogas quirúrgicas también, que hoy en día ya no se abre no se hace heridas, no se hace incisiones, se hace una cirugías que pueden inclusive sin afectar la piel con láser asistido para hacer la cirugía, eso está siendo muy revolucionado ahora, por dos razones cuando a ti te hacen una herida el riesgo de infección es casi del 60% en los hospitales públicos, a ti te hacen una herida por una apendicetomía, o para cualquier caso, el momento en que se te cierra ya tienes un 20% de que se te infecte por contaminación, y entonces eso hace que el postoperatorio en este caso sea muy caro, muy riesgoso por un lado y caro por otro, porque esa persona va a padecer de una enfermedad de tiempo no sotomial hospitalaria que no tenía, entonces además la que llevo al hospital sale con una que no tenían entonces son costos de tratamiento en una persona, porque inclusive pueden llegar a morirse, hay casos donde esas personas llega a una septicemia porque no se les controlo la bacteria, son muy agresivas y un mes luego entran a septicemia y no hay formas de sacarlas, entonces ese es otro riesgo.  Y las tecnologías de atención social, porque mire yo siempre he insistido y él te dice que tenes, y yo le pregunto si yo supiera que tengo no estaría acá, entonces es lo que me pregunto el que atiende no es un número no es una máquina, esta con malgenio entonces es parte social humana es lo que de una manea debe resurgir en lo que llamamos las tecnologías y eso se hace por medio de unas asistencias. 


P30: 19. Transcripcion Felipe Garcia.docx - 30:9 [Grupos, educación, manejo del ..]  (42:42)   (Super)
Códigos:	[TECNOLOGIA] 
No memos

Grupos, educación, manejo del dolor por ejemplo, hoy en día hay dos formas de manejar el dolor, clínicamente cortar los nervios, o socialmente porque parte del dolor es un efecto psicosomático, entonces esos grupos sociales que manejan el dolor son muy importantes, entonces eso debería ser una teoría de tipo social, hacer que la persona que está en una sensación de dolor con ayuda muy bajas de medicamentos y fármacos puedan manejar ese dolor. Otro por ejemplo es las discapacidades, se podrían asociar las discapacidades y hacer que no sea solo las prótesis si no como usar las prótesis en que momento y para que usarlas que eso implica un trabajo social, la inclusión social es una tecnología importante no solo desde el punto de vista conceptual si no también de acción como tecnología. Entonces hoy en día los terapistas ocupacionales por ejemplo, están amarrados a eso a tecnologías sociales, hacer que esas disfunciones por manejo no farmacológico puedan convertirse pasar a totalmente en funciones, porque a veces la disfunción viene desde muy adentro de nuestro sistema nervioso más que todo de nuestro cerebro, entonces el manejo por ejercicio por hábitos hace que parte de estas disfunciones pueda recuperarse y eso se llama la tecnología social yo colocaría ese gran grupo de intervenciones yo lo llamaría tecnología social.

P30: 19. Transcripcion Felipe Garcia.docx - 30:10 [FG: Mire el problema para un b..]  (45:45)   (Super)
Códigos:	[TECNOLOGIA] 
No memos

FG: Mire el problema para un buen diagnóstico vuelvo y repito, son dos temas se llama sensibilidad y especifico, cualquier diagnostico debe ser especifico más del 95% ósea que solamente se tenga errores en un 5% y que sea especifico que cuando yo tomo la gótica de sangre y la pongo en un glucómetro y me mide mis niveles de glucemia ese glucómetro debe estar calibrado, y la persona debe saber usarlo. El diagnóstico más simple cuando tú tienes una hiperglicemia y te dicen mira el azúcar, los niveles de glucosa en sangre, ahora se hacen en portátiles inclusive aquí y comienzo yo y me pincho una gótica de sangre y peor ahora me trago una pildorita que me la incorporan acá y me están diciendo cuando me sube los niveles de azúcar en sangre de colesterol. Esas son las tecnologías que llaman en tiempo real, todos estos dispositivos, todo lo puede ver por allí, por el celular por todos los dispositivos, eso tiene ventajas y desventajas, primera ventaja que ha masificado mucho algunos diagnósticos que antes eran costosos y demandaban mucho tiempo. Yo me acuerdo cuando a usted le median la glicemia en semanas o días, era todo un procedimiento químico para determinar las reacciones enzimáticas los niveles de glucosa, hoy en día con un paso en 30 segundos un glucómetro te dice tiernas tanto de glucemia, eso es una base y eso ha hecho que ese tipo de metabolitos, que de una manera tensionan y pueden determinar el estado metabólico normal o patológico tenga mucho acceso, acceso al público el costo del glucómetro ha bajado antes costaba dos millones de pesos, <hora cuesta como cien mil, ósea que esa es otra cosa, a medida que se hace más consumible la tecnología va bajando. Eso es bueno pero también implica que la automedicación que es el otro problema se expanda y la automedicación tiene un gran problema y es que no se sabe cuál es el efecto de esa medicación ahí es donde interviene el médico y es el que tiene que saber cuáles son los efectos de esto y esto otro. Pero la tecnología antes no puede ser manejada de manera masiva, por ejemplo una persona semiótica que primero pues utilizan energía muy penetrantes, todavía no se ha podido tener una resonancia magnética portable entonces esas tecnologías primero necesitan espacio, muy específicos, anti vibratorios antiondas, y lógicamente son exigidos porque una resonancia sale en media hora, una hora, entonces hace que por día ese quipo tenga pocos usuarios para ingresar y para atender las necesidades del sistema,. Entonces las tecnologías en ese sentido tienen dos grandes, ahora viene las tecnología de lo que tú dices los marcadores que son las tecnologías genómicas, si tienes o no cáncer o tienes el riesgo para sufrir riesgo de cáncer de mama estas cosas, que de alguna manera se tiene que sacar el DNA entonces es caro una especie de muestra y te van a analizar x o y marcadores, entonces ese instrumento es caro y pienso que el sistema mismo de salud no lo podría… por ejemplo un screening de 600 marcadores de riesgo te cuesta 3 millones de pesos. 

P30: 19. Transcripcion Felipe Garcia.docx - 30:11 [FG: Pero porque en estados uni..]  (49:49)   (Super)
Códigos:	[TECNOLOGIA] 
No memos

FG: Pero porque en estados unidos hay consumo hay un consumo masivo, el problema es que cuando lo traes a Colombia debes tener importación costos de inmigración, el costo de ganancia entonces se sube, nosotros mismo decíamos se consiguen marcadores por 400 dólares da un millón algo, pero aquí no pagamos el local, la luz el agua, si lo transciendes a usarlo a un mercado se va a subir en un 100 % y aquí el post no lo está cubriendo entonces yo fui uno de los pioneros que en Colombia se hiciera la prueba de carga viral y la genotipicacion de VIH en Colombia, esa fue mi formación en esa época solo se cubría el ELISA, y el confirmatorio, y nosotros lo hacíamos, cuando empezaron a llegar las tecnologías de punta que era la carga viral que era cuantos virucitos tenía por decilitro de sangre para poder traer los tratamientos que se daban, eso llego aquí a Colombia en esa época costaba dos millones de pesos una prueba de eso, ahora esa prueba de carga viral te puede valer cien mil pesos, porque la hicimos popular, se masifico al químico había que darle si le estaba funcionando o no el fármaco en la prueba viral, entonces empezaba a subir y el problema del VIH es que no hay un fármaco que te controle 100% todo el tiempo y hubo otro problema, virus que no respondían a esos fármacos porque su genética variaba, entonces se hizo la genotipicacion, que era ver el genotipo de ese virus que no tienes, entonces un ejemplo la cual vas introduciendo tecnologías, que precisamente eran muy introducidas, a costo muy alto y la vas masificando, entonces se hizo a través de las leyes en los servicios del post, en este momento carga viral y genotipicacion son servicios del post para población en riesgo para prostitutas para homosexuales para quienes tienen que ver con el riesgo de contagio de enfermedades sexuales.  Pero eso se puede extrapolar a los que son cardiacos a los que son hipertensos y están llegando pero a costos muy altos, el screening de los infantes en estados unidos solo se saca sangre del talón y es un screening como de 2000 marcadores entonces ya se sabe qué riesgo tienen, ese examen en Colombia solo se hacen para problemas tiroideos y es obligatorio hacerlo, sangre talón y se hace la prueba de TCH para determinar si ese niñito va a tener problemas en tiroides o en retraso mental o problemas de aprendizaje, que es más o menos eso. Pero no se puede ir mas allá porque es más caro, entonces quien lo paga el que tiene 20 millones de pesos ese examen, entones el problema de las tecnologías es problema de los usuarios y de los costos y por eso estoy diciendo ese sistema se vuelve inequitativo en los estratos más bajos y no se puede acceder, yo lo puedo pagar porque tengo un nivel de ingreso entre comillas suficiente para pagarlo, puedo hacer el sacrificio, estrato 4 5 o 6, de ahí abajo no se puede hacer a duras penas tienen para comer. Por eso decía yo en términos tecnológicos Colombia tiene que tomar dos decisiones grandes, que le ofrece a la población de bajos ingresos eso si lo tiene que asumir el estado 100% porque no van a tener capacidad de pagar eso y obviamente las tecnologías son más predicativas pues el diagnostico se hace más seguro y el tratamiento más efectivo y baja costos, a mí me da un dolor de cabeza voy a la EPS y me dicen tómese una aspirina y resulta que a los dos días no se me quito el dolor de cabeza, eso es otro costo por otra atención, y el médico le dice cámbielo por ibuprofeno pero no se tiene el origen de ese dolor de cabeza, hay que hacerle entonces resonancia estudios cerebrales para ver si el problema está en la cabeza en los músculos o en el cráneo. Entonces la tecnología en ese caso es determinante, mire en el reino unido salió hace dos año un término que todo el mundo manejaba pero que ninguno gobierno había aceptado, y en 2009, 2010 el reino unido dice que él apoya a lo que llama la medicina genómica personalizada, eso trajo una revolución muy grande en el sistema de salud del reino unido porque ya el estado dice que hay que atenderlo a los pacientes de manera personalizada y esa es la tendencia en muchos países, nosotros estamos muy lejos porque desafortunadamente aquí todavía se manda aspirina para el dolor de cabeza y a los 3 días la persona tiene un shock aspirinico y se muere, o se desangra. Entonces no tenemos esa capacidad y nos va a costar mucho porque el sistema esta desequilibrado, por ejemplo los países tropicales tienen mucho problema de infecciones, la tecnología de enfermedades infecciosas para detectarlas y para tratarlas es bajo, la malaria en la costa pacífica se diagnostica por una gota gruesa pero no se cura, no se tiene cura en estos momentos y son pacientes que reinciden, y que día a día te tienes que ir cambiando de fármacos y es un proceso, esos costos ese paciente se vuelve crónico porque no se curó, el problema de los hipertensos, el hipertenso tarde que temprano tiene que involucrarse en un programa de crónicos que ofrecen las EPS, porque los tienen que controlar en el momento que un crónico hipertenso se le suba la presión arterial y tenga que irse a una clínica a que le midan la presión y mire cuanto le cuesta, pero si lo tengo controlado por una especia de programa, lo tiene controlado y le ahorra al sistema costos extras por atenciones episódicas. 

P30: 19. Transcripcion Felipe Garcia.docx - 30:12 [Yo siempre he dicho la vida es..]  (53:53)   (Super)
Códigos:	[TECNOLOGIA] 
No memos

Yo siempre he dicho la vida está por encima de todo y cuando usted ponen en manos de una tecnología la vida, quien es el humano o la tecnología, la tecnología es una ayuda importante pero el humano es el centro de un sistema de salud y no hablemos de salud, de bienestar humano, porque hoy en día las mismas naciones unidas hablan de bienestar que es la misma integralidad, el hombre, el  individuo dentro de un ecosistema que está recibiendo impulsos está recibiendo señales, que lo pueden enfermar o lo pueden dar una vida saludable, el bienestar es físico es emocional, es inclusive el entorno que es lo que yo digo, usted puede comerse unos frijoles y en ciertos estratos bajos esos frijoles tienen connotaciones de higiene porque el agua, por muchas cosas, en cambio usted va para arriba y ese frijol es el mero frijol prácticamente se vuelve un alimento, entonces abajo se vuelve un problema de salud arriba se vuelve en un alimento por el entorno. Este es un sistema metodológico básicamente, es un instituto metodológico que va a poner lo que llaman los estándares que es la otra cosa, a nosotros nos traen los estándares de estados unidos, los niveles de glicemia todo viene con referencia de sajones, siempre he insistido de sajón no tenemos ni siquiera la S, somos otro metabolismo otra fisiología, comemos diferente, entonces nuestro metabolismo es otra cosa y ves gente que tiene 400 de colesterol que deberían tener yéndose a una clínica y están tranquilos y normales, personas que manejan 150 a 200 niveles de glicemia, persona que manejan inclusive tensiones arteriales de 180, 120 y están normales peor para el sistema es un hipertenso, por eso hoy en día ese sistema de medicina personalizada cobra mucho sentid

P30: 19. Transcripcion Felipe Garcia.docx - 30:13 [Yo siempre he insistido nosotr..]  (57:57)   (Super)
Códigos:	[TECNOLOGIA] 
No memos

 Yo siempre he insistido nosotros tenemos un directo en regalías para cáncer de mama, porque en estos momentos predecir cáncer de mama 10 años o 20 antes puede ser mas tanto para la persona como para el sistema de salud, pero los marcadores vienen de allá arriba de los sajones y un estudio que se hizo es que nuestra muestra étnica es muy diferentes, la estructura de nuestros genes es más afro indígena, y los marcador no son los sajones un marcador es un pedacito de DNA que hace la diferencia entre una etnia y la otra y muchas veces esos pedacitos caen dentro de genes que están controlando la salud entonces ese cambiecito puede hacer resistente a una droga o no, entonces cuando se usa esa droga contra una enfermedad no responde o de pronto si me responde son los famosos SNP los marcadores los perinofijos de nucleótidos que esa es la exploración genética que se hace hoy en día, la individualización del genoma para una atención personalizada para ver qué tipo de tratamiento le doy yo a esta persona. 

P30: 19. Transcripcion Felipe Garcia.docx - 30:14 [e da un indicio de riesgo, no ..]  (61:61)   (Super)
Códigos:	[TECNOLOGIA] 
No memos

e da un indicio de riesgo, no le dice que va a tener pero ahora si vienen un manejo preventivo. 

P30: 19. Transcripcion Felipe Garcia.docx - 30:15 [Cuando su metabolismo estaba y..]  (65:65)   (Super)
Códigos:	[TECNOLOGIA] 
No memos

Cuando su metabolismo estaba ya tocado, pero lo puede predecir en un niño o una persona joven, le puede decir oiga usted no se va a morir de infarto pero tiene mucho riesgo, entonces haga una vida tranquila deporte, alimentación buena, eso es lo que hace las tecnologías sociales, y eso es lo más importante de las tecnologías médicas de hoy en día la predicción y la prevención del riesgo y la susceptibilidad, riesgo es poblacional susceptibilidad es individual, entonces cuando le hago escaneo del genoma suyo y tengo una serie de cambios que me dicen este cambio puede generar una predisposición a ser hipertenso y este otro hace como un conjunto yo le digo usted tiene marcadores que le incrementan su susceptibilidad a riesgo coronario, e hipertensión se lo estoy diciendo a los 18 años, no a los 50 eso ya es una ganancia y le baja costos al sistema, pero se llama educación usted tiene que educar la población que es el otro problema que tiene el sistema nuestro no educa, es un sistema de tratamiento ni siquiera de prevención, el tratamiento no educa o educa muy tarde ya cuando usted es hipertenso, y el sistema ya está con los costos. Por eso el instituto de tecnología tiene que primero, valorar las tecnologías existentes pero también marcadores reales nuestros y en eso estamos haciendo mucho énfasis en secuenciar nosotros hemos descubierto casi 80 exomas del pacifico. Exoma es la pate que codifica por las proteínas de un organismo ósea, la proteína son las que manejan toda la maquinaria funcional, una de ellas se llaman enzimas y manejan el metabolismo, el metabolismo es una manera de decirlo de cuando estas con diabetes, con hiperglicemia porque metabolismo es quien produce una serie de efectos normales o anormales, y se te van acumulando  y producen enfermedad, el Exoma es la cantidad de genoma que es funcional, o te mantiene vivo. Y eso viene porque nuestros genes tienen un pedacito que se llama un Exoma seguido de un intron, y otro Exoma, tiene intrones y exones pero el intron no amplifica por nada, es una cosa silenciosa el exón sí, es un pedacito de la proteína que esta codificada y va a ser traducida y procesada en un organismo y en una célula, entonces cuando usted secuencia todo esos exones que son la parte proteica se llama Exoma, que son más o menos 35 millones de pares de base que tiene el genoma completo, un genoma tiene 3.500 millones de pares de bases 


P30: 19. Transcripcion Felipe Garcia.docx - 30:16 [Uno solo, uno tiene 3.500 mill..]  (69:69)   (Super)
Códigos:	[TECNOLOGIA] 
No memos

Uno solo, uno tiene 3.500 millones de pares de bases en cada célula. 

P30: 19. Transcripcion Felipe Garcia.docx - 30:19 [y en estos momentos hablamos d..]  (89:89)   (Super)
Códigos:	[TECNOLOGIA] 
No memos

y en estos momentos hablamos de plataformas, porque la historia médica no tiene que ser física, entonces yo la tengo en tiempo real, es que esa es la tecnología administrativa, el Big Data no ha entrado al sistema se maneja muy local, nosotros por lo menos estamos manejando plataformas para que las EPS y las IPS pasen todos los datos y los pasen a datos virtuales, de manera que con un botoncito queda la historia en tiempo real de esa persona, y la puedes compartir con todo el sistema. 

P30: 19. Transcripcion Felipe Garcia.docx - 30:20 [No es un derecho, prácticament..]  (93:93)   (Super)
Códigos:	[TECNOLOGIA] 
No memos

 No es un derecho, prácticamente. El derecho a la salud es que yo tenga igualdad de acceso a todo lo que me haga saludable, y la ley lo dice, pone una parte así como de las naciones unidas pero ni siquiera define que es salud, la salud la define de manera muy estocástica o muy factual, yo siempre he dicho que el gran problema en este momento del médico es que viene el enfermo y no se sabe si está sano, no se sabe porque tú puedes estar muy sano en tu corazón pero tu pie esta malo, entones que es estar enfermo y que estar sano, más bien hay un equilibrio que se llama bienestar que es otra cosa, que tú seas funcional y que te haga la vida lo más amena posible sabiendo que estas enfermo, yo siempre lo he dicho en mis conferencias, nacemos enfermos, nuestro genoma está enfermo, para que expresemos esa enfermedad es diferente ahí viene la parte del manejo ambiental el seguimiento clínico importante, los hábitos  todo eso. Uno ya nace enfermo el sano quien es, yo no conozco a ninguna persona sana en términos clínicos manejamos niveles de intensidad de afecciones o de alteraciones de lo que llamamos la función humana, pero ninguna persona es sana, entonces viene el problema de que es enfermedad, y ese es el problema el derecho a la salud que es el derecho que me pongan a mi sano 100% no, que me manejen una especie de entorno, que permita ser funcionar y me permita tener una especie de bienestar integral para yo poder desempeñarme un rol social en mi sociedad. Y si tú miras las naciones unidas, derecho a la salud, derecho a la atención integral, derecho a la educación, al tratamiento derecho pero como, yo reclamo que yo tengo que estar sano si estoy sano estoy más enfermo, pero aguanto tal cosa y soy funcional y con eso vivo. El problema es que hoy en día cobran mucha importancia a lo que llaman rehabilitación humana y más allá de los fisioterapeutas, la terapia ocupacional porque mucho de lo que usted ve en el sistema es ocupacional, viene de hábitos de entornos que no son realmente aceptables para usted desempeñarse. 

P30: 19. Transcripcion Felipe Garcia.docx - 30:23 [FG: Entonces ese sistema tiene..]  (112:112)   (Super)
Códigos:	[modelo de salud futuro] [TECNOLOGIA] 
No memos

FG: Entonces ese sistema tiene el gran defecto de digamos esa administración del sistema esta corrupta, en ciertas partes es un sistema tan factual que esta al dominio de lo comercial es un sistema que no va a funcionar. Entonces yo pienso que el sistema integrado de salud no sé si pueda funcionar como tal a mí me da cosita hablar de ese tema integral en salud, porque si fuera integrado tendría que colocar las medicinas que llamamos no tradicionales vs las occidentales alopáticas que llamamos tradicionales. Yo no soy como tan simplista pero creo que las tendencias se tienen que ver, relacionada mente ambas subsisten ambas sociedades están, toda sociedad está enferma toda comunidad humana va a estar enferma, si uno mira las estadísticas de cáncer a los 60 va a tener cáncer de mama, pero que se debía que la tecnología puede detectar cáncer de mama hasta edad muy bajita, sale la famosa mamografía, entonces ya el cáncer de mama se vuelve muy frecuente, será que estaba así, será que siempre ha habido así grandote y por la tecnología diagnostica lo subimos, esa es la pregunta. O será que precisamente está yendo hacia allá, que los ambientes lo está tocando tanto. Tal vez en los canceres gástricos hay una explicación, por la dieta y ahí sí puedo creer que el cáncer gástrico haya aumentado, bien sea por la parte de enlatados. El de próstata por ejemplo, yo el otro día estaba hablando con un estudiante de doctorado que es urólogo, es que el cáncer de próstata tiene que aumentar, como así que factores hay para que haya cáncer de próstata aparte de la edad. 

P30: 19. Transcripcion Felipe Garcia.docx - 30:24 [En cierta parte si, entonces s..]  (116:116)   (Super)
Códigos:	[TECNOLOGIA] 
No memos

En cierta parte si, entonces se vuelven modas entonces el otro farmacéutico empieza a producir toda la información para crear fármacos y entonces viene el problema del comercio, se inunda el comercio por cáncer de próstata. 

P32: 21.Transcripcion Oscar Gutierrez.docx - 32:12 [OG: Uno la identificación de l..]  (44:44)   (Super)
Códigos:	[TECNOLOGIA] 
No memos

OG: Uno la identificación de los problemas más acrucciantes, en este caso las enfermedades crónicas no transmisibles, diabetes, respiratorias mentales y circulatorias, la idenficacion de esos enfermos o de las personas que eventualmente van a generar riesgos. No hay que aumentar tanto los médicos, hay que aumentar la capacidad del paciente de comunicarse con su médico o con su sistema médico. La medicina regenerativa va a llegar el momento en que los diabéticos, es el mejor ejemplo de la aplicación de la medicina regenerativa porque vamos a necesitar a por muy bien tratada que este la diabetes con insulina el daño continua por debajo, la única forma es establecer la función del páncreas endocrino, trasplantándolo pero no hay suficientes motociclistas que donen órganos, entonces vamos a tener que buscar la solución que nos da la tecnología de tomar órganos de animales y cultivarlos en ambientes humanos para poderlos trasplantar a humanos sin que sea rechazado, ese es un ejemplo en la aplicación de la medicina regenerativa. Los corazones, la enfermedad cardiovascular además de toda la prevención que se puede hacer modificando estilos de vida, aunque no lo crea es muy difícil, esta cuestión de coma poquita grasa coma saludable, y haga ejercicio no es de los últimos 10 años, desde que yo era chiquito venían esas propagandas, y en estos 40 años la población aumento la ingesta aumento la talla y disminuyo la actividad física a pesar de toda la propaganda, simplemente porque la revolución tecnológica le puso al alcance de las personas la posibilidad de no tener que moverse, entonces antes usted caminaba cuadras para coger el bus ahora usted sale y se monta en su moto o en su carro. La comida chatarra, mire la discusión que hay en Colombia sobre el impuesto a las gaseosas quien gano el round, las gaseosas porque van a dejar cantidad de gente sin trabajo lo que no ponen es el otro lado de la ecuación que es cuanta gente se va a enfermar por el exceso de ingesta de calorías indiscriminadamente. Las medidas preventivas que es disminuir las enfermedades crónicas como las cardiovasculares, donde entra la medicina regenerativa; a nosotros nos gusta como humanos soluciones efectivas no que le toque trabajar toda la vida para lograrlo, entonces yo como engordo, no hago ejercicio tomo alcohol, meto drogas y al final voy a que me pongan un corazón nuevo entonces la medicina regenerativa está trabajando por lo menos en lograr que la producción de corazones mecánicos pueda uno comprar en un almacén eso ya es una figura ficcional o la posibilidad de desarrollar un corazón que pueda ser trasplantado sea un hibrido biológico, mecánico o logar que el cuerpo con manipulación genética acepte un órgano animal mientras logramos por ingeniería entre 200 o 300 años producir un corazón desde el nuevo  a partir de células madres


P32: 21.Transcripcion Oscar Gutierrez.docx - 32:13 [OG: Yo espero que en 3 años te..]  (47:48)   (Super)
Códigos:	[TECNOLOGIA] 
No memos

OG: Yo espero que en 3 años tengamos un páncreas artificial, ya lo tenemos diseñado acá en la universidad del Valle pero no hacemos mucho escándalo. No va a ser una bomba de insulina como los que se producen hoy, nosotros tenemos una ventaja sobre los países desarrollados y es que logramos por primera vez haber quimerizado, si usted se enfermara y necesitara un órgano como no tiene hermano gemelo que es idéntico la mejor posibilidad es que le diera un órgano su papa o su mama, porque la mitad de la información es de uno de ellos dos, así que el rechazo podría ser más limitado, entonces quimerizacion es coger un órgano pero antes de trasplantarlo yo le saco células madres a usted, cultivo al órgano para que adquiera su identidad y después de lo pongo, y cuando usted lo recibe su sistema inmune lo reconozca y no le monte la respuesta inmune de rechazo, eso es lo que estamos trabajando en la universidad. De pura chiripa no sabía de qué me iba a hablar usted y me traje esto que lo había llevado para llevárselo a los hijos. (Muestra un video) 
Esta muchacha tenía cáncer de tráquea la solución era sacarle esa tráquea y necesitaba un trasplante el problema es que tenía 28 años y  ponerle un órgano significaría ponerle droga para evitar el rechazo, pero ella quería casarse con este muchacho y quería tener hijos y tomar esa droga le impedía tener hijos, ahora no hacerse el trasplante simplemente se iba a morir, aunque este sistema de quimerizacion que le resumí ahora no había sido probado en humanos era un plan que veníamos trabajando hace 10 años, empezamos a trabajar desde el 98, entonces un juez ordeno que aunque no se hayan hecho pruebas animales se le trasplantara la tráquea y con esa orden de juez la EPS tuvo que pagar y la IPS tuvo que hacerlo, entonces lo que veníamos trabajando hizo eso trajo la tráquea cuando apareció el motociclista yo la llame y le sacamos sangre, cultivamos la tráquea y luego se le puso, le fue muy bien y a los 4 meses se le quito toda la droga y eso fue hace 7 años. 


P32: 21.Transcripcion Oscar Gutierrez.docx - 32:14 [OG: La recupero momentáneament..]  (56:56)   (Super)
Códigos:	[TECNOLOGIA] 
No memos

OG: La recupero momentáneamente pero después la cicatrización le estrecho. Pero se quedó sin tumor y quedo viviendo. Esto es medicina regenerativa lo que se hizo con la tráquea lo podemos hacer con corazón y con corazón, y de hecho en eso estamos trabajando. Estos son ya la historia de lo que hemos hecho y este es el impacto de enfermedades cardiovasculares como diabetes, trauma, lo que le cuesta al país un trauma y este proceso de quimerizacion es trabajo y como usted ve le sestamos trabajando al animal, porque si nosotros podemos quimerizar de humano a humano podríamos estar más cerca de quimerizar de animal a humano, entonces ya habría otro brazo económico que sería criar animales para donar órganos modificados genéticamente que le sirva a la gente sin necesidad de usar mucha droga inmune y eso es lo que estamos haciendo aquí, lo que hicimos con la tráquea lo hicimos con corazón con pulmón, y próximamente vamos a trasplantar un hígado entre una oveja y un cerdo, quimerizándolo con células madre del cerdo si eso resulta estamos cerca. 

P32: 21.Transcripcion Oscar Gutierrez.docx - 32:15 [OG: Es el uso de las TIC, ento..]  (68:69)   (Super)
Códigos:	[TECNOLOGIA] 
No memos

OG: Es el uso de las TIC, entonces antes de llegar acá lo que tenemos que hacer es que el paciente que sus órganos  no se deterioren cuidándolo en casa, pero el sistema actual hace que el paciente tenga que hacer 5 puntos antes que lo atiendan y que lo vea el médico general que no resuelve nada, ir al médico especialista y volver son 5 o 6 puntos antes de empezar a solucionar el problema. Las naciones unidas vienen desde hace tiempo trabajando en el plan móvil y hacerlo con estos equipos, entonces usted puede producir un electrocardiograma, un electro cardiógrafo y ya hay yo conozco la tecnología, no lo tengo acá pero se lo podría mostrar en una próxima entrevista donde en la forma de esos parchecitos que se ponen tienen un par de electrodos y se los pone el sistema los puede pagar y esto se comunica con el médico y con sus institución y el medico lo ve y dice haga tal cosa.
Elm Health eso es de las naciones unidas. La incorporación de la tecnología en la atención básica de salud para los retos de la atención en salud, esta es una entonces esta era la forma antigua en la práctica médica, donde el medico iba a ver el paciente en el maletín veía el laboratorio, el medico iba hacia sus pruebas básicas la formula y se iba, un solo contacto. Pero con el aumento de la complejidad de la sociedad la gente se fue organizando en ciudades y pueblos y de todas maneras no lo veía en su casa o en su barrio entonces iba a un hospital, aumentaba la complejidad y ahora en ciudades como Sao paulo  el medico ya no va a la casa usted va a al médico y le manda exámenes que llegan a una fábrica de exámenes se demora 3 o 4 días, usted tiene que pedir cita al médico y empieza la solución. Esto es un laboratorio actualmente entonces esto es todo lo que se ha incorporado en tecnología y ahora lo que necesitamos es incorporar la tecnología a la casa, a solucionar antes de que el paciente viaje de aquí hacia los centros tecnológicos y esto como se puede hacer, con tecnología. Esta es la incidencia de la pobreza en las áreas urbanas y está en las salas rurales, con el aumento de la tecnología y con el aumento de la complejidad el medico ya no es resurtir el medico como lo estamos entrenando ahora, por ley una mujer embarazada debe tener por lo menos 1 o 3 ecografías durante el embarazo, pero en todo el país solo hay ecógrafo en 318 municipios, entonces el sistema está incumpliendo la ley entonces tenemos que buscar la forma no que el paciente se desplace si no ir vía portátil creando medios portátiles a ir a buscar el paciente para evitar que el paciente llegue complicado, esto se hacía antiguamente solo para unas enfermedades como la tuberculosis y habían carros por todo el país tomando fotografía a los niños . Eso mismo vamos a tener que hacerlo para llevar salud para que la gente no venga enferma y si se enferma poder cogerla en sus estadios tempranos y solucionar el problema, yo voy a buscar el problema de todas las EPS, un gentío en urgencias porque urgencias se convirtió en sitio de solución, si usted quiere que le solucionen el problema espere al sábado por la noche y amanece en urgencias. La tecnología de las telecomunicaciones esto ya se está aplicando en varios países, mire el problema con lo que tenemos en Colombia y es que los exámenes que son necesarios hoy en día porque los médicos no tienen tiempo para revisarlo de cabeza a pies, la mayoría de los exámenes de laboratorio se ordenan en urgencias, o en consulta médica general o especializada, en la general que se debería solucionar el 80% de los problemas estos son datos de ASEMI. Y estos son los exámenes solicitados y yo tengo eso cuales son la mayoría de exámenes y la gente tiene que ir a hacer eso y tienen que ir a que se los tomen. Entonces el uso por médico general en exámenes, el solo ordena al 18% de los pacientes, en cambio en el servicio de urgencias 	de cada 100 personas que consultan solo 28 son urgencias, y mire como la consulta especializada es la que ordena la mayoría de exámenes que son determinantes para el tratamiento, entonces lo que hay que hacer es darle la capacidad resolutiva al médico general, dotándolo de herramientas, de entrenamiento es que un muchacho sale ahora y no vuelve a tocar un libreo a menos que haga una especialidad que es el sueño de todos para poder solucionar su problema de vida, es que se desvirtuó el papel del médico, 

______________________________________________________________________

Código: Valores : Poder politico de los actores {23-0}

P 8: 1. Transcripcion Martha Lucia  ospina.docx - 8:21 [Pues en orden, creo que el may..]  (47:47)   (Super)
Códigos:	[Valores : Poder politico de los actores] 
No memos

Pues en orden, creo que el mayor poder lo tiene la industria, es que ahí tiene todas las IPS juntas y no son toda la bolsa de IPS, un cierto grupo de EPS es el segundo privadas grandes, en tercer lugar el propio congreso, por eso de hecho estas acuden al congreso es su interlocutor. 


P 8: 1. Transcripcion Martha Lucia  ospina.docx - 8:22 [Por eso en primer lugar el pod..]  (51:51)   (Super)
Códigos:	[Valores : Poder politico de los actores] 
No memos

Por eso en primer lugar el poder lo tiene el congreso, seguido de la industria, en tercer un grupo de IPS de ciertas características, seguido de ministerio, seguido de las asociaciones de pacientes algunas de ellas en último lugar está definitivamente las EPS y los trabajadores del sector salud. Aunque obviamente estos se encargan de hacer creer que son las EPS, ellas son tal cual como el verdugo de Maquiavelo en el príncipe, el príncipe tiene el verdugo que cobra impuestos que pone la cara, y cuando el pueblo se queja el príncipe lo manda al balcón y lo manda a decapitar, la EPS es el verdugo y estos otros hacen toda la tarea para que sigan siendo las señaladas

P 9: 2. Transcripcion Alex Duran_ok.docx - 9:2 [Viene la siguiente pregunta, e..]  (54:56)   (Super)
Códigos:	[Valores : Poder politico de los actores] 
No memos

Viene la siguiente pregunta, en el país hay varios actores en el sistema de salud las EPS, la asociación colombiana de hospitales, la asociación de profesionales, los usuarios, cuando se tramitan reformas todos van al gobierno al ministerio donde se discuten las normas, dentro de estos gremios ¿Cuál  cree usted que es  el  de mayor poder político? 

AD: Las EPS, es mas ellos invierten en política, eso en estos momentos no son la sociedad civil son las EPS quienes tiene poder político en este país, por eso no le pasa nada a cafesalud, por ejemplo yo ahora a pesar de que defiendo el modelo de aseguramiento y de la administración de un tercero, también soy duro criticando su figura y pues en estos días yo decía cuando salga de la secretaria en ninguna EPS me van a dejar trabajar porque dirán que este es el que dice que nosotros no hacemos por la gente, que nos prima lo financiero, entonces ahí no voy a estar pero a mí eso me vale poco. 

P10: 3. Transcripcion-Alexandra_Matallana_ok.docx - 10:1 [¿Dentro del sistema de salud c..]  (160:162)   (Super)
Códigos:	[Valores : Poder politico de los actores] 
No memos

 ¿Dentro del sistema de salud cuál cree que es el actor que tiene mayor poder político?

AM: las EPS, tienen la plata, el sector farmacéutico, ¿que EPS  me preguntas tú?

P10: 3. Transcripcion-Alexandra_Matallana_ok.docx - 10:2 [omo actores las EPS, la indust..]  (166:166)   (Super)
Códigos:	[Valores : Poder politico de los actores] 
No memos

omo actores las EPS, la industria farmacéutica mueve mucho políticamente, mueve mucho porque tiene dinero y tiene el poder, el que tiene el dinero tiene el poder, ese me parece un actor que tiene bastante pode

P11: 4. Transcripcion Armando Gonzales_ok.docx - 11:2 [Hay una cosa que yo le quiero ..]  (59:61)   (Super)
Códigos:	[Valores : Poder politico de los actores] 
No memos

Hay una cosa que yo le quiero preguntar, hay varios actores del sistema de salud EPS, IPS, gobierno, usuario, vigilancia, superintendencia. ¿Cuál considera usted que es el actor que tiene mayor poder político dentro del sistema de seguridad social en salud, entre EPS, IPS, gobierno, entidades territoriales, departamento y municipio? 

AG: El gobierno es quien debe dar las políticas la filosofía, las financiación que es lo más importante cuando se requiere que la del estado para poder hacer esa influencia, sobre todo en el sistema de salud. Es que el sistema de salud es un sistema totalmente desequilibrado desde cualquier punto de vista, desde que el sistema de salud no tenga la financiación adecuada ya hay desequilibrio.

P12: 5.Transcripcion Carlos Fajardo_ok.docx - 12:2 [Los sistemas de salud son dado..]  (84:90)   (Super)
Códigos:	[Valores : Poder politico de los actores] 
No memos

Los sistemas de salud son dados por la política, por el sistema político del país, entonces el sistema de salud esta permeado por la política. ¿Cuál de los actores (gobierno, EPS, IPS, usuarios, laboratorios), cuál de los actores cree usted tiene más poder político dentro del sistema general de seguridad social en salud? 

CF: En lo que se ha visto en estos años yo diría que inicialmente, habría una EPS demasiado grande en Colombia que es la saludcoop y que tenía mucha inferencia en la parte política como tal a través de la liquidación, de la intervención que el gobierno le hizo desapareció eso, pero también existen IPS, que están permeados y que son representados por políticos, que son una forma y un foco de desarrollo de la parte política, el ejemplo más claro es el HUV. 

PC: Yo diría no la política dentro de la institución, si no el poder político digamos en el congreso para cambiar por ejemplo leyes, toda esa cosa. 

CF: Creo que hoy quien más ha incidido en los cambios de leyes y eso son las comunidades científicas, porque cuando se plantea la ley estatutaria, la reforma estatutaria fue mucha porción de las comunidades científicas junto a los médicos y junto a las IPS de tipo estatal, porque hoy las EPS somos vistas como el patito feo. Pero uno dice las normas como me favorecen para verdaderamente esas políticas ayuden y cada vez nos meten más cosas nos obligan a más cosas, la UPC creció un poquito más de la inflación por decir algo, que eso es parte de lo que se maneja en la política como tal, yo diría que de parte de las EPS no es fuerte en ese 

P13: 6 y 7.Transcripcion Diego Gomes y Gonzalo Gonzales_ok.docx - 13:2 [Entre los actores del sistema ..]  (75:79)   (Super)
Códigos:	[Valores : Poder politico de los actores] 
No memos

Entre los actores del sistema ¿cuál cree usted que tiene mayor poder político? 

GG: Yo creo que un actor que no está en el sector, y son los políticos mismos que están en otras áreas y que han sacado dinero del sistema.

DG: Sin duda, los senadores y congresistas, el mayor actor que está influenciando para mal todo el sistema de salud son los intereses corruptos de un grupo de políticos.

P15: 9. Transcripcion Julian Duran_ok.docx - 15:2 [El sistema de salud tiene vari..]  (88:90)   (Super)
Códigos:	[Valores : Poder politico de los actores] 
No memos

El sistema de salud tiene varios actores, los prestadores de servicios las EPS, las farmacéuticas principalmente esos tres, el gobierno sería un cuarto actor, los usuarios un quinto, ¿cuál de los actores que hay usted considera tiene un mayor poder político? 

JD: pues el mayor poder político lo tienen las EPS, porque ellas son las que en ultimas están decidiendo y controlando esa cadena de valor de la salud en Colombia , las EPS pueden controlar las IPS, porque les subcontratan los servicios con ella, también pueden controlar las farmacéuticas porque son las EPS las que compran los productos, entonces en ese sentido tienen mucho poder y también son quienes ofrecen el servicio a los usuarios y en Colombia los usuarios en ese sentido a pesar de que el sistema de justicia los ha protegido mucho con las tutelas, las EPS a veces se empecinan en no cumplir esos fallos judiciales entonces allí se difumina un poco el poder de los usuarios. 

P15: 9. Transcripcion Julian Duran_ok.docx - 15:16 [ues el poder político lo tiene..]  (94:94)   (Super)
Códigos:	[Valores : Poder politico de los actores] 
No memos

ues el poder político lo tienen los políticos más allá si es el congreso o si son los partidos el poder político son de los políticos.

P16: 10.Transcripcion Angela Tascon.docx - 16:2 [Dentro de los actores del sist..]  (40:42)   (Super)
Códigos:	[Valores : Poder politico de los actores] 
No memos

Dentro de los actores del sistema hay unos que tienen más poder políticos que otros, usted cual considera que es el actor que tiene más poder político dentro del sistema, me refiero a que puede incidir sobre quien hace las leyes todo el estado. 

AT: Yo diría que las EPS son quienes tienen más poder porque de hecho las EPS manejan el tema del capital entonces son como externas yo considero que las IPS somos internas no tenemos ni siquiera el poder de decisión para tarifas, entonces yo diría que unos de los actores fundamentales y que manejan el poder y demás son las EPS porque tienen mucho respaldo político, las EPS tienen mucho respaldo político y las aseguradoras, las de ARL y demás también.

P18: 11. Transcripcion Fabian Mendez.docx - 18:3 [¿Cuál de los actores del siste..]  (57:63)   (Super)
Códigos:	[Valores : Poder politico de los actores] 
No memos

 ¿Cuál de los actores del sistema general de seguridad social actual cree usted que tiene el mayor poder político? 

FM: Pues las grandes corporaciones que están detrás del sistema, tienen mucho poder político, yo no creo que christus health venga a Colombia a mejorar la salud de la población colombiana, entonces yo creo que detrás de eso hay un interés muy grande de corporaciones con mucho poder económico.

PC: ¿Entonces para usted esas corporaciones podría ejemplificarlas dentro de los actores colombianos como las EPS? 

FM: Están las EPS pero también está la industria farmacéutica, yo creo que las dos tienen mucho poder, vea lo que paso acá con Comfenalco, en estos días en dinero yo leía que lo que le molesto a  British no fue ni siquiera el precio del medicamento, si no el tonito que uso el ministro una compañía farmacéutica internacional puede molestarle el tono de un ministro de un país, estamos ya en una situación en que tienen tanto poder que ellos pueden decir estamos violando principios de libre mercado, no me hable usted con este tono, como así es el ministro de salud de un país, que basado en el derecho a la salud de la gente pues dice yo no puedo mantener el sistema con este precio, acépteme este otro precio, usted es monopolio pero resulta de que por medio esta la salud de un país, ni siquiera un precio promedio, es el precio en el mundo.

P19: 12. Transcripcion Fabio Osorio.docx - 19:2 [Mira yo he señalado 7 actores ..]  (56:58)   (Super)
Códigos:	[Valores : Poder politico de los actores] 
No memos

 Mira yo he señalado 7 actores del sistema de salud y nosotros siempre hemos hablado del poder político de los actores del sistema de salud, ponle 7 al actor que consideres le mayor poder político, 6 al que le sigue y así. 

FO: Yo creo que acá en Colombia para mí el congreso son los que tienen más poder político tienen, ellos pueden dirigir al sistema de salud yo creo que el congreso, el ministerio de salud con poder político, las farmacéuticas, las EPS porque ellos compran los medicamentos, para mí los pacientes son los que tienen menos poder político tienen, los trabajadores de la salud menos, las IPS como son manejadas cada vez que cambian de gobernador cambian de director de hospitales y clínicas para poder cumplir sus negocios, las EPS tienen su poder político, para mí el congreso y yo le daría un 6 al ministerio y a las EPS. Entonces yo creo que para no hacerlo así para manejar tú orden corregiría el 4 y el 5, las EPS le alcahuetean el congreso y el ministerio le alcahuetean a las EPS, estos manes las crean y estos las realizan entonces. El orden mío fue, congreso, ministerio de salud, EPS, la industria farmacéutica, las IPS, los trabajadores de la salud y lo ultimo los pacientes. 

P22: 13. Transcripcion Maria Ines Pantoja.docx - 22:2 [Cuál de los actores del sistem..]  (70:72)   (Super)
Códigos:	[Valores : Poder politico de los actores] 
No memos

 Cuál de los actores del sistema general de seguridad social en salud, tiene el mayor poder político.

MP: las EPS sin duda, no lo dudó ni un minuto, porque dentro del congreso de la republica hay mucha gente que tiene intereses en las EPS sea por lo que sea, y por eso no se ha podido cambiar, si tuviéramos un congreso transparente que piense en la gente, las cosas serían diferentes. Están en mora yo les dije una vez, la ley estatutaria dice que la violación del derecho a la salud debe tener sanciones, y las sanciones debe hacerlo el congreso de la república y no lo han hecho, nosotros le vamos a hacer una petición de porque no han cumplido el artículo de la ley estatutaria, ya en febrero  cumplen los dos años. 

P23: 14. Transcripcion Mario Hernandez.docx - 23:3 [¿Cuál cree usted que es el act..]  (56:58)   (Super)
Códigos:	[Valores : Poder politico de los actores] 
No memos

 ¿Cuál cree usted que es el actor dominante políticamente con mayor poder político en el sistema general de salud? 

MH: Las EPS, sin duda. Hay que entender que hay una relación ahí muy compleja porque desde el primer momento, e incluso si usted encuentra la discusión que se dio en el momento de la conformación de la ley 100, la discusión de los proyectos de ley que había en ese momento, encontrara que Juan Luis Londoño no quería meterse con el tema de las aseguradoras privadas, quera ver más bien como hacía para poder convencerlas de que se metieran ellas en seguridad social,  y por eso se ofreció ese mecanismo de pago de la UPC a los que estaban ahí que son los ACEMI, que tenía seguros privados en medicina prepagada, y se les dijo mire venga la única forma de mejorar el aseguramiento y convertirlo en universal, es que ustedes lo administren pero no puede ser con venta libre de pólizas, porque eso sería como en Estados Unidos, la propuesta es que mire le damos una plata anual una póliza anual a cambio de un POS y por eso entonces hay la confusión tan terrible que existe, que los recursos son públicos porque son fiscales y parafiscales de la seguridad social, se le entrega esta gente e incluso en la ley 100 dice, ellos solamente podrían tomar como gastos empresariales el 10% y el 90% debería ser una cuenta de orden para pagar las facturas de los prestadores y solamente para eso. Pues no, no lo han hecho nunca. Asumen que la UPC es su póliza comercial y la usan como comercial y los gobiernos no han hecho nada distinto, luego se volvieron aliados los gobiernos son aliados de ese agente dominante, los gobiernos dependen de esos agentes dominantes porque destruyeron las instituciones públicas de seguridad social y era parte del plan , de abrir los mercados abrir la inversión privada en todos los sectores ,pensiones, salud, riesgos laborales, los elementos centrales de las seguridad social al mercado, entonces claro que ahí no es que haya un actor innato, es que hay una lógica de convivencia política pública con decisión de gobierno y unos agentes que son los dominantes, porque son los que administran los recursos públicos para su propio beneficio.

P26: 16. Transcripcion Senador Ospina.docx - 26:2 [Cuénteme una cosa, el sistema ..]  (29:31)   (Super)
Códigos:	[Valores : Poder politico de los actores] 
No memos

Cuénteme una cosa, el sistema de salud tiene unos actores, EPS, ciudadanos, IPS, laboratorios de medicamentos, toda esta cosa. ¿Usted cual considera de los actores de salud que tiene el mayor poder político?

SO: Las EPS, porque tienen una gran capacidad de lobby  tienen  mucha población, porque de todas maneras han logrado constituirse en una nueva especie de institucionalidad y porque son muchos menos. Los otros actores como los prestadores del servicio son muchos no tienen ese nivel de organización y no tienen esa capacidad económica para poder influir mediáticamente o políticamente en las posicione

P28: 17. Transcripcion-Gustavo-Morales (1).docx - 28:3 [PC: pasemos ahora si a una pre..]  (63:66)   (Super)
Códigos:	[Valores : Poder politico de los actores] 
No memos


PC: pasemos ahora si a una pregunta, nombrarle a usted los actores de salud me paree que usted los conoce, los de siempre actores son gobierno, ministerio, la secretaria de salud, municipal departamental, las EPS, las IPS todo eso hasta el mismo congreso. De todos estos actores cuál cree usted que tiene mayor poder político para influenciar en las medidas que se toman del país.

GM: Los médicos, la ley estatutaria demostró que son el verdadero poder del sistema de salud. Es un poco sorpresiva la respuesta. 

P29: 18. Transcripcion Carlos Hernandez.docx - 29:13 [Indiscutiblemente la industria..]  (61:61)   (Super)
Códigos:	[Valores : Poder politico de los actores] 
No memos

 Indiscutiblemente la industria farmacéutica,

P30: 19. Transcripcion Felipe Garcia.docx - 30:7 [considero que el mayor poder p..]  (34:34)   (Super)
Códigos:	[Valores : Poder politico de los actores] 
No memos

considero que el mayor poder político está en manos de la industria farmacéutica 


P30: 19. Transcripcion Felipe Garcia.docx - 30:25 [Las farmacéuticas, seguro que ..]  (124:124)   (Super)
Códigos:	[Valores : Poder politico de los actores] 
No memos

Las farmacéuticas, seguro que si sobre INVIMA sobre el ministerio de salud, sobre los tomadores de salud, sobre la misma base que diagnostica y receta, es que la EPS es un instrumento de acción para mí, de quien de los que están ahí de la farmacéutica que es la que va a decir recete esto, de el medico que va a decir ya estoy acá, del mismo sistema de asistencia hospitalaria, entonces claro las EPS han creado sus propias clínicas y me parece muy bueno que las IPS creen sus clínicas porque sale saludcoop, era una EPS y puso sistema de práctica, es el flujo que le da más ganancia y le da menos perdida, no tengo que pagar por intermediación ya la tengo. 


P30: 19. Transcripcion Felipe Garcia.docx - 30:27 [FG: Plata para sacar recursos ..]  (135:135)   (Super)
Códigos:	[Valores : Poder politico de los actores] 
No memos

FG: Plata para sacar recursos del fosiga que le debía, ahora es ese esperpento que te da una especie de outsourcing que te distribuye las enfermedades catastróficas y te devuelve plata, ese es el loving político que hacia las EPS en el momento que había fosiga, porque fosiga era un fondo grandísimo ineficiente, que retenía las platas y los costos de enfermedades y ahora la ganancia la da la EPS, porque el estado le va a devolver la plata que yo le invertí allí era una ganancia redonda la EPS no perdía, pero cuando se le demora mucho la plata ahí si pierde, entonces ese era el problema de la presión política de la EPS sobre el fosiga. Y eso es en ese famoso cambio de sistema.

P31: 20.Transcripcion Hellen Mendoza.docx - 31:23 [HM: indiscutiblemente las EPS]  (122:122)   (Super)
Códigos:	[Valores : Poder politico de los actores] 
No memos

HM: indiscutiblemente las EPS 

P32: 21.Transcripcion Oscar Gutierrez.docx - 32:17 [OG. considero que el actor de ..]  (74:74)   (Super)
Códigos:	[Valores : Poder politico de los actores] 
No memos

OG. considero que el actor de mayor poder político en el sistema de salud en colombia son las EPS 

______________________________________________________________________

Código: Valores : Valores que determinana el nivel de humanidad del SGSSS {32-0}

P 8: 1. Transcripcion Martha Lucia  ospina.docx - 8:20 [n el sistema para mí la solida..]  (31:31)   (Super)
Códigos:	[Valores : Valores que determinana el nivel de humanidad del SGSSS] 
No memos

n el sistema para mí la solidaridad porque así funciona el sistema y la solidaridad por principio sigue existiendo porque todos aportamos a la bolsa, la definición es que el pequeño aporte de muchos permite el gran consumo de pocos, sin embargo se ha perdido muchísimo porque en general se tiene una expectativa tan enorme del sistema, que no es un pensamiento solidario, no es un pensamiento de si yo estoy aportando pero no uso el sistema pero es para que otros usen, no es le voy a sacar hasta el último peso que le he aportado a este sistema y ama que me den lo mejor en casa porque le he aportado al sistema y no es un pensamiento solidario y no es solo en eso, es una enfermedad del colombiano tenemos un egoísmo muy bravo. El segundo que sería para mí la igualdad pero la quiero transformar, igualdad en derecho pero lo que yo prefieren es equidad en el gasto, mayor inversión en quien más lo necesita y menor inversión o ninguna en quien no, y es algo que nos cuesta mucho lo que hablábamos el país más desigual si uno se pondría a ver en temas de salud, habrán otros peores pero nosotros tenemos muchas diferencias por zonas rurales dispersas enormes diferencias por ingreso, todo geográfico pero también por ingreso y volvamos al indicador de diarrea que te decía al principio, es un indicador del que uno se siente feliz pero cuando uno lo ajusta por tipo de vivienda como un indicador proxy de pobreza por el tipo de construcción de la vivienda el 70% de las muertes por diarrea en menores de 5 años las pone la población más pobre, entonces claramente Colombia es un país que ha mejorado los indicadores de salud a expensas de los ricos no de los pobres por eso me gusta la igualdad pero entendido como un ejercicio de equidad por eso estamos como estamos. El tercero es el respeto, y el respeto claramente cuando la ley estatutaria y te acuerdas cuando hubo un movimiento fuertísimo para que hubiera una reforma porque este es un país de vocación reformista, acá piensa que s reforma y todo queda arreglado y no caen en cuenta que la reforma es volver a empezar, y volver a empezar para reparar sobre lo construido. En ese tiempo tuve la oportunidad de ver a todos los actores y todos decían no, lo que ningún periodista no se atrevió a decir o no sabían es que el no de cada actor era distinto, el no de uno era no quiero porque quiero que este se vaya, el no de otro es no quiero porque me quieren eliminar a este, no quiero porque el fulano gana, todos los no eran en contra de los demás todo el mundo quiere que el otro desaparezca no hay confianza ni respeto de ninguna índole, porque priman los derechos individuales. Los únicos que yo veo sin ser perfectos de todos los que quieren estar en el sistema y sobrevivir las IPS pequeñas curiosamente, uno ve las IPS que hace su tareas sus tema y dicen si yo hago esto y trabajo en la secretaria y quiero esto, los únicos estoy generalizando pero es una actitud base de resto. Y dentro de las EPS hay de diferentes tipos, hay un grupo de EPS sin duda, unas 5 EPS que tienen una vocación aseguradora y querían hacer gestión de riesgo y la tenían desde hace tiempo y así piensan pero son 5 y el resto hay otras que han ido aprendiendo sobre la marcha, otras que no saben en qué negocio están, entonces el respeto es un valor fundamental. El que debería seguir es el dialogo por supuesto y el ultimo que no es menos importante la libertad, pero la libertad en este contexto que debería ser una libertad con una reglas claras y con vigilancia creo que en este caso tenemos exceso de libertad para algunos. 


P 9: 2. Transcripcion Alex Duran_ok.docx - 9:1 [Vamos a entra en los valores, ..]  (50:52)   (Super)
Códigos:	[Valores : Valores que determinana el nivel de humanidad del SGSSS] 
No memos

Vamos a entra en los valores, nosotros tomamos a Adela cortina y dice que cualquiera actividad para ser humana debe tener 5 valores, y esos 5 valores para ella son Libertad, igualdad, solidaridad, respeto, y el dialogo. La primera pregunta en orden de importancia de 1 a 5, ¿cómo cree que un sistema de salud debe ordenar estos valores?

AD: El primero indudablemente yo pienso que la igualdad, el segundo el respeto, el tercero la libertad, el cuarto el dialogo y el quinto la solidaridad. 


P10: 3. Transcripcion-Alexandra_Matallana_ok.docx - 10:3 [vamos a entrar a una tercera p..]  (141:143)   (Super)
Códigos:	[Valores : Valores que determinana el nivel de humanidad del SGSSS] 
No memos

vamos a entrar a una tercera parte que son los valores, nosotros hemos seguido el enfoque de Adela cortina, que dice que toda actividad para ser humana necesita de 5 valores: libertad, igualdad, solidaridad, respeto y dialogo. Esos 5 valores uno debe ser más importante que otros para el desarrollo, ¿usted en qué orden de importancia los ubicaría en el Sistema General de Seguridad Social de salud en Colombia?

AM: la igualdad de primero, segundo: respeto, Tercero: solidaridad, Cuarto: libertad y Quinto: dialogo. Todas son muy importantes.


P11: 4. Transcripcion Armando Gonzales_ok.docx - 11:1 [Uno de las cosas que hemos mir..]  (43:45)   (Super)
Códigos:	[Valores : Valores que determinana el nivel de humanidad del SGSSS] 
No memos

Uno de las cosas que hemos mirado nosotros es el riesgo moral en el sistema, también hace parte la negación de los servicios, entonces hemos consultado Adela cortina y nos dice que hay 5 valores para que un servicio sea humano, queremos trabajar más hacia la humanización del servicio, que son libertad, igualdad, solidaridad, respeto y dialogo, yo le paso acá para que usted los tenga a mano, ¿qué importancia se les da a estos valores, señáleme el primero que usted considere el más importante, en el actual sistema?

AG: Yo creo que el primero es respeto, es el valor más importante y pienso que todo está definido hacia eso, aquí una de los valores que tenemos es el respeto profundo que tenemos para todas las personas que se traduce en una vocación de servicio que es lo que nosotros defendemos todo el tiempo, nosotros todo el tiempo trabajamos por lograr que la gente se sienta bien atendida, se sienta respetada, se sienta que cumplimos con los compromisos que hemos adquirido. Solidaridad no sé cómo se miraría desde el punto de vista del sistema de seguridad de salud que tenemos, solidaridad me imagino que es esa gran intercomunicación que debe haber entre todas las clínicas y hospitales que conforman la red y estar atentos a recibir los pacientes, y estar siendo solidarios, ahí no tanto con la otra clínica, sino con el paciente mismo que nosotros tenemos que estar solidarios.  Y en eso es algo que nosotros nos destacamos muchísimo. Igualdad te podría decir yo interpretándolo desde el punto de vista, nosotros acá no establecemos ningún tipo de segregación por ningún paciente, no lo tenemos y eso yo creo que suena a frases de cajón. Libertad es la libertad que tiene todo el mundo pues en el caso de nosotros de salud escoger la clínica donde quiere ser tratado, ese sería como de los más importantes que todo el sistema de salud que es la libertad de escogencia, eso antes era donde le señalaban a uno que tenía que ir, y eso vino con la ley 100 la libertad de escogencia del profesional o la clínica. El dialogo como que es inherente a todos estos valores, dialogar con todos los públicos de interés, pero siempre con un gran objetivo de lograr la satisfacción de los pacientes. 

P12: 5.Transcripcion Carlos Fajardo_ok.docx - 12:18 [Los 5 no, yo creo que hay algu..]  (74:74)   (Super)
Códigos:	[Valores : Valores que determinana el nivel de humanidad del SGSSS] 
No memos

Los 5 no, yo creo que hay algunos que tienen ciertas restricciones en el sistema, por ejemplo la libertad, en este hay restricciones nadie puede decir yo me puedo ir a atender con x entidad, hay restricciones porque ahí le dicen no usted tiene que hacerse atender con la red que tiene la EPS y la misma  EPS dice no, si yo tengo esta red pero yo lo atiendo en determinados sitios, hay una restricción en un valor fundamental la libertad. La igualdad podría decirse que hoy si existe hoy, porque allá podrá encontrar gente del régimen contributivo y del subsidiado sin distingo de que sistema sea. En solidaridad se dio mucho cuando el contributivo le aportaba al subsidiado, ese principio a través de que todos ponemos para un mismo sistema, se está dando para ese mismo lado. Respeto el sistema carece mucho de eso, bajo esa concepción del respeto como tal si hay mucha falencia en ese aspecto porque no se prima a veces la urgencia, no se prima la persona como tal si no, prima más la parte financiera, prima más a veces el papeleo el trámite administrativo antes que la persona como tal para que pueda ser realmente diagnosticada y formulada oportunamente que su situación de salud mejore de la forma más rápida. Creo que hay una gran diferencia en lo que corresponde a la parte del subsidiado yo hablo por la empresa Emssanar, porque mucha gente hoy no se quiere ir del subsidiado porque dice que tiene mejor oportunidad que el contributivo. Y también pasara en el subsidiado casos de a veces un niño con cáncer por ejemplo y es tanto la tramitología que a veces el niño algo quiere o necesita urgente y que debería tenerse en cuenta por respeto a la persona, fue tratar la traumatología. Nosotros no estamos excento de que eso nos pase, tenemos un millón ochocientos afiliados. 


P12: 5.Transcripcion Carlos Fajardo_ok.docx - 12:1 [Si nosotros fuéramos a practic..]  (80:82)   (Super)
Códigos:	[Valores : Valores que determinana el nivel de humanidad del SGSSS] 
No memos

Si nosotros fuéramos a practicar esos valores que orden le daría usted? 

CF: Yo el orden que le daría seria que lo más importante es la persona, el respeto como tal, segundo la igualdad, el tercero la libertad, la solidaridad y el dialogo. 


P13: 6 y 7.Transcripcion Diego Gomes y Gonzalo Gonzales_ok.docx - 13:1 [PC: El modelo teórico que util..]  (61:65)   (Super)
Códigos:	[Valores : Valores que determinana el nivel de humanidad del SGSSS] 
No memos

PC: El modelo teórico que utilizamos fue el de Previsión Humana y Social, la PHS toca mucho la parte de valores, consultamos a Adela cortina y ella habla que para que cualquier actividad sea humana necesita de 5 valores, libertad, igualdad, solidaridad, respeto y dialogo. Yo quisiera doctor que usted me digiera en el sistema de salud en qué orden de importancia deben ir estos valores siendo el primero el más importante y así sucesivamente. 

GG: Yo pienso que el primero es igualdad, la igualdad permite la libertad, la libertad supone el respeto y el dialogo, y todo esto debe ser apoyado por la solidaridad entre todos eso es una democracia en última instancia.

DG: Yo lo pongo desde el individuo, pero lo primero para mi es cumplir con mi deber de preservar mi salud y conservar mi salud, no lo tienes aquí. El segundo fundamental es el respeto por todos los demás cuando yo estoy en una colectividad, de lo que yo abuse es a costa de otros, la niña Abudara debío ser consiente que 5 mil niños no tuvieron atención en salud adecuada por atenderla a ella de un cáncer terminal y eso es respeto por los demás, y el tercer punto solidaridad, yo debo tener absoluta claridad de que la salud va a depender de mi colaboración en todo momento porque eso es lo que me permita mantener mi salud después, porque en este momento es de mi solidaridad que estamos atendiendo a muchos que estaban enfermos sin yo estar enfermos, para mí son esos tres.

P14: 8. Transcripcion Jorge E. Robledo_ok.docx - 14:1 [Vamos a entrar a los valores, ..]  (30:31)   (Super)
Códigos:	[Valores : Valores que determinana el nivel de humanidad del SGSSS] 
No memos

Vamos a entrar a los valores, usted ha sido bastante crítico de la corrupción  en sus debates. Adela Cortina dice que cualquier sistema para llamarse humano debe cumplir con 5 valores, libertad, igualdad solidaridad respeto y dialogo, dice ahí en la cortina, mi pregunta va en este sentido. ¿En qué orden de importancia cree usted que debe dársele en un sistema de salud a estos valores?, ¿cree usted que debe darse en un sistema de salud?
JR: Todas estas son palabras que suenan muy bonita y suenan muy bien, el problema es que usted cuando a esas palabras le impone política que no funcionan así pues todo esto se vuelve en publicidad, propaganda frases huecas, si porque yo a eso le atravieso una frase que es de Friedman que dice "una solo hay una, una y solo una responsabilidad social de las empresas y su razón social, cual es la de hacer las cosas para aumentar sus utilidades"  entonces cuando usted al frente de estas cuatros palabras pone ganancia, pues ellos le dicen si libertad, si pero primero ganancia, igualdad si pero primero ganancia, solidaridad si pero primero ganancia. Entonces no hay nada que hacer, un sistema como estos puede tolerar el lucro en el mundo de las IPS, pero el lucro metido en la administración de los recursos y más en un país pobre, porque si usted tuviera un país súper rico donde la plata sobrara y usted puede despilfarrar, pero es que aquí las ganancias de las EPS y todas sus corruptelas se hacen reduciendo medicamentos, reduciendo personal médico, y en general de salud y reduciendo procedimientos quirúrgicos hospitalarios porque no tiene de donde más salir. Entonces todo esto, un ejemplo es la libertad de pasar de una EPS otras que eso no los ofrecieron en una cosa maravillosa, no se cumple yo le dije un día a Santamaría cuando era ministro de salud, óyeme ustedes porque no auditan estas empresas, porque el estado no sabe cuándo vale una aspirina y ellos son quienes deciden esa vaina, y no es que el sistema está diseñado para que la competencia entre las empresas el sistema se autocorrija por  la vía de que el ciudadano se sale de la mala empresa y se va para la buena. Nos tratan como imbéciles doctor. Y es así como funciona el sistema, entones robar este sistema es una ganga, entones todas estas palabras son muy bonitas, pero resulta que en manos de ellos no son un objetivo un propósito, sino una trampa y el discurso que ellos se echan todo el día es este, pero si uno va y mira al respeto de las colas de yo no sé cuántos o bueno todo lo que hay ahí. 

P14: 8. Transcripcion Jorge E. Robledo_ok.docx - 14:2 [Usted considera que en el sist..]  (32:33)   (Super)
Códigos:	[Valores : Valores que determinana el nivel de humanidad del SGSSS] 
No memos

 Usted considera que en el sistema  como esta no hay valores 
JR: Es que uno no le puede pedir a las hienas comportamientos de venaditos, entonces uno le puede pedir a un sistema que está diseñado para una cosa, no sé si usted me ha oído una frase que yo repito mucho, "es que el sistema de salud por este asunto del aseguramiento del  control del capital financiero, la ley 100 no es la ley para la salud de los colombianos, sino para el negocio de las EPS con la salud de los Colombianos", entones eso trastoca todo porque un gerente al final del año no le preguntan cuántos salvo ni nada de eso, si no doctor cuanto gano, y si el gerente muestra que salvo muchas vidas pero la EPS no dio los rendimientos que eran lo echan y punto

P15: 9. Transcripcion Julian Duran_ok.docx - 15:1 [Esta entrevista está dividida ..]  (73:75)   (Super)
Códigos:	[Valores : Valores que determinana el nivel de humanidad del SGSSS] 
No memos

Esta entrevista está dividida en tres partes, la primera parte es la visión de futuro que acabamos de terminar y ahora vamos a pasar a una segunda parte que usted toco, que es la corrupción, nosotros le llamamos los riesgos morales. Adela  Cortina dice que toda actividad para que sea llamada humana debe tener 5 valores, usted los tiene ahí en la ficha 6, que son:  libertad, entendido como poder actual sin interferencia ajena y participar en las decisiones en la sociedad en que vivo, igualdad frente a lo que yo considero importante, solidaridad el apoyo mutuo para nosotros acceder los unos y los otros a los servicios de salud sin  distingo, respeto es no compartir lo que el otro piensa pero respetar ese pensamiento, y dialogo no entendido como una negociación entre los actores si no como un elemento al dialogar y respeto y no cometer actos no éticos contra el sistema. Yo le quiero preguntar también está el derecho a la salud, la ley 1751 del 2015 consigno el derecho a la salud como un derecho fundamental que comprende el acceso a los servicios de salud de todas las personas de manera oportuna eficaz y con calidad. Hecho este preámbulo le voy a hacer 4 preguntas, primero que para que un sistema de salud sea humano debe practicar 5 valores libertad, igualdad, solidaridad, respeto, y dialogo. Entonces podría decirme en orden de importancia cuál de estos valores debe ser el primero, cual el segundo, cual el tercero, cual el cuarto y cual el quinto que debe tener el sistema nacional de seguridad social de Colombia.

JD: primero: Solidaridad, el segundo: respeto, el tercero seria la libertad, cuarto: igualdad y quinto seria el dialogo. 

P15: 9. Transcripcion Julian Duran_ok.docx - 15:13 [Pues el grado en que se están ..]  (79:79)   (Super)
Códigos:	[Valores : Valores que determinana el nivel de humanidad del SGSSS] 
No memos

Pues el grado en que se están expresando es muy bajo, se manifiestan en muy poco porcentaje.

P15: 9. Transcripcion Julian Duran_ok.docx - 15:14 [Pues serian todos los valores,..]  (83:83)   (Super)
Códigos:	[Valores : Valores que determinana el nivel de humanidad del SGSSS] 
No memos

Pues serian todos los valores, los cinco valores porque todos son complementarios y todos deben hacer parte del sistema de salud que este tenga en cuenta al individuo como ser humano. 


P15: 9. Transcripcion Julian Duran_ok.docx - 15:15 [Pues primero que todo porque e..]  (87:87)   (Super)
Códigos:	[Valores : Valores que determinana el nivel de humanidad del SGSSS] 
No memos

 Pues primero que todo porque es un sistema donde predomina la cuestión económica, entonces las EPS como prestadoras del servicio de salud están actuando bajo una lógica económica donde a ellas lo que les interesa es maximizar los ingresos, maximizar sus ganancias y minimizar los costos y eso implica entonces que no se le preste a las personas los servicio siguiendo estos cinco valores, muchos de los servicios que están dentro del sistema por ejemplo del POS no son prestados y al afiliado le toca instaurar demandas penales para poder que se le preste el servicio y de esa forma se estan violando los valores y tampoco se le prestan otros servicios básicos y el afiliado tiene que insistir permanentemente entonces vemos que es un sistema donde  las personas no  les interesan a los prestadores y tampoco  están considerando estos valore

P16: 10.Transcripcion Angela Tascon.docx - 16:1 [: para mirar los valores yo mi..]  (32:34)   (Super)
Códigos:	[Valores : Valores que determinana el nivel de humanidad del SGSSS] 
No memos

: para mirar los valores yo miro un riesgo moral, entonces principalmente considero que el sistema debe trabajar la parte de valores, entonces consulte a un autora que se llama Elizabeth Cortina, ella dice que todas las actividades para ser humana necesita 5 valores, libertad, igualdad, solidaridad, respeto y dialogo. Si usted fuera a organizar estos cincos valores en el sistema de salud, siendo para usted el más importante el primero que ponga y el menos importante el último en que parte los pondría.

AT: Yo creo que pondría de primero solidaridad, segundo respeto, igualdad, libertad y dialogo. Yo el dialogo de ultimo porque se mezclan mucho si yo soy solidario te respeto si yo te respeto reconozco que somos iguales, que tenemos libertad y ya todas estas juntas me facilitan el dialogo. 


P18: 11. Transcripcion Fabian Mendez.docx - 18:1 [: La segunda parte que trabaja..]  (37:43)   (Super)
Códigos:	[Valores : Valores que determinana el nivel de humanidad del SGSSS] 
No memos

: La segunda parte que trabajamos fue los valores, los trabajamos desde la doctora Cortina, ella dice que hay 5 valores que son fundamentales para que cualquiera actividad sea humana, la libertad, la igualdad, la solidaridad, el respeto y el dialogo. Yo se los paso y usted me dice cuál es la importancia que debe darse en el sistema de salud a estos valores, diciéndome primero el más importante y así hasta el quinto. 

FM: Yo colocaría de primero la solidaridad, luego respeto, igualdad, cuarto dialogo y quinto libertad.

PC: Tiene algún valor que usted quisiera agregar y que usted crea que sea importante y acá no este.

FM: Aquí no dice justicia. Porque igualdad es diferente a justicia, y es un asunto importante porque los que más necesitan y para tener mejor condiciones

P18: 11. Transcripcion Fabian Mendez.docx - 18:2 [: ¿Cómo se practican estos val..]  (45:47)   (Super)
Códigos:	[Valores : Valores que determinana el nivel de humanidad del SGSSS] 
No memos

: ¿Cómo se practican estos valores en el sistema actual, usted cree que se practican o no se practican?

FM: En principio hay una base de solidaridad, porque todos contribuimos a un sistema, creo que el respeto si es de los principios que más se violan, no hay respeto por la dignidad humana, no hay respeto por los principios fundamentales del derecho a la vida y al de la salud. Hay en principio libertad pero es una libertad cortada porque finalmente no hay ese llamado que se hizo de que supuestamente íbamos a lograr en una economía de mercado libertad, y que eso iba a influir en la calidad de los servicios, a la postre no se reflejan, tiene esos servicios pero no tiene otros. Entonces no creo que la libertad sea un principio que funciones. Y el dialogo se ha reducido a relaciones jurídicas más que todo en el sistema, hay que tener la capacidad de entablar demandas y de establecer mecanismos jurídicos para que se respeten sus derechos. Ahora con todo y todo de todos modos los mecanismos jurídicos son los que han permitido, que de alguna forma la gente defienda sus derechos. 

P19: 12. Transcripcion Fabio Osorio.docx - 19:1 [Ya salimos de la primera parte..]  (32:34)   (Super)
Códigos:	[Valores : Valores que determinana el nivel de humanidad del SGSSS] 
No memos

 Ya salimos de la primera parte, para entrar a los valores nosotros tomamos la teoría de Adela cortina que hablaba de la humanización, ella dice que para cualquier actividad sea denominada humana debe tener 5 valores que son libertad, igualdad, solidaridad, respeto y dialogo. Cuál es la importancia que debe dársele a los valores poniendo el que tú consideres el más importante de primero y así en orden hasta el quinto.

FO: Para mí el primero sería la igualdad es esencial porque le da la misma oportunidad a todos los pacientes de tener atención oportuna, que todos tengan independientemente del estrato social igual atención igual enfermedad. Yo creo que el respeto es fundamental el segundo, dialogo, libertad y solidaridad, para mí como psicólogo yo creo que en ese orden porque hay que respetar al paciente creo que debe haber una libertad para que el paciente decida si quiere la atención o no, debe haber una solidaridad porque es muy triste que nosotros no pensemos sino hasta que nos enfermamos, los otros no importan si están enfermos o no y el dialogo siempre tiene que estar entre paciente y personal de enfermería. 

P19: 12. Transcripcion Fabio Osorio.docx - 19:10 [yo creo que la misma ley 100 l..]  (38:38)   (Super)
Códigos:	[Valores : Valores que determinana el nivel de humanidad del SGSSS] 
No memos

yo creo que la misma ley 100 los nombra, pero acá en Colombia una cosa es lo que muestran los medios de comunicación y otra cosa lo que es, acá en Colombia no hay igualdad repito el ejemplo porque acá no es la misma enfermedad de un paciente que este clasificado sisben 1, a un paciente como el presidente que tiene su prepagada y loa tienden hasta en estados unidos, yo no creo que el presidente santos madrugue a pedir una cita ni madrugue a que le autoricen 10 veces un medicamente, acá no hay ese valor de igualdad yo creo que es inhumano porque hay gente que fallece en las casas cuando los llaman que los van a operar y cuando tienen la cita muchas veces lo hemos visto ya en el hospital no que pena ya lo enterramos. 

P22: 13. Transcripcion Maria Ines Pantoja.docx - 22:1 [Vamos a entrar a un terreno qu..]  (46:52)   (Super)
Códigos:	[Valores : Valores que determinana el nivel de humanidad del SGSSS] 
No memos

Vamos a entrar a un terreno que yo estudie con sacerdotes, hermanos maristas. Y para nosotros los valores se han perdido, entonces yo tome una autora que se llama Adela Cortina, ella dice que los valores son algo que son valiosos para las personas y dice que una cosa para que esa humana debe tener 5 valores libertad, igualdad, solidaridad, respeto y dialogo. Y le voy a hacer la primer pregunta acerca de valores, que veo que usted habla mucho de esta iniciativa. Entonces si usted tuviera oportunidad de ordenar los valores del sistema de salud siendo el primero el más importante, como ordenaría usted esos valores.

MP: Para mí el respeto primero, cuando yo soy capaz de respetar al otro, yo no cometo tantas barbaridades por el otro. El segundo la libertad, porque es un valor que nos lo da la constitución y me hace a mi ser persona y elegir por mí mismo. Luego la solidaridad, el cuarto pondría la igualdad y quinto el dialogo, no quitando importancia de ninguno.

PC: ¿Cuénteme porque primero pone el respeto y de ultimo el dialogo, porque en ese orden?

MP: Porque yo le decía que el respeto es lo básico, si yo respeto la persona le voy a dejar su libertad lo voy a tratar de igual a igual, leía yo que la gente famosa es una ilusión social porque todos somos iguales, todo ser humano tiene la misma capacidad de desarrollo que de pronto unos tienen las oportunidades que no tuvieron otros. Cuando yo soy capaz de respetarlo a usted me siento igual a igual, le dejo la libertad y soy solidaria con usted y en lo católico me siento que usted es mi hermano y no lo voy a atropella en nada y voy a tener enfocada mi vida que lo más importante es el ser humano. Mi papa me decía que el mejor regalo que se le puede hacer a otra persona es el respeto, y eso me ha marcado en la vida. Y si yo lo respeto a usted yo respeto su palabra, dialogo y soy capaz de asumir muchas cosas que usted me presenta y yo las respeto. 

P22: 13. Transcripcion Maria Ines Pantoja.docx - 22:3 [¿Estos valores se expresan en ..]  (54:68)   (Super)
Códigos:	[Valores : Valores que determinana el nivel de humanidad del SGSSS] 
No memos

¿Estos valores se expresan en la práctica en el sistema de salud?

MP: Empezando por el respeto que fue lo que yo hice en orden, la gente es totalmente irrespetada, le dicen no hay y ellos tranquilos y si no hay que puedo hacer yo, y yo les digo que pena pero su obligación es darme el medicamento. Ahí hay una falta de respeto, como así que puedo hacer yo si ustedes son los responsables de darme el medicamento, que ha sucedido como la gente va y va, ya las personas que atienden en ventanilla han empezado a tratar mal a los pacientes y hay pacientes que se alteran y tratan mal a la gente, tanto el respeto de los pacientes como el respeto hacia ellos. Le decía yo al ministro a mí porque me educaron porque si no yo le había dado un puño a unos cuantos, a uno le provoca, como será la gente que esta allá dele y dele. Yo le decía si a mí me hubieran educado si no fuera religiosa, yo estaría en una rebeldía aterradora. La Libertad, en la resolución 13436 de 1991 nos dan a nosotros el derecho a elegir libremente, creo que en la ley estatutaria quedo de sexto, nos dan el derecho a elegir libremente los médicos, las instituciones, y el personal de salud. Hace tres años le estamos pidiendo a la superintendencia de salud que las EPS  nos regalen la red de prestadores y es uno de los requisitos de habilitación y no tienen red, y la gente llega allá y le dicen váyase a tal parte, están irrespetando el derecho a la libertad, y le dicen a las personas solo tengo un médico, usted se imagina a los maestros que les digan solo tengo un especialistas para la cantidad de maestros que hay, entonces solo un especialista me obliga a ir allá. La igualdad ya hablábamos del régimen subsidiado y contributivo y cuando la persona es más vulnerable es la persona que más mal tratan, entonces ahí va la igualdad. Y la solidaridad eso si se ha perdido totalmente, porque si usted ve que están maltratando a otro y usted no es solidario con la persona. Imagínese que yo fui mucha ves a una EPS y había un ancianito haciendo cola de 85 años más o menos y la muchacha le decía es que usted como no me pone cuidado, toda la gente haciendo cola y nadie protesto por el señor, y yo le dije señorita, tenga la bondad de respetar al señor y le dije como usted quiera que el señor de 85 años tenga la audición que usted tiene, le dije puede ser su papa su abuelito. Y el viejito todo angustiado, entonces se formó una pelotera, y me llevaron a donde el gerente. Pero nadie de los que estaban en la cola fue capaces de ser solidarios con esa persona, podemos ver lo que le pasa a otra persona y no nos comprometemos con nadie, y yo creo que la solidaridad no es que la UPC pague a la UPC del otro. La solidaridad va más de eso, se trata de sentir a otro como mi hermano.

PC: El papa dijo una cosa muy interesante sobre eso, nosotros somos capaces de dar la limosna pero no somos capaces de darle la mano al mendigo, y el decía que solo le damos la limosna y no lo miramos, yo pienso que la iglesia estaba en mora de tener un papa más humano, y no solamente en la palabra si no en la práctica. 

MP: Es un religioso y entiende el sentido de la pobreza y de la humildad, y la iglesia tiene que seguir renovándose pero eso es algo largo

PC: el respeto, pero usted si me ha dicho que acá no respetan a nadie, en el sistema de salud.

MP: No atropellan totalmente a las personas, porque es un atropello eso de mandarlo a un lugar donde no le van a prestar el servicio, de hacerlo ir 10 20 veces a reclamar la misma cosa, eso es un irrespeto total hacia las personas, y lo que usted decía lo que hicieron ellos con nosotros los pacientes de no tener la gentileza de ir a esa audiencia, ellos mandaron el tal video ahí protegidos, y eso psicológicamente tiene su sentido, yo me siento más segura en mi ambiente de trabajo porque yo domino, pero ellos no salieron de su pedacito de tierra. 

PC: Pero no los estaban llamando cualquiera, los llamo la corte constitucional quien los llamo, si esos señores no le hacen caso a la corte constitucional. A los pacientes nos han respetado toda la vida.

MP: Ellos no respetan a nadie porque han tenido una posición dominante ante el sistema y es porque tienen el dinero, y el dinero hace que la gente tenga posición dominante, y ellos se sintieron seguros allá en sus oficina con sus escudo y sus cosas y yo acá estoy segura, pero cuando me voy a la calle a, la EPS con las personas soy una cualquiera y me tratan como una cualquiera. Entonces ellos no quisieron acercarse a nosotros. 

P23: 14. Transcripcion Mario Hernandez.docx - 23:1 [Profesor Mario, yo he tomado d..]  (44:46)   (Super)
Códigos:	[Valores : Valores que determinana el nivel de humanidad del SGSSS] 
No memos

Profesor Mario, yo he tomado de Adela la cortina para trabajar los valores usted trabaja la bioética, y dice en uno de sus textos que toda actividad para ser humana necesita 5 valores, que son libertad, la igualdad, la solidaridad, el respeto y el dialogo. Yo le quiero preguntar en qué orden de importancia cree usted que debe dársele a estos valores en un sistema de salud

MH: Para mi primero está la solidaridad y esta simplemente porque es el núcleo duro de expresión de algo que me parece fundamental que es la igualdad, solidaridad e igualdad como lo relaciono, si partimos de la base como hace Adela la cortina de un valor que permita efectivamente considerar la igualdad entre las personas ese valor no es otro que la dignidad, es un valor supremo, superior en el sentido de que es no transable es inmanente, para la condición humana, y eso es lo que permite sustentar una idea de igualdad entre los seres humanos, esa igualdad basada en la dignidad es lo que permite entonces ordenar las acciones humanas en principio en base a la solidaridad, que significa justamente una acción colectiva para lograr el máximo de igualdad. Ese es mi ordenamiento de valores, ahora la libertad estaría en un tercer momento en el sentido de que es algo que requiere todo un ejercicio que implica responder por los actos no solamente ejercer la libertad sino ejercer una autonomía con base en la responsabilidad por los actos y de ahí entonces se puede demandar respeto por las distintas posiciones, y cuando se asume una posición de respeto se puede hacer un diálogo, es decir, no hay forma de entrar a un dialogo si no se considera al otro como un igual con unos valores y con unos planteamientos que son en sí mismo valiosos  varios prima en primera instancia y que permite entonces entrar a discutir diferencias o similitudes y construir entonces acuerdos, de manera que la posición original de cada cual pueda ser transformable en medio del diálogo, es decir todos estos elementos son los que uno impulsaría como una cosa valida con cierta universalidad, el problema es que no son los valores que están predominando en el mundo contemporáneo al contrario, los valores del mundo contemporáneo están completamente puestos en una lógica distinta, donde el primer valor es la libertad, por ejemplo como se defiende en el principal país  que domina el orden global que se llama Estados Unidos, la libertad por encima de todo como el primero y único valor, del cual se derivan la propiedad en medida de que mi trabajo libre y mi libre empresa y mi iniciativa privada yo tengo entonces una propiedad derivada de mi trabajo que es un segundo valor que está por encima de todas las cosas, entonces en esa perspectiva de un liberalismo extremo que es esa un poco la lógica  neoliberal, basada más en  el mercado que en cualquier otro ordenador de esos valores pues todo eso se organiza a partir de la competencia entre seres libres e iguales, y a partir de la transacción, las titularidades, que permiten entonces las transacciones entre propietarios es lo que defiende Robert Nozick cuando habla de su tal Anarchy, state and utopia el estado debería solamente estar regulando los contratos entre libres e iguales y toda la distribución se haría a través del mercado, a través de las titularidades y los intercambios contractuales. Eso es un modelo absolutamente liberal que yo creo que no asume esta forma de organización de valores que yo he planteado, sino otra completamente distinta y eso es fundamental en la disputa en un sistema de salud

P23: 14. Transcripcion Mario Hernandez.docx - 23:2 [¿Usted cree que el sistema de ..]  (48:54)   (Super)
Códigos:	[Valores : Valores que determinana el nivel de humanidad del SGSSS] 
No memos

 ¿Usted cree que el sistema de salud esta guiado por lo que usted me acaba de decir, por el ordenamiento de que esta primero la libertad y segundo la propiedad?

MH: Si, es mi convicción y no solo eso sino que asume que el estado tiene que proteger primero eso, libertad propiedad y después hacer una vigilancia de los contratos, y lo máximo que podría hacer es una regulación de esos contratos cuando efectivamente produzcan grandes impactos en la sociedad, por ejemplo la regulación de la competencia entre aseguradores, que es el modelo clásico de la ley 100. De manera que todo lo demás viene por añadidura, la solidaridad se confunde con atender a los pobres, se confunde con una especie de subsidiaridad que es lo que sostiene la política del régimen subsidiado y todos los otros sectores con subsidio a la demanda de los pobres, pero que es lo que define quien merece y quien no, el hecho de ser pobre es decir, no tener propiedad suficiente para comprar en el mercado y ese le otorgan entonces supuestamente un merecimiento de un subsidio a la demanda por parte de los recursos públicos, un subsidio porque es pobre, no por igual dignidad sino por ser pobre, y eso está absolutamente claro hay que demostrar que se es pobre para merecer un subsidio, eso es una lógica neoliberal aunque tenga la regulación de la competencia  que es distinto a la lógica kantiana que tiene una perspectiva más social demócrata, menos monetarista y que es distinto a la perspectiva marxista y critica y anticapitalista de partir de la base que no debería haber siquiera explotación de un ser humano por otro ser humano, ni apropiación del trabajo conjunto construido por todos, es decir no debería siquiera haber propiedad privada que ese es el otro sistema de valores. Bueno es que hay que aceptar que hay varios sistemas de valores y esos sistemas de valores están en disputa en la contemporaneidad, y sobre todo la modernidad en todos los 400 años que llevamos de modernidad, están en disputa y hay otros valores ordenados de otras maneras en sociedades por ejemplo ,orientales o en propiedades aborígenes suramericanas o africanas, pues tiene que haber que reconocer que esos son otros valores, distintos a la modernidad liberal por ejemplo, vea que si uno quiere atar ese debate de valores con el sistema de salud existente tiene que comprender la diferencias, y tiene que ver los valores que ordenan de facto en la realidad del sistema para poder decir, bueno esto tiende a cambiar o tiende a consolidarse. 

PC: Usted cree que como están ahora los valores ordenados en la situación que usted dice orientado en el orden que usted da, libertad y propiedad, ¿seguirán a futuro?

MH: Yo creo que si, como están las cosas no veo una correlación de fuerzas que vaya a cambiar eso desafortunadamente y estoy luchando para cambiar ese estado de cosas, pero veo que es muy difícil hay una gran hegemonía cultural una cosa muy profunda que es ya como un consenso de dominación, nos parece ya natural que haya tanta riqueza acumulada en unos pocos, y tanta pobreza en tanta gente y creemos en una cosa como culturalmente aceptada que eso es por esfuerzo personal, Luis Carlos Sarmiento Angulo se volvió rico él solito por su esfuerzo personal y las gentes pobres que piden en la calle es porque no quieren trabajar. Entonces hay una serie de valores en nuestra cultura colombiana cotidiana, que es muy difícil de modificar, entonces eso me parecen restricciones reales que tenemos en el contexto actual y me parece que todavía no hay una discusión suficiente para cambiar eso, para pensar que eso no debería ser así, sino debería ser de otra manera.


P24: 15. Transcripcion Martha Balbuena.docx - 24:1 [yo tome a cortina que habla qu..]  (60:62)   (Super)
Códigos:	[Valores : Valores que determinana el nivel de humanidad del SGSSS] 
No memos

 yo tome a cortina que habla que toda actividad para ser humana necesita de 5 valores, libertad, igualdad solidaridad respeto y dialogo; yo me preguntaba como ordenamos estos valores en nuestro país en un sistema de salud, usted como los ordenaría en primer lugar el que usted considere ser el primero el segundo y así hasta el quinto. 

MC: Yo lo que digo es cuando miro la parte ética yo hablo de unos principios o valores, pero hablo de unos conceptos que son importantísimos dentro de la parte operativa y de la parte organizativa y es el concepto de responsabilidad y si usted mira el concepto de responsabilidad ahí están esos principios, yo hablo del concepto donde todos como equipo de salude debe estar abogando, por el enfermo o por la persona sana y sus derechos, hablo del concepto de trabajo en equipo la cooperación y trabajo el concepto de la solicitud y dentro de esos 4 conceptos ahí se mete todo, entonces en el concepto de solicitud lo que necesitamos es que cuando el paciente o la familia requiera un servicio eso sea oportuno, entonces dentro de la responsabilidad esta la igualdad, el primero para mi seria la igualdad pero dentro de esa parte de responsabilidad y dentro de la parte de trabajo en equipo esta esa parte de solidaridad. El respeto igual hay unos que están tan este, el de responsabilidad tendría respeto, la otra parte de ser solidario y la parte de igualdad y el dialogo entra dentro de la abogacía, si no hablo con los demás no me importan ellos. La libertad comprendería todo lo demás para mi sería el último, sin ella no podríamos hacer nada, pero tiene que estar sin perjudicar a los demás no entrar a libertinaje.


P26: 16. Transcripcion Senador Ospina.docx - 26:1 [PC: Senador yo he tomado a la ..]  (25:27)   (Super)
Códigos:	[Valores : Valores que determinana el nivel de humanidad del SGSSS] 
No memos

PC: Senador yo he tomado a la profesora Adela Cortina, ella dice que todo servicio humano si tiene 5 valores, libertad, igualdad, solidaridad, respeto y dialogo. ¿Usted en qué orden cree que un sistema debería tener estos valores?, siendo el primero el más importante para usted

SO: yo te voy a hacer una reflexión ahí que me parece importante que la tengas, en los modelos de salud yo creo que la libertad o la libre escogencia puede ser mal interpretada, porque no podríamos tener un modelo de salud sostenible, si a cada médico se le ocurre demandada lo que quiera, si a cada paciente se le ocurre exigir lo que quiera porque eso no estaría estandarizado y seria improcedente desde una ecuación económico y no sería sostenible en el tiempo. Por eso entonces bajo esa premisa de libertad yo la dejaría en quinto lugar, empezaría diciendo que toda la gente merece igualdad, que toda la gente merece respeto, que la solidaridad viene de la mano de quienes tengan más puedan financiar a quienes tengan menos, el dialogo como una herramienta fundamental, y la libertad entendida en los parámetros que le dije.


P28: 17. Transcripcion-Gustavo-Morales (1).docx - 28:1 [Vamos a hablar de los valores,..]  (56:58)   (Super)
Códigos:	[Valores : Valores que determinana el nivel de humanidad del SGSSS] 
No memos

 Vamos a hablar de los valores, porque para mí el problema más grave en la salud es la manera en cómo se fugan los recursos. Nosotros tomamos a Adela Cortina por la teoría de prospectiva social ella dice que deben hacer futuros humanos, y ella dice que son 5 valores para que cualquier actividad sea humana: igualdad, libertad, solidaridad, respeto y dialogo. Cuál es la importancia que debe dársele a estos 5 valores ordenando de uno a cinco como primero el que considere más importante y así sucesivamente hasta llegar a 5. 

GM: Entendiendo como actividad el sistema de salud, que bonita pregunta me pusiste a pensar. Entonces yo diría lo siguiente, el primer valor sería el de igualdad porque la salud es un derecho fundamental no debe haber distinciones por lugar de origen, ni raza, ni ingresos. Yo diría que el segundo es solidaridad, si queremos un sistema igualitario definitivamente unos tienen que ser solidarios con otros y unos debemos que aportar más que los otros. El tercero diría que es el dialogo, porque en un sistema complejo como el nuestro dónde hay actores de distinto origen hay que ponerse de acuerdo en lo fundamental. El cuarto yo diría que es el respeto, no sé qué se refiere con respeto.

P28: 17. Transcripcion-Gustavo-Morales (1).docx - 28:2 [Si hay dialogo, uno debe respe..]  (60:62)   (Super)
Códigos:	[Valores : Valores que determinana el nivel de humanidad del SGSSS] 
No memos

Si hay dialogo, uno debe respetar lo que piensa el otro, si tu dialogas no es para imponer. 

GM: y yo te diría que el valor menos importante en un sistema de salud generalizado con cobertura universal es el de libertad. Es más, me atrevo a decir una herejía, tenemos que olvidarnos de la liberta en el sistema de salud, tenemos que olvidarnos de la libertad de los pacientes y de los médicos en la salud, ósea esta vaina de que los médicos puedan prescribir lo que quieran y eso lo paga el sistema eso se tiene que acabar. Yo creo que la libertad acá si es antípoda de igualdad, a todo el mundo se le tiene que decir vea el sistema le cubre esto, si quiere algo más páguelo de su bolsillo. El dialogo respetuoso y solidario debe permitirnos crear un pacto social de lo que vamos a cubrir, pero fuera de eso cual libertad papito. de la misma manera que la policía te cuida de los delincuentes, pero no de los tropezones, no le podemos pedir todo al sistema porque nos vamos a morir, yo creo que es el orden, que bonito necesito leer ese texto de Adela

P29: 18. Transcripcion Carlos Hernandez.docx - 29:12 [Primero solidaridad, segundo i..]  (57:57)   (Super)
Códigos:	[Valores : Valores que determinana el nivel de humanidad del SGSSS] 
No memos

Primero solidaridad, segundo igualdad, tercero libertad, cuarto cuarto respeto y finalmente el diálogo. 

P30: 19. Transcripcion Felipe Garcia.docx - 30:6 [Yo considero que el primer val..]  (31:31)   (Super)
Códigos:	[Valores : Valores que determinana el nivel de humanidad del SGSSS] 
No memos

Yo considero que el primer valor de un sistema de salud debe ser la igualdad, seguido del respecto, como tercero el diálogo y finalmente la solidaridad y la libertad.


P31: 20.Transcripcion Hellen Mendoza.docx - 31:20 [HM: Porque la IPS tiene que te..]  (82:83)   (Super)
Códigos:	[Valores : Valores que determinana el nivel de humanidad del SGSSS] 
No memos

HM: Porque la IPS tiene que tener un actuario usted tiene información usted tiene cuántos años lleva una población independientemente de que sea EPS, o perca pita, usted le traen una información de un actuario que no corresponda con la suya entonces la información no es real y si no es real imposible hacer una nota técnica sobre esta base. Pero yo a donde iba, que yo estaba hablando de los pagos y luego nos devolvemos a su punto puede suceder cuando hablábamos de los giros directos y que las EPS están influyendo en los giros directos, yo percibo que hay una orientación de la EPS para pagarles a las IPS que tienen capitas y otro tipos de pagos prospectivos y las personas que trabajan por actividad no recibe plata, entonces a donde llevan a las IPS; yo necesito flujo de efectivo yo me meto a trabajar con usted modelos de riesgo compartido porque la única manera que tengo de garantizar mi flujo de cartera, entonces si yo hoy trabajado un modelo de pago global prospectivo como sea una capital tradicional, ajustada un PGP, un pago por desempeño, un paquete, el que usted quiera o combinación de los mismos porque el universo es tan grande que usted puede trabajar uno solo lo puede combinar para hacer el negocio que para usted sea el más rentable, a mí me pagan a los 30 días, es más si yo convenio el negocio me lo pagan por adelantado como una cápita tradicional si trabajo por evento, pueden pasar 6 meses hasta que yo reciba mi plata entonces antes del giro directo venia y era directo, ya no tanto, ahora la IPS dice espere, tengo que garantizar el pago de las IPS que me garanticen el modelo de riesgo compartido, lo que sobra es para pagar las IPS que trabajan por actividad. Entonces todas las IPS necesitan migrar a unos modelos de contratación en donde el riesgo sea compartido, ventajas y desventajas si nos devolvemos al primario de las teorías agencia principal, que ventaja le veo yo y yo soy de la prestación la ventaja del pago por evento es que usted le paga todo lo que yo hice si me paga una apéndice me la paga, si yo no pues no me la paga el problema es que yo vivo de cada una de las actividades que haga, entonces puede suceder que la IPS genere una mayor necesidad y estimule la utilización y entonces usted tiene acá un paciente que actúa como principal que no le importa no tiene por qué velar por la racionalidad de los servicios porque a nadie le está cobrando nada a  él le están cobrando la misma cuota moderadora o el mismo copago por una o diez cirugías, y el en su cabeza dice que entre mejor le hagan es mejor para el porqué lo van a curar y por allá actúa la EPS porque dependiendo de cómo usted se ponga van a cambiar y quien es el agente y quien el principal porque esto es una relación donde hay 3, hay un cliente pagador un cliente no pagador y un prestador del servicio, yo puedo estimular el sobre uso le pudo decir a un paciente venga cada 8 días, y cada 8 días le hago un elecardiograma y el lapicero lo puede todo, yo justifico bien lo que estoy haciendo y eso nos ha llevado a un principio que le ministros dice y yo lo comparto plenamente y es que hoy tenemos un sistema que se está colapsando porque el lapicero del médico es lo que esta llevando que los sobrecostos sean absurdos, eso es verdad. La ventaja para la IPS es que no tiene ningún tipo de transferencia de riesgo , la desventaja es que no recibe la plata y que tiene glosas, tiene devoluciones, y tiene una cantidad de proceso administrativos que son tan costosos que al final lo que al principio lo que pudo haber sido visto como una ventaja financiera se convierte en una desventaja porque el costo de atención se me eleva demasiado, me sale más caro radicar una factura que lo que realmente me voy a ganar por hacer un procedimiento. El modelo de pago global prospectivo cualquiera que sea el que usted diseñe a usted le están pagando y el concepto macro es garantizar la salud, y va muy de la mano con la política PAIS y el modelo MIAS, teóricamente a usted que le pagan le pagan por mantener la población sana cuando está haciendo modelos de coldecapita ojala fueran capitas ajustadas donde no le trasladen los dos tipos de riesgo y cuando este trabajando en niveles superiores a usted le pagan por atender ciertas patologías en una población donde usted tiene todo un análisis actuarial que le va a decir para donde va eso. Eso que le está diciendo a usted señor IPS, usted tiene que ajustar su costo tiene que ajustar su gasto y disminuir al máximo las estancias no necesarias porque si se devuelve al evento, si yo tengo las camas desocupadas y mañana no tengo con que llenar la cama, le puedo dar a mi paciente un día más de estancia el paciente no se pone bravo y asume si lo dejo un día más e porque necesita estar y no necesariamente se tiene que quedar, probablemente lo estoy haciendo para ganar un día más de hospitalización y ese día mas de hospitalización la EPS después va a venir como me lo glosa, ellos van a tener todo un equipo administrativo y yo también para defender la glosa y para decir que efectivamente el día de glosa es pertinente. En un buen modelo de pago global prospectivo que es hacia donde el ministro quiere orientar el sistema y es muy de la mano con el desarrollo que se ha hecho en canada, todo el mundo pone un poquito la EPS sigue siendo el garante del aseguramiento porque no traslada el 100%del riesgo, la IPS asume una parte del riesgo, cosa que no se ve en los pagos por evento, el paciente debe garantizar su autor cuidado y va a tener todo lo que se necesita para tal, eso con que viene con el MIAS y toda la cultura del autocuidado y todo el manejo que se le va a dar a la medicina familiar de lo que se espera de eso, y se va a controlar el costo al controlar el costo teóricamente no deberíamos tener unos problemas de sobre iniciación que ha conllevado que la UPC sea insuficiente. Lo otro que es absolutamente necesario y para allá va el modelo es más ya está andando hacia allá es se acaba el post deja de existir y como deja de existir el post, sencillamente lo que no este en el listado no existe y como no existe no se puede tutelar, cuando se sanciona ya la ley de la ley estatutaria en salud la ley dice que la salud es un derecho, recuérdese que antes el concepto era diferente y antes tenía que en tutelar el derecho la vida el derecho a la integridad el derecho a la salud mental para poder llegar a la cobertura del servicio y que le garantizaran a uno algunas cosas en salud, hoy no ya no tiene que hacerlo es su derecho pero su derecho esta sanscrito y lo que no está acá se convierte en gasto de bolsillo
PC: Pero ya el derecho lo redujeron al acceso, al acceso como oportunidad y calidad y a qué servicio porque hay excepciones.

P31: 20.Transcripcion Hellen Mendoza.docx - 31:22 [HM: Igualdad, solidaridad, lib..]  (118:118)   (Super)
Códigos:	[Valores : Valores que determinana el nivel de humanidad del SGSSS] 
No memos

HM: Igualdad, solidaridad, libertad, dialogo y respeto.  Pero es difícil ordenarlos

P31: 20.Transcripcion Hellen Mendoza.docx - 31:24 [PC: Cual es el nivel de confia..]  (124:126)   (Super)
Códigos:	[Valores : Valores que determinana el nivel de humanidad del SGSSS] 
No memos

PC: Cual es el nivel de confianza entre los actores del sistema general de seguridad social.

HM: Ninguno.


P32: 21.Transcripcion Oscar Gutierrez.docx - 32:16 [OG: El más importante seria la..]  (72:72)   (Super)
Códigos:	[Valores : Valores que determinana el nivel de humanidad del SGSSS] 
No memos

OG:  El más importante seria  la igualdad, seguido de la libertad, el respeto, continuaria el dialogo y finalmente la solidaridad. 

______________________________________________________________________
